# The Role of the Private Sector in the COVID-19 Pandemic: Experiences From Four Health Systems

**DOI:** 10.3389/fpubh.2022.878225

**Published:** 2022-05-27

**Authors:** Lauren J. Wallace, Irene Agyepong, Sushil Baral, Deepa Barua, Mahua Das, Rumana Huque, Deepak Joshi, Chinyere Mbachu, Baby Naznin, Justice Nonvignon, Anthony Ofosu, Obinna Onwujekwe, Shreeman Sharma, Zahidul Quayyum, Tim Ensor, Helen Elsey

**Affiliations:** ^1^Dodowa Health Research Centre, Research and Development Division, Ghana Health Service, Accra, Ghana; ^2^Public Health Faculty, Ghana College of Physicians and Surgeons, Accra, Ghana; ^3^HERD International, Kathmandu, Nepal; ^4^ARK Foundation, Dhaka, Bangladesh; ^5^Leeds Institute of Health Sciences, Nuffield Centre for International Health and Development, University of Leeds, Leeds, United Kingdom; ^6^Department of Economics, University of Dhaka, and ARK Foundation, Dhaka, Bangladesh; ^7^Health Policy Research Group, The College of Medicine, University of Nigeria, Enugu Campus, Nsukka, Nigeria; ^8^Centre of Excellence for Urban Equity and Health, Brac James P Grant School of Public Health, BRAC University, Dhaka, Bangladesh; ^9^Department of Health Policy Planning and Management, School of Public Health, College of Health Sciences, University of Ghana, Legon, Ghana; ^10^Ghana Health Services, Accra, Ghana; ^11^Department of Health Sciences, University of York, York, United Kingdom

**Keywords:** COVID-19, private sector, Ghana, Nigeria, Bangladesh, Nepal, government, policy

## Abstract

As societies urbanize, their populations have become increasingly dependent on the private sector for essential services. The way the private sector responds to health emergencies such as the COVID-19 pandemic can determine the health and economic wellbeing of urban populations, an effect amplified for poorer communities. Here we present a qualitative document analysis of media reports and policy documents in four low resource settings-Bangladesh, Ghana, Nepal, Nigeria-between January and September 2020. The review focuses on two questions: (i) Who are the private sector actors who have engaged in the COVID-19 first wave response and what was their role?; and (ii) How have national and sub-national governments engaged in, and with, the private sector response and what have been the effects of these engagements? Three main roles of the private sector were identified in the review. (1) Providing resources to support the public health response. (2) Mitigating the financial impact of the pandemic on individuals and businesses. (3) Adjustment of services delivered by the private sector, within and beyond the health sector, to respond to pandemic-related business challenges and opportunities. The findings suggest that a combination of public-private partnerships, contracting, and regulation have been used by governments to influence private sector involvement. Government strategies to engage the private sector developed quickly, reflecting the importance of private services to populations. However, implementation of regulatory responses, especially in the health sector, has often been weak reflecting the difficulty governments have in ensuring affordable, quality private services. Lessons for future pandemics and other health emergencies include the need to ensure that essential non-pandemic health services in the government and non-government sector can continue despite elevated risks, surge capacity to minimize shortages of vital public health supplies is available, and plans are in place to ensure private workplaces remain safe and livelihoods protected.

## Introduction

Much of the initial impact of COVID-19 was on urban populations; the United Nations estimated that perhaps 95% of cases occurred in urban areas (1). The livelihoods of the urban poor, particularly migrant workers and those working in the informal sector who are reliant on a daily-wages, were severely undermined by stringent lockdowns (2). In urban settings, the population depend on the private sector for both health-care and their livelihoods. Evidence from Low and Middle Income Countries (LMICs) consistently shows private providers are more likely to be used by urban citizens, particularly women, than their rural counterparts (3, 4). This is particularly true for poor households in urban areas who lack accessible public health-care and so are dependent on informal drug shops and pharmacies (4–6). Any disruption to the sector will have a substantial effect that is likely to be amplified for poorer communities. The World Health Organization's recent action plan to engage the private sector in the COVID-19 response highlights the global recognition of this issue (7).

The private health-care sector in LMICs is characterized by diversity ranging from informal medicine sellers to services run by Non-Governmental Organizations (NGOs), and for-profit specialist hospitals (8). Across all four countries, urban populations rely heavily on the private sector for health-care, for their livelihoods and provision of services and utilities. For example, on average in the four countries more than a fifth of deliveries in urban areas are in for-profit private facilities as compared to around 11% in rural areas (9–12). Greater urban reliance on the private sector for other reproductive, maternal and child health services is also recorded (3). Urban households are also much more dependent on private sector employment than rural areas where family agriculture and small businesses are common; International Labor Organization (ILO) data suggest, for example, that urban workers are more than twice as likely as rural workers to be private sector employees across the four countries (derived from ILOSTAT ilostat.ilo.org/data/).

A key lesson from the Ebola outbreak of 2014-16 in West Africa was the need for all actors within the health system, including the private sector to be mobilized in the response (13). While previous research has focused on private sector health-care providers, the COVID-19 response has highlighted the need to understand the role of private actors outside the health sector and created a demand for innovative strategies and partnerships between the public and private sector (14). At the same time, the pandemic has drawn attention to weaknesses in government's ability to plan and regulate private sector activity (15). Given the disproportionate vulnerability of urban populations to COVID-19 and its social and economic impacts, understanding how to strengthen urban governance to deliver an effective response is vital.

We assess the way in which the private sector has acted in response to COVID-19 and seek to understand the Government's reaction to this response. We present findings of a policy and media review of the private sector response to the pandemic and government's interaction with this response in urban settings in four LMICs: Ghana, Nigeria, Nepal and Bangladesh. The private sector response was identified as a major theme a larger study undertaken in each country on the COVID-19 response in urban areas. The study was undertaken through a collaboration between CHORUS and CATALYSE projects. CHORUS (Community-led Responsive and Effective Urban Health Systems) is a research programme consortium focusing on urban health systems in the four study countries. CATALYSE (COVID-19 in West and Central African Health Systems) focuses on policy and systems responses to COVID-19 in West and Central Africa. The differences in rates of urbanization, disease burden and health system structures between the four countries provide valuable insights of relevance to rapidly growing cities across Africa and South Asia.

We focus on two related questions.

1. Who are the private sector actors (profit and not for profit) who have engaged in the COVID-19 first wave response and what was their role?
2. How have national and sub-national governments engaged in, and with the private sector response and what have been the effects of these engagements?

In the next section we describe the overall research methods used in the study followed by a results section that presents evidence on each research question. The final section discusses the results in the context both of the ongoing pandemic and future responses to public health emergencies. The short period of the research and rapidly evolving nature of the pandemic means that the accompanying recommendations are necessarily tentative.

## Methods

### Study Design

The study design was a multiple case study of four low and middle income countries–Bangladesh, Ghana, Nepal and Nigeria. The case definition was “private sector engagement in national and sub-national governments' initial COVID-19 response.” Sources of data were from a desk review of media reports and policy documents. A qualitative document analysis of media reports and policy documents was conducted in each of the four countries. Researchers reviewed, examined and interpreted data to gain meaning and empirical knowledge of the response to the first wave of COVID-19. The method for data collection, review and analysis followed the steps of document analysis recommended by Kayesa et al. (16).

### Search and Retrieval Process

This article draws on a subset of information from a larger study that undertook detailed policy and media review to understand local governments' response to the first wave of the pandemic (17). The time period of the review begins on the 30^th^ January 2020, when the WHO announced COVID-19 as a Public Health Emergency of International Concern and ends on 30^th^ September 2020 (18). All four teams retrieved news reports from websites of radio and televisions stations, and news agencies. A common list of research questions and search terms were agreed across the teams and written up as a concept note prior to the country work. Teams developed a list of keywords based on this list focusing on extracting documents related to the response to COVID-19 in urban areas together with lists of international and local news agencies to search (Table 1). The lists prioritized reputable newspapers and online news agencies with a large readership. News portals and online versions of paper based newspapers were chosen to aid searching data and extraction. In Ghana, the team used the Factiva (Dow Jones) database while the Nigeria team also undertook a general search using google scholar. News outlets publishing in English and also the national language (Nepali and Bangla) were included.

**Table 1:** Media outlets and policy stakeholders covered and search terms used.

	**Media sites**		**Policy stakeholders (government and non-government)**	
	**Media title**	**Search terms**	**Website of government ministries and non-government agencies**	**Search terms**
**Bangladesh**	The Daily Star, The Business Standard, Dhaka Tribune, Bangla Tribune, Prothom Alo bdnews24.com, Sarabangla.net, Dainikazadi Somokal, Financial Express, New Age Bangladesh, Risingbd.com, The Independent, Desh Review, New Age, Orthoshuchok, The Daily Sun, The Daily Ittefaq	∙ Keywords used individually: COVID-19, Urban, Urban Poor, Urban area, Slum, Health, Health Service, Healthcare, Social Distance, Lockdown, Vaccine, Mask, Sanitizer, Handwash ∙ Keywords used in combination: Travel, Flight, Education, Urban, Urban Poor, Urban area, Slum, Health, Health Service, Healthcare, Vaccine.	Civil Aviation Authority of Bangladesh, Directorate General of Health Service, Ministry of Public Administration, Ministry of Foreign Affairs, Ministry of Disaster, Management and Relief, Bangladesh Parjatan Corporation, Ministry of Agriculture, Dhaka Stock Exchange, Supreme Court, Asian Development Bank, NGO Affairs Bureau, Ministry of Education, Coxs Bazar District Office Administration, Ministry of Power, Energy & Mineral Resources, Biman Bangladesh, Airlines, Bangladesh Garment Manufacturers and Exporters Association (BGMEA)	∙ Keywords used individually: COVID-19, Urban, Urban Poor, Urban area, Slum, Health, Health Service, Healthcare, Social Distance, Lockdown, Vaccine, Mask, Sanitizer, Handwash ∙ Keywords used in combination: Travel, Flight, Education, Urban, Urban Poor, Urban area, Slum, Health, Health Service, Healthcare, Vaccine.
**Ghana**	Daily Graphic, Daily Guide, Citi News; My Joy Online. Factiva database used to facilitate search and extraction of articles.	Articles that contained Covid* or corona* in the title or in the lead paragraph	Ministry of Information; Ghana Health Service; Ministry of Health; Ministry of Local Government and Rural Development; Ministry of Finance; Ghana Statistical Service; COVID-19 National Trust Fund; Ghana COVID-19 Private Sector Fund; UNICEF; United Nations; United Nations Development Program; UNFPA; USAID; World Bank; WHO;	COVID-19, COVID, coronavirus, Ghana
**Nepal**	The Kantipur Daily, Onlinekabar, The Himalayan Times	COVID, Corona virus, Pandemic, Epidemic, Emergency, Crisis, Quarantine, Response, Isolation, Health services, PCR test, Rapid Diagnostic Kit (RDT), Ministry of Health and Population, Health Facilities, Hospitals, Lockdown, Travel Restriction	Government of Nepal, Ministry of Health and Population, Health Emergency and Disaster Management Unit (HEDMU) and Health Emergency Operation Center (HEOC) Department of Health Services, Epidemiology and Disease Control Division (EDCD) Government of Nepal, National Public Health Laboratory Ministry of Federal Affairs & General Administration Ministry of Home Affairs Public Health Update	COVID, Corona virus, Pandemic, Epidemic, Emergency, Crisis, Quarantine, Response, Isolation, Health services, PCR test, Rapid Diagnostic Kit (RDT), MoHP, Health facilities, Hospitals, Lockdown, Travel Restriction
**Nigeria**	Vanguard, Punch, The Nation, Premium Times, Daily Trust, This Day, The Guardian, Daily Post, Sun, Business Day, Tribune, Independent; Sahara Reporters, Pulse Nigeria, Observer Research Foundation, Human Rights Watch, Nigeria Watch, Africa Times, Africa Newsroom, The Cable, Ripples, Devex, Radio Nigeria. Other online news: WHO-AFRO, All Africa, Save the Children	COVID-19 OR (COVID, coronavirus); Nigeria OR (Enugu, Anambra, Onitsha); Federal government OR (State government, Local government); Response OR (policy, guideline, intervention, strategy, plan); Urban areas OR (city, metropolis)	Federal & State Ministries of Health; Nigeria Center for Disease Control; Federal Ministry of Education; Presidential Task Force on COVID-19 Websites of non-government agencies–UNDP, UNICEF, WHO-Nigeria, Plan Nigeria, KPMG, Christian Aid WhatsApp groups –Nigeria Health Economics Association; Network of Emerging Leaders in Health Policy and Systems	COVID-19 OR (COVID, coronavirus); Nigeria OR (Enugu, Anambra, Onitsha); Federal government OR (State government, Local government); Response OR (policy, guideline, intervention, strategy, plan); Urban areas OR (city, metropolis)

For the policy review, each team compiled lists of government ministries, departments and committees involved or likely to be involved in the COVID-19 response. This included committees and agencies newly established to coordinate the response to the pandemic. A further list of donors and multi-lateral organizations (e.g., UN agencies) and NGOs was also compiled. Each website was searched to extract strategy and policy documents, protocols, government orders, reports and minutes of meetings related to COVID-19 strategy and response. The research teams all had considerable experience and knowledge of the health systems within their country contexts. Several senior team members were engaged in the COVID-19 response at policy level in their respective countries. This highly embedded nature of the research teams helped to ensure an appropriate and thorough search and retrieval process for both media and policy documents (16).

### Screening, Data Coding and Extraction

Over the period of the study, 48,936 media articles and 260 policy documents were retrieved. Eligibility of references for inclusion was determined by a quick scan of the media report or the executive summary to determine if mention was made of issues related to the contribution of the private sector in the pandemic and roles of national and local authorities and other stakeholders in influencing these contributions. We consider within the private sector private-for-profit providers, ranging from small informal providers to large corporations, as well as non-government organizations (8). All retrieved documents (media and policy) were recorded and labeled using an excel spreadsheet or word document table. Following screening of 48,936 media reports, we found 44,515 items to be irrelevant or duplicates.

The remaining 4,421 media reports together with the 260 policy documents (all of which met the inclusion criteria) were used in the analysis.

### Data Analysis

Data were extracted from the included media reports and policy documents under each research question. These data were then grouped into country-specific themes and country level reports were produced, and are available on the research programme website, chorusurbanhealth.org (19–22). The initial reports were shared and discussed among the research teams to identify areas of commonality. From this process, the role of the private sector emerged as a common and important phenomenon within the COVID-19 response in urban areas across the four consortium countries. The team identified the two research questions specific to the private sector which form the basis for this paper.

**Figure 1:**
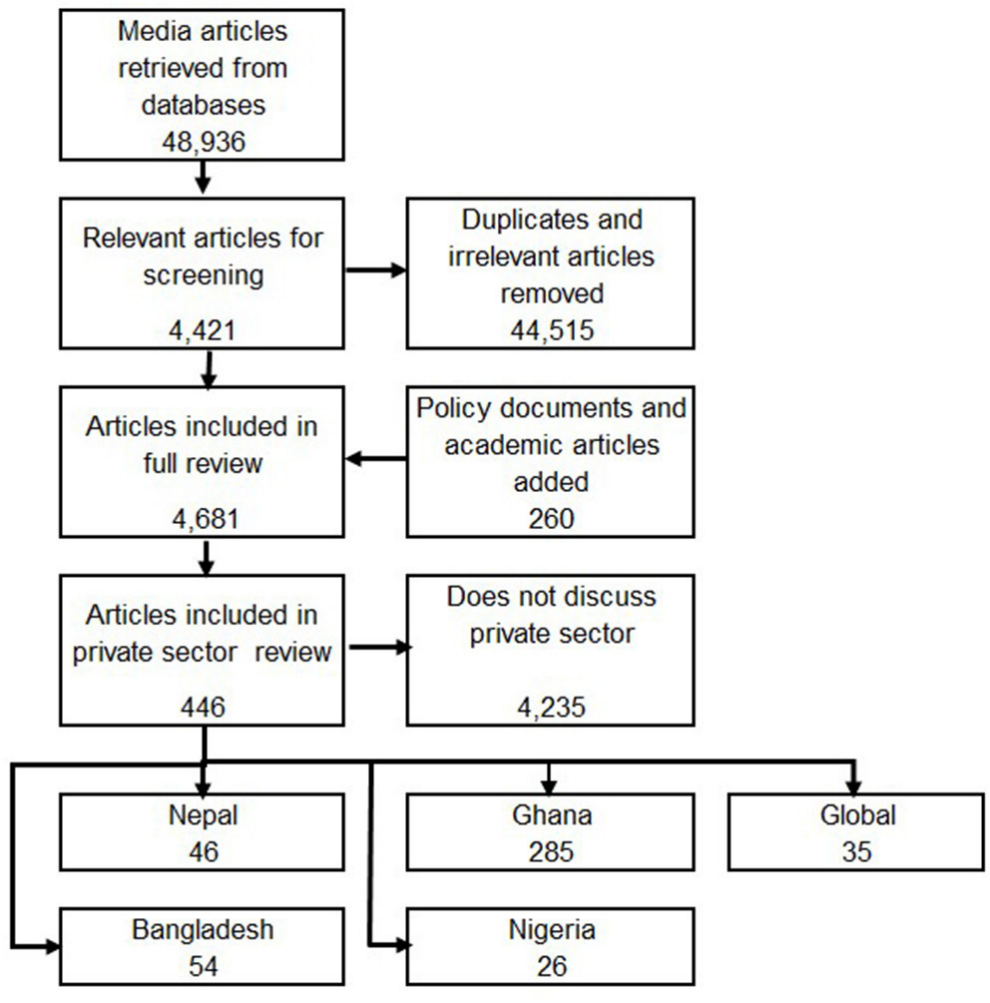
PRISMA flow diagram of extracted references (main study and private sector review).

Following identification of these research questions, a core team reanalysed data related to the private sector to fully describe and offer interpretations to answer our research questions. A total of 446 documents were identified that have a focus on the private sector including 54 from Bangladesh, 285 from Ghana, 46 from Nepal, 26 from Nigeria while 35 have a multi-country focus. Media reports (newspapers and other online media sites) made up 361 of the documents identified, there were 40 policy documents and 45 academic or statistical resources (Figure 1). A larger number of documents were generated in Ghana because of the use of the Factiva search engine to search media reports. Data were analyzed manually and first sorted by research question looking for themes, commonalities and contrasts. Despite the variation in numbers of documents extracted for each country, the same themes, commonalities and contrasts emerged from analysis of this data. Following the initial sorting, a common thematic framework was developed for this second-stage analysis and shared with all country teams. Following an iterative process where a team of two reviewers (MD, TE) applied the framework and consulted with partner teams, the following key themes were used to structure the analysis: (i) direct public health response; (ii) protecting livelihoods and relief; (iii) private sector service adaptation; (iv) public-private partnerships; (v) contracting; and (vi) regulation.

The team summarized the main findings by theme and sub theme with each theme linked to representative referenced examples. To ensure that the emerging themes resonated across the different contexts, sub-themes were only included when they applied to at least three countries.

## Results

RQ 1 Who are the private sector actors and what has been their role in the COVID-19 response?

The private sector in all four countries includes private health service providers, private business and industry players. Private health service providers found included private self-financing (for profit) health care providers such as hospitals, clinics and laboratories, private not for profit organizations such as Non-Governmental Organizations (NGOs) and Faith-Based Organizations (FBOs) and mission facilities. The extracted evidence suggests that the private sector in every country has played three main roles in the COVID-19 response (Table 2). First, the private sector has had a substantial function in directly supporting the public health response, secondly in efforts to protect livelihoods and provide relief to the vulnerable and thirdly in private sector service adaptation in response to the pandemic.

**Table 2:** Types of private sector participation in the COVID-19 response.

	**Ghana**	**Nigeria**	**Bangladesh**	**Nepal**	
**RQ 1 Who are the private sector actors and what has been their role in the COVID-19 response?**					
**1.1 Direct public health response**	Contribution to national funds	All types of private organizations to National COVID-19 Trust Fund and COVID-19 Private Sector Fund	All types of private organizations to Coalition Against COVID-19 (CACOVID)	All types of private organizations to Prime Minister's Relief and Welfare Fund	All types of private organizations to Coronavirus Prevention, Control and Treatment Fund
Facilities for quarantine, isolation and treatment	Faith-based organizations and hotels; Conversion of churches and hotels into isolation centers; COVID-19 Private Sector Fund construction of a 100-bed infectious disease center	CACOVID Fund Construction & equipping of medical facilities	Conversion of large private hotels into isolation centers; Business association Construction of a 50-bed field hospital	Conversion of spaces in hotels, school buildings and stadia into isolation centers; NGOs and private companies funding beds in public isolation wards	
Logistics for infection prevention, control and case management	All types of private organizations financial or in-kind donations of logistics for the public health response; Telecomms; provision of SIM cards and data bundles to National Security and COVID-19 Response Teams for use by contact tracers; Private company national mass disinfection exercise	All types of private organizations: financial or in-kind donations of logistics for the public health response	All types of private organizations made financial or in-kind donations of logistics for the public health response; Pharmaceutical company supplemented government pre-funding to the Serum Institute of India to ensure priority delivery of vaccines	All types of private organizations made financial or in-kind donations of logistics for the public health response	
Risk communication	All types of private organizations to campaigns using media to circulate safety tips and promote emergency contact numbers; Telecomms Zero rating of government websites	NGOs peer-to-peer, door-to-door education and distribution of flyers in slums and informal settlements	Telecoms toll-free line for subscribers to obtain information on COVID-19 from public health organizations	Telecomm companies help spread awareness of COVID-19 and conducted hotline services.	
**1.2 Protecting livelihoods**	Food relief and financial assistance to households	COVID-19 Private Sector Fund Feed a Kayayo initiative provided meals for head-porters during partial lockdown. Fund for distribution to the vulnerable, public agencies, or directly to communities. Private foundation Nkosuo program, a public private partnership supported enterprises with grants and soft loans, with a specific focus on assisting businesses in the informal sector owned by the vulnerable	CACOVID funding for distribution of essential foods to vulnerable households	Private foundations, companies, NGOs, individuals provided food aid to daily wage laborers and vulnerable families. NGOs financial assistance for low-income families in urban slums and remote areas; Private bank gave option for clients to digitally donate for the poor and financial support to more than 7,000 migrants returning from abroad. Financial relief packages	Private companies provided commodities to daily wage laborers
**1.3 Private sector service adaptation**	Reducing services in response to the threat of COVID-19		Private health facilities reduced services to patients presenting with COVID-19 symptoms in the early stages of the pandemic	Private health facilities reduced services to patients presenting with COVID-19 symptoms in the early stages of the pandemic	Private hospitals refused to admit COVID-19 patients in the early stages of the pandemic.
Adjusting services to promote preventative protocols	Private schools delivered online distance learning in private schools; Private companies introduced measures for workplace safety, including remodeling services	Private schools provided online distance learning in private schools;	Private schools provided online distance learning in private schools; Private companies introduced measures for workplace safety, including remodeling services	Private schools provided online distance learning in private schools; Private companies introduced measures for workplace safety, including remodeling services	
Adjusting production processes and services to respond to new market opportunities	Private companies diversified to manufacture of PPE, and products for hand hygiene such as sanitisers, as well as drugs for managing COVID-19 such as hydroxychloroquine. Some private health facilities restructured to provide medical services to COVID-19 patients; some involved in the national treatment network. Private laboratories restructured services to provide COVID-19 testing following accreditation	Private companies diversified to manufacture PPE, and products for hand hygiene such as sanitisers; Private laboratories restructured services to provide COVID-19 testing following accreditation Private universities built and strengthened platforms for long term delivery of distance learning.	Private companies diversified to manufacture PPE. City corporations in Bangladesh designated a number of hospitals in the private sector as COVID-19 hospitals	Some private health facilities restructured to provide medical services to COVID-19 patients;	

### Direct Public Health Response

#### Contribution to National Funds

In all four countries, private sector organizations and individuals provided financial resources to funds established by the government to respond to the pandemic and, in some cases, Funds established by the private sector itself. In Nepal, Bangladesh and Ghana private individuals and organizations were invited to contribute to national funds established by the Government (23–29). Participation was sometimes reluctant. In the early stages of the pandemic in Nepal, for example, skepticism was expressed by the sector about whether contributing to a government fund was effective (19). Later private companies in Nepal, notably banks, goods manufacturers and large telecoms companies donated substantial amounts (30). In addition to publicly managed funds, private business leaders in Nigeria and Ghana set up separate funds to alleviate both the public health and financial impact of COVID-19 (31–34).

#### Facilities for Quarantine, Isolation and Treatment

In all four countries, the private sector supported management of COVID-19 cases by funding and identifying spaces for quarantine, isolation and treatment. In Bangladesh, Ghana and Nepal private spaces including hotels, school buildings and churches were converted into residential quarantine and isolation centers (35–39). The private sector funded, either through national COVID-19 Funds or by direct company contribution, the construction and equipping of COVID-19 testing and treatment facilities in Nigeria, Ghana and Bangladesh (40–45).

#### Logistics for Infection Prevention, Control and Case Management

Private sector actors in all countries made financial or in-kind donations to contribute logistics needed for the public health response. These included equipment and consumables needed for infection prevention and control (such as Personal Protective Equipment (PPE), sanitiser and soap, handwashing stations, thermometer guns); treatment (including ventilators and other equipment); consumables (including oxygen, medicines like Remdesivir and Vitamin C), and testing (such as PCR machines and rapid testing kits) to facilities and agencies (46–53). Other support included the provision of data bundles and SIM cards for COVID-19 response teams and market disinfection in Ghana (54, 55). A pharmaceutical company in Bangladesh supplemented government pre-funding to the Serum Institute of India to ensure priority delivery of vaccines. In return, the company was given exclusive rights to distribute the vaccine in the country (56).

#### Risk Communication

Private organizations also played a role in mass COVID-19 education campaigns. Support included collaborations between private companies and civil society organizations to raise awareness about COVID-19 prevention (57–60), collaboration between telecoms companies to provide public health safety information (61, 62), and involvement in door to door distribution of information, including in areas such as informal settlements and slums (63).

### Protecting Livelihoods: Food Relief and Financial Assistance to Households

In all four countries, food relief was provided by the private sector to poor and vulnerable communities and business, sometimes in collaboration with public agencies. This included: the Feed a Kayayo initiative funded by the COVID-19 private sector fund providing meals to head-porters in Ghana (64); food for daily wage laborers and vulnerable families in Nepal (65, 66); and food aid provided by companies and NGOs for poor families, directly in the case of Bangladesh and Ghana and *via* the national private sector fund in the case of Nigeria and Ghana (67–74).

In two of the countries, Bangladesh and Ghana, private sector companies also provided direct financial relief to vulnerable individuals to mitigate the impact of the COVID-19 related lockdowns and other restrictions. In Bangladesh there were numerous examples, particularly *via* NGOs who have a remit to provide support to vulnerable households, and also via for-profit organizations (75–78). In Ghana, a partnership between government and a private company was formed to support enterprises with grants and soft loans, with a specific focus on assisting businesses in the informal sector owned by the vulnerable (79).

### Private Sector Service Adaptation

An important feature of the private sector response to the pandemic was the adjustments the sector made to delivery of their health and non-health services.

#### Reducing Services in Response to the Threat of COVID-19

A feature of the response documented in Nigeria, Nepal and Bangladesh in the early stages of the pandemic, was that many private health facilities reduced services, particularly to patients presenting with COVID-19 symptoms. Many hospitals in Dhaka immediately refused to treat patients with COVID-19 as they lacked appropriate facilities and were anxious that their staff and other patients may contract COVID-19 (80–82). A similar situation was apparent in Nepal where private hospitals were unwilling to bear the risk of treating COVID infected patients (83). In Nigeria, one media article reported a survey that found 26% of households could not access any medical care because of a reduction in services available through both private and public facilities (84).

#### Adjusting Services to Promote Preventative Protocols

Private businesses such as banks, factories, schools, transport drivers and market sellers introduced preventive protocols to protect customers and staff, sometimes in response to government mandates to follow COVID-19 preventive protocols. In all four countries, for example, private schools and colleges began to deliver online distance learning following government instructions to close schools (85–87). Companies remodeled their services to protect customers and staff including encouraging the use of e-banking services and safety at work facilities such as temperature checks and handwashing (88–93). These measures were often rapidly introduced and did have unintended consequences particularly for poorer households: for instance, public health measures such as market closures and decongestion led some businesses to hike prices, making essential items such as food less accessible (94). Conversely, in some cases procedures were not introduced quickly enough. Poorer workers, such as garment workers in urban areas of Bangladesh, for example, suffered most from lack of safe workplaces (95, 96).

#### Adjusting Production Processes and Services to Respond to New Market Opportunities

In all countries, companies adjusted their production processes to provide products essential to the control and management of the pandemic. In Ghana, Nigeria and Bangladesh this included diversification by companies into the production of PPE, sanitisers, medicines and Veronica buckets (97–101). Private universities in Nigeria took advantage of the ban on in person teaching to build and strengthen ICT platforms to deliver remote learning (102). In the medical sector, private companies in all four countries restructured services to provide COVID-19 tests and treatment services (103–109).

RQ2 Role of government in the private sector response to COVID-19

### Identification of Private Sector Actors in National Plans

Our data suggest that during the pandemic governments in all four countries recognized the importance of involving the private sector in the public health response in order to fill gaps left by the public sector. In Ghana, private sector representatives were involved by the Ministry of Health in developing the national strategic COVID-19 response plan as well as by the Ministry of Finance in developing the economic response (110, 111). An important role for private sector laboratories was identified in the Nigeria national strategy on COVID-19 testing (112). In Bangladesh, the COVID-19 plan identified the importance of private laboratories and hospitals in contributing to the overall health system response capacity (107, 108). The Nepal emergency response plan highlights the involvement of the private sector through a partnership model underpinned by memorandas of understanding (MOUs) (113, 114).

The study suggests a combination of collaborative public-private partnerships, contracting and regulation have been used to influence the involvement of the private sector in the response to the first wave of COVID-19 (Table 3).

**Table 3:** Government role in influencing private sector participation in COVID-19.

	**Ghana**	**Nigeria**	**Bangladesh**	**Nepal**
**RQ2 Role of government in the private sector response to COVID-19**				
2.1 Identification of private sector actors in national plans	Private sector representatives were involved by the Ministry of Health, in developing the national strategic COVID-19 response plan as well as by the Ministry of Finance in developing the economic response.	Important role for private sector laboratories was identified in the Nigeria national strategy on COVID-19 testing.	The COVID-19 plan identified the importance of private laboratories and hospitals in contributing to the overall health system response capacity.	The Nepal emergency response plan highlights the involvement of the private sector through a partnership model underpinned by memoranda of understanding (MOUs).
2.2 Public-private partnerships	COVID-19 Private Sector Fund & Ghana Health Service/Government of Ghana-partnership to increase capacity to manage COVID-19 patients whereby the private sector fund initiated construction and contributed in cash and in kind to build a 100-bed infectious disease hospital. Zipline & Ministry of Health partnership with a drone delivery service to more efficiently transport COVID-19 test samples. Health Frontiers and Ghana Airport Company partnership to provide antigen tests for arriving airline passengers.	Construction of isolation/treatment centers; provision of palliatives; through CACOVID.	Partnership between government, an NGO and a private pharmaceutical company to establish new quarantine, and treatment centers for COVID-19 patients such as a 50-bedded unit in Narayanganj; Private institute in India, Pharmaceutical company and Government of Bangladesh co-funding of the Serum institute of India to ensure priority delivery of vaccines.	Partnership between government and private sector laboratories to deliver COVID-19 testing services.
2.3 Contracting	Government of Ghana & Garment companies; contracted with selected local garment companies to produce PPE for health workers; Government of Ghana and hotels contracted with hotels to offer quarantine and isolation services for returning travelers.		Contracting of hotels to provide accommodation for staff providing COVID-19 services.	Government of Nepal & Private hospitals signed MOUs to provide treatment services at an agreed rate; Department of tourism & hotels contracting with the hospitality industry to obtain room space in hotels to supplement their own quarantine and isolation capacity; Government of Nepal & Private company agreements with a private company to procure PPE for health workers.
2.4 Regulation	Minimum standards developed by the FDA for the production of PPE; guidelines for laboratory testing developed by Ghana Health Service and enforced by the Health Facility Regulatory Agency; Safety guidelines for the re-opening of private schools; Regulation of the activities of traders to comply with government directives on preventative protocols and market decongestion, and in some cases, market closures and testing of traders.	CACOVID supported health services, including laboratories and isolation and treatment centers, required accreditation before they were permitted to deliver COVID-19 services.	Price controls introduced for masks and hand sanitisers and businesses fined for non-compliance; Government threatened to revoke licenses of private hospitals if they refused to deliver medical care	The Government of Nepal introduced a directive requiring health facilities to resume treating patients following a 3 month period during which private facilities severely curtailed access to services for anyone that was suffering or suspected to suffer from COVID-19; Price controls used to regulate fees charged in hospitals and laboratories- Health Ministry threatened action against facilities charging excess fees; confiscation of illegal masks and hand sanitizers from market and fines.

### Public-Private Partnerships

Collaborative public-private partnerships were used to expand and extend services including the private-public funding of the Serum Institute of India to deliver vaccines to Bangladesh (56), partnerships to provide quarantine and hospital capacity for COVID-19 patients in Ghana and Bangladesh (45, 115) and laboratory capacity in Nepal and in Ghana (116, 117). In Nigeria, construction and operation of COVID-19 treatment, testing and isolation centers was undertaken through partnership between the private sector fund, CACOVID and government {Not listed, 2020 #227}. Siimilarly, in Ghana, the private sector fund initiated construction and contributed in cash and in kind to build a 100-bed treatment center {Not listed, 2020 #366}. In Ghana, a partnership was developed between a private company and the Ministry of Health to use drones to more efficiently transport COVID-19 samples (118).

### Contracting

Governments agreed to purchase services from the private sector to fill gaps in COVID-19 management and prevention. In Nepal and Ghana, hotels were contracted to provide quarantine capacity (35, 36, 119). The Nepalese Government also signed MOUs with private hospitals to deliver services at fixed rate without further cost to patients (104). In Bangladesh, hotels were contracted to provide accommodation for staff delivering COVID-19 services (120).

In some cases, governance problems arose because of over-rapid contracting. In Nepal, for example, agreements with a private company were made to procure PPE following frequent news stories stressing the lack of such equipment risked health worker lives (121). However, this order was later rescinded because the contracts, made in haste, did not adhere to government procurement rules (122).

In the initial stages of the pandemic, the private health sector in Nepal had relatively little role in COVID-19 response largely due to the lack of policy clarity about the rules for engaging in service provision. This led to delays contracting the sector to deliver essential services in short supply such as PCR tests (123) leading to criticism that the government was not engaging sufficiently with the private sector in the management of the response (124). In Bangladesh, the government was accused of missing valuable opportunities to collaborate with non-government organizations to obtain a cheap (lateral flow) COVID-19 testing system (125).

### Regulation

The regulation of private sector COVID-19 related activity has taken a number of forms, including price controls, the enforcement of guidelines, safety protocols and directives.

Profiteering or price gouging was evident in all countries, particularly affecting the ability of poor households to access essential public health services such as COVID-tests and hospital services (126–128). Price controls were introduced in Bangladesh and Nepal. In Bangladesh, price controls were used for masks and hand sanitiser and businesses were fined for non-compliance (129, 130). In Nepal, controls were used to regulate fees charged to COVID-19 patients in hospitals (131). The Nepal Health Ministry threatened action against private hospitals and laboratories that were charging fees for PCR tests in excess of those mandated by the government (132). However, facilities continued to flout the decision to reduce the maximum price of a PCR test for weeks after the prices were controlled (133). Although the prices of private sector tests fell in Nepal, perhaps in response to market pressure, they remained above the government prescribed maximum (134).

The diversification of the private sector into the production of COVID-19 related goods and services has necessitated the specification of new standards. The Food and Drug Administration in Ghana, for example has developed minimum standards for the production of PPE (135) Diversification into COVID-19 products has led to allegations of both poor quality, as with tailors in Bangladesh who began to produce PPE without knowledge of the standards required (136), and corruption, when products such as hand sanitiser were deliberately faked (100, 137). In Nepal there were incidents of black marketing of commodities including masks and thermometers (138, 139) while in Ghana, unregistered medicines falsely promoted as COVID-19 cures were seized by the FDA (140).

Regulation has been required to ensure the safe provision of services in private facilities, including educational and health facilities. In Ghana safety protocols were developed to allow private schools to re-open, after at first being required to close completely (141). Traders in Ghana were asked to comply with government directives to decongest markets, including through mechanisms such as relocation exercises, rotational systems of trading, and in some cases, market closures and testing of traders (142, 143). In all four countries, guidelines on COVID-safe private medical services were developed. In Nigeria, all CACOVID-supported health services, including laboratories and isolation and treatment centers, required accreditation from the state or national government before they were permitted to deliver COVID-19 services (144, 145). In Ghana, the Ministry of Health developed and enforced national guidelines for laboratory testing as well as for treatment, with private health facilities required to comply, including entering their testing data into the national surveillance database (106). Despite these regulations, some facilities engaged in problematic practices, leading to the need for government regulatory action (106, 146). In Bangladesh, for example, a private hospital was sealed off after evidence that COVID-19 tests had been forged (147).

The Nepalese government introduced a directive requiring health facilities to resume treating patients. This followed a 3 month period during which private facilities severely curtailed access to services for anyone that was suffering or suspected to suffer from COVID-19 (148). Similarly, in Bangladesh, the Government told private hospitals not to turn away emergency patients and forced to threatened to revoke licenses of private hospitals if they refused to deliver care (149). Some hospitals ignored these instructions leading to the need for further intervention by authorities (150).

## Discussion

The review suggests that the private sector has played a substantial and varied role in the COVID-19 response in all four countries. In all countries, private sector organizations have contributed funding and supplies, have adapted their services and diversified into providing new ones. In addition, governments have attempted to collaborate with the private sector, to regulate their activities and enforce regulations where necessary.

### Motivations for Private Sector Action

The review suggests a number of motivating factors behind COVID-19 related private sector action. Some of the contributions, such as the cash and in-kind donations by private companies to the public health and relief efforts across the countries, might be classified as part of corporate social responsibility toward the pandemic. Corporate funding for national funds as well as one off health service infrastructure investments were well publicized with acknowledgments on fund websites and much publicity in the national press; for example: (26, 49, 79). As discussed elsewhere for the hospitality sector, there is evidence that corporate social responsibility during the pandemic has boosted firm value with a positive impact on business survival (151).

A second factor motivating the private sector appears to be business survival and revenue protection. Use of hotels to provide quarantine space in Nepal, for example, provided much needed support to the tourism sector. Empty conference venues in Bangladesh performed a similar function. Similarly, efforts to protect workers and keep places of work safe from COVID-19 infection have both important benefits for workers and the general population but also enabled businesses themselves to survive (152). Similar revenue protection during the pandemic is reported in other settings. A recent international newspaper review suggested that liquidity problems in the private medical sector resulting from a decline in revenues and increasing costs of service provision acted as a stimulus to either stop treating expensive COVID-19 patients or increase the prices for treatment (15).

In other cases, the financial benefits of private sector responses have been a little more speculative although potentially more profitable. Diversification by textile and beverage companies in Ghana into the production of PPE and hand sanitiser protects the current revenue base without necessarily being a longer run corporate strategy. Supplementing Government of Bangladesh pre-funding to the Serum Institute for the development of vaccines gave the company exclusive rights to distribute a resulting vaccine in the country part of which will be sold to private paying customers.

### Improving the Government Response to the Private Sector

Governments in each country have been forced to quickly develop and adapt strategies to work with the private sector. In all countries the private sector was identified as a key player in the response and were consulted with and written into accompanying strategies. The response by the sector and success of government in managing the response is likely to depend on the extent to which public and private sector incentives and motivations align. As reflected above, private enterprises had strong incentives to cooperate with government in raising funds to alleviate the harm done by COVID-19. They also had a strong self-interest in abiding by and even leading the introduction of public health measures within their own workplaces. Private funding for COVID-19 initiatives, frequently in collaboration with government, have been high profile and often substantial: Devex suggests that in Ghana a third of COVID-related spending has come from the private sector (153).

Government control and regulation over other aspects of the private sector response has proved rather more difficult. Governments in each country have faced a series of challenges in regulating the private sector. While our examples are specific to the COVID-19 response, they probably reflect the general difficulties of dealing with large, dispersed and often historically unregulated private sectors. These include: ensuring that appropriate private (non-COVID) health services were still delivered; regulating the price and quality of COVID-19 products and services; developing effective partnerships with the private sector to complement or address gaps in the work of the public sector.

#### Ensuring Services

The motivations for private health facilities to continue to deliver health services during the pandemic are perhaps complicated. As in other countries, the pandemic required complex and costly safety measures to be introduced. Private facilities often took a strongly risk adverse approach to providing services. Many facilities were unable or unwilling to risk admitting COVID-19 patients to protect patients and staff leading to reports that patients could not access health-care. Government took action in several countries to request and cajole private services to re-open including, in Bangladesh, a threat to revoke their licenses (149). Such action was only partly successful. In Bangladesh and Nepal it was reported that private hospitals had still not opened up many months after government requested that they re-open (150, 154).

#### Effective Regulation

Government was challenged in delivering consistent and effective regulation to the markets emerging from the demands created for COVID-19 products. These challenges include limiting profiteering or price gouging due to limited supply of essential products. This is a practice noted in a number of other countries as is the observation that government often appears to be unable to control the practice (15). A further challenge was the quality control of products developed from business diversification and limits on the exports of essential supplies. It is often unclear whether governments really have the ability to enforce regulation. The responsibility for regulation often falls on local government which often lack the resources to enforce nationally set standards (155). In both Nepal and Bangladesh there were reports that companies continued to flout the standards set by government. The difficulties in regulating an often powerful private sector are reflected in other countries. In India, for example, while states announced that care for COVID-19 patients should be free or highly subsidized in private and public facilities many large hospitals ignored the request and patients complained of being directed first to unsubsidised beds (156).

#### Public-Private Collaboration

The recognition of the private sector as an important stakeholder and source of financial support has not always translated into rapid utilization of capacity through partnerships and direct contracting. Delays and under-utilization of private sector resources was reported in Nepal, Ghana and Bangladesh. Where collaboration between government and the private sector appears to have been most successful is where there is a clear definition of the gaps in state provision followed by transparent agreements to jointly produce with or contract from the private sector. This was the case in Ghana in contracting with the garment sector for PPE and purchase of hotel space for quarantine by the Nepal government. The rapid use of private companies to provide quarantine space was in stark contrast to the weak efforts in several countries to ensure that private medical services remained accessible, affordable and safe.

## Preparing for the Next Pandemic: The Role of Governments and the Private Sector

The private sector as employer and provider of essential services is of growing importance to the increasingly urban population across the four countries and other similar contexts. The dependence of urban populations on private health services meant that they were particularly impacted in the initial stages of the pandemic when many private facilities closed or severely curtailed services. The experience of the four countries suggests a number of ways in which the response to the private sector might be improved in order to make the health system more resilient to future health crises including pandemics.

Fundamental to an improved response is to ensure that essential health services continue to be delivered including through the private healthcare sector upon which so much of the population is dependent, especially in urban areas which have borne the brunt of the pandemic in most countries. There is some evidence that working with the private sector can improve access to basic health services for poor households (157). Private services have invariably been included in strategic plans for coping with the pandemic. The study suggests that these intentions are often not translated into rapid delivery of services both because rules on protecting non-COVID patients were unclear and rules on procurement of private services not well understood. This is not just an issue in low resource settings. In the UK, the majority of private sector capacity block purchased by the public sector in the early stages of the pandemic was unused because of confusion over contracting processes and a mismatch between population need and available resources (158). The experience suggests the need for a clear framework to continue essential health services either by taking over private sector capacity or through clear rules that allow rapid procurement of non-government services.

A second requirement is the ability to provide the surge capacity necessary to overcome shortages in logistics in the early stages of the pandemic. Around the world, in rich as well as poorer countries health systems struggled to ensure that supplies of essential products and services necessary to deal with the pandemic were available (159). The reasons for this are multiple and include an ideological preference for private sector delivery of services (160). In all four countries a strategy is needed for ensuring supply chain resilience either by ramping up public production or developing systems to ensure rapid, quality procurement from the private sector, a theme picked up in other contexts from Iran to South Africa (161, 162).

Finally, plans—including regulations and means of enforcement-are needed to protect the population, both in terms of health care treatment and prevention and ensuring that private workplaces remain safe during a public health emergency. Within cities, local governments responsible for enforcement of regulation need the human and resource capacity to apply and enforce regulations within the complex and dynamic environments in which they operate. This is particularly challenging when regulations may disproportionately affect the poorest in the city. Planning for future pandemics must engage city governments and consider the protection of not only the health of city dwellers but also their financial means of survival.

## Limitations

The study was largely restricted to the first phase of the pandemic, until September 2020. It was restricted to documents made available electronically. It may have missed government policy documents that were not made publicly available. The pandemic evolved rapidly and unpredictably and the conclusions drawn are confined to the experience of the first 6 months only. Conclusions and recommendations will benefit from further analysis and discussion once the pandemic has past. The review was largely descriptive in scope and so does not attempt to quantify the impact of private sector behavior or the government's own response to that behavior. Qualitative interviews would have helped to elucidate some of the themes, but resource constraints and pandemic restrictions made this impossible. Although the role of different levels of government in influencing the private sector response was included in the analysis very few media reports included references to local government.

## Conclusion

The private sector has been a key player in the COVID-19 response, not only within the health sector but also across multiple sectors impacting on health, prevention and livelihoods. This is particularly evident in urban areas, where the private sector dominates. Governments have followed a variety of strategies, including partnerships, contracting and regulation to attempt to manage the private sector response. Early, collaborative planning where local governments can reflect the realities and vulnerabilities of urban populations is vital if future pandemics are to be controlled whilst mitigating negative social and economic impacts on the urban poor.

## Data Availability Statement

The original contributions presented in the study are included in the article/Supplementary Material, further inquiries can be directed to the corresponding author.

## Author Contributions

LJW, HE, TE, and MD: conceptualization. OO, RH, JN, SB, and ZQ: supervision. DB, CM, BN, SS, LJW, DJ, and AO: investigation. LJW, HE, TE, and IA: writing original draft preparation. All authors contributed to the article, writing-review and editing, and approved the submitted version.

## Funding

The study in Nepal, Nigeria, and Bangladesh was part of CHORUS funded by UK Aid, from the UK Government, Grant 301132 (involving HE, TE, MD, OO, RH, JN, SB, ZQ, DB, CM, BN, AO, SS, DJ, and IA). In Ghana (involving LJW, IA, AO, and JN) the study was funded jointly by IDRC, Grant 109479 as part of the CATALYSE project (Exploring and learning from evidence, policy and systems responses to COVID-19 in West and Central Africa) and UK Aid as part of CHORUS (Community-led Responsive and Elective Urban Health Systems). The funders had no role in study design, data collection and analysis, decision to publish, or preparation of the manuscript.

## Author Disclaimer

The views expressed do not necessarily reflect the UK or Canadian government's official policies.

## Conflict of Interest

SB, DJ, and SS were employed by HERD International.

The remaining authors declare that the research was conducted in the absence of any commercial or financial relationships that could be construed as a potential conflict of interest.

## Publisher's Note

All claims expressed in this article are solely those of the authors and do not necessarily represent those of their affiliated organizations, or those of the publisher, the editors and the reviewers. Any product that may be evaluated in this article, or claim that may be made by its manufacturer, is not guaranteed or endorsed by the publisher.
