# Supplementary material for: The Role of the Private Sector in the COVID-19 Pandemic: Experiences From Four Health Systems
Source: Front Public Health. 2022 May 27;10:878225. doi: 10.3389/fpubh.2022.878225 (PMC9195628; doi:10.3389/fpubh.2022.878225)
Supplement: Supplementary file 1 [file Data_Sheet_1.pdf]

Nigeria media articles – NATIONAL DAILIES

Date published	Title or full citation of media report	Source
27/01/2020	Coronavirus: Bill and Melinda Gates Foundation Gives $10m To China, Africa	Nigerian Tribune
31/01/2020	FG Develops Action Plan Against Coronavirus, Raises Response Committee	Nigerian Tribune
31/01/2020	FG strengthens response plan to stop importation of coronavirus	Vanguard
01/02/2020	FG reviews national strategies for Coronavirus, sets up committee to develop action plan	The Guardian
03/02/2020	Lagos Sets up Incident Command for Coronavirus	This Day
08/02/2020	Coronavirus: WHO Deploys Experts To Support Nigeria's Response Plan	Nigerian Tribune
12/02/2020	BREAKING: Nigeria Gets Three Centres To Test For Coronavirus	Nigerian Tribune
13/02/2020	Coronavirus, Lassa Fever: FG Offers N36 Million To Scientists For Solution	Nigerian Tribune
20/02/2020	Nigerians React To Use Of Chloroquine For Fighting Coronavirus	The Guardian
24/02/2020	Coronavirus: FG releases N386m for preparedness	Vanguard
28/02/2020	Nigeria confirms first case of coronavirus	The Guardian
28/02/2020	Coronavirus: FG Deploys Rapid Response Teams Across States	This Day
28/02/2020	Nigerians Panic as Lagos Scouts for Persons in Contact with Italian	This Day
28/02/2020	BREAKING: FG Confirms First Case Of Coronavirus In Lagos	Nigerian Tribune
28/02/2020	Don't Panic Over Confirmed Case Of Coronavirus — FG	Nigerian Tribune
28/02/2020	NCDC Deploys Rapid Response Teams To Lagos	Nigerian Tribune
29/02/2020	FAAN, Port Health intensify screening at Lagos Airport	Vanguard
01/03/2020	NCAA harmonises COVID-19 preventive measures in airports	Vanguard
01/03/2020	FCT Residents Decry Scarcity Of Face Masks	Nigerian Tribune
01/03/2020	NCDC Releases 4th Advisory To Nigerians On COVID-19	Nigerian Tribune
02/03/2020	The challenge and opportunities of Coronavirus	Punch
02/03/2020	We trained 342 medical personnel before index case – LASEMA	TheNation
03/03/2020	Schools In Lagos Begin Preventive Measures To Protect Pupils, Students	Nigerian Tribune
04/03/2020	Lagos sensitises religious leaders to COVID-19	Punch
04/03/2020	Ship owners get new guidelines to avoid Covid19	The Guardian
05/03/2020	FG may review 2020 budget — Finance Minister	Vanguard
05/03/2020	Buhari applauds Dangote Foundation’s N200m donation	The Guardian
06/03/2020	COVID-19 and social media	Vanguard
06/03/2020	NCDC to Spend N1.6bn in 28 Days to Tackle Covid-19	This Day
06/03/2020	Onne Port stations response team, holding bay at terminals	Vanguard
09/03/2020	FAAN strengthens airport safety	Vanguard
09/03/2020	FG confirms another case of coronavirus in Nigeria	Vanguard
09/03/2020	FG urges personal hygiene to curb spread of coronavirus	Vanguard
09/03/2020	Buhari names 12-member presidential task force to control spread	Punch
09/03/2020	Health Minister Cautions Against Misinformation	Nigerian Tribune
10/03/2020	Common myths, controversies, misconceptions about coronavirus	Punch
11/03/2020	The World Health Organization (WHO) just declared the novel coronavirus outbreak to be a pandemic.	This Day
12/03/2020	FG Increases Health Measures At Airports	Nigerian Tribune
12/03/2020	Nigeria Doesn't Stockpile Medical Consumables For Emergency Response -Expert	Nigerian Tribune
13/03/2020	NCDC advises FG on COVID-19 test centres location	Punch
13/03/2020	Why Nigeria isn’t implementing travel bans — Health Minister	Vanguard
13/03/2020	Why FG Remains Adamant To Place Travel Ban — Health Minister	Nigerian Tribune
14/03/2020	Firm partners FAAN to prevent Coronavirus spread	The Guardian
15/03/2020	Suspected COVID-19 patient isolated in Enugu	Vanguard
15/03/2020	Enugu Quarantines COVID-19 Suspect	This Day
15/03/2020	Ban church, mosque worships for 40 days, Buhari’s campaign DG tells FG	Vanguard
15/03/2020	No plan to close schools over COVID-19	TheNation
15/03/2020	Suspected COVID19 Patient: Enugu govt releases funds for emergency response	Vanguard
15/03/2020	Nigeria Records Decline In Issuance Of Visas	Nigerian Tribune

Date published	Title or full citation of media report	Source
16-Mar	Nigeria's dysfunctional healthcare delivery system and COVID-19 incubus	Vanguard
16-Mar	Enugu case still undergoing medical test, minister declares	The Guardian
16-Mar	Enugu COVID-19 suspect tests negative	Punch
16-Mar	Mass gathering can amplify spread of COVID-19 – NCDC	Vanguard
16-Mar	FG Suspends $3.3bn Eurobond Issuance	Nigerian Tribune
16-Mar	FG To Begin Secondary Screening Of Travellers From High-Risk Countries	Nigerian Tribune
16-Mar	We'll Place Travel Restriction If… — FG	Nigerian Tribune
17-Mar	Nigeria should deploy military to tackle pandemic, says Gbajabiamila	Punch
17-Mar	Abayomi calls for synergy between doctors in private, public hospitals	Vanguard
17-Mar	FG Extends Travel Caution to Germany, France, Spain	This Day
17-Mar	FG turns to varsities, ACEs to tackle COVID-19	Punch
17-Mar	Events of mass gatherings must reinforce messages on general hygiene — NCDC	Vanguard
17-Mar	FG Inaugurates Presidential Task Force on Coronavirus	This Day
17-Mar	FG suspends aviation conference over COVID-19	Vanguard
17-Mar	Halting COVID-19 In Nigeria	Nigerian Tribune
18-Mar	FG bars officials from foreign trips	Vanguard
18-Mar	Shut all borders now — Senate, House of Reps, NMA, others	Vanguard
18-Mar	Experts Urge FG to Sustain Measures against COVID-19	This Day
18-Mar	FG Suspends Visa on Arrival Policy, Bans Travellers from 13 High-risk Countries	This Day
18-Mar	Enugu govt approves N320m relocates state Isolation Centre to ESUT Teaching Hospital	Vanguard
18-Mar	LASG religious leaders bicker over plan shutdown of churches, mosques	Vanguard
18-Mar	Lagos govt places temporary ban on large religious gatherings	Vanguard
18-Mar	FG cancels visas issued for 13 countries	Vanguard
18-Mar	Lagos govt bans religious congregational services over COVID-19	Vanguard
18-Mar	Enugu Government approves relocation of isolation centre	Vanguard
18-Mar	Coronavirus: Lagos announces closure of schools	Punch
18-Mar	Reps Ask FG To Shutdown Worship Centres	Nigerian Tribune
18-Mar	Emefiele Announces N1.1trn Intervention Fund	Nigerian Tribune
19-Mar	FG shutdown 104 unity colleges, public tertiary institutions	TheNation
19-Mar	Lagos to Close all Sports Facilities	This Day
19-Mar	FG Strategy Of Self-Quarantine Is Counter-Productive — NLC	Nigerian Tribune
19-Mar	Enugu Govt Shuts Down Primary And Secondary Schools	Nigerian Tribune
19-Mar	FG, State Governors Agree On Establishment Of 36 Functional Test, Isolation Centres	Nigerian Tribune
19-Mar	Nigeria's Travel Ban and the Delayed Measure against COVID-19	This Day
20-Mar	Osinbajo Chairs FG/States Special Committee on COVID-19	This Day
20-Mar	FG Restricts International Flights to Lagos, Abuja Airports	This Day
20-Mar	NUC orders closure of all universities nationwide	The Guardian
20-Mar	WAEC postpones examination over COVID-19	Vanguard
20-Mar	COVID-19 Awareness Not Reaching Grassroots — CSOs	Nigerian Tribune
21-Mar	FG shuts Enugu, Port Harcourt and Kano Airports	Vanguard
21-Mar	FG Stops Railway Operations in Nigeria	This Day
21-Mar	Lagos Reduces Social Gathering from 50 to 20	This Day
21-Mar	Nigeria closes all airports to international flights	The Guardian
21-Mar	Ortom expresses concern over safety of IDPs, Cameroonian refugees	Vanguard
22-Mar	COVID-19 fund: SERAP asks Buhari to instruct EFCC, ICPC to track, monitor spending	Vanguard
22-Mar	Lagos Issues Public Transport Guidelines	This Day
22-Mar	Nigeria Moves to Lockdown as 11 New Cases are Confirmed in Lagos, Abuja	This Day
23-Mar	Enugu police enforces restriction order, stops unnecessary arrests, detention	Vanguard
23-Mar	NSCDC deploys 9500 to curtail spread of COVID- 19	Vanguard
23-Mar	FG to recall retired doctors, nurses over COVID-19	TheNation
23-Mar	FG shuts land borders over COVID-19	TheNation
23-Mar	FG cautions religious, political leaders over flouting its directives	Vanguard

Date published	Title or full citation of media report	Source
30-Mar	CBN-Led Coalition Begins Supply Of Medical Facilities In Six Geo-Political Zones	Nigerian Tribune
04-Apr	BoI donates N700 million towards COVID-19 relief aid	The Guardian
17-Apr	Donations To COVID-19 Relief Fund Hit N25.8bn From 107 Donors, Says CBN	Nigerian Tribune
19-Apr	CACOVID to Distribute Food Worth N23bn to 10m Nigerians	This Day
20-Apr	CACOVID set to distribute food items to 1.7m vulnerable people in 774 local govts	Vanguard
04-Jun-20	Health workers disagree over govs' takeover of COVID-19 battle	Punch
04/06/2020	Misinformation, Anxiety Fueled Covid-19, Says Google. 4 June 2020	This Day
04/06/2020	Ehanire commends Edo government on COVID-19 response	The Guardian
04/06/2020	COVID-19: FG Will Approve Palliative for Security Agencies, Says PTF	This Day
04/06/2020	Hunger Games: The Socioeconomic Divide and Response to Disease Outbreaks	This Day
04/06/2020	COVID-19: NIGERIA EXPERIENCING STEADY RISE IN MATERNAL, CHILD MORTALITY — PTF	Nigerian Tribune
04/06/2020	COVID-19: AFRICAN NATIONS GET $9.8BN AS IMF SUPPORTS 66 COUNTRIES WITH $23BN	Nigerian Tribune
04/06/2020	NIGERIANS HAWKING FAKE COVID-19 CONTRACT PAPERS, PTF RAISES ALARM	Nigerian Tribune
04/06/2020	COVID-19: PTF DECRIES REFUSAL OF CITIZENS ON CONTACT TRACING, ISOLATION	Nigerian Tribune
04/06/2020	COVID-19: DON'T TRANSFER RESPONSIBILITY TO GOVERNORS, NMA PRESIDENT TELLS FG	Nigerian Tribune
04-Jun	NNPC launches App for COVID-19 contacts tracing, begins sale of products online	The Guardian
04-Jun	Government not making good use of academic resources, says Adeyemi	The Guardian
04-Jun	How COVID-19 has derailed efforts to end AIDS, by Idoko	The Guardian
04-Jun	FG Laments Ravaging Effect of COVID-19 on Revenues	This Day
04-Jun	COVID-19: Hacey holds maternal health support programme in Lagos, Ogun, Oyo	The Guardian
04-Jun	PTF calls for decentralization of COVID-19 response to LGA level, identifying high burden LGAs	The Guardian
04-Jun	Why flights after COVID-19 will be 'expensive' – PTF	Punch
05/06/2020	ACCOUNTING FOR COVID-19 FUNDS	This Day
05/06/2020	COVID-19: Nigeria's Response and Big Challenges	This Day
05/06/2020	COVID-19: MDAs Earmark N20m to Improve Hygiene, Upgrade Facilities	This Day
05/06/2020	Horrendous revelations of COVID-19 – Part 2	The Guardian
05/06/2020	FG removes antiviral drugs from COVID-19 treatment guidelines	Punch
05/06/2020	Inadequate PPE supply; a health risk to the frontline workers in Africa amidst the COVID-19 pandemic	The Guardian
05/06/2020	Top nine coronavirus myths in Africa	TheNation
06/06/2020	Umahi gives palliatives to Ebonyi indigenes in Lagos	The Guardian
06/06/2020	COVID-19: Lagos begins home care for asymptomatic patients, cases hit 11,844	Punch
06/06/2020	We panic, expect the worst treating COVID-19 patients –Frontline health workers	Punch
06/06/2020	Hotel owners seek palliatives from Lagos State, fault 'register to reopen' policy	The Guardian
06/06/2020	COVID-19: Helpgate Foundation organises sensitisation programme	The Guardian
06/06/2020	There is no certified treatment, rapid test kit for COVID-19, says Ehanire	The Guardian
06/06/2020	Lagos commences home-based care for asymptomatic COVID-19 cases management	The Guardian
06/06/2020	COVID-19: AfDB approves Nigeria's $288.5m loan	Punch
06/06/2020	Lagos accredits three private hospitals for COVID-19 treatment	Punch
06/06/2020	COVID-19: NNPC COMMENCES CONSTRUCTION OF N21BN INFECTIOUS DISEASE HOSPITAL IN KATSINA	Nigerian Tribune
15-Jun	CACOVID TAKES AWARENESS CAMPAIGN TO GRASSROOTS	Nigerian Tribune
07-Jul	Coronavirus: International transport and supply chains key to COVID-19 recovery – UN agencies	The Guardian
17-Jul	Why we didn't distributes looted COVID-19 palliatives – Committee	Vanguard
23-Jul	COVID-19 palliatives in warehouses were awaiting distribution — CACOVID	Vanguard
07-Aug	CISLAC Asks N'Assembly to Probe Delay in Palliatives Distribution	This Day
08-Aug	FG inaugurates committees for COVID-19 recovery	Vanguard
09-Aug	NCDC launches COVID-19NigeriaStories blog	Vanguard
11-Aug	N28.7bn Spent On Food Items As Palliatives For Nigerians — CACOVID	Nigerian Tribune

Date published	Title or full citation of media report	Source
11-Aug	COVID-19: THE PTF COLLABORATION MODEL	This Day
13-Aug	States get N66.5b as COVID-19 response grant	The Guardian
31-Aug	COVID-19 vaccine: Nigerians risk global travel ban, Senate warns, NMA allays fears	Punch
01-Sep	FG Launches COVID-19 Incidence Tracker Dashboards	This Day
06-Sep	COVID-19: Chloroquine used only in clinical trials in Nigeria, says NCDC boss	Vanguard
17-Sep	How we spent N31bn in 4 months to fight COVID-19', FG replies SERAP, CODE	Vanguard
19-Sep	Accounting for COVID-19 funds	Punch
23-Oct	Making personal protective equipment against COVID-19 available in hospitals	The Guardian
26-Oct	Establishment of National Emergency Medical Service & Ambulance System (NEMSAS) for COVID-19 response	The Guardian
01-Nov	What Scientists Must Do to Access CBN's Research Grant	This Day
14-Nov	Assessment of Public Health Emergency Operations Centres (PHEOC) in response to COVID-19 pandemic	The Guardian
19-Nov	FG commends wapTV on COVID-19 enlightenment activities	The Guardian
21-Nov	World Bank Approves $114.28m for Nigeria to Boost States' COVID-19 Response	This Day
25-Nov	How non-communicable diseases fuel COVID-19 deaths in Nigeria	The Guardian
27-Nov	Group to close Eti-Osa isolation centre in two weeks	The Guardian
04-Dec	CACOVID donates N1.4bn medical equipment, 26,400 test kits to boost Covid-19 testing across Nigeria	The Guardian
06-Dec	NIMR to deploy non-invasive ventilators for COVID-19 patients	The Guardian
07-Dec	COVID-19 must not overshadow HIV — YEDI	Vanguard
7th November, 2020.	Plateau COVID-19 medical research team submits products to regultory agencies. David. ().	The Sun Newspaper
29th October, 2020.	Medical Guild to chart roadmap on healthcare, mental delivery. Kuni Tyessi. ().	Thisday Newspaper
28th May, 2020.	PTF decries continued refusal by hospitals to treat patients for fear COVID-19. Nkechi Onyedika-Ugoeze. ().	The Guardian Newspaper
10th November, 2020.	Obaseki tasks scientists on innovations, homegrown vaccine for COVID-19. ().	The Nigerian Observer
17th November, 2020.	Strengthening health systems, citizen's participation against COVID-19.).	Daily trust
30th October, 2020.	Leveraging COVID-19 tools to fund Nigeria's epidemic preparedness. Anthonia Obokoh. ().	Business Day
4th September, 2020.	States to retain Ta reliefs till June 2021. Chuks Okocha.)	Thisday Newspaper
10th September, 2020.	Nigeria media and the fight against COVID-19 misinformattion. ().	Thisday Newspaper
12 September, 2020.	Group presents gender analysis survey on COVID-19 impact on households. Thomas-Odia. ().	The Guardian Newspaper
10 June, 2020.	Sexual and gender-based violence: Hidden social pandemic under rader of COVID-19 lockdown-Part 2. Ejiro Umukoro. ().	The Guardian Newspaper
21st September, 2020.	COVID-19 in Nigeria: States under-testing for virus, FG says. Juliana Taiwo-Obalonye. ().	The Sun Newspaper
7th August, 2020.	W'Bank approves $114.28m for Nigeria to fight COVID-19. Ajiri Daniels. ().	The Sun
7 October, 2020.	Advancing financial inclusion for women. Obinna Chima. (r).	Thisday Newspaper
16th October, 2020.	IMF commits $100b loans to Nigeria, 79 other economies. Alao Abiodun. ().	The Nation
5 May, 2020.	COVID-19: Nigeria needs updated legislation on infectious diseases- FG. Ahmed Musa. ().	Daily Independent
24 July, 2020.	COVID-19: FG asks states to ban Sallah activities. Ekene Coleman. ()	Daily Independent

Date published	Title or full citation of media report	Source
24 August, 2020.	COVID-19: Our case fatality on decline, FG says as figure reaches 1000. ().	Vanguard
31 August, 2020.	Fighting for Nigeria: Heroes of battle against COVID-19. Obinna Chima, Kunle Aderinokun and Martins Ifijeh. ().	Thisday Newspaper
12 May, 2020.	Nigeria's COVID-19 communications needs to be more inclusive. Omorinsola Balogun. ().	Business day
7 July 2020.	Slowing spread of COVID-19 with contact tracing apps. Chukwuma Muanya.).	The Guardian Newspaper
14 June, 2020.	Little to cheer in health sector, one year after Buhari's second term. Chukwuma Muanya. ().	The Guardian Newspaper
5 May, 2020.	Coronavirus: Buhari seeks int'l cooperation. Egena Sunday Ode. ().	Peoples Daily
3rd September, 2020.	Africa countries sign up for COVID-19 vaccine initiative. ().	The premium times
8th November, 2020.	NACA, UN, others identify preventive measures against COVID-19 spread. ().	The Punch Newspaper
30th October, 2020	Abuja shuts three isolation centres as COVID-19 infection drops. Eniola Akinkuotu.).	The Punch Newspaper
22nd September, 2020.	FG to states: Fight COVID-19 with N32bn we gave you. ().	Daily Trust
26th June, 2020.	The 'new normal': Living with and defeating COVID-19.	Daily Trust
28th October, 2020.	Implement 15% budgetary allocation to health, NMA tasks FG. Gabriel Olawale. ().	Vanguard
5th August, 2020.	CBN's COVID-19 intervention. Rasheed Hassan. ().	Thisday Newspaper

Nigeria media articles – BLOGS AND ONLINE NEWS

Source/URL	Main purpose
http://saction.org/civil-societys-role-in-nigerias-covid-19-response/	Civil Society's Role In Nigeria's COVID-19 Response
http://www.factcheckng.com/2020/05/how-csos-ngos-aged-fight-covid-19-in.html	How CSOs, NGOs, aged fight COVID-19 in Nigeria
http://www.healthnews.ng/nigerias-covid-19-response-threatened-by-social-stigma/	Nigeria's COVID-19 response threatened by social stigma
https://africa.cgtn.com/2020/06/28/nigerias-economic-hub-expands-covid-19-response-capacity/	Nigeria's economic hub expands COVID-19 response capacity
https://africa.unwomen.org/en/news-and-events/stories/2020/04/covid-19-and-the-link-to-violence-against-women-and-girls	COVID-19 and the link to Violence Against Women and Girls
https://medium.com/@SustainableDFS/for-nigerias-poor-covid-19-is-just-one-out-of-a-myriad-of-life-hazards-baeca528a2db	For Nigeria's poor, Covid-19 is just one of a myriad of life hazards
https://nairametrics.com/2020/06/08/covid-19-survey-confirms-job-losses-hardship-for-nigerians/	COVID-19: Survey confirms job losses, hardship for Nigerians
https://ncdc.gov.ng/news/258/nigeria-launches-covid-19-online-course-on-infection-prevention-and-control-%28ipc%29	Nigeria Launches COVID-19 Online Course on Infection Prevention and Control (IPC)
https://phys.org/news/2020-05-nigerian-tackle-covid-urban-poor.html	Nigerian government's plan to tackle COVID-19 leaves urban poor further disadvantaged
https://publicservices.international/resources/news/internally-displaced-persons-in-nigeria-face-double-risk-boko-haram-and-covid?id=10935&lang=en	Internally displaced persons in Nigeria face double risk: Boko Haram and Covid
https://www.aa.com.tr/en/africa/nigeria-ramps-up-response-efforts-to-smash-coronavirus/1771922	Nigeria ramps up response efforts to smash coronavirus
https://www.bloomberg.com/news/articles/2020-07-05/nigerian-security-response-to-covid-is-deadlier-than-the-disease	Nigerian Security Response to Covid Is Deadlier Than the Disease
https://www.bond.org.uk/news/2020/04/development-in-the-face-of-covid-19-views-from-nigeria	Development in the face of Covid-19: Views from Nigeria
https://www.devex.com/news/opinion-covid-19-is-a-game-changer-for-health-financing-in-nigeria-97117	Opinion: COVID-19 is a game-changer for health financing in Nigeria
https://www.e-ir.info/2020/06/22/nigerias-soft-power-in-the-face-of-covid-19/	Nigeria's Soft Power in the Face of COVID-19
https://www.hrw.org/news/2020/03/25/nigeria-covid-19-cases-rise	Nigeria: COVID-19 Cases On the Rise, Ensure Emergency Preparedness for Displaced, Vulnerable People
https://www.hrw.org/news/2020/04/14/nigeria-protect-most-vulnerable-covid-19-response	Nigeria: Protect Most Vulnerable in COVID-19 Response. Extended Lockdown Threatens Livelihoods of Millions
https://www.orfonline.org/expert-speak/nigeria-must-not-forget-its-poor-in-the-covid-19-world-64389/	Nigeria must not forget its poor in the Covid-19 world
https://www.proshareng.com/news/Fiscal%20Policy/COVID-19--Federal-Government-of-Nigeria-Announces-Fiscal-Stimulus-Measures/50321	COVID-19: Federal Government of Nigeria Announces Fiscal Stimulus Measures
https://www.ripplesnigeria.com/special-report-without-palliatives-small-businesses-families-suffer-amid-covid-19-lockdown-in-enugu/	SPECIAL REPORT… Without palliatives, small businesses, families suffer amid COVID-19 lockdown in Enugu
https://www.savethechildren.net/news/save-children-covid-19-set-impact-hundreds-thousands-children-dangerous-mix-poverty-hunger-and	COVID-19 set to impact hundreds of thousands of children in dangerous mix of poverty, hunger and protection risks
https://www.tekedia.com/examining-nigerias-response-to-covid-19-pandemic/	Examining Nigeria's Response to Covid-19 Pandemic

Source/URL	Main purpose
https://www.theafricareport.com/33300/pandemic-to-poverty-nigeria-in-the-post-covid-future/	Nigeria: Pandemic to poverty in the post-COVID future
https://www.thinkglobalhealth.org/article/coronavirus-nigeria-case-community-engagement	Coronavirus in Nigeria: A Case for Community Engagement
https://www.voanews.com/covid-19-pandemic/nigeria-resumes-domestic-flights-amid-pandemic	Nigeria Resumes Domestic Flights Amid Pandemic
https://www.von.gov.ng/covid-19-urban-poor-to-benefit-from-palliatives-humanitarian-affairs-minister/	COVID-19: Urban poor to benefit from palliatives – Humanitarian Affairs Minister
https://www.who.int/health-cluster/news-and-events/news/Nigeria-multi-sector-covid-19/en/	Multi-sector collaboration in Nigeria's COVID-19 Response
https://www.wsscc.org/media/resources/fighting-covid-19-pandemic-should-not-be-one-emergency-action	Fighting the COVID-19 pandemic should not be a one-off emergency action
https://www.afro.who.int/news/who-supports-anambra-state-boost-covid-19-testing-capacity-trains-cohort-health-workers	To boost COVID-19 testing capacity, trains cohort of health workers
http://www.nigerianwatch.com/governor-obiano-shuts-down-eke-awka-market-as-anambrans-ignore-covid-19-safeguards/	To ensure there is adherence to Covid local preventive guidelines. Thus, curtailing the spread of corona virus in the market. This was as a result of non-compliance by the market traders and customers with Covid-19 protocols including wearing of protective face masks, provision of running water and soap for washing of hands, keeping of physical distancing.
https://theconversation.com/lagos-makes-it-hard-for-people-living-in-slums-to-cope-with-shocks-like-covid-19-138234	Lagos makes it hard for people living in slums to cope with shocks like COVID-19
https://www.one.org/africa/blog/how-nigeria-minimize-impact-covid-19/	COVID-19's potential impact in Nigeria
https://theconversation.com/why-nigerias-efforts-to-support-poor-people-fail-and-what-can-be-done-about-it-137122	Why Nigeria's efforts to support poor people fail, and what can be done about it
https://nigeriahealthwatch.com/a-case-for-phcs-in-the-fight-against-covid19/#.Xw31-hQo_IU	A case for PHCs in the fight against COVID19
https://www.cfr.org/blog/how-nigeria-has-responded-covid-19-so-far	How Nigeria Has Responded to COVID-19 So Far
https://www.cgdev.org/blog/nigeria-ready-leave-lockdown	Is Nigeria Ready to Leave Lockdown?
http://rosalux.sn/en/5146/	Challenges of Fighting COVID-19 Pandemic the Nigerian Case
https://theconversation.com/coronavirus-corruption-in-health-care-could-get-in-the-way-of-nigerias-response-136913	Coronavirus: corruption in health care could get in the way of Nigeria's response
https://www.thinkglobalhealth.org/article/coronavirus-nigeria-case-community-engagement	Coronavirus in Nigeria: A Case for Community Engagement
https://nigeriahealthwatch.com/fragile-progress-at-the-brink%e2%80%8a-%e2%80%8acovid-19-and-the-nigerian-health-sector/#.XxmBQRQo_IU	Fragile progress at the brink - COVID-19 and the Nigerian health sector
https://nigeriahealthwatch.com/rayuwa-da-korona-how-two-local-radio-stations-are-responding-to-covid-19-in-kano-state/#.XxmBTRQo_IU	Rayuwa Da Korona: How two local radio stations are responding to COVID-19 in Kano State
https://banwo-ighodalo.com/grey-matter/policy-regulatory-measures-against-coronavirus-pandemic-nigeria?utm_source=Mondaq&utm_medium=syndication&utm_campaign=LinkedIn-integration	Policy & Regulatory Measures Against the Coronavirus Pandemic in Nigeria
https://reliefweb.int/report/nigeria/covid-19-forum-advocates-palliatives-almajiri-children	COVID-19: Forum advocates palliatives for Almajiri children
https://www.cfr.org/blog/bayelsa-nigerian-government-response-covid-19-falls-short-promises	In Bayelsa, Nigerian Government Response to COVID-19 Falls Short of Promises

Source/URL	Main purpose
https://www.cfr.org/blog/nigeria-responds-first-coronavirus-case-learning-2014-ebola-response	Nigeria Responds to First Coronavirus Case, Learning From 2014 Ebola Response
https://www.cfr.org/blog/bayelsa-nigerian-government-response-covid-19-falls-short-promises	In Bayelsa, Nigerian Government Response to COVID-19 Falls Short of Promises
https://www.chathamhouse.org/expert/comment/coronavirus-nigeria-s-fiscal-flu	Coronavirus: Nigeria's 'Fiscal Flu'
https://www.creativeassociatesinternational.com/stories/nigeria-program-pivots-to-support-coronavirus-prevention/	Nigeria program pivots to support coronavirus prevention
https://www.internationalhealthpolicies.org/featured-article/opportunities-amidst-adversity-in-nigerias-covid-19-response/	Opportunities Amidst Adversity in Nigeria's COVID-19 Response
https://theconversation.com/covid-19-nigeria-should-prioritise-power-supply-to-health-care-facilities-134444	COVID-19: Nigeria should prioritise power supply to health care facilities
-	
http://saction.org/civil-societys-role-in-nigerias-covid-19-response/	Civil Society's Role In Nigeria's COVID-19 Response
https://nigeriahealthwatch.com/psychosocial-support-a-priority-for-covid-19-survivors-in-nigeria-oped/#.XyCDKhQo_IU	Psychosocial Support a Priority for COVID-19 Survivors in Nigeria
https://gga.org/nigeria-a-pandemic-and-a-weak-health-system/	Nigeria: A pandemic and a weak health system
https://www.globalcitizen.org/en/content/challenges-for-health-care-workers-nigeria-covid/	5 Challenges Facing Health Care Workers in Nigeria as They Tackle COVID-19
https://www.theafricareport.com/33300/pandemic-to-poverty-nigeria-in-the-post-covid-future/	Nigeria: Pandemic to poverty in the post-COVID future
https://theconversation.com/nigerias-post-covid-19-recovery-plan-has-some-merit-but-it-misses-the-mark-140974	Nigeria's post-COVID-19 recovery plan has some merit. But it misses the mark
https://www.theigc.org/blog/the-shadow-pandemic-gender-based-violence-and-covid-19/	The pandemic: Gender-based violence and COVID-19
Campbell, J., & McCaslin, J. (2020). How Nigeria has responded to COVID-19 so far. Retrieved from https://www.cfr.org/blog/how-nigeria-has-responded-covid-19-so-far	Examining committedness to combating coronavirus in Nigeria
John, E. (2020). Covid-19 implications for public transport and shared taxi in Nigeria. Retrieved from https://www.transformative-mobility.org/news/covid-19-implications-for-public-transport-and-shared-taxi-in-nigeria	Transport system in urban centres and how they are affected by Covid-19
Muggah, R., & Florida, R. (2020). Megacity slums are incubators of disease - but coronavirus response isn't helping the billion people who live in them. Retrieved from https://theconversation.com/megacity-slums-are-incubators-of-disease-but-coronavirus-response-isnt-helping-the-billion-people-who-live-in-them-138092	Studying the fragility and resilience of cities and urban peripheries in Nigeria amidst COVID-19
Ayeni, T. (2020). Coronavirus: Nigeria's varied responses to controlling COVID-19. Retrieved from https://www.theafricareport.com/27773/coronavirus-nigerias-varied-responses-to-controlling-covid-19/	Understanding interventions by State and Federal Governments in the fight against COVID-19
Obiwulu, O., & Abia, B. (2020). Internally Displaced Persons (IDPs) as vulnerable populations at risk. Retrieved from http://cseaafrica.org/covid-19-in-nigeria-internally-displaced-persons-idps-as-vulnerable-populations-at-risk/	Examining reslience of city governments against coronavirus and its effects, especially on IDPs

Source/URL	Main purpose
Dixit, S., Ogundeji, Y., & Onwujekwe, O. (2020). How well has Nigeria responded to COVID-19? Retrieved from brookings.edu/blog/future-development/2020/07/02/how-well-has-nigeria-responded-to-covid-19/	Examining impacts of policies in ameliorating dificulties created by COVID-19
The first 90 days- How has Nigeria responded to the COVID-19 outbreak? #covid19naijaresponse. Chibuike Alagboso and Bashar Abubakar. 29th June, 2020. Nigeria Health Watch).	
Coronavirus - Nigeria: Shoring up COVID-19 prevention in Nigeria's border states; in Ogun state in the country's south-west where the country's first COVID-19 case was deteced, they are now helping to curb the importation of COVID-19. (28th October, 2020. African Press Organization).	
Coronavirus -Nigeria: National health sectore COVID-19 pandemic response Action plan undergoes validation, to be ratified by National Council on Health; it is expected to meet the need of all actors in the health sector and also act as a single point of entry for all to the COVID-19 pandemic response. (21 June 2020. African Press Organization).	Validation of National Health Sector COVID-19 pandemic response action plan.
COVID-19: How lockdowns affected health access in African and Asian slums. Funke Fayehun, Browyn Harris, Frances Griffiths, Narjis Rizvi, Pauline Bakibinga and Syed A.K. Shifat Ahmed. (22nd October, 2020. The Conversation).	Reports how lockdowns introduced to curb the spread of the coronavirus decreased access to all healthcare services in seven slums in Kenya, Nigeria, Bangladesh and Pakistan.
Medical malpractice in public health emergencies: A review of medical response to COVID-19 in Nigeria. Perchstone & Graeys. (Mondaq Business Briefing, 5th June 2020)	Observes medical malpractice and negligence to the duty of care among some medical practitioners/institutions in a psndemic situation.

Nepal Media articles

Date of publication (yyyy/mm/dd)	Name of news media (select from drop-down menu)	Title of news/headline
2020-08-19	Online Khabar (Nepali)	‘निषेधाज्ञा नमान्नेलाई प्रशासनले कारवाही गर्छ, कोभिडले पाता फर्काउँछ’
2020-06-22	Online Khabar (Nepali)	574.8 million spent on the fight against Corona in the Farwestern
2020-03-03	Online Khabar (Nepali)	Army Operation COVID-19 Nepal: From Wuhan to Kharipati
2020-06-23	Online Khabar (Nepali)	Home Ministry Circular: Who come to the valley should be kept in home quarantine for 14 days
2020-03-11	Online Khabar (Nepali)	If necessary, the government can rescue from any country: Shrestha
2020-02-04	Online Khabar (Nepali)	Let's keep Nepalis returned from China in Parliament House: Yadav
2020-01-27	Online Khabar (Nepali)	Outbreak of corona virus is not frightening in Nepal, but we are still alert: Tourism Minister
2020-03-17	Online Khabar (Nepali)	Prakashman's instruction to the ward chairmen: Join in the service of the people who are scared of Corona
2020-06-26	Online Khabar (Nepali)	Today, 1,200 Nepalis are coming from different countries
2020-04-06	Kantipur (Nepali)	१४ दिन पुग्यो, परीक्षण भएन
2020-04-18	Kantipur (Nepali)	१५ लाख नेपालीको रोजगारी जोखिमम
2020-04-07	Kantipur (Nepali)	१७० सेट पीपीई सहयोग
2020-04-21	Kantipur (Nepali)	१८१ जना दशगजाम
2020-03-25	Kantipur (Nepali)	१२५ यात्रीको पहिचानपछि निगरानी
2020-04-17	Kantipur (Nepali)	रोजगारदाता कम्पनी र श्रमिकलाई १९ करोड राहत
2020-04-07	Kantipur (Nepali)	रोकिएनन् महाकाली तरर` भित्रिनेहरू
2020-04-05	Kantipur (Nepali)	रोगको भन्दा भोकको पीर
2020-04-09	Kantipur (Nepali)	स् वास्थ्य सामग्री किन्न सेना रविभाग दुवैको प्रक्रिया सुरु
2020-03-24	Kantipur (Nepali)	स्वास्थ्य सामग्री आइपुग्ने छैन टुंगो
2020-03-27	Kantipur (Nepali)	स्वास्थ्य सामग्री पर्सि आइपुग्न
2020-03-27	Kantipur (Nepali)	स्थानीयस्तरमै पीपीइ
2020-03-27	Kantipur (Nepali)	सुरक्षाकर्मीलाई स्वास्थ्य सुरक्षा छैन
2020-04-04	Kantipur (Nepali)	सरकारले नसक्ने, सेनालाई सुम्पने !
2020-04-05	Kantipur (Nepali)	सेना र स्वास्थ्य सेवा विभाग दुवै खरिद प्रक्रियामा
2020-04-06	Kantipur (Nepali)	सेनाले अघि बढायो स्वास्थ्य सामग्री खरिद प्रक्रिया
2020-04-21	Kantipur (Nepali)	सेनाले पूर ा गऱ्यो खरि द प्रक्रिया
2020-04-12	Kantipur (Nepali)	सेनालाई चीन, भार त रकोरियाको जवाफ
2020-03-24	Kantipur (Nepali)	संक्रमण रोक्न १० करोडको कोष
2020-03-26	Kantipur (Nepali)	संक्रमणको जानकारी १४ घण्टा ˘लुकाइयो ’
2020-04-13	Kantipur (Nepali)	संक्रमित भेटिएको क्षेत्र ‘सिल’
2020-04-15	Kantipur (Nepali)	संक्रमित भर्ना भएपछि अस्पताल सुनसान

Date of publication (yyyy/mm/dd)	Name of news media (select from drop-down menu)	Title of news/headline
2020-04-05	Kantipur (Nepali)	संक्रमितका १०० सहयात्री सम्पर्क बाहिर
2020-04-07	Kantipur (Nepali)	सुदूरपश्चिममा छैनन् चिकित्सक
2020-04-15	Kantipur (Nepali)	सांसदको बजेट कारोना कोषमा राख्न सहमति
2020-04-17	Kantipur (Nepali)	सामग्री ल्याउन दुवै वाइडबडी चीनतप
2020-04-08	Kantipur (Nepali)	सुनसान अस्पताल
2020-03-24	Kantipur (Nepali)	सीमा बन्दले पनि रोकिएन आवागमन
2020-04-07	Kantipur (Nepali)	सीमा क्षेत्रमा थप सुरक्षा फौज
2020-04-08	Kantipur (Nepali)	सीमामा ४१५ चेकप्वाइन्ट थप
2020-03-25	Kantipur (Nepali)	सीमामा दिनभर अलपत्र
2020-04-04	Kantipur (Nepali)	२५ हजार बढी किट भन्सार मै
2020-04-20	Kantipur (Nepali)	˘ असुरक्षाले’ अर्थ समितिको भर्चुअल बैठक स्थगित
2020-04-10	Kantipur (Nepali)	˘सांसदलाई बजेट नछुट्याऔं, स वास्थ्य पूर्वाधार बनाऔ
2020-04-10	Kantipur (Nepali)	˘सांसदलाई बजेट नछुट्याऔं, स्वास्थ्य पूर्वाधार बनाऔ
2020-04-12	Kantipur (Nepali)	˘साइकलबाट झर्दा रगतले सारी भिजेको हुन्थ्या
2020-08-21	Online Khabar (Nepali)	‘७७ जिल्लामै पीसीआर परीक्षणको व्यवस्था मिलाऊ’
2020-03-27	Himalayan Times (English)	‘Announce relief package for daily wage earners’
2020-03-26	Kantipur (Nepali)	˘देशभर एक लाख ३० हजार अट्ने क्वारन्टाइन` ’
2020-06-27	Himalayan Times (English)	‘Covid victims deserve dignified last rites’
2020-07-19	Himalayan Times (English)	‘Enough is Enough’ campaigners begin hunger strike once again
2020-03-19	Himalayan Times (English)	‘Govt serious about COVID-19 pandemic’
2020-03-26	Himalayan Times (English)	‘Govt working to ensure supply of daily essentials’
2020-03-17	Himalayan Times (English)	‘Health desk at Rani border ineffective
2020-03-15	Himalayan Times (English)	‘Hospitality sector to be hit entire year’
2020-04-17	Kantipur (Nepali)	˘प्रवासी फर्कने व्यवस्था मिलाऊ
2020-06-28	Himalayan Times (English)	‘Nepal needs to break free from the shackles of the past’, Page 2
2020-02-12	Himalayan Times (English)	‘Preparation to quarantine evacuees complete’
2020-05-03	Himalayan Times (English)	‘Quarantined people can suffer mental health problems’
2020-07-09	Himalayan Times (English)	‘Respect rights of vulnerable people’
2020-04-06	Kantipur (Nepali)	˘चीनले नेपाललाई सामग्री उपलब्ध गराउँछ
2020-06-12	Himalayan Times (English)	‘Send critical patients to specialised hospitals’
2020-05-05	Himalayan Times (English)	‘Test, trace, test to arrest COVID transmission’
2020-06-01	Himalayan Times (English)	‘Trace, test, improve quarantines, increase capacity of health centres’

Date of publication (yyyy/mm/dd)	Name of news media (select from drop-down menu)	Title of news/headline
2020-06-01	Himalayan Times (English)	‘Trace, test, improve quarantines, increase capacity of health centres’
2020-04-06	Kantipur (Nepali)	˘गम्भीर बिरामीले उपचार पाएनन्
2020-04-15	Kantipur (Nepali)	˘गरिबको कोही नहुँदो रहेछ’
2020-03-27	Kantipur (Nepali)	˘शंकास्पद बिरामी जाँच्दा पीपीई चाहिँदैन’
2020-06-08	Kantipur (Nepali)	‘अब पुँजीबजार खोल्नुपर्छ’
2020-04-16	Online Khabar (Nepali)	‘अब लकडाउनको मोडल फरक हुन्छ, आशंकित समुदाय सिल हुनसक्छ’
2020-05-12	Kantipur (Nepali)	‘अर्थतन्त्र आफैं पुनर्निर्देशित हुँदैछ’
2020-07-03	Kantipur (Nepali)	‘अर्थतन्त्रको मनोबल बढाउने नीति’
2020-05-22	Kantipur (Nepali)	‘आगामी बजेटमा पौने दुई खर्बको राहत’
2020-05-19	Kantipur (Nepali)	‘आरडीटी किटले संक्रमण विस्तारमा सघाउँछ’
2020-08-24	Online Khabar (Nepali)	‘उपत्यकामा १५ हजार संक्रमित हुनसक्छन्’
2020-08-10	Online Khabar (Nepali)	‘उपत्यकामा सार्वजनिक यातायात र अत्यावश्यकबाहेकका सेवा रोकौं’
2020-06-20	Kantipur (Nepali)	‘कमजोर अर्थतन्त्रलाई भी सेपमा रिकभरी गर्छौं’
2020-06-27	Kantipur (Nepali)	‘काम गरिखान पाऊँ’
2020-08-16	Kantipur (Nepali)	‘कामदारले उपचार पाएनन्’
2020-05-23	Online Khabar (Nepali)	‘कि डाक्टर र नर्स देऊ कि कोरोना संक्रमितको उपचार गर’
2020-07-16	Kantipur (Nepali)	‘कोरोना खर्चको लेखापरीक्षण छिटै हुन्छ’
2020-08-17	Kantipur (Nepali)	‘कोरोना नजिकै छैन सोच्नु गलत’
2020-08-31	Kantipur (Nepali)	‘कोरोना लागे बाँचिएला, खानै नपाए कसरी बाँच्ने?’
2020-06-02	Kantipur (Nepali)	‘कोरोना सँगसँगै अघि बढ्नुपर्छ’
2020-04-29	Online Khabar (Nepali)	‘कोरोनाको उपचार गर्ने भएपछि अरू बिरामी झन् सुरक्षित छन्’
2020-06-12	Kantipur (Nepali)	‘कोरोनाले मर्छ कि भुकले’
2020-05-21	Kantipur (Nepali)	‘क्वारेन्टाइन व्यवस्थित गर्नू, परीक्षण बढाउनू’
2020-06-01	Kantipur (Nepali)	‘क्वारेन्टाइनका सबैलाई परीक्षण गर्नू’
2020-06-13	Kantipur (Nepali)	‘खर्च सार्वजनिक गर्छौं, जोखिमप्रति सचेत होऔं’
2020-06-03	Kantipur (Nepali)	‘गर्भवती र सुत्केरीलाई सेवा देऊ’
2020-07-17	Kantipur (Nepali)	‘गुणस्तर नजाँची सेनिटाइजर बेच्न नदेऊ’
2020-03-23	Kantipur (Nepali)	‘चिकित्सक नै असुरक्षित’
2020-03-23	Kantipur (Nepali)	‘चिनियाँ मोडल’ अपनाउने तयारी
2020-07-11	Kantipur (Nepali)	‘छिनछिनमा दुर्व्यवहार सहनुपर्‍यो’
2020-08-01	Kantipur (Nepali)	‘ठूलाबडा’ नै लगाउँदैनन् मास्क
2020-05-09	Kantipur (Nepali)	‘ढुकुटी रित्तियो, अब थेग्न सकिन्न’
2020-08-01	Kantipur (Nepali)	‘तलब माग्दा निकालियौं’
2020-07-19	Kantipur (Nepali)	‘नीति छिटो कार्यान्वयन होस्’
2020-06-18	Kantipur (Nepali)	‘पल्टनबाट फोन आउन थाल्यो’
2020-05-08	Kantipur (Nepali)	‘पारि रहेकालाई झिकाइदेऊ’
2020-06-12	Kantipur (Nepali)	‘पीसीआर रिपोर्ट नेगेटिभ आए मात्रै घर फिर्ता’
2020-07-29	Kantipur (Nepali)	‘पीसीआरको दायरा घटाउने मनसाय छैन’
2020-06-08	Kantipur (Nepali)	‘पुनः परीक्षण नगरी पठाउनु घातक’
2020-05-17	Kantipur (Nepali)	‘पुनरुत्थान केन्द्रित नीति आएन’
2020-05-12	Kantipur (Nepali)	‘फजुल खर्च घटाऔं, ठूला आयोजनालाई निरन्तरता दिऊँ’

Date of publication (yyyy/mm/dd)	Name of news media (select from drop-down menu)	Title of news/headline
2020-07-03	Kantipur (Nepali)	‘बढी मारमा खेल पत्रकारिता’
2020-06-02	Kantipur (Nepali)	‘बन्द प्रशिक्षण तत्काल सुरु गरौं’
2020-03-17	Kantipur (Nepali)	‘बिनामास्क गाडी नचढ्नुस्’
2020-08-03	Kantipur (Nepali)	‘भयावह अवस्था अब आउँछ’
2020-05-05	Kantipur (Nepali)	‘मानव जीवन सबैभन्दा महत्त्वपूर्ण’
2020-05-20	Kantipur (Nepali)	‘मापदण्ड पुर्‍याए होम क्वारेन्टाइन पनि सुरक्षित’
2020-06-14	Kantipur (Nepali)	‘मास्क लगाए सुरक्षित हुन सकिन्छ’
2020-07-26	Kantipur (Nepali)	‘मृत्युदर फरक पर्न सक्छ’
2020-06-11	Kantipur (Nepali)	‘मैले भनेपछि भैगो त!’
2020-05-05	Kantipur (Nepali)	‘राजनीतिमा लाज लाग्ने कुरा भइरहेको छ’
2020-06-15	Kantipur (Nepali)	‘राहत र उपचारमा बेवास्ता भयो’
2020-06-17	Kantipur (Nepali)	‘रोकथाममा गम्भीर होऊ’
2020-06-12	Kantipur (Nepali)	‘लकडाउन खुलेपछि मात्रै कर तिर्न लगाउनू’
2020-06-02	Kantipur (Nepali)	‘लकडाउनबारे पुनर्विचार होस्’
2020-05-08	Kantipur (Nepali)	‘लकडाउनले संक्रमणलाई धकेल्यो, रोकेन’
2020-06-11	Kantipur (Nepali)	‘लडकाउन गरिरहे पनि बढी संक्रमित देखिन सक्छन्’
2020-07-11	Kantipur (Nepali)	‘वित्तीय सुविधा नपाए गाडी चलाउँदैनौं’
2020-05-26	Kantipur (Nepali)	‘विदेशका नेपालीलाई प्राथमिकता हेरेर ल्याउँछौं’
2020-04-07	Online Khabar (Nepali)	‘विदेशबाट आएकाहरु स्वघोषणा गरेर स्वास्थ्यकर्मीको सम्पर्कमा जानुस्’
2020-05-05	Kantipur (Nepali)	‘विद्यार्थी भर्ना नलिनू’
2020-05-04	Kantipur (Nepali)	‘विद्यार्थी भर्ना वैशाख २६ बाट’
2020-05-28	Kantipur (Nepali)	‘संक्रमण भयावह हुने जोखिम’
2020-06-03	Kantipur (Nepali)	‘संक्रमण व्यापक भए सामूहिक प्रतिरक्षा’
2020-05-31	Kantipur (Nepali)	‘संक्रमणका केन्द्र’ क्वारेन्टाइन
2020-07-09	Kantipur (Nepali)	‘संक्रमित बढे भेन्टिलेटर र आईसीयू पुग्दैन’
2020-03-18	Kantipur (Nepali)	‘सबै सवारी चलाऔं’
2020-06-07	Kantipur (Nepali)	‘सरकार बाँसुरी बजाएर नबसोस्’
2020-05-20	Online Khabar (Nepali)	‘सामान्य संक्रमित’ लाई अस्पताल बाहिरै राखिने
2020-05-12	Kantipur (Nepali)	‘सीमामा रोक्दा संक्रमण भित्रभित्रै फैलिने डर’
2020-06-01	Kantipur (Nepali)	‘सुत्केरी र गर्भवती बचाऊ’
2020-04-05	Online Khabar (Nepali)	‘सुदूरपश्चिम उच्च जोखिममा छ, राष्ट्रिय संकट आउन सक्छ’
2020-06-11	Kantipur (Nepali)	‘स्वास्थ्य सामग्री खरिद पारदर्शी’
2020-04-25	Kantipur (Nepali)	‘स्वास्थ्य सुरक्षा अपनाएर लकडाउन खुकुलो पार्ने तयारी’
2020-07-07	Kantipur (Nepali)	‘स्वास्थ्यकर्मीको सुविधा नकाट’
2020-06-03	Kantipur (Nepali)	‘होटल टिकाउने बजेट आएन’
2020-04-06	Kantipur (Nepali)	र्‍यापिड र पीसीआर किटबाट परीक्षण गरिन
2020-04-15	Kantipur (Nepali)	र्‍यापिड किटबाट ५६ जिल्लामा परीक्षण
2020-04-07	Kantipur (Nepali)	र्‍यापिड किटबाटै परीक्षण तयार
2020-04-17	Kantipur (Nepali)	ंक्रमितसँगै आएका युवक एक महिनापछि क्वारेन्टाइनमा
2020-04-23	Kantipur (Nepali)	‘राज्यले अपमान मात्र गर्‍यो’
2020-04-06	Kantipur (Nepali)	‘स्वास्थ्यकर्मीलाई हौसला चाहिएको छ
2020-04-18	Kantipur (Nepali)	‘सरकार निमुखाको दर्शक भयो’
2020-04-14	Kantipur (Nepali)	‘सांसदको बजेटले अस्पताल बनाऔं’

Date of publication (yyyy/mm/dd)	Name of news media (select from drop-down menu)	Title of news/headline
2020-04-17	Kantipur (Nepali)	'बसौं खाना छैन, जाऔं दिदैनन्'
2020-03-24	Kantipur (Nepali)	'एकअर्काबाट दूरी राखौं, घरमै बसौं'
2020-04-22	Himalayan Times (English)	1,616 swabs tested
2020-07-14	Himalayan Times (English)	1,705 patients of coronavirus disease recover in a day
2020-05-20	Online Khabar (Nepali)	१० जनामा कोरोना भेटिएपछि कचनकवलका ५ वडा सिल
2020-06-10	Kantipur (Nepali)	१० दिनसम्म रिपोर्ट नआएपछि २४ जना भागे
2020-06-15	Himalayan Times (English)	11 COVID-19 patients sent home before test reports in Kanchanpur
2020-04-07	Himalayan Times (English)	11 discharged from isolation, 69 test negative for COVID-19
2020-06-02	Himalayan Times (English)	12 migrants returning from India killed in accident
2020-08-30	Himalayan Times (English)	12 succumb to coronavirus, 884 new cases
2020-03-25	Himalayan Times (English)	125 who flew with COVID-19 patient traced
2020-08-11	Kantipur (Nepali)	१२९ जनाले गरे कोरोना बिमा दाबी
2020-03-14	Himalayan Times (English)	13,000 people screened for coronavirus in Gaur
2020-03-27	Himalayan Times (English)	137 foreigners stranded on trekking trails rescued in two days
2020-04-13	Online Khabar (Nepali)	१३औं संक्रमितको रिपोर्ट : र्‍यापिड टेस्टमा नेगेटिभ, पीसीआर टेस्ट पोजेटिभ
2020-08-31	Himalayan Times (English)	14 deaths in 24 hours
2020-06-10	Himalayan Times (English)	14 health workers infected with coronavirus so far
2020-08-05	Himalayan Times (English)	14 hospitals for swab collection
2020-05-13	Himalayan Times (English)	14 quarantined
2020-06-24	Kantipur (Nepali)	१४ दिन कटाउँदै, पठाउँदै
2020-02-25	Kantipur (Nepali)	१४ दिन क्वारेन्टाइन 'पर्याप्त' होइन
2020-03-07	Himalayan Times (English)	15 health desks to be set up in Saptari district
2020-05-05	Himalayan Times (English)	15 Nepalgunj COVID patients admitted to hospital for treatment
2020-03-19	Kantipur (Nepali)	१५ एयरलाइन्सका उडान रोकिने
2020-03-16	Kantipur (Nepali)	१५५ आइसोलेटेड शय्या तयार, २३५ आईसीयू थपिने
2020-04-20	Himalayan Times (English)	15-point suggestion put forth to manage COVID-19 situation
2020-08-02	Himalayan Times (English)	161 beat COVID-19

Date of publication (yyyy/mm/dd)	Name of news media (select from drop-down menu)	Title of news/headline
2020-02-03	Himalayan Times (English)	180 Nepalis in China plead to return home Govt likely to airlift them this week, but evacuee quarantine location yet to be finalised
2020-03-13	Himalayan Times (English)	19-bed isolation ward in Bharatpur Hospital
2020-05-24	Kantipur (Nepali)	२ दिनमै भित्रिए साढे १० हजार
2020-07-02	Kantipur (Nepali)	२ हजार ६१ बालबालिकामा संक्रमण
2020-06-04	Himalayan Times (English)	200-plus cases in a day, third day in a row
2020-04-12	Himalayan Times (English)	21 swab samples sent to Pokhara for test
2020-06-08	Kantipur (Nepali)	२१३ संक्रमित थपिए, निको हुने ४६७
2020-05-24	Himalayan Times (English)	22 health workers, including 5 doctors, to be kept in isolation
2020-05-15	Kantipur (Nepali)	२२ जिल्लामा पुग्यो संक्रमण
2020-07-05	Himalayan Times (English)	232 new COVID-19 cases detected
2020-05-13	Kantipur (Nepali)	२४ घण्टामा ७ हजार जना उपत्यका भित्रिए
2020-04-23	Himalayan Times (English)	249 undergo RDT
2020-06-02	Himalayan Times (English)	25 COVID-19 patients recover in Bhairahawa
2020-04-26	Himalayan Times (English)	25 quarantined
2020-05-29	Online Khabar (Nepali)	२५ जनाका लागि क्वारेन्टिनको तयारी, संक्रमित नै सय पुगे
2020-03-19	Kantipur (Nepali)	२५ जनाभन्दा बढी भेला हुन प्रतिबन्ध
2020-06-12	Himalayan Times (English)	250 more diagnosed with novel coronavirus disease
2020-03-29	Himalayan Times (English)	254 foreign returnees in home quarantine
2020-07-09	Himalayan Times (English)	255 more diagnosed with coronavirus in Nepal, 253 discharged
2020-04-12	Himalayan Times (English)	259 coronavirus suspects in Sudurpaschim test negative
2020-06-20	Himalayan Times (English)	26 coronavirus patients discharged
2020-06-05	Himalayan Times (English)	27 COVID-19 patients discharged
2020-07-06	Himalayan Times (English)	27 more diagnosed with COVID in Kathmandu
2020-05-24	Himalayan Times (English)	27 patients undergoing COVID-19 treatment in Kathmandu valley
2020-06-30	Kantipur (Nepali)	270 Shops in Thamel Area are Displaced
2020-06-14	Himalayan Times (English)	273 new cases of coronavirus today
2020-06-06	Himalayan Times (English)	278 more infected with novel coronavirus disease today

Date of publication (yyyy/mm/dd)	Name of news media (select from drop-down menu)	Title of news/headline
2020-06-11	Himalayan Times (English)	279 more test positive for novel coronavirus
2020-06-27	Himalayan Times (English)	27th fatality
2020-06-10	Himalayan Times (English)	28 coronavirus patients recover
2020-03-05	Himalayan Times (English)	28 isolation wards prepared in Province 1
2020-05-28	Kantipur (Nepali)	२८ टन स्वास्थ्य सामग्री आउँदै
2020-06-28	Himalayan Times (English)	28th fatality
2020-06-22	Himalayan Times (English)	3 more Nepalis die of COVID-19 in Qatar
2020-04-21	Kantipur (Nepali)	घर फर्किंदा २ जनाको बाटैमा मृत्य
2020-04-04	Kantipur (Nepali)	घर फर्कन ज्यानको बाजी
2020-04-16	Kantipur (Nepali)	घर पुग्ने आत्तुरीले महाकालीमा हेलिए
2020-04-22	Kantipur (Nepali)	घरजानेकोलको
2020-06-12	Himalayan Times (English)	30 patients recover
2020-04-30	Himalayan Times (English)	30 quarantined
2020-04-17	Online Khabar (Nepali)	३० हजार र्‍यापिड टेस्ट किट पठाइयो
2020-06-19	Kantipur (Nepali)	३० हजार संक्रमितका लागि सामग्री जोहो
2020-05-22	Himalayan Times (English)	300-bed quarantines to be set up in Jajarkot
2020-03-22	Himalayan Times (English)	30-bed isolation ward in Jajarkot District Hospital
2020-07-03	Himalayan Times (English)	31 more infected with COVID-19 in valley
2020-06-12	Himalayan Times (English)	31 recovered patients discharged in Karnali
2020-06-26	Kantipur (Nepali)	३१२ जना डिस्चार्ज
2020-06-09	Himalayan Times (English)	314 new COVID-19 cases come to light
2020-06-07	Himalayan Times (English)	323 new COVID cases, two die of infection
2020-06-21	Himalayan Times (English)	331 new coronavirus cases detected
2020-04-26	Kantipur (Nepali)	३५ आए, ३६ गए
2020-08-23	Online Khabar (Nepali)	३५ जिल्लामा पूर्ण र १२ मा आंशिक निषेधाज्ञा
2020-06-02	Kantipur (Nepali)	३५ हजारको टिकटलाई १ लाख
2020-07-07	Himalayan Times (English)	35th fatality
2020-03-26	Kantipur (Nepali)	घट्न थाल्यो आयात
2020-06-17	Kantipur (Nepali)	४ जिल्ला उच्च जोखिममा
2020-04-26	Online Khabar (Nepali)	४० हजार र्‍यापिड टेस्ट भइसक्यो, अझै परीक्षण भएको छैन गुणस्तर

Date of publication (yyyy/mm/dd)	Name of news media (select from drop-down menu)	Title of news/headline
2020-06-22	Himalayan Times (English)	421 more hospitalised with novel coronavirus Country’s COVID count rockets past 9,000
2020-04-18	Himalayan Times (English)	421 prisoners from across the country released amid coronavirus scare
2020-06-15	Himalayan Times (English)	425 more diagnosed with coronavirus
2020-05-26	Himalayan Times (English)	43 corona patients discharged
2020-08-28	Kantipur (Nepali)	४५५ सेनिटाइजर गुणस्तरहीन’
2020-07-08	Kantipur (Nepali)	४६ प्रतिशत निको भए
2020-06-29	Himalayan Times (English)	463 more test positive for novel coronavirus
2020-06-30	Himalayan Times (English)	476 more diagnosed with coronavirus
2020-07-03	Himalayan Times (English)	49-day-old infant dies of the virus
2020-03-15	Kantipur (Nepali)	५ अस्पताल कोरोना केन्द्रित गरिने
2020-03-23	Himalayan Times (English)	5,000-bed quarantine facilities to be set up in valley
2020-05-04	Kantipur (Nepali)	५० प्रतिशतभन्दा बढी पारिश्रमिक दिन कठिन’
2020-05-11	Himalayan Times (English)	500 undergo RDT, 25 PCR tests in Bhojpur
2020-08-26	Online Khabar (Nepali)	५०० बेडको आइसोलेसन सेन्टर बनाउन सांसदको नेतृत्वमा समिति
2020-05-13	Kantipur (Nepali)	५१ दिनपछि खुल्यो सेयर बजार
2020-06-23	Himalayan Times (English)	535 more diagnosed with contagion
2020-04-15	Himalayan Times (English)	54 people who came into contact with COVID-19 patient quarantined
2020-06-28	Himalayan Times (English)	554 new COVID-19 cases reported across country
2020-04-20	Kantipur (Nepali)	छोराछोरी आऊ भन्छन्, कसरी जानु?’
2020-05-28	Kantipur (Nepali)	६ लाखको कोरोना परीक्षण गर्न ६ महिना लाग्ने
2020-08-28	Himalayan Times (English)	6,000 more beds for COVID patients in valley
2020-02-10	Online Khabar (Nepali)	60 health workers will be deployed for supervision of students brought from China
2020-05-15	Himalayan Times (English)	600 swab samples collected from Kalimati market
2020-03-23	Himalayan Times (English)	60-bed quarantine coming up in Dhankuta
2020-03-24	Himalayan Times (English)	62 isolation wards prepared in Karnali
2020-08-23	Himalayan Times (English)	634 new cases of coronavirus, nine succumb to infection
2020-03-22	Himalayan Times (English)	64 Nepalis quarantined for violating immigration rules

Date of publication (yyyy/mm/dd)	Name of news media (select from drop-down menu)	Title of news/headline
2020-07-29	Himalayan Times (English)	65-year-old dies
2020-07-19	Kantipur (Nepali)	६६% निको
2020-08-03	Himalayan Times (English)	68-year-old woman from Birgunj dies
2020-02-05	Kantipur (Nepali)	७ लाख मास्क निर्यात
2020-03-05	Kantipur (Nepali)	७० हजार विदेशी यात्रु घटे
2020-06-19	Kantipur (Nepali)	७०५ पत्रकारको कोरोना बिमा
2020-03-18	Himalayan Times (English)	70-bed hospital to be readied in 72 hrs
2020-03-11	Online Khabar (Nepali)	71 workers working at Pokhara Airport in self-quarantine
2020-03-11	Kantipur (Nepali)	७१ चिनियाँ क्वारेन्टाइनमा
2020-04-24	Himalayan Times (English)	72 kept in quarantine
2020-03-20	Kantipur (Nepali)	७२ घण्टामै ५० शय्याको अस्पताल
2020-06-16	Kantipur (Nepali)	७३ जिल्लामा सार्वजनिक सेवा सुरु
2020-06-08	Kantipur (Nepali)	७५ दिनपछि खुल्यो सुनचाँदी बजार
2020-06-20	Himalayan Times (English)	75-year-old lung cancer patient recovers
2020-06-04	Himalayan Times (English)	76-year-old man succumbs to the virus
2020-04-27	Himalayan Times (English)	77 quarantined
2020-08-23	Kantipur (Nepali)	७८ हजार श्रमिक स्वदेश फिर्ने प्रतीक्षामा
2020-04-22	Kantipur (Nepali)	डस्चार्ज भए सुदूरपश्चिमका पहिलो संक्रमित
2020-03-14	Kantipur (Nepali)	८०% संक्रमित विशेष उपचारबिनै निको
2020-07-01	Kantipur (Nepali)	800 Swaps are on Queue as Testing Mechine is Broken
2020-04-21	Himalayan Times (English)	81 per cent COVID patients in Nepal asymptomatic
2020-07-01	Himalayan Times (English)	83-year-old beats coronavirus
2020-03-22	Himalayan Times (English)	90 beds for COVID-19 suspects in Panchthar
2020-07-01	Kantipur (Nepali)	90 Percent Shopping Malls in Valley Start Operating
2020-07-09	Himalayan Times (English)	92pc COVID patients recover, return home in Rautahat
2020-05-30	Himalayan Times (English)	97 quarantined
2020-07-10	Kantipur (Nepali)	९९.७५ कोरोना संक्रमितमा लक्षण छैन
2020-05-01	Kantipur (Nepali)	A Woman Died In Isolation
2020-04-11	Kantipur (Nepali)	बढाइँदै परीक्षण
2020-06-30	Himalayan Times (English)	Aanbukhairani RM closed for a week
2020-06-05	Himalayan Times (English)	Aathrai RM sealed

Date of publication (yyyy/mm/dd)	Name of news media (select from drop-down menu)	Title of news/headline
2020-06-23	Online Khabar (Nepali)	About 260 million rupees has been spent on corona prevention in Province 1
2020-04-19	Himalayan Times (English)	Action taken against ambulance driver
2020-07-30	Himalayan Times (English)	Activist Iih refuses to be hospitalised
2020-08-09	Himalayan Times (English)	Activist Iih to end hunger strike today
2020-06-12	Himalayan Times (English)	Activists fear quarantines turning into COVID hotspots
2020-07-02	Himalayan Times (English)	ADB approves $3m grant from APDRF
2020-06-19	Himalayan Times (English)	ADB, UNICEF join hands to supply medical equipment
2020-07-09	Himalayan Times (English)	Adequate social security key to minimise impact of COVID-19, says PM Oli
2020-04-23	Kantipur (Nepali)	बाडे, उसैबाट डर
2020-04-20	Kantipur (Nepali)	बालबच्चा बचाउन घस्रिदै घरतिर
2020-03-25	Kantipur (Nepali)	बागलुङ र सप्तरीमा सेना परिचालन
2020-06-11	Himalayan Times (English)	Agitated valley traders protest as government halts market operation
2020-06-03	Himalayan Times (English)	AHW attacked
2020-04-11	Himalayan Times (English)	All COVID-19 patients in Nepal normal
2020-04-26	Himalayan Times (English)	All flights to remain suspended till May 15
2020-07-21	Himalayan Times (English)	All flights to resume from August 17
2020-04-29	Himalayan Times (English)	All Gandaki districts to have ICU
2020-08-25	Himalayan Times (English)	All local govts inside valley to remain shut until August 31
2020-05-01	Kantipur (Nepali)	All Migrants are Returned from Employment
2020-03-11	Online Khabar (Nepali)	All political parties are with government in the fight against Corona
2020-07-01	Online Khabar (Nepali)	All the offices of the Rural municipality are closed after the health workers confirmed with corona
2020-06-04	Himalayan Times (English)	Alternative approaches to dealing with coronavirus
2020-04-08	Himalayan Times (English)	Ambulance drivers at risk of contracting COVID-19
2020-04-17	Himalayan Times (English)	Ambulance stopped
2020-01-27	Himalayan Times (English)	American of Nepali origin screened at TIA, admitted to STIDH
2020-06-30	Kantipur (Nepali)	An Alarming Rate of Suicide

Date of publication (yyyy/mm/dd)	Name of news media (select from drop-down menu)	Title of news/headline
2020-07-16	Himalayan Times (English)	Another 697 recover from respiratory contagion
2020-01-27	Online Khabar (Nepali)	Another corona virus infection in Nepal
2020-04-19	Kantipur (Nepali)	aofgd} ;Gb]x
2020-06-24	Himalayan Times (English)	Apex court stays COVID quarantine guideline
2020-06-16	Himalayan Times (English)	APN's initiative
2020-04-28	Himalayan Times (English)	Area sealed after COVID case found
2020-08-22	Himalayan Times (English)	Arrange 10,000 ventilators, House panel tells govt
2020-04-25	Himalayan Times (English)	ASI injured in dispute over relief distribution in Debgaun
2020-06-26	Himalayan Times (English)	Asymptomatic cases being sent home in Bajura district
2020-04-01	Himalayan Times (English)	Attending corona patients a must for private hospitals: SC
2020-07-10	Himalayan Times (English)	Authorities provide face masks to locals
2020-04-07	Kantipur (Nepali)	देउवाद्वारा राष्ट्रिय सहमतिको आह्वान
2020-04-16	Himalayan Times (English)	BAC agrees to provide fund to fight against virus
2020-03-06	Himalayan Times (English)	Bagmati govt urges people not to shake hands
2020-08-24	Himalayan Times (English)	Bagmati Province to set up PCR labs in three more hospitals as virus cases increase
2020-06-19	Himalayan Times (English)	Bajura ANM exudes sense of service in the time of COVID crisis
2020-04-15	Himalayan Times (English)	Bajura lacks essential medical equipment
2020-05-30	Himalayan Times (English)	Bajura quarantine facilities in a shambles, sans health workers
2020-05-01	Himalayan Times (English)	Bajura quarantine gets sub-standard rice
2020-06-04	Himalayan Times (English)	Bandipur RM sealed
2020-08-10	Himalayan Times (English)	Bank sealed after employees contract virus
2020-03-24	Himalayan Times (English)	Banke residents in home quarantine
2020-05-18	Himalayan Times (English)	Banke youth succumbs to novel coronavirus disease
2020-06-30	Himalayan Times (English)	Banks mull slashing interest rate on deposits from new fiscal year, Page 5
2020-07-21	Himalayan Times (English)	Bara DAO issues prohibitory order as virus cases increase

Date of publication (yyyy/mm/dd)	Name of news media (select from drop-down menu)	Title of news/headline
2020-04-10	Himalayan Times (English)	Bara families get rotten rice in the name of relief
2020-06-14	Himalayan Times (English)	Bara locals panic after COVID-19 confirmed as cause of five-year-old boy’s death
2020-08-11	Himalayan Times (English)	Bara to set up 400-bed isolation ward
2020-03-06	Online Khabar (Nepali)	Bhaktapur festival postponed due to corona
2020-02-06	Himalayan Times (English)	Bhaktapur locals protest against likely quarantine site
2020-02-01	Online Khabar (Nepali)	Bharatpur Hospital has set up an isolation ward to treat those infected with the corona virus
2020-07-10	Himalayan Times (English)	Bharatpur Hospital providing dialysis service to COVID patients
2020-03-16	Himalayan Times (English)	Bheri Hospital ill-prepared to tackle possible coronavirus outbreak
2020-05-10	Himalayan Times (English)	Bheri Municipality provides relief materials to needy
2020-08-26	Himalayan Times (English)	Bhojpur entry points sealed for 15 days
2020-06-23	Himalayan Times (English)	Bhojpur lawmaker in quarantine facility
2020-03-27	Himalayan Times (English)	Bir Hospital launches phone consultation service
2020-04-11	Himalayan Times (English)	Bir hospital starts COVID-19 tests
2020-07-27	Himalayan Times (English)	Biratnagar shuts services other than essential ones
2020-02-09	Online Khabar (Nepali)	Black market of mask in the name of Corona
2020-04-13	Kantipur (Nepali)	दक्षिणी सीमा ‘ सिल’ गर्न सुझाव
2020-01-20	Himalayan Times (English)	Blood, throat swab sent to HK for diagnosis
2020-04-16	Himalayan Times (English)	Border points closed
2020-04-21	Himalayan Times (English)	Border points closed in Khotang
2020-04-06	Himalayan Times (English)	Borders closed
2020-08-09	Himalayan Times (English)	BPKIHS faces challenge managing COVID-19 cases
2020-06-16	Himalayan Times (English)	Bread winners return home, kin living on loan
2020-06-19	Himalayan Times (English)	Budget for improvement of quarantine facilities
2020-08-26	Himalayan Times (English)	Budhanilkantha Municipality to set up 50-bed isolation facility
2020-05-31	Himalayan Times (English)	Budhiganga Municipality to insure staffers, health workers

Date of publication (yyyy/mm/dd)	Name of news media (select from drop-down menu)	Title of news/headline
2020-07-30	Himalayan Times (English)	Budhinanda Municipality charging Rs 1,400 for RDT
2020-07-02	Himalayan Times (English)	Business activities closed in Khotang
2020-03-24	Himalayan Times (English)	Businesses closed
2020-06-25	Himalayan Times (English)	Businesses start paying taxes though not obliged to
2020-06-03	Himalayan Times (English)	Businesses, general public told to clear tax dues by June 7
2020-03-15	Online Khabar (Nepali)	Businessmen of Rupendehi ready to make quarantine in their hotel
2020-06-29	Online Khabar (Nepali)	But feel hungry!
2020-03-07	Himalayan Times (English)	Call for House panel to monitor preparedness to fight the virus
2020-05-18	Himalayan Times (English)	Call to test surgery patients, pregnant women
2020-06-18	Himalayan Times (English)	Can COVID-19 cause long-term effects?
2020-08-11	Himalayan Times (English)	Capital areas sealed after COVID cases rise
2020-07-14	Himalayan Times (English)	Capital budget spending at two-decade low of 40 per cent
2020-08-21	Himalayan Times (English)	Capital city to get 5,000-bed isolation centre
2020-05-24	Himalayan Times (English)	Cardiology, cath lab services halted in MCVTC
2020-06-18	Himalayan Times (English)	Cases surge past 7,000 after unprecedented intraday spike
2020-02-19	Online Khabar (Nepali)	CDOs, ministers, students and parents are feeling relief at the quarantine site
2020-04-16	Kantipur (Nepali)	आरडीटी पोजिटिभ भएकाको कन्ट्याक्ट ट्रेसिङ हुनुपछ
2020-04-18	Kantipur (Nepali)	आरडीटी पोजिटिभको स्वाब संकलनमै३ दिन
2020-04-14	Kantipur (Nepali)	आरडीटीबाट यसरी गरिन्छ परीक्षण
2020-04-22	Kantipur (Nepali)	आरडीटीमा नेगेटिभ, पीसीआरमा पोजिटिभ
2020-04-16	Kantipur (Nepali)	आरडीटीमा पोजिटिभ, स्वाब परीक्षणमा नेगेटिभ
2020-04-12	Kantipur (Nepali)	आरडीटीकै भरमा घर
2020-04-13	Kantipur (Nepali)	ओम्नीबाट टेन्डरबाहेकको किट खरिद
2020-04-11	Kantipur (Nepali)	ओली-मोदी टेलिफोन वाता
2020-04-17	Kantipur (Nepali)	आपत्मा सघाउँदै आविष्कार
2020-04-06	Kantipur (Nepali)	आइसोलेसनबाट भागेका पक्राउ
2020-05-17	Himalayan Times (English)	Chandragiri Municipality sealed after five confirmed COVID-19 cases
2020-06-15	Himalayan Times (English)	Chief Minister Gurung inspects COVID laboratory in Pokhara
2020-02-02	Himalayan Times (English)	China assures it is taking proactive measures

Date of publication (yyyy/mm/dd)	Name of news media (select from drop-down menu)	Title of news/headline
2020-06-01	Himalayan Times (English)	China cities aid Pokhara metropolis
2020-02-18	Himalayan Times (English)	China evacuees show no symptom of COVID-19
2020-06-17	Himalayan Times (English)	China provides health materials worth millions
2020-07-23	Himalayan Times (English)	Chinese national dies of COVID in Nepal
2020-03-11	Himalayan Times (English)	Chinese quarantined
2020-03-17	Himalayan Times (English)	Chitwan DAO devising plan to fight COVID-19
2020-07-02	Himalayan Times (English)	Chitwan hotels turn into quarantine facilities
2020-05-20	Himalayan Times (English)	Chitwan to set up quarantine centres in all wards
2020-04-09	Kantipur (Nepali)	अपरसन थिएटरमा बिरामीले खोकेपछि...
2020-06-14	Himalayan Times (English)	CM Employment Programme benefits poor in Jumla district
2020-06-02	Himalayan Times (English)	CNI seeks collective effort to fight crisis
2020-04-18	Kantipur (Nepali)	अलपत्र परकालाई राहत दिन निर्देशन
2020-04-18	Kantipur (Nepali)	अलपत्रलाई घर पुऱ्याउनू : सर्वोच्च तर प्रधानमन्त्री भन्छन्- जो जहा छन् त्यहीँ रोक्न
2020-03-14	Himalayan Times (English)	Committee formed to prevent virus spread
2020-08-18	Himalayan Times (English)	Community spread
2020-08-07	Himalayan Times (English)	Community spread feared in Siraha
2020-08-15	Himalayan Times (English)	Community spread of COVID-19 feared in Damak Municipality
2020-04-03	Himalayan Times (English)	Concern raised over Nepalis stranded on border
2020-01-26	Online Khabar (Nepali)	Congress demands protection from corona virus
2020-01-31	Online Khabar (Nepali)	Congress demands rescue of Nepalis from Wuhan affected by corona virus
2020-06-04	Himalayan Times (English)	Conquering COVID-19 strategic plan released
2020-06-01	Himalayan Times (English)	Contact tracing of six passengers begins in Gandaki
2020-07-07	Himalayan Times (English)	Contagion confirmed in seven NAC staffers
2020-06-16	Himalayan Times (English)	Coordination panel formed
2020-06-28	Himalayan Times (English)	Cop tests positive for coronavirus

Date of publication (yyyy/mm/dd)	Name of news media (select from drop-down menu)	Title of news/headline
2020-03-28	Himalayan Times (English)	Co-passengers of COVID patient who boarded Air Arabia being tracked down
2020-01-25	Kantipur (Nepali)	Corana Virus: Becareful but not Terrified
2020-06-25	Online Khabar (Nepali)	Corona infection confirmed in Dailekh youth who committed suicide
2020-05-31	Himalayan Times (English)	Corona patients forced to spend night outside hospital
2020-01-24	Online Khabar (Nepali)	Corona virus infection was also found in Nepal
2020-01-28	Online Khabar (Nepali)	Corona virus: Nepal needs to be alert!
2020-01-23	Online Khabar (Nepali)	Corona virus: There is no substitute for caution
2020-01-26	Online Khabar (Nepali)	Corona Virus: What are being done to prevent infection in Nepal?
2020-06-25	Himalayan Times (English)	Coronavirus cases continue to surge as 629 infected in a day
2020-06-10	Himalayan Times (English)	Coronavirus cases cross 4,000 in Nepal
2020-06-13	Himalayan Times (English)	Coronavirus cases surge past 5,000
2020-06-20	Himalayan Times (English)	Coronavirus cases surge past 8,000
2020-08-29	Himalayan Times (English)	Coronavirus claims 12 more lives
2020-08-13	Himalayan Times (English)	Coronavirus claims eight lives in a day
2020-06-24	Himalayan Times (English)	Coronavirus claims fifth life in Karnali
2020-07-05	Himalayan Times (English)	Coronavirus detected in valley’s drainage water
2020-07-04	Himalayan Times (English)	Coronavirus fatality in Kathmandu
2020-01-26	Himalayan Times (English)	Coronavirus fear grips tourism sector
2020-01-25	Himalayan Times (English)	Coronavirus has no boundaries, says Health Minister Dhakal
2020-02-03	Himalayan Times (English)	Coronavirus hits Nepal-China trade
2020-03-12	Himalayan Times (English)	Coronavirus impacts Sauraha hoteliers
2020-08-11	Himalayan Times (English)	Coronavirus in APF personnel increases risk of community spread in Jhapa
2020-06-23	Himalayan Times (English)	Coronavirus infected persons moving freely in Bajura
2020-03-14	Himalayan Times (English)	Coronavirus infection threat spreads panic
2020-06-08	Himalayan Times (English)	Coronavirus infects 213 more, Nepal count 3,448
2020-06-06	Himalayan Times (English)	Coronavirus insurance policy gets continuity

Date of publication (yyyy/mm/dd)	Name of news media (select from drop-down menu)	Title of news/headline
2020-08-06	Himalayan Times (English)	Coronavirus Insurance Programme formally launched
2020-05-14	Himalayan Times (English)	Coronavirus patient discharged from hospital after recovery
2020-03-08	Himalayan Times (English)	Coronavirus screening starts at Simara Airport
2020-07-04	Himalayan Times (English)	Coronavirus spreads in community
2020-02-13	Himalayan Times (English)	Coronavirus suspect goes missing
2020-01-29	Himalayan Times (English)	Coronavirus suspects test negative
2020-04-16	Himalayan Times (English)	Coronavirus threat affects immunisation campaign
2020-03-03	Himalayan Times (English)	Coronavirus wards to be set up
2020-06-22	Himalayan Times (English)	Country faces challenges of COVID-19, monsoon related disasters
2020-06-24	Himalayan Times (English)	Country's COVID count crosses 10,000
2020-07-13	Himalayan Times (English)	COVID cases 'on a decline', but EDCD suspects community spread in valley
2020-06-19	Himalayan Times (English)	Covid cases continue to surge exponentially
2020-07-10	Himalayan Times (English)	COVID cases decline mysteriously
2020-07-16	Himalayan Times (English)	COVID claims 36-year-old
2020-06-01	Himalayan Times (English)	COVID claims life of a two-year-old girl
2020-07-24	Himalayan Times (English)	COVID claims one
2020-06-19	Himalayan Times (English)	COVID confirmed in man who killed self
2020-06-29	Himalayan Times (English)	COVID crisis: Banks to adopt austerity measures, Page 5
2020-05-26	Himalayan Times (English)	COVID fatality: Body handover kicks up row
2020-08-10	Himalayan Times (English)	COVID fear looms large with more people entering Manang
2020-08-31	Himalayan Times (English)	COVID infected woman dies
2020-08-21	Himalayan Times (English)	COVID infection spreads at community level in Jhapa
2020-08-10	Himalayan Times (English)	COVID patient dies

Date of publication (yyyy/mm/dd)	Name of news media (select from drop-down menu)	Title of news/headline
2020-08-03	Himalayan Times (English)	COVID patient recovers after plasma therapy
2020-05-21	Himalayan Times (English)	COVID patient stable
2020-06-03	Himalayan Times (English)	COVID patients quarantined for want of isolation wards
2020-06-07	Himalayan Times (English)	COVID recovery
2020-08-12	Himalayan Times (English)	COVID scare spoils Krishna Janmashtami festival
2020-08-12	Himalayan Times (English)	COVID spreads at community level in Rautahat
2020-06-24	Himalayan Times (English)	COVID test halted at Narayani Hospital for past three days
2020-04-17	Himalayan Times (English)	COVID test: Question mark over efficacy of labs outside capital
2020-07-03	Himalayan Times (English)	COVID tests not expanded in Sudurpaschim
2020-07-05	Himalayan Times (English)	COVID victim’s body laid to rest amid obstruction
2020-06-08	Himalayan Times (English)	COVID vistims cremated before the result came
2020-03-14	Himalayan Times (English)	COVID-19 call centre
2020-07-20	Himalayan Times (English)	COVID-19 cases up in Saptari
2020-05-31	Himalayan Times (English)	COVID-19 claims Dailekh youth
2020-06-26	Himalayan Times (English)	COVID-19 confirmed in Dailekh suicide case
2020-07-14	Himalayan Times (English)	COVID-19 control, disaster management top priorities
2020-06-04	Himalayan Times (English)	COVID-19 curve flattening in Narainapur
2020-07-06	Himalayan Times (English)	COVID-19 death toll of Nepalis abroad climbs to 143
2020-07-26	Himalayan Times (English)	COVID-19 detected in community
2020-04-24	Himalayan Times (English)	COVID-19 fear in army barracks after Bhulke residents test positive
2020-03-08	Himalayan Times (English)	COVID-19 fear spurs hoarding of face masks
2020-07-18	Himalayan Times (English)	COVID-19 hospital gets 40-bed isolation ward
2020-04-28	Himalayan Times (English)	COVID-19 hospital operational in BPKIHS

Date of publication (yyyy/mm/dd)	Name of news media (select from drop-down menu)	Title of news/headline
2020-06-30	Himalayan Times (English)	COVID-19 infected participating in social functions
2020-08-20	Himalayan Times (English)	COVID-19 infected woman dies in Dharan
2020-08-08	Himalayan Times (English)	COVID-19 infected youth dies
2020-07-17	Himalayan Times (English)	COVID-19 infected youth’s body disposed
2020-03-29	Himalayan Times (English)	COVID-19 patient accuses doctors of denying him treatment
2020-03-29	Himalayan Times (English)	COVID-19 patient confident he will recover soon
2020-05-16	Himalayan Times (English)	COVID-19 patients shifted to corona special hospital
2020-03-10	Himalayan Times (English)	Covid-19 scare keeps most revellers indoors
2020-04-09	Himalayan Times (English)	COVID-19 sledgehammers economic activities
2020-08-07	Himalayan Times (English)	COVID-19 spread in community thru factory in Dharan
2020-03-16	Himalayan Times (English)	COVID-19 suspect sent to Kathmandu
2020-08-30	Himalayan Times (English)	COVID-19 tracing easier said than done
2020-06-20	Himalayan Times (English)	COVID-19 victim laid to rest in Banke
2020-03-29	Himalayan Times (English)	COVID-19 website
2020-06-05	Himalayan Times (English)	COVID-19: The gender conundrum
2020-05-25	Himalayan Times (English)	COVID-infected person flees from hospital
2020-06-23	Himalayan Times (English)	Covira app launched to measure COVID risk
2020-04-18	Himalayan Times (English)	Crisis management centre to be set up
2020-06-26	Himalayan Times (English)	Crores mobilised to combat COVID
2020-04-10	Kantipur (Nepali)	अग्रपंक्तिका स्‍ वास्थ्यकर्मी र प्रहरीकै तलब कट्टा
2020-06-26	Online Khabar (Nepali)	Cyber crime has increased in the lockdown
2020-04-06	Kantipur (Nepali)	मूल सडकमा कडाइ, भित्र भीडभाड
2020-04-08	Kantipur (Nepali)	मेसिनले नधान्दा काठमाडौं पठाइयो स्वाब
2020-06-08	Himalayan Times (English)	Dailekh becoming COVID hotspot in Karnali
2020-03-29	Himalayan Times (English)	Daily wage earners in Tanahun face starvation

Date of publication (yyyy/mm/dd)	Name of news media (select from drop-down menu)	Title of news/headline
2020-03-27	Himalayan Times (English)	Daily wagers bear the brunt of lockdown, govt fails to provide reprieve
2020-06-15	Himalayan Times (English)	DAO puts more restrictions on entry into valley
2020-05-16	Himalayan Times (English)	DAO seals Katuwalpauwa amid virus scare
2020-08-08	Himalayan Times (English)	DAOs agree on 16-day restrictions in valley
2020-03-06	Himalayan Times (English)	DAOs urge people to avoid gatherings
2020-02-04	Himalayan Times (English)	DDA warns black marketeers
2020-06-25	Himalayan Times (English)	Dealing with summer heat amid COVID-19 pandemic
2020-07-31	Himalayan Times (English)	Death in quarantine
2020-06-01	Himalayan Times (English)	Death toll of Nepalis abroad rises to 121
2020-05-01	Kantipur (Nepali)	Debate in Management of Corona Hospital
2020-06-04	Himalayan Times (English)	Decision protested
2020-07-15	Himalayan Times (English)	Decision to ramp up tests in Sudurpaschim yet to be implemented
2020-05-19	Himalayan Times (English)	Decision to turn Kalaiya Hospital into COVID treatment facility draws flak
2020-07-01	Kantipur (Nepali)	Demand For Introducing Relief Program
2020-05-05	Himalayan Times (English)	Dengue cases up in Gandaki Province
2020-05-27	Himalayan Times (English)	Deuba renews demand for relief package to journos and media houses
2020-03-28	Himalayan Times (English)	Deuba urges govt to act
2020-04-19	Kantipur (Nepali)	मोहनापारि दशगजाभित्रै 'सिल
2020-08-13	Himalayan Times (English)	Dhading DAO issues prohibitory order
2020-03-17	Himalayan Times (English)	Dhangadi sub-metropolis conducting health check-up at border point
2020-08-07	Himalayan Times (English)	Dhangadi sub-metropolis sets up isolation ward
2020-08-12	Himalayan Times (English)	Dharan sub-metropolis sets up isolation ward
2020-08-03	Himalayan Times (English)	Dharan to conduct mass PCR tests
2020-03-22	Himalayan Times (English)	Dial 1180 in case of COVID-19 suspicion: KMC
2020-04-22	Himalayan Times (English)	DIGs told to strictly enforce lockdown

Date of publication (yyyy/mm/dd)	Name of news media (select from drop-down menu)	Title of news/headline
2020-01-23	Himalayan Times (English)	Dire straits
2020-06-13	Himalayan Times (English)	Directive on RDT creates panic in Sudurpaschim
2020-01-30	Online Khabar (Nepali)	Discussions are underway to rescue Nepali students from China: Ministry of Health
2020-05-05	Himalayan Times (English)	Disinfectant tunnel
2020-04-30	Himalayan Times (English)	Disinfection tunnel to be inaugurated today
2020-06-25	Online Khabar (Nepali)	Disruption in management of corpses in Pokhara again
2020-02-02	Himalayan Times (English)	Disseminate information on coronavirus in tourist areas: Stakeholders
2020-03-31	Himalayan Times (English)	Distance maintained
2020-08-25	Himalayan Times (English)	Docs advocate plasma therapy for critically ill COVID patients
2020-03-24	Himalayan Times (English)	Docs favour treatment only in designated hospitals
2020-03-21	Himalayan Times (English)	Docs seek more protective equipment
2020-03-07	Himalayan Times (English)	Doctors at BPKIHS accused of ditching patient amidst coronavirus scare
2020-07-03	Himalayan Times (English)	DoFE starts issuing re-entry permits
2020-06-16	Himalayan Times (English)	Don’t be careless, keep following safety measures
2020-03-13	Himalayan Times (English)	Drastic steps planned to prevent outbreak
2020-06-24	Himalayan Times (English)	Drive to end stigma, discrimination against coronavirus patients launched
2020-03-20	Himalayan Times (English)	DRRMA dwells on preparedness, response plan for COVID-19
2020-06-08	Himalayan Times (English)	Duty comes first for these Koshi lab heroes
2020-07-26	Himalayan Times (English)	Ease restrictions on domestic flights: Airline operators
2020-06-21	Himalayan Times (English)	E-conference on COVID-19 held in Karnali Province
2020-01-20	Himalayan Times (English)	EDCD to display banners in Mandarin
2020-03-27	Kantipur (Nepali)	भारतबाट आएकालाई क्वारन्टाइनमा रोख्नू
2020-04-20	Kantipur (Nepali)	भोकै परका ५ सय मजदुरले मागे राहत
2020-04-25	Himalayan Times (English)	Efficacy of rapid corona tests questioned as 16 test positive after negative RDT
2020-05-05	Himalayan Times (English)	Eight injured in dispute over relief distribution

Date of publication (yyyy/mm/dd)	Name of news media (select from drop-down menu)	Title of news/headline
2020-08-28	Himalayan Times (English)	Eight more die
2020-03-27	Himalayan Times (English)	Eight suspected COVID-19 patients in isolation wards
2020-08-04	Himalayan Times (English)	Elderly COVID patient dies
2020-06-09	Himalayan Times (English)	Elderly man succumbs to virus
2020-08-05	Himalayan Times (English)	Elderly succumbs to contagion
2020-08-27	Himalayan Times (English)	Eleven more succumb to coronavirus
2020-06-11	Himalayan Times (English)	Emergency ward of province hospital closed
2020-06-12	Himalayan Times (English)	Enough is enough! How it all started and where is it headed
2020-03-07	Himalayan Times (English)	Enough stock of essentials: Govt
2020-06-17	Himalayan Times (English)	Ensuring safety and livelihood of waste workers
2020-03-05	Online Khabar (Nepali)	Entering Nepal saying 'Delhi riots bigger than Corona'!
2020-06-04	Himalayan Times (English)	Entry points closed
2020-05-08	Himalayan Times (English)	Entry points sealed
2020-04-10	Kantipur (Nepali)	भएकै प्रयोगशाला उपयोग गरिएन
2020-07-01	Kantipur (Nepali)	Equipment were halted in China But Nepal keeps Purchasing Them in the Country
2020-07-03	Himalayan Times (English)	EU, UNICEF to prevent malnutrition in Nepal
2020-02-05	Himalayan Times (English)	Evacuees could be quarantined in Bhaktapur
2020-02-29	Himalayan Times (English)	Evacuees to return home on 17th day
2020-05-01	Kantipur (Nepali)	Exams after Three Weeks of Lockdown End
2020-08-24	Himalayan Times (English)	Expand PCR tests across country: Deuba
2020-05-01	Himalayan Times (English)	Expecting mother expelled from operation theatre
2020-01-29	Himalayan Times (English)	Face masks rerouted to China, shortage hits market
2020-03-17	Himalayan Times (English)	Face masks, sanitisers crunch in Nepalgunj
2020-07-03	Himalayan Times (English)	Family of deceased in dire straits
2020-04-18	Himalayan Times (English)	Federal govt starts sending doctors to provinces, 34 docs reach Dhangadi

Date of publication (yyyy/mm/dd)	Name of news media (select from drop-down menu)	Title of news/headline
2020-03-29	Himalayan Times (English)	Fifth COVID-19 case confirmed in teenager from Baglung
2020-06-02	Himalayan Times (English)	FinMin Khatiwada defends budget for FY 2020-21
2020-06-10	Himalayan Times (English)	First COVID death in Chitwan
2020-05-17	Himalayan Times (English)	First COVID-19 death in Nepal
2020-04-05	Himalayan Times (English)	First local transmission of COVID-19
2020-05-11	Himalayan Times (English)	Fiscal budget to prioritise revitalising crisis-hit economy
2020-08-07	Himalayan Times (English)	Five deaths in a day
2020-03-07	Himalayan Times (English)	Five hospitalised for COVID-19
2020-06-08	Himalayan Times (English)	Five infected persons isolated in Dadeldhura
2020-04-21	Himalayan Times (English)	Five involved in distribution of relief held
2020-08-18	Himalayan Times (English)	Five local levels sealed in Tanahun
2020-08-08	Himalayan Times (English)	Five more fatalities
2020-05-06	Himalayan Times (English)	Five quarantined
2020-04-08	Himalayan Times (English)	Flight suspension extended till April 30
2020-05-21	Himalayan Times (English)	Flights to remain suspended till June 14
2020-08-11	Himalayan Times (English)	Flights to remain suspended till September 1
2020-06-01	Himalayan Times (English)	FNCCI calls for lockdown extension review
2020-04-26	Himalayan Times (English)	Food distributed
2020-06-19	Himalayan Times (English)	Food insecurity increases by 23 per cent in Nepal
2020-04-02	Himalayan Times (English)	Foreign returnees facing humiliation
2020-06-14	Himalayan Times (English)	Foreign returnees to be quarantined in Dhulikhel
2020-03-20	Himalayan Times (English)	Foreigners not complying with mandatory self-quarantine measures
2020-05-02	Himalayan Times (English)	Four areas sealed in Nepalgunj

Date of publication (yyyy/mm/dd)	Name of news media (select from drop-down menu)	Title of news/headline
2020-06-03	Himalayan Times (English)	Four health workers test positive in valley, 288 COVID cases in a day, Nepal count tops 2,000
2020-08-15	Himalayan Times (English)	Four more deaths
2020-08-11	Himalayan Times (English)	Four more succumb to contagion
2020-08-12	Himalayan Times (English)	Four more succumb to COVID-19
2020-08-14	Himalayan Times (English)	Four new coronavirus fatalities
2020-08-27	Himalayan Times (English)	Four succumb to virus in 24 hours in Birgunj
2020-03-02	Himalayan Times (English)	Four suspected COVID-19 patients in hospital
2020-07-26	Himalayan Times (English)	Fourteen health workers quarantined in Rajbiraj
2020-07-01	Himalayan Times (English)	Fourteen new COVID patients diagnosed in Kathmandu valley
2020-07-20	Himalayan Times (English)	Free COVID-19 test for cancer, heart patients
2020-06-04	Himalayan Times (English)	Frontline workers to get hazard allowance
2020-04-09	Himalayan Times (English)	Funeral workers demand PPE
2020-04-12	Kantipur (Nepali)	नेपाल छिर्न अनेक जुक्ति
2020-04-19	Kantipur (Nepali)	नेपाली अर्थतन्त्रले कति गुमाउला
2020-08-05	Himalayan Times (English)	Gai Jatra celebrated amid COVID pandemic
2020-07-02	Himalayan Times (English)	Gandaki COVID cases near one thousand
2020-07-03	Himalayan Times (English)	Gandaki PA panel president donates perks to corona fund
2020-06-11	Himalayan Times (English)	Gandaki Province collecting details at entry point
2020-06-14	Himalayan Times (English)	Gandaki province local levels to procure PCR machines
2020-03-24	Himalayan Times (English)	Gandaki Province shuts vehicular movement
2020-04-06	Kantipur (Nepali)	नारायणीमा २० आइसोलेसन शय्या थप
2020-04-06	Himalayan Times (English)	Give priority to needy people in relief distribution: MoFAGA
2020-03-25	Himalayan Times (English)	Gokarneshwor Municipality to set up 27-bed centre for coronavirus patients
2020-03-18	Himalayan Times (English)	Good samaritans willing to turn houses into quarantine facilities
2020-04-12	Himalayan Times (English)	Government issues COVID-19 fund directive

Date of publication (yyyy/mm/dd)	Name of news media (select from drop-down menu)	Title of news/headline
2020-06-16	Himalayan Times (English)	Government offices resume service in two shifts
2020-06-09	Himalayan Times (English)	Government response to Covid crisis abysmal: NC
2020-08-08	Himalayan Times (English)	Government rules out complete lockdown
2020-05-09	Himalayan Times (English)	Government set to begin pool testing to trace novel coronavirus infection
2020-03-12	Himalayan Times (English)	Government still not prepared to fight coronavirus outbreak
2020-03-19	Himalayan Times (English)	Government takes drastic steps to counter novel coronavirus threat
2020-02-02	Himalayan Times (English)	Government to evacuate Nepalis from Hubei
2020-04-26	Himalayan Times (English)	Government urged to adopt reliable testing method
2020-04-04	Himalayan Times (English)	Government urged to protect migrant workers, marginalised groups
2020-02-03	Himalayan Times (English)	Government urged to take precautionary measures against coronavirus
2020-05-02	Himalayan Times (English)	Government's stimulus packages inadequate, laments private sector
2020-04-01	Himalayan Times (English)	Govt adopts three-pronged approach to fight COVID-19
2020-02-13	Himalayan Times (English)	Govt allays fear of coronavirus in Sindhupalchowk
2020-06-15	Himalayan Times (English)	Govt asked to strengthen law and order
2020-04-01	Himalayan Times (English)	Govt cancels Visit Nepal 2020 campaign
2020-04-04	Himalayan Times (English)	Govt decision to outsource PPE procurement contract to NA draws flak
2020-06-07	Himalayan Times (English)	Govt directed to ensure regular medical services to citizen
2020-05-15	Himalayan Times (English)	Govt does away with issuing vehicle passes
2020-03-25	Himalayan Times (English)	Govt doing its bit to prevent COVID-19 outbreak in prisons
2020-06-12	Himalayan Times (English)	Govt eases lockdown
2020-05-31	Himalayan Times (English)	Govt extends lockdown till June 14
2020-03-14	Himalayan Times (English)	Govt fails to deliver right information at right time
2020-02-24	Himalayan Times (English)	Govt halts Nepali workers from going to South Korea

Date of publication (yyyy/mm/dd)	Name of news media (select from drop-down menu)	Title of news/headline
2020-04-11	Himalayan Times (English)	Govt hospitals in Bara turning patients away
2020-06-20	Himalayan Times (English)	Govt issues COVID safety guidelines
2020-05-22	Himalayan Times (English)	Govt issues COVID-19 Unified Hospital Operation Order
2020-06-06	Himalayan Times (English)	Govt issues new guidelines for COVID testing
2020-05-01	Himalayan Times (English)	Govt launches relief package targeting workers
2020-06-23	Himalayan Times (English)	Govt managing ventilators, ICUs by estimating corona cases
2020-06-04	Himalayan Times (English)	Govt may declare state of public health emergency
2020-05-02	Himalayan Times (English)	Govt plans to ease lockdown, restart economy
2020-05-05	Himalayan Times (English)	Govt plans to evacuate over 35,000 illegal migrants
2020-05-27	Himalayan Times (English)	Govt projects 2.3 per cent economic growth for this fiscal
2020-07-18	Himalayan Times (English)	Govt rules out COVID community spread in valley
2020-03-23	Himalayan Times (English)	Govt seals border, creates Rs 500 million fund
2020-05-23	Himalayan Times (English)	Govt still unprepared to bring back Nepalis
2020-04-05	Himalayan Times (English)	Govt to conduct rapid testing in three districts
2020-07-21	Himalayan Times (English)	Govt to conduct sero-survey of COVID community spread
2020-06-15	Himalayan Times (English)	Govt to let pvt labs conduct PCR tests
2020-04-06	Himalayan Times (English)	Govt to mobilise 50,000 health volunteers
2020-05-20	Himalayan Times (English)	Govt to rescue Nepalis stranded abroad ‘on priority basis’
2020-03-27	Himalayan Times (English)	Govt told to ensure smooth supply of daily essentials
2020-03-03	Himalayan Times (English)	Govt told to halt flights to high-risk countries
2020-03-12	Himalayan Times (English)	Govt urged to act on war footing
2020-03-16	Himalayan Times (English)	Govt urged to declare ‘economic emergency’
2020-03-19	Himalayan Times (English)	Govt urged to ensure supply of daily essentials

Date of publication (yyyy/mm/dd)	Name of news media (select from drop-down menu)	Title of news/headline
2020-07-06	Himalayan Times (English)	Govt urged to guarantee citizens' right to health
2020-03-20	Himalayan Times (English)	Govt's measures to fight COVID-19 inadequate, say MPs
2020-06-05	Himalayan Times (English)	GPKTC turned into Corona hospital
2020-04-27	Himalayan Times (English)	Grant of Rs 30 million
2020-03-25	Himalayan Times (English)	Grocery stores can operate during week-long shutdown, reiterates govt
2020-03-27	Kantipur (Nepali)	नगर जुटे आइसोलेसन कक्षमा
2020-05-22	Himalayan Times (English)	Gulmi teacher succumbs to coronavirus
2020-04-15	Kantipur (Nepali)	जसले त्रासका बेला आशा जगाए ७५३ स्थानीय तहका जनप्रतिनिधिलाई यतिबेला धपेडी छ। कोभिड-१९ को त्रास भएसागै जनचेतनामूलक कार्यक्रम सुरू गरेका उनीहरुले अहिले आफ्नै बजे
2020-08-10	Himalayan Times (English)	Handigaon sealed in Kathmandu
2020-01-28	Kantipur (Nepali)	Has not Spread From Infected
2020-06-16	Himalayan Times (English)	Health centre sealed
2020-03-06	Himalayan Times (English)	Health check at airport in Bhadrapur
2020-03-18	Himalayan Times (English)	Health desk established at Pokhara Lakeside
2020-03-16	Himalayan Times (English)	Health desk on border with Sikkim
2020-02-11	Himalayan Times (English)	Health desk operated
2020-02-04	Himalayan Times (English)	Health desk set up
2020-01-31	Himalayan Times (English)	Health desk set up at Tatopani checkpoint
2020-03-06	Himalayan Times (English)	Health desk set up at Nepalgunj airport
2020-02-01	Himalayan Times (English)	Health desk set up at Pokhara airport
2020-03-05	Himalayan Times (English)	Health desk set up at Rupaidiha border
2020-01-30	Himalayan Times (English)	Health desks set up for coronavirus test
2020-03-11	Himalayan Times (English)	Health desks to be set up in Mustang
2020-04-19	Himalayan Times (English)	Health experts for more polymerase chain reaction tests

Date of publication (yyyy/mm/dd)	Name of news media (select from drop-down menu)	Title of news/headline
2020-04-13	Himalayan Times (English)	Health experts in Panchthar start COVID-19 sample tests
2020-07-02	Himalayan Times (English)	Health materials distributed among youths
2020-06-06	Himalayan Times (English)	Health ministry pitches for declaring health emergency
2020-01-20	Himalayan Times (English)	Health system needs to be restructured
2020-01-20	Himalayan Times (English)	Health system needs to be restructured, Page 5
2020-03-16	Himalayan Times (English)	Health workers asked not to stay on leave
2020-04-30	Himalayan Times (English)	Health workers at Koshi Hospital fighting coronavirus undeterred, undaunted’
2020-03-30	Himalayan Times (English)	Health workers attending COVID-19 patient to undergo test
2020-03-18	Himalayan Times (English)	Health workers in Bhojpur district directed not to go on leave
2020-06-16	Himalayan Times (English)	Help sought for lab
2020-06-04	Himalayan Times (English)	Hepatitis patient being treated in quarantine facility
2020-04-04	Kantipur (Nepali)	जनताको जीवनलाई सबैभन्दा माथि राख
2020-01-31	Online Khabar (Nepali)	High level monitoring committee formed for corona virus prevention
2020-06-13	Himalayan Times (English)	High-level COVID control panel disbanded
2020-04-11	Himalayan Times (English)	High-level panel formed to monitor rights situation
2020-06-03	Himalayan Times (English)	High-level panel to decide on easing lockdown by tomorrow, Page 1
2020-04-04	Kantipur (Nepali)	ज्वरोका बिरामी जाँच्दैनन् अस्पताल
2020-04-20	Kantipur (Nepali)	जटिल बन्दै कन्टचाक्ट ट्रेसिङ
2020-03-24	Kantipur (Nepali)	जति धेरै परीक्षण, उति जोखिम कम
2020-05-25	Himalayan Times (English)	Holding areas set up
2020-07-04	Himalayan Times (English)	Holding centre in Khotang
2020-07-25	Himalayan Times (English)	Holding, isolation centre for women established
2020-03-09	Himalayan Times (English)	Holi being celebrated amid COVID-19 scare
2020-06-04	Himalayan Times (English)	Home isolation for asymptomatic, mild Covid cases
2020-06-30	Kantipur (Nepali)	Home Ministry is not Supportive to Nepali Army for Briging Health Equipment with Financial Support From China

Date of publication (yyyy/mm/dd)	Name of news media (select from drop-down menu)	Title of news/headline
2020-05-22	Himalayan Times (English)	Hospital provides delivery service amid COVID fear
2020-06-15	Himalayan Times (English)	Hospital sealed
2020-08-08	Himalayan Times (English)	Hospital shuts down services
2020-02-27	Himalayan Times (English)	Hospitals ill-equipped to handle COVID-19 patients
2020-05-17	Himalayan Times (English)	Hospitals in Karnali to get medical equipment worth Rs 10mn
2020-03-08	Himalayan Times (English)	Hospitals outside Kathmandu ill-equipped to tackle COVID-19
2020-04-07	Himalayan Times (English)	Hospitals outside Kathmandu ill-prepared to fight coronavirus outbreak
2020-03-29	Himalayan Times (English)	Hospitals outside valley lack PPE, infrastructure
2020-08-13	Himalayan Times (English)	Hospitals told to test health workers' swab samples for COVID
2020-05-14	Himalayan Times (English)	Hospitals treating COVID face shortage of beds
2020-05-02	Himalayan Times (English)	Hospitals turn away patients with fever
2020-03-22	Himalayan Times (English)	Hospitals waiting for reagent to test COVID-19
2020-07-09	Himalayan Times (English)	Hospitals warned against charging for tests
2020-08-23	Himalayan Times (English)	Hotel quarantine not for profit, but govt trying to haggle: HAN
2020-06-04	Himalayan Times (English)	Hotels in five locations selected for quarantine
2020-07-27	Himalayan Times (English)	Hotels in Sauraha ready to welcome guests
2020-05-24	Himalayan Times (English)	Hotels to provide rooms to quarantine healthcare workers
2020-07-31	Himalayan Times (English)	Hotels, restaurants start resuming services
2020-03-18	Himalayan Times (English)	Hotline service
2020-06-23	Himalayan Times (English)	House panel points out discrepancies in purchase of medical supplies
2020-08-07	Himalayan Times (English)	House sealed after coronavirus claims man
2020-06-08	Himalayan Times (English)	How a PCR is done
2020-05-02	Kantipur (Nepali)	How COVID-19 Infection Spreads
2020-06-30	Kantipur (Nepali)	How to Move in Post-COVID-19 Era?

Date of publication (yyyy/mm/dd)	Name of news media (select from drop-down menu)	Title of news/headline
2020-05-01	Himalayan Times (English)	Human rights monitoring panel formed in all districts
2020-04-28	Himalayan Times (English)	Hundreds of Kathmandu returnees stranded at Thankot check post
2020-06-12	Himalayan Times (English)	Hundreds protest against govt’s ‘incompetence’
2020-06-27	Himalayan Times (English)	Hunger strike against government inefficiency, Page 2
2020-07-02	Himalayan Times (English)	Hunger strike by youth activists enters sixth day
2020-04-07	Kantipur (Nepali)	जहाँ आफन्तलाई पनि प्रवेश निषेध छ
2020-04-17	Kantipur (Nepali)	जहाको सास्ती सरकारले देख्दैन
2020-03-11	Online Khabar (Nepali)	If necessary, the government can rescue from any country: Shrestha
2020-01-31	Online Khabar (Nepali)	If there is a risk of corona, we will bring back Nepali students from China : Minister Dhakal
2020-04-03	Himalayan Times (English)	Imadol locals protest against Indonesian Islamic leaders sheltering in mosque
2020-03-13	Online Khabar (Nepali)	In print today: How reliable is the corona test in Nepal?
2020-01-27	Kantipur (Nepali)	Increase Attention
2020-05-18	Himalayan Times (English)	India gifts 30,000 PCR testing kits to Nepal
2020-03-16	Himalayan Times (English)	India offers $10 million to fight COVID-19
2020-04-23	Himalayan Times (English)	India provides 23 tonnes of medicines to Nepal
2020-06-07	Himalayan Times (English)	India returnees brought to Panchthar sans PCR tests
2020-04-03	Himalayan Times (English)	Industries allowed to resume operations with residential workers
2020-06-02	Himalayan Times (English)	Infected, non-infected sharing same toilet in Bajura’s isolation ward, Page 2
2020-07-10	Himalayan Times (English)	Infection rate high among returnee migrants
2020-08-12	Himalayan Times (English)	Informal, salaried workers hit hard by COVID-19 in Kathmandu valley: Study
2020-06-29	Online Khabar (Nepali)	Instructions to strictly enforce the loosened lockdown criteria
2020-06-14	Himalayan Times (English)	Insurance firms start paying COVID claims
2020-06-13	Himalayan Times (English)	Isolation building
2020-08-07	Himalayan Times (English)	Isolation centre vandalised over food quality
2020-08-31	Himalayan Times (English)	Isolation facility set up in Dhankuta Municipality
2020-03-19	Himalayan Times (English)	Isolation ward being set up in Lamjung hospital

Date of publication (yyyy/mm/dd)	Name of news media (select from drop-down menu)	Title of news/headline
2020-06-26	Himalayan Times (English)	Isolation ward established in Dhangadi
2020-04-08	Himalayan Times (English)	Isolation ward in Bajura without docs, equipment
2020-08-10	Himalayan Times (English)	Isolation ward set up
2020-07-08	Himalayan Times (English)	Isolation wards empty as COVID-19 cases stay put in quarantine
2020-06-28	Himalayan Times (English)	Isolation wards packed
2020-03-15	Himalayan Times (English)	Isolation wards set up in Ramechhap district
2020-07-30	Himalayan Times (English)	Israel gifts medical supplies to Province 2
2020-02-03	Online Khabar (Nepali)	It takes at least 2 days to bring back Nepali from China: MoHP
2020-06-22	Himalayan Times (English)	It’s okay to be worried, but don’t be too worried
2020-03-24	Kantipur (Nepali)	वृद्धवृद्धालाई घरभित्रै राख्नुस्
2020-03-28	Himalayan Times (English)	Janakpur Provincial Hospital declines to treat woman suffering from high fever
2020-07-16	Himalayan Times (English)	Jhapa at high risk of COVID-19 spread
2020-06-28	Online Khabar (Nepali)	Just spoke but PCR machine did not reach Doti
2020-04-09	Kantipur (Nepali)	परीक्षण सातै प्रदेशमा तर सुस्त
2020-04-15	Kantipur (Nepali)	परीक्षणबिनै क्वारेन्टाइनबाट घर
2020-03-26	Kantipur (Nepali)	प्रदेशहरूमा क्वारन्टाइन र आइसोलेसनको तयारी
2020-04-20	Himalayan Times (English)	Kailali COVID patient recovers, discharged
2020-04-22	Himalayan Times (English)	Kailali youth recovers, discharged
2020-03-23	Himalayan Times (English)	Kanchanpur, Nepalgunj under indefinite lockdown
2020-05-12	Himalayan Times (English)	Kapilvastu sealed for a week
2020-05-05	Himalayan Times (English)	Karnali Province to be sealed for a week
2020-03-08	Himalayan Times (English)	Karnali to take precautions against virus infection
2020-08-24	Himalayan Times (English)	Kaski NC for continuing prohibitory order
2020-05-01	Kantipur (Nepali)	Kathmandu is Becoming Insecure
2020-04-03	Himalayan Times (English)	Kathmandu patients still to recover
2020-06-17	Himalayan Times (English)	Kathmanduite among 380 fresh COVID cases
2020-08-27	Himalayan Times (English)	Kavre local levels reluctant to set up isolation wards

Date of publication (yyyy/mm/dd)	Name of news media (select from drop-down menu)	Title of news/headline
2020-05-23	Himalayan Times (English)	Kavre quarantines almost vacant
2020-08-14	Himalayan Times (English)	Kawasoti Municipality sealed for nine days
2020-04-08	Kantipur (Nepali)	पाहुना राखे ५० हजारजरिवाना
2020-02-10	Online Khabar (Nepali)	Kharipati quarantine
2020-04-16	Kantipur (Nepali)	पीसीआरको दायरा घट्द
2020-03-27	Kantipur (Nepali)	पीपीई बनाउँदै पाटन अस्पताल
2020-04-06	Kantipur (Nepali)	पीपीई भन्दै ॅगाउन’ पठाइया
2020-03-26	Kantipur (Nepali)	पीपीईको सट्टा रनकोॅट
2020-06-04	Himalayan Times (English)	KMC Hospital sealed
2020-08-01	Himalayan Times (English)	KMC set to introduce local curriculum at schools
2020-06-06	Himalayan Times (English)	KMC set to launch ‘Cash for Work’ scheme
2020-03-30	Himalayan Times (English)	KMC to distribute relief through one-door system
2020-03-28	Himalayan Times (English)	KMC to provide relief package to homeless, daily wage earners
2020-06-19	Himalayan Times (English)	KMC to provide relief to COVID-19-hit businesses, public
2020-08-01	Himalayan Times (English)	KMC urges people to avoid large gatherings
2020-06-25	Himalayan Times (English)	KMC waives tax on various sectors
2020-04-05	Kantipur (Nepali)	फर्काउने अस्पताललाई कारबाही गर्न निर्देशन
2020-02-25	Himalayan Times (English)	Korea-returnees visit hospital
2020-04-16	Kantipur (Nepali)	सिमानामा थपिए प्रहरी र सशस्त्रका फौज
2020-04-13	Himalayan Times (English)	Lab tests conducted
2020-07-01	Kantipur (Nepali)	Labrotary Added For Testing COVID-19
2020-03-19	Himalayan Times (English)	Lack of kits at Nepal’s only testing centre hampering COVID-19 fight
2020-04-18	Himalayan Times (English)	Lack of kits slows down coronavirus testing in Sudurpaschim
2020-05-01	Kantipur (Nepali)	Land Should Not be Kept Barren
2020-03-22	Himalayan Times (English)	Large number of people leaving valley amid virus scare
2020-05-08	Himalayan Times (English)	Lawmakers tested for COVID, result today
2020-04-13	Kantipur (Nepali)	दिल्ली र गोन्जाउबाट स् वास्थ्य सामग्री ल्याइँद
2020-03-13	Online Khabar (Nepali)	Let's go back to the village, let's give priority to agriculture
2020-03-24	Himalayan Times (English)	Little done at Western Regional Hospital

Date of publication (yyyy/mm/dd)	Name of news media (select from drop-down menu)	Title of news/headline
2020-05-01	Kantipur (Nepali)	Livelihood Should not be Uneased
2020-04-07	Kantipur (Nepali)	विदेशबाट आएका सबैलाई क्वारन्टाइनमा राख्नू : सर्वोच्च
2020-04-07	Kantipur (Nepali)	विपन्नमाथि नै बढी मार
2020-03-11	Himalayan Times (English)	Local bourse plunges 6.02pc on virus worries
2020-06-22	Online Khabar (Nepali)	Local government priorities in the Corona epidemic
2020-08-30	Himalayan Times (English)	Local level buys VTM at its own expense
2020-08-09	Himalayan Times (English)	Local level in Jhapa imposes lockdown
2020-05-13	Himalayan Times (English)	Local level in Tanahun to provide textbooks at students' doorsteps
2020-04-01	Himalayan Times (English)	Local levels collecting details of returnees
2020-03-29	Himalayan Times (English)	Local levels in Lamjung to set up quarantine facilities
2020-07-20	Himalayan Times (English)	Local levels to prepare list of unemployed people
2020-04-09	Himalayan Times (English)	Local levels told to ensure basic amenities in quarantine centres
2020-07-19	Himalayan Times (English)	Local levels yet to expand quarantine facilities
2020-05-21	Himalayan Times (English)	Locals capture quarantine centre in Banke
2020-03-07	Himalayan Times (English)	Locals told to take precautions against spread of coronavirus
2020-08-10	Himalayan Times (English)	Locals, kin of deceased vandalise Narayani Hospital
2020-05-29	Himalayan Times (English)	Locals, police clash over quarantine in Rautahat
2020-08-04	Himalayan Times (English)	Lockdown at religious sites affects Rakshya Bandhan celebrations
2020-06-08	Himalayan Times (English)	Lockdown defiance up in valley
2020-05-10	Himalayan Times (English)	Lockdown eased in Kavre
2020-05-12	Himalayan Times (English)	Lockdown eased in Udayapur
2020-08-13	Himalayan Times (English)	Lockdown extended
2020-08-24	Himalayan Times (English)	Lockdown extended by a week in Sindhupalchowk
2020-05-18	Himalayan Times (English)	Lockdown extended by another 15 days
2020-04-15	Himalayan Times (English)	Lockdown extended till April 27 - Flights suspended, borders sealed till April 30

Date of publication (yyyy/mm/dd)	Name of news media (select from drop-down menu)	Title of news/headline
2020-03-30	Himalayan Times (English)	Lockdown extended till April 7 midnight
2020-05-04	Himalayan Times (English)	Lockdown hits immunisation drive against diarrhoea
2020-08-06	Himalayan Times (English)	Lockdown imposed in Dharan, Kailari
2020-08-14	Himalayan Times (English)	Lockdown in Butwal
2020-07-22	Himalayan Times (English)	Lockdown lifted, some restrictions remain
2020-05-06	Himalayan Times (English)	Lockdown likely to be extended, decision today
2020-04-26	Himalayan Times (English)	Lockdown modality will be changed: CM Gurung
2020-04-17	Himalayan Times (English)	Lockdown tightened in Dhading district
2020-07-05	Himalayan Times (English)	Lockdown tightened in Lamjung
2020-03-26	Kantipur (Nepali)	चिनियाँ कम्पनीद्वारा २ हजार किट सहयोग
2020-04-11	Kantipur (Nepali)	चितवनबाट हिडे रै कोहलपुर
2020-04-10	Kantipur (Nepali)	हिंसापीडितलाई झन् पीडा
2020-04-10	Kantipur (Nepali)	हिडेरै १६३ किलोमिटर
2020-04-26	Himalayan Times (English)	Malaria seen in people staying in quarantine
2020-03-28	Himalayan Times (English)	Man from Kailali tests positive for COVID-19
2020-06-26	Himalayan Times (English)	Man from Lalitpur among two corona fatalities
2020-05-29	Himalayan Times (English)	Man from Lalitpur fifth to succumb to COVID in Nepal
2020-03-26	Himalayan Times (English)	Man from UAE third Nepali with COVID
2020-05-13	Himalayan Times (English)	Man jumps off roof to avoid quarantine
2020-06-24	Himalayan Times (English)	Manage COVID crisis effectively: Lawmakers
2020-07-30	Himalayan Times (English)	Masks distributed
2020-07-28	Himalayan Times (English)	Mass testing begins in Jhapa’s Mechinagar
2020-08-26	Himalayan Times (English)	Maternity Hospital to get 200 isolation beds
2020-02-16	Online Khabar (Nepali)	Mayor of Changunarayan demands immediate health desk for locals
2020-06-06	Himalayan Times (English)	Mayor quarantined

Date of publication (yyyy/mm/dd)	Name of news media (select from drop-down menu)	Title of news/headline
2020-06-24	Himalayan Times (English)	Mayor reaches Jumla with swabs for PCR test
2020-07-14	Himalayan Times (English)	Mayur company resumes bus service in Kathmandu
2020-06-23	Himalayan Times (English)	Meals provided for COVID infected
2020-08-14	Himalayan Times (English)	Mechi AMDA Hospital sealed
2020-06-17	Himalayan Times (English)	Medical equipment
2020-03-26	Himalayan Times (English)	Medical help thru mobile app
2020-06-09	Himalayan Times (English)	Medicine sales down amid COVID-19 pandemic
2020-04-12	Himalayan Times (English)	Mental effects of lockdown on children a matter of growing concern among parents
2020-03-21	Himalayan Times (English)	Minister Bhattarai pledges to improve sanitation at airports
2020-06-20	Himalayan Times (English)	Minister Gyawali calls for concerted effort to fight against COVID-19 pandemic
2020-06-09	Himalayan Times (English)	MoFA submits list of citizens needing urgent evacuation
2020-06-22	Himalayan Times (English)	MoFAGA fiat to facilitate implementation of CCMC decisions
2020-05-07	Himalayan Times (English)	MoHP gets medical assistance worth 45.3 million rupees
2020-04-03	Himalayan Times (English)	MoHP issues notice
2020-04-28	Himalayan Times (English)	Monitoring under way
2020-07-04	Himalayan Times (English)	More COVID patients discharged than diagnosed in a day
2020-05-01	Kantipur (Nepali)	More Discussion is Needed in Education Sector
2020-07-05	Himalayan Times (English)	More patients recovering from COVID-19 in Karnali
2020-06-12	Himalayan Times (English)	More PCR machines
2020-05-27	Himalayan Times (English)	More PCR tests necessary for accurate diagnosis
2020-03-20	Himalayan Times (English)	More people leaving Kathmandu valley
2020-03-31	Himalayan Times (English)	More than 2,000 lockdown violators punished
2020-08-26	Himalayan Times (English)	More than 4,000 COVID patients recover in Sudurpaschim
2020-07-28	Himalayan Times (English)	More women journalists losing their jobs due to coronavirus pandemic

Date of publication (yyyy/mm/dd)	Name of news media (select from drop-down menu)	Title of news/headline
2020-04-28	Himalayan Times (English)	Most Bhojpur quarantine centres do not meet government-set criteria
2020-03-23	Himalayan Times (English)	Most border points closed in Province 2
2020-04-29	Himalayan Times (English)	Most quarantine facilities in Bajura ill-managed
2020-04-19	Himalayan Times (English)	Move to set up quarantine in Tribhuvan Park opposed
2020-06-11	Himalayan Times (English)	MoWCSC seeks people's access to regular health services
2020-02-05	Himalayan Times (English)	MPs flay laxity to evacuate Nepalis from China
2020-03-08	Himalayan Times (English)	Mugu to set up health desks on border
2020-06-21	Himalayan Times (English)	Municipal area sealed
2020-05-15	Himalayan Times (English)	Municipality sealed
2020-06-23	Himalayan Times (English)	Municipality sealed over virus fears
2020-03-19	Himalayan Times (English)	NA conducts rehearsal to respond to COVID-19
2020-03-22	Himalayan Times (English)	NA personnel deployed at Jamunaha entry point
2020-03-17	Himalayan Times (English)	NA sets up makeshift tents for quarantine
2020-05-10	Himalayan Times (English)	Narayani Hospital to resume general services soon
2020-04-20	Himalayan Times (English)	Narayani Hospital yet to start PCR testing
2020-02-17	Himalayan Times (English)	Nasal, throat swabs collected from 175 evacuees who finally arrive from China
2020-04-07	Himalayan Times (English)	Nationwide lockdown extended till April 15
2020-05-07	Himalayan Times (English)	Nationwide lockdown extended till May 18
2020-04-27	Himalayan Times (English)	Nationwide lockdown extended till May 7
2020-06-18	Himalayan Times (English)	NC registers notice of attention at HoR over govt handling of COVID-19
2020-06-17	Himalayan Times (English)	NC calls for new testing guidelines
2020-05-07	Himalayan Times (English)	NC forms 14-member special committee
2020-06-01	Himalayan Times (English)	NC leaders find fault in fiscal budget

Date of publication (yyyy/mm/dd)	Name of news media (select from drop-down menu)	Title of news/headline
2020-05-25	Himalayan Times (English)	NC proposes relief for COVID-hit businesses
2020-03-28	Himalayan Times (English)	NC submits memo to high-level committee
2020-06-17	Himalayan Times (English)	NC submits memorandum to Gandaki CM
2020-06-16	Himalayan Times (English)	NC takes govt to task as contagion spreads
2020-05-16	Himalayan Times (English)	NC urges government to rescue stranded Nepalis
2020-05-14	Himalayan Times (English)	NC urges govt to act responsibly
2020-04-16	Himalayan Times (English)	NC urges govt to fully gear up against COVID-19
2020-06-11	Himalayan Times (English)	NC urges govt to get its act together
2020-04-19	Himalayan Times (English)	NC urges govt to help stranded persons reach home safely
2020-08-14	Himalayan Times (English)	Nearly a quarter of workers lost jobs due to coronavirus: NRB
2020-06-29	Online Khabar (Nepali)	Negative in Saudi, positive in Khotang! Sealed in Rupakot
2020-07-01	Kantipur (Nepali)	Negligence in Home Quarantine
2020-01-25	Himalayan Times (English)	Nepal confirms first case of coronavirus
2020-03-24	Himalayan Times (English)	Nepal confirms second COVID-19 case
2020-02-08	Himalayan Times (English)	Nepal donates masks to China
2020-03-25	Himalayan Times (English)	Nepal government lets in stranded Nepalis within days of sealing border
2020-02-26	Himalayan Times (English)	Nepal ill-prepared for coronavirus outbreak
2020-03-05	Himalayan Times (English)	Nepal Police to launch awareness programme
2020-07-31	Himalayan Times (English)	Nepal to reopen Everest to climbers despite coronavirus case rise
2020-06-02	Himalayan Times (English)	Nepal volunteers become local heroes during virus pandemic
2020-01-17	Himalayan Times (English)	Nepal vulnerable to new China virus, warn doctors
2020-06-25	Himalayan Times (English)	Nepal, World Bank sign $100 million DPC
2020-06-16	Himalayan Times (English)	Nepal’s coronavirus count tops 6,000
2020-08-25	Himalayan Times (English)	Nepal’s COVID death toll tops 150

Date of publication (yyyy/mm/dd)	Name of news media (select from drop-down menu)	Title of news/headline
2020-07-22	Himalayan Times (English)	Nepal’s COVID recovery rate: 69.34 per cent
2020-04-30	Himalayan Times (English)	Nepal’s economic growth set to slump to 2.27 per cent this fiscal
2020-07-24	Himalayan Times (English)	Nepal’s economic growth to shrink to 2.1 pc: WB
2020-06-05	Himalayan Times (English)	Nepal’s economy to suffer $1.4bn hit due to coronavirus
2020-08-17	Himalayan Times (English)	Nepali Army deployed in Birgunj metropolis
2020-04-18	Himalayan Times (English)	Nepali Congress asks govt to provide relief to the poor
2020-03-05	Himalayan Times (English)	Nepali Congress postpones meetings due to coronavirus scare
2020-07-01	Himalayan Times (English)	Nepali families face hunger, skip meals as pandemic hits remittances, Page 2
2020-01-30	Kantipur (Nepali)	Nepali Students in China Say, When Will We Return Home
2020-01-27	Kantipur (Nepali)	Nepali Students in China Say: Rumor Sounds More Risky
2020-02-09	Himalayan Times (English)	Nepalis returning from Hubei to be quarantined in two NEATC buildings
2020-05-30	Himalayan Times (English)	Nepalis stranded abroad to be evacuated
2020-03-03	Himalayan Times (English)	Nepalis urged to scrap foreign visits
2020-07-08	Himalayan Times (English)	Nepal's virus caseload tops 16,000
2020-08-23	Himalayan Times (English)	New COVID-19 rules for hospitals
2020-06-07	Himalayan Times (English)	NHRC concerned about journos’ rights, condition in quarantine
2020-05-20	Himalayan Times (English)	NHRC tells government to respect rights of those in quarantine
2020-07-07	Himalayan Times (English)	NHRC urges govt to increase PCR tests
2020-04-07	Kantipur (Nepali)	लम्बियो लकडाउन
2020-04-06	Kantipur (Nepali)	ललितपुरदेखि पैदलै सप्तरी
2020-05-08	Himalayan Times (English)	NMA demands 10pc of total budget for health sector
2020-06-15	Himalayan Times (English)	No COVID-19 death of Nepalis abroad recorded last week
2020-05-01	Kantipur (Nepali)	No Demad of Loan in Banks After Lockdown
2020-06-05	Himalayan Times (English)	No insurance for coronavirus from today
2020-07-16	Himalayan Times (English)	No jobs, few crops: Coronavirus, pests leave people fearing hunger
2020-06-29	Himalayan Times (English)	No new COVID death of Nepalis abroad reported last week

Date of publication (yyyy/mm/dd)	Name of news media (select from drop-down menu)	Title of news/headline
2020-04-01	Himalayan Times (English)	No new COVID-19 case reported: Health ministry
2020-06-19	Himalayan Times (English)	No one attends funeral of coronavirus victim
2020-04-05	Himalayan Times (English)	No preparation to fight COVID-19 in Jajarkot
2020-01-27	Himalayan Times (English)	Novel coronavirus scare
2020-07-28	Himalayan Times (English)	NRB pins relief to COVID impact
2020-04-28	Himalayan Times (English)	NRB to bring relief package for businesses next week
2020-04-12	Kantipur (Nepali)	लकडाउन बढाउन मुख्यमन्त्रीहरूको सुझाव
2020-04-15	Kantipur (Nepali)	लकडाउन लम्बियो
2020-04-13	Kantipur (Nepali)	लकडाउनमा बलात्कारका ४० घटना
2020-04-21	Kantipur (Nepali)	लकडाउनमा महिला असुरक्षित
2020-04-16	Kantipur (Nepali)	लकडाउनले मात्रै समाधान हुँदैन : कांग्रेस
2020-03-25	Kantipur (Nepali)	लकडाउनकोआंशिक उल्लंघन
2020-04-14	Kantipur (Nepali)	यसरी घरमै पढाइ
2020-04-11	Kantipur (Nepali)	यसरी तयार हुदैछ विशेष अस्पताल
2020-04-20	Kantipur (Nepali)	यसरी हुदै छ १३ संक्रमितको हेरचाह
2020-08-06	Himalayan Times (English)	Odd-even rule back
2020-08-29	Himalayan Times (English)	Odd-even rule for vehicles in Kaski
2020-04-12	Kantipur (Nepali)	युवाले बनाए ‘सेनिटाइजिङ टनेल’
2020-02-07	Himalayan Times (English)	Oli directs authorities to bring back Nepalis from China
2020-04-23	Himalayan Times (English)	Oli in favour of letting leaders split parties
2020-03-14	Himalayan Times (English)	On-arrival visas scrapped till April 30
2020-08-31	Himalayan Times (English)	One house, one person PCR test in Durgabhagawati RM
2020-05-22	Himalayan Times (English)	One kept in isolation
2020-08-06	Himalayan Times (English)	One more COVID-19 death in Parsa, total casualties 17
2020-06-30	Himalayan Times (English)	One more death
2020-06-19	Himalayan Times (English)	One tests positive
2020-05-04	Himalayan Times (English)	One tests positive
2020-08-19	Himalayan Times (English)	One-week curfew clamped in valley

Date of publication (yyyy/mm/dd)	Name of news media (select from drop-down menu)	Title of news/headline
2020-06-29	Himalayan Times (English)	One-year-old recovers
2020-06-23	Himalayan Times (English)	Only lockdown has eased, for many life's looking more difficult now
2020-08-27	Himalayan Times (English)	Opposition parties find fault with government's response to pandemic
2020-07-03	Himalayan Times (English)	Over 2,000 minors infected
2020-03-31	Himalayan Times (English)	Over 500 Nepali migrant workers stranded on border
2020-06-27	Himalayan Times (English)	PA meet obstructed over quarantine guideline, Page 2
2020-07-18	Himalayan Times (English)	Panchthar district free of coronavirus cases
2020-08-22	Himalayan Times (English)	Pandemic dampens Teej spirit
2020-04-21	Himalayan Times (English)	Panel to assess corona impact on industries
2020-05-02	Kantipur (Nepali)	Parliamentary Committee Directs to Ease Lockdown
2020-03-21	Himalayan Times (English)	Partial lockdown from Monday
2020-08-25	Himalayan Times (English)	Partial lockdown in Gauradaha
2020-06-05	Himalayan Times (English)	Party venues to host foreign returnees
2020-07-21	Himalayan Times (English)	Pashupati all set to reopen for public
2020-04-09	Himalayan Times (English)	Patient dies in isolation ward
2020-02-01	Himalayan Times (English)	Patient suspected of coronavirus infection referred to Kathmandu
2020-08-22	Himalayan Times (English)	Patient tests positive for coronavirus, hospital sealed
2020-04-19	Himalayan Times (English)	Patient treated in isolation ward dies
2020-05-17	Himalayan Times (English)	Patients complain about lack of care, inadequate food
2020-04-02	Himalayan Times (English)	Patients with fever being turned away due to COVID fear
2020-07-03	Himalayan Times (English)	Pay attention to mental health, prevention of suicide: WHO
2020-08-04	Himalayan Times (English)	PCR lab set up
2020-08-19	Himalayan Times (English)	PCR lab yet to come into operation in Jhapa
2020-06-03	Himalayan Times (English)	PCR report now mandatory for COVID-19 insurance

Date of publication (yyyy/mm/dd)	Name of news media (select from drop-down menu)	Title of news/headline
2020-06-18	Himalayan Times (English)	PCR report yet to come even after two weeks
2020-08-31	Himalayan Times (English)	PCR test cheaper
2020-08-17	Himalayan Times (English)	PCR tests expanding in Sudurpaschim
2020-06-10	Himalayan Times (English)	Peaceful protesters beaten up
2020-04-10	Himalayan Times (English)	Penniless and nothing to eat
2020-03-30	Himalayan Times (English)	People mentally stressed, doctors suggest they limit social media
2020-06-01	Himalayan Times (English)	People opting to sanitise homes, office spaces as COVID-19 infections keep rising everyday, Page 6
2020-04-24	Himalayan Times (English)	People’s representatives feud mars relief distribution in Sunsari
2020-06-24	Online Khabar (Nepali)	Person in quarantine comittted sucide
2020-06-18	Himalayan Times (English)	Person who killed self tests COVID positive
2020-04-19	Kantipur (Nepali)	उपत्यकाबाट बाहिरिनेमाथि थप कडाइ
2020-06-02	Himalayan Times (English)	Plan to evacuate Nepalis from abroad unveiled
2020-06-02	Himalayan Times (English)	Plan to evacuate Nepalis from abroad unveiled, Page 1
2020-05-16	Himalayan Times (English)	Plan to revive COVID-hit economy missing
2020-05-26	Himalayan Times (English)	Plan to set up coronavirus treatment centre at Mechi Zonal Hospital draws flak
2020-08-19	Himalayan Times (English)	Plasma therapy used to treat COVID patient in Dharan
2020-07-31	Himalayan Times (English)	Pokhara hotels back in business
2020-04-18	Himalayan Times (English)	Pokhara metropolis to manage free food
2020-06-26	Himalayan Times (English)	Pokhara residents protest victim’s burial
2020-08-02	Himalayan Times (English)	Police arrest dozens of protesters
2020-03-22	Himalayan Times (English)	Police begin crackdown on rumour-mongers
2020-06-14	Himalayan Times (English)	Police fire tear gas, shots to control quarantined people
2020-06-10	Himalayan Times (English)	Police halt operation of businesses in valley
2020-08-29	Himalayan Times (English)	Police halt home delivery service, arrest staff

Date of publication (yyyy/mm/dd)	Name of news media (select from drop-down menu)	Title of news/headline
2020-07-30	Himalayan Times (English)	Police launch COVID awareness campaign
2020-03-29	Himalayan Times (English)	Police personnel at high risk of contracting COVID-19
2020-03-19	Himalayan Times (English)	Police seize one million masks
2020-04-09	Himalayan Times (English)	Police using man-catcher to detain lockdown violators
2020-06-08	Himalayan Times (English)	Poor lab testing capacity keeps people waiting for reports
2020-04-09	Himalayan Times (English)	Poor people get relief
2020-03-22	Himalayan Times (English)	Precaution adopted at Chhinnamasta temple
2020-06-01	Himalayan Times (English)	Pregnant woman recovers from COVID-19 in five days
2020-06-10	Himalayan Times (English)	Pregnant woman with COVID goes under knife
2020-07-26	Himalayan Times (English)	Preparations on to reopen hotels
2020-03-18	Himalayan Times (English)	Preparations to cope with coronavirus begin in Khotang, isolation ward set up
2020-05-11	Himalayan Times (English)	Preparations under way to add more quarantine facilities in Kavre
2020-06-26	Online Khabar (Nepali)	Prepared Phisiotherapy guideline for Corona infected
2020-03-06	Online Khabar (Nepali)	Preparing to table a proposal of urgent public importance in Parliament about COVID-19
2020-06-25	Himalayan Times (English)	Prez meets leaders
2020-06-06	Himalayan Times (English)	Prez sends message
2020-03-28	Himalayan Times (English)	Private sector concerned about delay in stimulus packages
2020-04-10	Himalayan Times (English)	Private sector keen to mass produce PPE, seeks incentives
2020-03-31	Himalayan Times (English)	Private sector rues inadequate relief package
2020-05-19	Himalayan Times (English)	Private sector threatens to take to the streets if demands not met in budget
2020-08-19	Himalayan Times (English)	Prohibitory order extended by 15 more days in Saptari
2020-08-30	Himalayan Times (English)	Prohibitory order extended in Kailali, Jhapa districts
2020-06-04	Himalayan Times (English)	Prohibitory order imposed in Gulmi, Jumla to contain coronavirus spread
2020-08-22	Himalayan Times (English)	Prohibitory order imposed in Kailali

Date of publication (yyyy/mm/dd)	Name of news media (select from drop-down menu)	Title of news/headline
2020-08-06	Himalayan Times (English)	Prohibitory order in 15 districts to curb COVID
2020-06-06	Himalayan Times (English)	Prohibitory order in two local levels
2020-08-16	Himalayan Times (English)	Prohibitory order lifted in Banke district
2020-08-21	Himalayan Times (English)	Prohibitory order, lockdown inadequate to fight COVID-19 pandemic, says NC
2020-08-27	Himalayan Times (English)	Prohibitory orders extended till September 2
2020-08-26	Himalayan Times (English)	Prohibitory orders in valley extended till September 2
2020-03-26	Himalayan Times (English)	Protective gear shortage imperils health workers
2020-02-15	Online Khabar (Nepali)	Protest in Nagarkot regarding qarantine for pilots and health workers who bring Chinese students
2020-03-22	Himalayan Times (English)	Province 1 government to construct 50-bed hospital
2020-03-21	Himalayan Times (English)	Province 1 govt to set up 50-bed provisional hospital in Biratnagar
2020-04-13	Himalayan Times (English)	Province 2 government expedites bid to trace contacts of three positive cases
2020-04-07	Himalayan Times (English)	Provinces following federal government’s one-door policy
2020-03-24	Kantipur (Nepali)	एक साता मुलुक बन्द
2020-04-08	Kantipur (Nepali)	एकै दिन दुई सयको स्वाब संकलन
2020-06-24	Himalayan Times (English)	Qarantined youth kills self
2020-05-02	Himalayan Times (English)	Quarantine centres in Bhojpur ill-managed, lack basic facilities
2020-04-05	Himalayan Times (English)	Quarantine centres sans basic facilities
2020-07-06	Himalayan Times (English)	Quarantine death
2020-07-05	Himalayan Times (English)	Quarantine facilities emptying in Province 2
2020-05-28	Himalayan Times (English)	Quarantine facilities in Tanahun ill-managed
2020-05-27	Himalayan Times (English)	Quarantine facility poses risk of virus spread
2020-02-04	Himalayan Times (English)	Quarantine location to be finalised today
2020-04-07	Himalayan Times (English)	Quarantine period could be extended
2020-06-30	Kantipur (Nepali)	Quarantine Remained Unsuccessful
2020-06-15	Himalayan Times (English)	Quarantine set up

Date of publication (yyyy/mm/dd)	Name of news media (select from drop-down menu)	Title of news/headline
2020-06-28	Himalayan Times (English)	Quarantine shelters emptying in Banke
2020-07-12	Himalayan Times (English)	Quarantined elderly dies
2020-05-29	Himalayan Times (English)	Quarantined people living in poorly managed facilities
2020-05-09	Himalayan Times (English)	Quarantined people sent home without any test
2020-06-18	Himalayan Times (English)	Quarantined people sent home without test results, villagers panic
2020-06-02	Himalayan Times (English)	Quarantined people's swabs collected after minor's death
2020-06-02	Himalayan Times (English)	Quarantined people's swabs collected after minor's death, Page 2
2020-06-08	Himalayan Times (English)	Quarantined youths die
2020-06-03	Himalayan Times (English)	Quarantines in Sudurpaschim bursting at seams
2020-06-22	Online Khabar (Nepali)	Question on Health Minister and Advisor Karki regarding Omni case
2020-03-25	Kantipur (Nepali)	चैत १५ गतेभित्र आपूर्ति गरिसकिने
2020-05-31	Himalayan Times (English)	Rajbiraj-based hospital to begin tests for coronavirus
2020-08-14	Himalayan Times (English)	Ramechhap municipal office halts services after staff infected
2020-04-06	Himalayan Times (English)	Rapid coronavirus antibodies tests begin today
2020-04-08	Himalayan Times (English)	Rapid diagnostic test starts in Kailali, Kanchanpur
2020-04-13	Himalayan Times (English)	Rapid diagnostic testing picks up pace in Gandaki Province
2020-04-10	Himalayan Times (English)	Rapid diagnostic tests carried out
2020-02-15	Himalayan Times (English)	Rasuwagadhi border to be closed till February 21
2020-01-30	Himalayan Times (English)	Rasuwagadi border shut over coronavirus threat
2020-03-23	Himalayan Times (English)	Rautahat border points shut
2020-08-16	Himalayan Times (English)	Rautahat local levels join hands to set up isolation ward, PCR lab
2020-04-12	Himalayan Times (English)	RDT kits in Nawalpur
2020-06-27	Online Khabar (Nepali)	Recovered from Corona but scared of neigbours
2020-05-04	Himalayan Times (English)	Recovered patients in hospital again

Date of publication (yyyy/mm/dd)	Name of news media (select from drop-down menu)	Title of news/headline
2020-07-09	Himalayan Times (English)	Releif distributed
2020-05-08	Himalayan Times (English)	Relief distributed
2020-07-16	Himalayan Times (English)	Relief distributed among farmers
2020-05-12	Himalayan Times (English)	Relief distributed among more than 5,000 families
2020-03-30	Himalayan Times (English)	Relief for hunkered down populace
2020-07-25	Himalayan Times (English)	Relief materials distributed
2020-04-10	Himalayan Times (English)	Relief packages elude the poorest of the poor
2020-04-30	Himalayan Times (English)	Relief provided
2020-04-23	Himalayan Times (English)	Remittances to Nepal to slump 14 per cent
2020-01-26	Kantipur (Nepali)	Request Hospitals to Submit Workplan
2020-05-01	Kantipur (Nepali)	Request To Invest More On Infrastructure
2020-05-28	Himalayan Times (English)	Restrictions eased in Bhulke after a month
2020-03-30	Himalayan Times (English)	Returnee migrant workers from India resist quarantine
2020-04-06	Himalayan Times (English)	Returnees in Lamjung refuse to stay in quarantine
2020-04-30	Himalayan Times (English)	Returnees not staying in self-quarantine in Kavre
2020-02-26	Himalayan Times (English)	Revenue collection nil at Rasuwa customs
2020-07-17	Himalayan Times (English)	Reverse migration puts spotlight on social, economic reintegration of returnees
2020-04-01	Himalayan Times (English)	Reverse migration spreads to Nepal
2020-04-18	Himalayan Times (English)	Reverse migration: Govt told to arrange transport for those trudging back home
2020-04-01	Himalayan Times (English)	Rise in GBV, sexual abuse cases feared
2020-01-26	Kantipur (Nepali)	Risk of Novel Corona Virus (Editorial)
2020-04-14	Himalayan Times (English)	Rs 1.98 billion collected for coronavirus fund
2020-05-30	Himalayan Times (English)	Rs 210-billion stimulus package in budget: FinMin
2020-04-22	Himalayan Times (English)	Rs 4 billion already spent to combat coronavirus
2020-04-09	Himalayan Times (English)	RTPCR machine installed in Dhangadi

Date of publication (yyyy/mm/dd)	Name of news media (select from drop-down menu)	Title of news/headline
2020-08-10	Himalayan Times (English)	Rural municipality in Jhapa imposes lockdown
2020-04-09	Kantipur (Nepali)	कस्तो पीपीई सुरक्षित ?
2020-04-09	Kantipur (Nepali)	कसलाई कति प्रोत्साहन भत्ता ?
2020-04-05	Kantipur (Nepali)	कैलाली, कञ्चनपुर र बागलुङमा ‘ र्‍यापिड टे स्ट’ बढाइन
2020-03-26	Kantipur (Nepali)	कर्णालीका १०३ जना क्वारन्टाइनमा
2020-03-17	Online Khabar (Nepali)	Sajha bus started providing service only after spraying sanitizer
2020-01-23	Kantipur (Nepali)	Sample of Suspected Patient Has Sent to Hong Kong
2020-01-21	Kantipur (Nepali)	Sample of Suspected Patient sent to Aboard for Testing
2020-03-01	Himalayan Times (English)	Samples collected for second time from quarantined Nepalis
2020-04-27	Himalayan Times (English)	Sanitiser tunnel
2020-07-17	Himalayan Times (English)	Saptari dwellers discard lockdown rules as Bihar poses virus threat
2020-06-05	Himalayan Times (English)	Saptari’s first Covid patient recovers
2020-03-05	Himalayan Times (English)	Sauraha hotels start cutting down on staff
2020-07-11	Himalayan Times (English)	Saving lives vital, NCP feud trivial, says PM Oli
2020-04-17	Himalayan Times (English)	SC asks govt to bring back migrant Nepalis stranded in foreign lands
2020-06-15	Himalayan Times (English)	Second death in Gulmi
2020-06-24	Himalayan Times (English)	Second death in Dailekh
2020-05-04	Himalayan Times (English)	Security on Nepal-India border to be tightened
2020-05-06	Himalayan Times (English)	Security tightened in Bhimad Municipality
2020-05-03	Himalayan Times (English)	SEE new dates uncertain due to govt indecision on lockdown
2020-03-28	Himalayan Times (English)	Senior citizens to get Rs 10,000 per month
2020-02-12	Himalayan Times (English)	Separate room for coronavirus patients
2020-04-08	Himalayan Times (English)	Seti Hospital health workers at risk
2020-06-04	Himalayan Times (English)	Seti Provincial Hospital refuses to admit COVID patients
2020-08-06	Himalayan Times (English)	Seven govt offices in Banke district sealed
2020-05-06	Himalayan Times (English)	Seven more COVID cases detected in Nepalgunj
2020-08-19	Himalayan Times (English)	Seven more succumb to new coronavirus; Nepal toll 114

Date of publication (yyyy/mm/dd)	Name of news media (select from drop-down menu)	Title of news/headline
2020-05-03	Himalayan Times (English)	Seven police personnel quarantined
2020-08-26	Himalayan Times (English)	Seven succumb to infection
2020-07-02	Himalayan Times (English)	Seventeen more COVID cases in valley
2020-05-07	Himalayan Times (English)	Seventeen of a Parsa family test positive for coronavirus infection
2020-04-10	Kantipur (Nepali)	कोरोना कोषमा ३० लाख
2020-04-10	Kantipur (Nepali)	काठमाडौंबाट पैदलै बाँके-बर्दिया
2020-05-06	Himalayan Times (English)	Shortage of laboratory materials imperils novel coronavirus testing
2020-08-28	Himalayan Times (English)	Shortage of VTM mars swab collection
2020-06-02	Himalayan Times (English)	Single-day COVID surge tops 200
2020-08-20	Himalayan Times (English)	Six fatalities
2020-05-07	Himalayan Times (English)	Six Indians discharged
2020-08-21	Himalayan Times (English)	Six succumb to virus
2020-03-31	Himalayan Times (English)	Six youths who flew with man who tested positive for COVID-19 quarantined
2020-05-04	Himalayan Times (English)	Sixteen test positive for neo-coronavirus
2020-06-13	Himalayan Times (English)	Sixteenth victim
2020-03-27	Kantipur (Nepali)	क्वारेन्टाइनबाट धमाधम भाग्द
2020-03-24	Kantipur (Nepali)	क्वारन्टाइनबाट ५४० जना भाग
2020-04-16	Kantipur (Nepali)	क्वारन्टाइनमैं किरिया
2020-03-26	Kantipur (Nepali)	क्वारन्टाइनमा राख्नै मुस्किल
2020-05-01	Kantipur (Nepali)	Slow Speed of Pandemic
2020-05-01	Kantipur (Nepali)	Slow Testing in Risky Zone
2020-06-11	Himalayan Times (English)	Space crunch forces COVID suspects into tent
2020-08-01	Himalayan Times (English)	Staffer gets infected, Banke district court closed
2020-01-26	Himalayan Times (English)	STIDH admits 2019-nCov suspects
2020-03-16	Himalayan Times (English)	Stock of kits to transport coronavirus samples limited
2020-06-08	Himalayan Times (English)	Stop RDTs: PA members
2020-02-12	Online Khabar (Nepali)	Story behind delaying in rescue of Nepalis from China
2020-04-13	Himalayan Times (English)	Substandard rice distributed to poor people in Bajura

Date of publication (yyyy/mm/dd)	Name of news media (select from drop-down menu)	Title of news/headline
2020-06-30	Kantipur (Nepali)	Sucide Can be Prevented Through Psychosocial Couseling
2020-06-30	Kantipur (Nepali)	Sucide or Murder!
2020-04-06	Himalayan Times (English)	Sudurpaschim Province expediting efforts to contain pandemic
2020-05-01	Kantipur (Nepali)	Suggest to Make Lockdown Ease
2020-07-29	Himalayan Times (English)	Suicide case tests COVID positive
2020-04-06	Himalayan Times (English)	Sukraraj hospital needs anaesthetists, critical care physicians
2020-07-31	Himalayan Times (English)	Sunsari denizens cautioned on threat of COVID-19 community spread
2020-03-06	Himalayan Times (English)	Supply of essentials intact, says govt
2020-06-06	Himalayan Times (English)	Surgical, N-95 masks mandatory in airports
2020-03-19	Himalayan Times (English)	Surveillance room established at Trinagar customs point as preventive measure
2020-05-03	Himalayan Times (English)	Swab collection booth
2020-06-06	Himalayan Times (English)	Swab collection halted for five days in Sudurpaschim
2020-06-09	Himalayan Times (English)	Swab collection halted in Jajarkot
2020-07-13	Himalayan Times (English)	Swab collection slows down
2020-08-09	Himalayan Times (English)	Swab sample collected from communities
2020-04-13	Kantipur (Nepali)	कहाँ-कहाँ घुमे तीन संक्रमित ?
2020-01-30	Kantipur (Nepali)	Symptom Could be Seen within 10 Days
2020-06-26	Himalayan Times (English)	Tanahun's Myagde RM closed for a week
2020-03-18	Himalayan Times (English)	Taskforce to tackle virus spread formed
2020-02-11	Himalayan Times (English)	Tatopani border closed
2020-06-04	Himalayan Times (English)	Tax clearance deadline put off till June 21
2020-07-01	Kantipur (Nepali)	Taxi Drivers Are in Strike
2020-08-25	Himalayan Times (English)	Technicians busy conducting PCR tests
2020-03-21	Himalayan Times (English)	Teku lab to get kits for testing 20,000 samples
2020-06-12	Himalayan Times (English)	Temporary COVID-19 hospital established
2020-03-21	Himalayan Times (English)	Temporary hospital established to treat COVID-19 patients comes into operation

Date of publication (yyyy/mm/dd)	Name of news media (select from drop-down menu)	Title of news/headline
2020-03-15	Himalayan Times (English)	Tension grips Nepalgunj as two cases of COVID-19 confirmed in Lucknow
2020-06-05	Himalayan Times (English)	Tenth COVID death
2020-04-01	Himalayan Times (English)	Test for virus to start in Pokhara
2020-05-08	Himalayan Times (English)	Testing expanded in coronavirus hotspots
2020-07-04	Himalayan Times (English)	Testing extended
2020-01-28	Himalayan Times (English)	Testing for coronavirus starts in Nepal
2020-05-01	Kantipur (Nepali)	Testing of COVID-19 has Stopped in the Absence of Kit
2020-05-20	Himalayan Times (English)	Thakre Rural Municipality sealed
2020-02-09	Online Khabar (Nepali)	The 'main criteria' for the rescue of Nepalis in Wuhan have yet to be met!
2020-07-01	Kantipur (Nepali)	The Residents of Kathmandu Are Doing Negligence
2020-06-25	Online Khabar (Nepali)	The villagers sacrificed animals to drive away Corona, the leader says - superstition
2020-03-15	Online Khabar (Nepali)	The work of the Ministry of Health for Corona: 'Theory' is fine
2020-06-22	Himalayan Times (English)	Third death in Gulmi
2020-05-01	Kantipur (Nepali)	Thousands are Happy and Millions are at Waiting
2020-08-05	Himalayan Times (English)	Thousands return home, desire to work
2020-08-06	Himalayan Times (English)	Three COVID suspects die in Saptari district
2020-08-29	Himalayan Times (English)	Three COVID-19 patients die at Dharan hospital
2020-08-24	Himalayan Times (English)	Three more fatalities
2020-07-28	Himalayan Times (English)	Three more succumb to COVID-19
2020-06-27	Himalayan Times (English)	Three of 593 new COVID cases from Kathmandu
2020-01-27	Kantipur (Nepali)	Three People are Suspected to Be Corona Infected
2020-08-09	Himalayan Times (English)	Three persons succumb to COVID
2020-04-24	Himalayan Times (English)	Three recover, discharged
2020-08-16	Himalayan Times (English)	Three succumb to coronavirus
2020-08-18	Himalayan Times (English)	Three succumb to the infection
2020-03-31	Himalayan Times (English)	Three suspected COVID-19 patients die while undergoing treatment in capital hospitals

Date of publication (yyyy/mm/dd)	Name of news media (select from drop-down menu)	Title of news/headline
2020-06-21	Online Khabar (Nepali)	Three villages sealed when one pregnant woman got COVID-19 positive
2020-04-12	Himalayan Times (English)	Three virus infected persons test positive again
2020-07-14	Himalayan Times (English)	Three wards of Lalitpur’s Mahalaxmi Municipality sealed
2020-05-02	Himalayan Times (English)	Throat swabs
2020-01-28	Himalayan Times (English)	TIA told to manage separate service for arrivals from China
2020-06-26	Online Khabar (Nepali)	Tighten Lockdown in Bhimsen Thapa Rural municipality
2020-04-17	Kantipur (Nepali)	तीन सातापछि पनि र हिरहन्छ संक्रमण
2020-05-01	Kantipur (Nepali)	To Receive Relief Package One has to Obey the Government
2020-03-27	Kantipur (Nepali)	तयारी अवस्थामा हब अस्पताल
2020-03-01	Online Khabar (Nepali)	Tokha Festival has been postponed due to Corona's terror
2020-06-02	Himalayan Times (English)	Tourist standard hotels agree to government rate for quarantine
2020-01-29	Online Khabar (Nepali)	Tourists are barred from entering Nepal from Rasuwagadhi and Tatopani
2020-06-04	Himalayan Times (English)	Traders defy lockdown as patience runs thin
2020-07-14	Himalayan Times (English)	Transport workers demand salary for lockdown period in Gandaki
2020-04-17	Himalayan Times (English)	Trauma centre starts RDT for patients who need surgery
2020-06-30	Kantipur (Nepali)	Treaated Like Prisoner
2020-06-30	Kantipur (Nepali)	Treated Like Prisoner
2020-04-14	Himalayan Times (English)	Twenty-six put under quarantine again due to ‘negligence of authorities’
2020-04-19	Himalayan Times (English)	Two COVID-19 patients discharged
2020-05-11	Himalayan Times (English)	Two COVID-19 patients discharged from hospital
2020-03-18	Himalayan Times (English)	Two COVID-19 suspects admitted to STIDH
2020-05-19	Himalayan Times (English)	Two hospitals sealed after COVID cases
2020-03-05	Online Khabar (Nepali)	Two medical clinics locked due to black market in masks
2020-06-14	Himalayan Times (English)	Two more succumb to COVID
2020-04-20	Himalayan Times (English)	Two more labs ready to verify PCR tests
2020-08-17	Himalayan Times (English)	Two more lives lost
2020-01-26	Online Khabar (Nepali)	Two more Nepalis are suspected of having corona virus

Date of publication (yyyy/mm/dd)	Name of news media (select from drop-down menu)	Title of news/headline
2020-06-19	Himalayan Times (English)	Two more succumb to virus
2020-05-02	Himalayan Times (English)	Two more test positive for COVID-19
2020-03-13	Himalayan Times (English)	Two quarantine centres to be established in Province 1
2020-04-23	Himalayan Times (English)	Two recover
2020-05-08	Himalayan Times (English)	Two test positive for COVID-19 in RDT
2020-08-01	Himalayan Times (English)	Two-week lockdown in Biratnagar
2020-03-27	Kantipur (Nepali)	गाउँमा पालना भएन लकडाउन
2020-06-10	Himalayan Times (English)	UNDP initiates livelihoods recovery programme
2020-04-21	Himalayan Times (English)	UNFPA hands over 1,200 PPE kits to government
2020-07-09	Himalayan Times (English)	Unilever’s initiative
2020-06-20	Himalayan Times (English)	Unspent pork barrel fund for COVID-19 fight
2020-06-25	Himalayan Times (English)	Urgent action needed to safeguard millions of children's future, says UNICEF
2020-03-07	Himalayan Times (English)	US provides PPE to contain COVID-19
2020-03-26	Himalayan Times (English)	Utilise lockdown time to test all suspected cases: Thapa
2020-05-16	Himalayan Times (English)	Valley local levels to conduct more tests to control coronavirus
2020-07-06	Himalayan Times (English)	Valley witnesses 92 new cases in a week
2020-04-24	Himalayan Times (English)	Vehicles allowed entry after sanitisation
2020-05-15	Himalayan Times (English)	Vehicles held for defying lockdown
2020-07-23	Himalayan Times (English)	Vehicular movement resumes in full swing in Kathmandu valley
2020-07-07	Himalayan Times (English)	Venturing out without wearing mask banned
2020-04-09	Kantipur (Nepali)	खाडीमा हजारौंको रोजगारी गुम्दै
2020-03-27	Kantipur (Nepali)	खाद्यवस्तु घरघर पुऱ्याउन गृहको निर्देशन
2020-04-16	Kantipur (Nepali)	खाने चीज न जाने ठाउ
2020-07-02	Himalayan Times (English)	Virus claims 45-year-old man
2020-08-14	Himalayan Times (English)	Virus kills one

Date of publication (yyyy/mm/dd)	Name of news media (select from drop-down menu)	Title of news/headline
2020-08-13	Himalayan Times (English)	Virus spreads at community level in Jhapa
2020-03-10	Himalayan Times (English)	Visa hurdle for citizens of eight corona-hit countries
2020-06-26	Himalayan Times (English)	Vitamin A, deworming drug drive on July 6, 7
2020-04-06	Kantipur (Nepali)	खरिपाटी क्वारन्टाइनको प्रशंसा किन हुन्छ ?
2020-06-10	Himalayan Times (English)	VTM crunch hits COVID testing
2020-07-17	Himalayan Times (English)	VTM kits donated
2020-05-28	Himalayan Times (English)	Wage earners hit hard due to prolonged lockdown
2020-08-27	Himalayan Times (English)	Ward office sets up 30-bed isolation facility
2020-01-26	Kantipur (Nepali)	Warnning to Chinese People
2020-04-05	Kantipur (Nepali)	wdfwd cfsl:ds sf]if
2020-03-24	Himalayan Times (English)	Week-long nationwide lockdown from today
2020-03-15	Online Khabar (Nepali)	Western Nepal Bus Company will carry passengers only after measuring the temperature
2020-04-30	Himalayan Times (English)	WFP sounds food crisis warning
2020-01-25	Kantipur (Nepali)	What is Novel Corana Virus?
2020-05-04	Himalayan Times (English)	Where returnees prefer cave to quarantine
2020-06-23	Online Khabar (Nepali)	Why PCR kit of 6.5 crore brought by army did not fit in labs?
2020-02-10	Online Khabar (Nepali)	Widebody flying china to bring nepali on Falgun 3
2020-07-23	Himalayan Times (English)	Willpower needed to defeat COVID-19'
2020-05-30	Himalayan Times (English)	Woman delivers baby in quarantine facility
2020-05-02	Himalayan Times (English)	Woman dies in isolation ward of Achham hospital
2020-06-03	Himalayan Times (English)	Woman dies in quarantine
2020-06-04	Himalayan Times (English)	Woman quarantined in Dadeldhura dies
2020-04-27	Himalayan Times (English)	Woman tests positive in RDT thrice, dies
2020-07-11	Himalayan Times (English)	Woman with COVID-19 delivers baby
2020-06-18	Himalayan Times (English)	Women's quarantine
2020-05-01	Kantipur (Nepali)	Work Halt Led Livelihood Stopped
2020-05-27	Himalayan Times (English)	Work in two teams, directs health ministry

Date of publication (yyyy/mm/dd)	Name of news media (select from drop-down menu)	Title of news/headline
2020-04-26	Himalayan Times (English)	Work to increase isolation ward’s capacity under way
2020-06-12	Himalayan Times (English)	World Bank’s $450m road support in Nepal to spur COVID-19 recovery
2020-03-25	Kantipur (Nepali)	हामी साथमा छौं : चीन
2020-06-11	Himalayan Times (English)	Yangbarak Rural Municipality procures VTM kits for swab test
2020-06-18	Himalayan Times (English)	Yet another suicide case tests positive for coronavirus
2020-06-05	Himalayan Times (English)	Yet another unprecedented overnight spike in COVID cases
2020-04-09	Kantipur (Nepali)	थप रणनीति बनाउन चार कार्यदल
2020-08-09	Himalayan Times (English)	Young woman dies for want of treatment
2020-07-27	Himalayan Times (English)	Youth activist’s fast-unto-death enters ninth day, government indifferent
2020-07-08	Himalayan Times (English)	Youth activists end hunger strike as govt buckles
2020-07-18	Himalayan Times (English)	Youth found dead in Lamjung quarantine
2020-07-05	Himalayan Times (English)	Youth kills self after testing positive
2020-05-02	Himalayan Times (English)	Youth kills self in quarantine
2020-06-06	Himalayan Times (English)	Youth leaves quarantine with no symptoms, dies at home of COVID!
2020-06-22	Himalayan Times (English)	Youth passes current into water tank, woman electrocuted
2020-04-26	Himalayan Times (English)	Youth recovers
2020-08-21	Himalayan Times (English)	Youth tests positive for virus after death
2020-08-16	Himalayan Times (English)	Youths ban entry of outsiders
2020-06-14	Himalayan Times (English)	Youths continue anti-government protest
2020-06-12	Himalayan Times (English)	Youths protest against government
2020-06-21	Himalayan Times (English)	Youths take to street against poor handling of COVID-19 yet again
2020-05-04	Kantipur (Nepali)	अग्रपंक्तिमा खटिनेले पाएनन् भत्ता
2020-07-08	Kantipur (Nepali)	अझै बनेन कोभिड अस्पताल
2020-04-28	Kantipur (Nepali)	अत्यावश्यक बाहेकलाई उपत्यका छिर्न नदिनू : गृह
2020-03-21	Kantipur (Nepali)	अत्यावश्यकबाहेकका सेवा चैत २१ सम्म बन्द
2020-05-31	Kantipur (Nepali)	अधिकांश संक्रमितमा लक्षण छैन
2020-03-13	Kantipur (Nepali)	अनअराइभल भिसा वैशाख १८ सम्म बन्द

Date of publication (yyyy/mm/dd)	Name of news media (select from drop-down menu)	Title of news/headline
2020-07-07	Kantipur (Nepali)	अनलाइन कक्षा बन्द गर्ने विद्यालयको चेतावनी
2020-07-19	Online Khabar (Nepali)	अनलाइन कक्षा सञ्चालन गर्न शिक्षा मन्त्रालयको अनुरोध
2020-08-28	Online Khabar (Nepali)	अनलाइन डेलिभरीमा सीडीओको नीति वुहानको भन्दा कडा !
2020-06-03	Kantipur (Nepali)	अनुमति दिन आलटाल
2020-06-22	Kantipur (Nepali)	अनुमतिबिना उपत्यका पस्नेलाई नाकाबाटै फिर्ता
2020-03-03	Kantipur (Nepali)	अन्तर्राष्ट्रिय आवागमनमा कडाइ
2020-06-04	Kantipur (Nepali)	अन्य रोग भए कोरोनाबाट मृत्यु नमानिने
2020-06-10	Kantipur (Nepali)	अपांगता भएकाहरू ओझेलमा
2020-08-16	Kantipur (Nepali)	अपांगता भएकाहरूलाई झन् गाह्रो
2020-08-04	Online Khabar (Nepali)	अब थप सचेत हुने बेला आएको छ : मुख्यमन्त्री भट्ट
2020-03-10	Kantipur (Nepali)	अब दैनिक १० हजार मास्क उत्पादन
2020-08-11	Online Khabar (Nepali)	अब रेस्टुरेन्टमा बसेर खान नपाइने
2020-06-03	Kantipur (Nepali)	अब लकडाउन थेग्न सक्दैनौं : व्यवसायी
2020-08-21	Online Khabar (Nepali)	अब लक्षण भएका संक्रमितलाई मात्र अस्पताल भर्ना गरिने
2020-06-01	Kantipur (Nepali)	अब सोच्नैपर्छ लकडाउनको विकल्प
2020-03-07	Kantipur (Nepali)	अमेरिकी सरकारले दियो उपकरण
2020-08-31	Kantipur (Nepali)	अर्को सातादेखि समुदायमा एन्टिबडी परीक्षण
2020-06-17	Online Khabar (Nepali)	अर्घाखाँचीको क्वारेन्टिनमा एक पुरुषले गरे आत्महत्या
2020-05-18	Kantipur (Nepali)	अर्थतन्त्र उकास्न तीनखुट्टे रणनीति
2020-05-22	Kantipur (Nepali)	अर्थतन्त्र उकास्न रोजगारी र राहत'
2020-05-15	Kantipur (Nepali)	अर्थतन्त्र जोगाउन पर्याप्त राहत'
2020-05-16	Kantipur (Nepali)	अव्यवस्थित क्वारेन्टाइनले संक्रमण : विज्ञ
2020-07-19	Kantipur (Nepali)	असमानतालाई महामारीले प्रस्ट देखायो’
2020-05-24	Kantipur (Nepali)	असाधारण अवस्थामा असामान्य बजेटको अपेक्षा
2020-06-10	Kantipur (Nepali)	अस्ट्रेलियाबाट ११ आए
2020-06-24	Kantipur (Nepali)	अस्ट्रेलियाबाट उडे २५९ नेपाली
2020-03-19	Kantipur (Nepali)	अस्पताल प्रवेशअघि ‘थर्मल गन’
2020-05-17	Kantipur (Nepali)	अस्पताल र स्वास्थ्यकर्मीबाट पनि संक्रमणको जोखिम
2020-07-12	Kantipur (Nepali)	अस्पताल सिल गर्नु पर्दैन'
3/22/20202	Kantipur (Nepali)	अस्पतालका ओपीडी खुलै
2020-07-11	Kantipur (Nepali)	अस्पतालको व्यवस्थापन हाम्रो होइन’
2020-02-04	Kantipur (Nepali)	अस्पतालमा छैनन् सुरक्षा सामग्री
2020-08-12	Online Khabar (Nepali)	अस्पतालमा मृत्यु भएका धादिङका युवकमा कोरोना पुष्टि
2020-08-12	Kantipur (Nepali)	अस्पतालमा शय्या अभाव
2020-03-20	Kantipur (Nepali)	अस्पतालमै छैन ज्वरो क्लिनिक
2020-03-13	Kantipur (Nepali)	अस्पतालमै बन्छ स्यानिटाइजर
2020-05-25	Online Khabar (Nepali)	अस्पतालमै मनाए कोरोना संक्रमितले ईद
2020-04-18	Online Khabar (Nepali)	अस्पताललाई कोभिड-१९ स्क्रिनिङ बुथ हस्तान्तरण
2020-08-14	Kantipur (Nepali)	अस्पताललाई युद्धस्तरमा सुधारौं
2020-05-25	Kantipur (Nepali)	अस्पतालहरूको दायित्व
2020-08-19	Kantipur (Nepali)	अस्पतालहरूमा संक्रमित अटाउनै मुस्किल
2020-08-26	Kantipur (Nepali)	अस्वाभाविक मूल्यवृद्धिले सास्ती
2020-05-23	Kantipur (Nepali)	अहिलेसम्म ७० निको भए

Date of publication (yyyy/mm/dd)	Name of news media (select from drop-down menu)	Title of news/headline
2020-06-06	Kantipur (Nepali)	आइपुगे १९४ जना
2020-05-14	Kantipur (Nepali)	आइसोलेसन नहुँदा पर्साका संक्रमित घरमै
2020-08-23	Online Khabar (Nepali)	आइसोलेसन बढाउन नेकपा रुपन्देहीको माग
2020-08-13	Online Khabar (Nepali)	आइसोलेसन बनाउन प्रदेश १ सरकारबाट १ करोड ६० लाख निकासा
2020-03-13	Kantipur (Nepali)	आइसोलेसन बेड थपियो
2020-03-17	Kantipur (Nepali)	आइसोलेसन बेड राख्न निर्देशन
2020-06-02	Kantipur (Nepali)	आइसोलेसन र क्वारेन्टाइन भद्रगोल
2020-02-06	Kantipur (Nepali)	आइसोलेसन सेन्टरबारे सरकार अनिर्णीत
2020-05-25	Kantipur (Nepali)	आइसोलेसनको ढोका फोडेर संक्रमित फरार
2020-03-06	Kantipur (Nepali)	आइसोलेसनको सुरक्षामा प्रहरी
2020-06-27	Kantipur (Nepali)	आइसोलेसनबाट भागेपछि सबै छाडिए
2020-06-03	Kantipur (Nepali)	आइसोलेसनमा जानै डराउँछन् स्वास्थ्यकर्मी
2020-08-14	Kantipur (Nepali)	आइसोलेसनमा बस्ने कर्मचारीको खर्च बैंकको
2020-03-17	Kantipur (Nepali)	आइसोलेसनमा व्यक्तिको घर
2020-07-31	Kantipur (Nepali)	आईसीयू जाने बिरामीको संख्या बढ्दो
2020-08-21	Kantipur (Nepali)	आईसीयू र भेन्टिलेटर थप्दै छौं : प्रवक्ता गौतम
2020-08-18	Kantipur (Nepali)	आईसीयूमा राख्नुपर्ने संक्रमितमध्ये ३ प्रतिशतसम्मको मृत्यु हुन सक्ने
2020-07-10	Kantipur (Nepali)	आतंकित होइन, संयमित बनौं
2020-05-04	Kantipur (Nepali)	आत्मनिर्भर अर्थतन्त्र निर्माणको अवसर
2020-06-29	Kantipur (Nepali)	आत्महत्या बढेर दिनहुँ १९
2020-06-28	Kantipur (Nepali)	आन्तरिक उडान कहिले खुल्छ?
2020-06-20	Kantipur (Nepali)	आफैं किट किन्दै, परीक्षण गर्दै
2020-07-12	Kantipur (Nepali)	आम बेरोजगारीले निम्त्याउने राष्ट्रिय संकट
2020-06-25	Kantipur (Nepali)	आयात खुम्चिँदा विदेशी मुद्रा सञ्चिति बढ्यो
2020-06-14	Kantipur (Nepali)	आयोजनाले पाउन थाले निर्माण सामग्री
2020-06-28	Kantipur (Nepali)	आरडीटी गर्दै घर पठाउँदै
2020-04-24	Kantipur (Nepali)	'आरडीटी पोजिटिभ र पीसीआर नेगेटिभ आए पनि पुनः परीक्षण'
2020-05-14	Online Khabar (Nepali)	आरडीटीमा सरकारको नीति : टेस्ट संख्या जोड्ने, पोजेटिभ गन्दै नगन्ने
2020-04-16	Online Khabar (Nepali)	आरुघाटका युवकको परिवार र छिमेकीमा पनि कोरोना संक्रमण परीक्षण गरिने
2020-06-11	Kantipur (Nepali)	आर्थिक क्षेत्र चलायमान बनाउन कांग्रेसको माग
2020-05-15	Kantipur (Nepali)	आर्थिक वृद्धिदर १५ हुने प्रक्षेपण
2020-05-25	Kantipur (Nepali)	आर्थिक शैथिल्यमा कोरोना मात्र दोषी छैन
2020-03-20	Kantipur (Nepali)	आवश्यक परे 'लकडाउन' गर्न मोडालिटी बनाइँदै
2020-06-29	Kantipur (Nepali)	आवेगले धेरै आत्महत्या
2020-08-22	Online Khabar (Nepali)	आशारामलाई कोभिड अस्पताल बनाउन खोज्दा स्थानीयको विरोध
2020-08-01	Kantipur (Nepali)	आश्रयस्थलबाट पनि निकालिन थाले कामदार
2020-04-16	Online Khabar (Nepali)	इटालीबाट आएकी महिलामा एन्टिबडी पोजेटिभ, टेस्ट गर्ने डाक्टर नै क्वारेन्टाइनमा
2020-08-04	Kantipur (Nepali)	ईः अस्पताल भर्ना
2020-07-06	Kantipur (Nepali)	ईटा उद्योगमा २४ हजार लाई रोजरी
2020-07-31	Online Khabar (Nepali)	उच्च अदालतको आदेश : प्रदेश २ मा भारतबाट प्रवेशमा कडाइ गर्नू

Date of publication (yyyy/mm/dd)	Name of news media (select from drop-down menu)	Title of news/headline
2020-08-28	Kantipur (Nepali)	उच्च जोखिममा कैदीबन्दी
2020-03-01	Kantipur (Nepali)	उच्चस्तरीय समन्वय समिति गठन
2020-06-12	Kantipur (Nepali)	उडान पर्खंदै ईपीएस कामदार
2020-05-01	Online Khabar (Nepali)	उदयपुरका बिरामी अस्पतालबाट फेरि निकालिए
2020-04-23	Online Khabar (Nepali)	उदयपुरमै कोरोना परीक्षण गर्न माग
2020-05-20	Kantipur (Nepali)	उद्धार गर्न ३ महिना लाग्ने
2020-07-13	Kantipur (Nepali)	उद्धारका लागि थप उडान
2020-05-19	Kantipur (Nepali)	उद्यम चलाउन उद्योग र वाणिज्यको पास
2020-05-15	Kantipur (Nepali)	उद्योग र आयोजना सञ्चालन पुनः अन्योलमा
2020-06-08	Kantipur (Nepali)	उद्योगलाई कामदारकै पिरलो
2020-05-30	Kantipur (Nepali)	उद्योगलाई प्रोत्साहन
2020-06-04	Kantipur (Nepali)	उद्योगीको विरोधपछि कर तिर्ने अवधि थपियो
2020-05-04	Kantipur (Nepali)	उद्योगीले मागे संरक्षण प्याकेज
2020-08-18	Kantipur (Nepali)	उपचार नपाइने भो
2020-08-09	Online Khabar (Nepali)	उपचार नपाएर मर्ने क्रम बढेको भन्दै वीरगञ्जमा प्रदर्शन
2020-05-12	Kantipur (Nepali)	उपचारमा आयुर्वेदिक औषधि !
2020-03-17	Kantipur (Nepali)	उपचारमा खट्दा संक्रमण भए १० लाख
2020-05-14	Kantipur (Nepali)	उपत्यका छिर्नेको मात्र परीक्षण !
2020-07-31	Kantipur (Nepali)	उपत्यका छिर्नेलाई निगरानी छैन
2020-07-01	Online Khabar (Nepali)	उपत्यका प्रवेश गर्ने नाकाबाट झण्डै ४ सय गाडी फर्काइयो
2020-06-15	Kantipur (Nepali)	उपत्यका प्रवेशमा तीन पक्षको स्वीकृति अनिवार्य
2020-05-14	Online Khabar (Nepali)	उपत्यका फर्किनेलाई आफ्नै कोठामा प्रवेश निषेध !
2020-05-08	Kantipur (Nepali)	उपत्यका भित्रिँदै छन् दिनमा पाँच हजार
2020-07-08	Kantipur (Nepali)	उपत्यका समुदाय संक्रमणको संघारमा’
2020-05-26	Kantipur (Nepali)	उपत्यकाका नाकामा ‘डेडिकेटेड टिम’ कहिले ?
2020-06-16	Kantipur (Nepali)	उपत्यकाका सपिङ मल खुले
2020-08-17	Online Khabar (Nepali)	उपत्यकामा कोरोना संक्रमितलाई अस्पताल पुर्‍याउनेहरु
2020-08-23	Online Khabar (Nepali)	उपत्यकामा खाद्यान्न र तरकारी पसल बिहान ९ बजेसम्म मात्र खोल्न पाइने
2020-06-23	Kantipur (Nepali)	उपत्यकामै घट्यो परीक्षण
2020-06-23	Kantipur (Nepali)	उपत्यकालाई जोगाऔं
2020-04-06	Online Khabar (Nepali)	ऊर्जामन्त्री पुनले रोल्पा पठाए पीपीईसहित स्वास्थ्य सामग्री
2020-07-09	Kantipur (Nepali)	ऋणको भुक्तानी म्याद बढाउन माग
2020-07-05	Kantipur (Nepali)	ऋणपत्र निष्कासन गर्न बैंकहरूलाई थप समय
2020-07-07	Online Khabar (Nepali)	एक घर, एक पीसीआर टेस्टको माग गर्दै गण्डकी प्रदेशमा संकल्प प्रस्ताव
2020-05-17	Kantipur (Nepali)	एक प्रतिशत बिन्दुले गरिबी बढ्ने
2020-04-10	Online Khabar (Nepali)	एक महिनामा झण्डै ३५ करोडको स्वास्थ्य सामग्री आयात
2020-05-26	Kantipur (Nepali)	एक महिनामा झन्डै ५० हजार भित्रिए
2020-05-24	Kantipur (Nepali)	एक महिनामा डेढ लाखले गरे कोरोना बिमा
2020-04-26	Kantipur (Nepali)	एक साता थप्न सिफारिस
2020-08-13	Kantipur (Nepali)	एक सातादेखि संक्रमित कोठामै
2020-05-03	Kantipur (Nepali)	एक सातामा थप १७ हजारले गरे कोरोना बिमा
2020-08-25	Kantipur (Nepali)	एकका लागि एक’ अभियानबाट पीसीआर प्रयोगशाला

Date of publication (yyyy/mm/dd)	Name of news media (select from drop-down menu)	Title of news/headline
2020-08-12	Online Khabar (Nepali)	एकजना कोरोना संक्रमित भेटिएपछि इच्छाकामना गाउँपालिका सिल
2020-07-14	Kantipur (Nepali)	एकै दिन १,७०५ जना निको भए
2020-05-10	Online Khabar (Nepali)	एकै दिन १३ सय जनाको पीसीआर परीक्षण
2020-06-20	Kantipur (Nepali)	एकै दिन ४९४ जना फर्किए
2020-03-18	Kantipur (Nepali)	एक्स्पो सेन्टरलाई कोरोना उपचार केन्द्र बनाइँदै
2020-08-18	Online Khabar (Nepali)	एटीएमबाट पैसा झिकेपछि स्यानिटाइजर अनिवार्य
2020-05-28	Kantipur (Nepali)	एडीबीको २५ करोड डलर सहायता स्वीकृत
2020-07-04	Kantipur (Nepali)	एडीबीको ३० लाख डलर अनुदान
2020-05-15	Kantipur (Nepali)	एनआईसीको १०५ ब्याज छुट
2020-08-28	Kantipur (Nepali)	एनबी बैंकले दियो ५ हजार थान रेमडेसिभिर
2020-05-10	Kantipur (Nepali)	एनबीआईले दियो ३० लाखको पीपीई
2020-05-11	Kantipur (Nepali)	एभरेस्टका कर्मचारीले दिए ३० लाखको राहत
2020-08-30	Kantipur (Nepali)	एभरेस्टका ग्राहकलाई पीसीआरमा छुट
2020-06-05	Kantipur (Nepali)	एयरपोर्टबाट सीधै होल्डिङ सेन्टर
2020-08-31	Kantipur (Nepali)	एयर–फिल्टर मास्क नलगाऔं
2020-06-09	Kantipur (Nepali)	एयरलाइन्स विवाद नमिल्दा विदेशबाट उद्धारमा ढिलाइ
2020-03-15	Kantipur (Nepali)	एसईई केन्द्रमा हेल्थ डेस्क
2020-05-11	Kantipur (Nepali)	एसईई हुन्छ, तयारीमै रहनुहोस् : शिक्षा मन्त्रालय
2020-03-19	Kantipur (Nepali)	एसईईसहित सबै परीक्षा स्थगित
2020-05-06	Kantipur (Nepali)	एसबीआईको १ करोड सहयोग
2020-05-03	Kantipur (Nepali)	एस्कर्टिङ’ मा स्याउ र लसुनका कन्टेनर
2020-07-17	Kantipur (Nepali)	ओपीडी खुले, बिरामी घटे
2020-05-28	Kantipur (Nepali)	ओपीडीबाटै संक्रमण सरे के गर्ने?
2020-06-16	Kantipur (Nepali)	ओमानबाट १६६ जना स्वदेश फिर्ता
2020-06-23	Kantipur (Nepali)	ओम्नीले ल्याएका पीसीआर काम नलाग्ने
2020-06-17	Kantipur (Nepali)	औषधि नै नखाई ७४ लाखको बिल
2020-06-17	Kantipur (Nepali)	कक्षा १० र ११ का परीक्षा
2020-04-29	Kantipur (Nepali)	कक्षा १०–१२ को परीक्षा सञ्चालन गर्न तीन विकल्प
2020-07-06	Kantipur (Nepali)	कक्षा ११ को बोर्ड परीक्षा नहुने
2020-03-23	Kantipur (Nepali)	कञ्चनपुरमा 'कर्फ्यु'
2020-07-11	Kantipur (Nepali)	कतारबाट सिधै क्वारेन्टाइनमा
2020-04-29	Kantipur (Nepali)	'कन्ट्याक्ट ट्रेसिङ धेरै गाह्रो रहेछ'
2020-05-05	Kantipur (Nepali)	कन्ट्याक्ट ट्रेसिङ र परीक्षणबारे अन्योल
2020-05-18	Online Khabar (Nepali)	कपिलवस्तु जिल्ला जेठ २० गतेसम्म सिल
2020-08-06	Online Khabar (Nepali)	कपिलवस्तुमा साउन ३२ गतेसम्म अत्यावश्यक बाहेकका सबै सेवा बन्द
2020-05-12	Online Khabar (Nepali)	कपिलवस्तुलाई जेठ ५ सम्म सिल गर्ने निर्णय
2020-08-03	Kantipur (Nepali)	कम खर्चिलो भएकाले उपयुक्त’
2020-06-23	Kantipur (Nepali)	कमजोर निणर्यले शिक्षा पछाडि
2020-08-10	Kantipur (Nepali)	कमजोर पूर्वतयारीले महामारी तीव्र
2020-02-10	Kantipur (Nepali)	कम्तीमा ७२ घण्टा चाहिन्छ : चीन
2020-06-04	Kantipur (Nepali)	कर कसरी तिर्ने?
2020-06-07	Kantipur (Nepali)	कर तिर्न सकिन्न
2020-08-16	Kantipur (Nepali)	करिब २० प्रतिशतमा लक्षण

Date of publication (yyyy/mm/dd)	Name of news media (select from drop-down menu)	Title of news/headline
2020-05-14	Kantipur (Nepali)	कर्जा असुलीमा अबको जोखिम
2020-05-05	Kantipur (Nepali)	कर्णाली प्रदेश एक साता सिल
2020-06-18	Online Khabar (Nepali)	कर्णालीमा कांग्रेसको प्रस्ताव : सरकारी पदाधिकारीको गाडी कोरोना नियन्त्रणमा प्रयोग गरौं
2020-04-07	Online Khabar (Nepali)	कर्णालीमा कोरोनाको जोखिम : लकडाउनले नपुग्ने भन्दै कोरियन मोडेलको खोजी
2020-05-17	Kantipur (Nepali)	कर्णालीमा खेती अभियान सुरु
2020-08-14	Online Khabar (Nepali)	कर्णालीमा प्रवेश गर्न पीसीआर अनिवार्य, होटल रेस्टुरेन्ट बन्द
2020-07-17	Kantipur (Nepali)	कर्णालीमा मेसिन धेरै, परीक्षण थोरै
2020-03-20	Online Khabar (Nepali)	कर्णालीमा हजार बेडको क्वारेन्टाइन, नाकाबाट नेपालीलाई मात्रै प्रवेश
2020-06-22	Kantipur (Nepali)	कर्मचारी कटौतीको तयारी
2020-05-05	Kantipur (Nepali)	कर्मचारी भर्ना बन्द
2020-05-29	Kantipur (Nepali)	कर्मचारीको भत्ता कटौती
2020-08-16	Online Khabar (Nepali)	कर्मचारीमा कोरोना देखिएपछि बुटवल उपमहानगरमा अत्यावश्यकबाहेकका सेवा बन्द
2020-07-30	Online Khabar (Nepali)	कर्मचारीमा कोरोना संक्रमण देखिएपछि जलेश्वर नगरपालिका बन्द
2020-05-25	Kantipur (Nepali)	कर्मचारीलाई ५० प्रतिशत तलब देऊ’
2020-06-15	Kantipur (Nepali)	कस्तो आउँदै छ प्रदेश बजेट ?
2020-05-11	Kantipur (Nepali)	कस्तो छ डिजिटल पूर्वाधार?
2020-06-09	Kantipur (Nepali)	कहाँ चुक्यो सरकार?
2020-08-09	Kantipur (Nepali)	कहिले हुन्छ दिनहुँ १० हजार परीक्षण?
2020-07-07	Kantipur (Nepali)	कांग्रेस महाधिवेशन फागुन ७–१० मै
2020-03-03	Kantipur (Nepali)	कांग्रेसको १४ बुँदे अपिल
2020-08-19	Online Khabar (Nepali)	काठमाडौं उपत्यकामा बुधबार मध्यरातदेखि निषेधाज्ञा
2020-08-14	Online Khabar (Nepali)	काठमाडौं उपत्यकामा भेला, प्रदर्शन र जात्रा सञ्चालनमा रोक
2020-07-18	Kantipur (Nepali)	काठमाडौं क्वारेन्टाइन
2020-07-30	Online Khabar (Nepali)	काठमाडौं प्रवेश गर्ने नाकामा स्वाब दिएकामध्ये २४ जनामा कोरोना पुष्टि
2020-08-14	Kantipur (Nepali)	काठमाडौं भित्रिने राजमार्गमा कडाइ
2020-08-13	Online Khabar (Nepali)	काठमाडौं महानगर वडा नम्बर १४ मा फलफूल तथा तरकारी पसल चार घण्टा मात्र खोल्न पाइने
2020-06-21	Kantipur (Nepali)	काठमाडौं संक्रमणको जोखिममा
2020-08-16	Online Khabar (Nepali)	काठमाडौं–१० मा फुटपाथ पसल र चियापसल बन्द
2020-08-12	Online Khabar (Nepali)	काठमाडौंका पसलहरुमा अपनाइँदैन सावधानी
2020-08-07	Online Khabar (Nepali)	काठमाडौंका सार्वजनिक यातायातमा छैन सुरक्षा सावधानी
2020-08-08	Online Khabar (Nepali)	काठमाडौंको गहनापोखरी–हाँडीगाउँ क्षेत्र सिल
2020-05-15	Online Khabar (Nepali)	काठमाडौंको चन्द्रागिरि नगरपालिका ‘सिल’
2020-04-17	Online Khabar (Nepali)	काठमाडौंबाट ३३ चिकित्सकको टोली सुदूरपश्चिम पुग्यो
2020-05-06	Kantipur (Nepali)	काठमाडौंमा कति जनाको भयो कोरोना परीक्षण?
2020-08-26	Online Khabar (Nepali)	काठमाडौंमा कौसीमै नाचियो गौरा (फोटो फिचर)
2020-05-21	Kantipur (Nepali)	काठमाडौंमा छैन क्वारेन्टाइन
2020-08-22	Online Khabar (Nepali)	काठमाडौंमा संक्रमितको संख्या बढेपछि...
2020-08-18	Kantipur (Nepali)	काठमाडौंलाई जोगाऊ
2020-08-21	Online Khabar (Nepali)	काभ्रेमा सार्वजनिक यातायात बन्द
2020-05-30	Kantipur (Nepali)	कामदार ल्याउन नेपाललाई खर्च नलाग्ने
2020-03-19	Kantipur (Nepali)	कामदारलाई छुट्टी दिँदै होटल

Date of publication (yyyy/mm/dd)	Name of news media (select from drop-down menu)	Title of news/headline
2020-08-16	Kantipur (Nepali)	कामदारलाई स्वदेश फर्काउने प्रक्रिया सुस्त
2020-08-30	Kantipur (Nepali)	कामनाको पनि छुट योजना
2020-08-24	Online Khabar (Nepali)	काममा नजाने वीरगञ्जका निजी अस्पतालका डाक्टरको निर्णय
2020-03-20	Kantipur (Nepali)	कारागारमा उच्च जोखिम
2020-03-17	Kantipur (Nepali)	कार्यान्वयन फितलो
2020-06-17	Kantipur (Nepali)	कार्यान्वयनको कसीमा प्रदेश बजेट
2020-04-07	Online Khabar (Nepali)	कालिकोटबाट 'थ्रोट स्वाब' लिएर फर्किए मन्त्री
2020-05-20	Online Khabar (Nepali)	कालीमाटीका सुरक्षाकर्मीमा संक्रमण देखिएपछि प्रहरीका ६ इकाइ सिल
2020-05-11	Kantipur (Nepali)	कालोबजारी आरोपमा दुई पक्राउ
2020-03-06	Kantipur (Nepali)	कालोबजारीविरुद्ध खबरदारी गर्ने
2020-05-03	Online Khabar (Nepali)	किट अभावले जुम्लामा सुरु भएन कोरोना परीक्षण
2020-05-03	Online Khabar (Nepali)	किट अभावले भैरहवामा कोरोना परीक्षण रोकियो
2020-06-13	Kantipur (Nepali)	किन उत्रिए युवा सडकमा ?
2020-07-07	Kantipur (Nepali)	किसानलाई १० करोड राहत दिइँदै
2020-07-14	Kantipur (Nepali)	किस्ताको दबाबले व्यवसायी आन्दोलित
2020-08-01	Kantipur (Nepali)	कीर्तिपुर मैदानमै आवासीय सुविधा, बाहिर जान र आउन बन्देज
2020-06-11	Kantipur (Nepali)	कुखुराको मासु अभाव
2020-06-29	Kantipur (Nepali)	कुन चरणमा पुग्यो कोरोना संक्रमण?
2020-06-18	Kantipur (Nepali)	कुवेतमा ५३ दिनदेखि जहाज कुर्दै २३९३ नेपाली
2020-03-17	Kantipur (Nepali)	कूटनीतिक ब्रिफिङ
2020-05-11	Kantipur (Nepali)	कूटनीतिक वार्ता कहिले ?
2020-06-23	Kantipur (Nepali)	कृषकलाई होइन, 'प्रपोजल' लाई अनुदान !
2020-05-13	Kantipur (Nepali)	कृषि उत्पादन बढाऊ'
2020-06-15	Kantipur (Nepali)	केएमजीका सबै कर्मचारीको कोरोना बिमा
2020-06-03	Online Khabar (Nepali)	केएमसी अस्पतालको आकस्मिकसहित सबै सेवा बन्द
2020-04-05	Online Khabar (Nepali)	कैलाली, कञ्चनपुर र बागलुङमा क–कसको हुने भयो कोरोना परीक्षण ?
2020-04-21	Online Khabar (Nepali)	कैलालीका एक युवकमा कोरोना एन्टिबडी पोजेटिभ
2020-05-30	Online Khabar (Nepali)	कैलालीका सात स्थानीय तह मिलेर कोरोना अस्पताल सञ्चालन गर्दै
2020-05-21	Online Khabar (Nepali)	कैलालीमा भारतीय भूमिबाट साढे ८ सय नेपालीको उद्धार
2020-08-10	Online Khabar (Nepali)	कोटेश्वरका थप ४ जनालाई कोरोना संक्रमण
2020-08-10	Online Khabar (Nepali)	कोटेश्वरको 'कोरोना संक्रमित क्षेत्र'मा चिहाउँदा...
2020-07-30	Online Khabar (Nepali)	कोभिड १९ उपचार गरेबापत निजी अस्पतालले १५ हजारसम्म शुल्क लिन पाउने
2020-08-14	Online Khabar (Nepali)	कोभिड १९ का बिरामीको उपचारका लागि जनमैत्री अस्पताल सञ्चालनमा ल्याउन माग
2020-08-17	Online Khabar (Nepali)	कोभिड कोषमा बेलायतबाट व्यवसायी लामिछानेको १५ लाख सहयोग
2020-07-30	Online Khabar (Nepali)	कोभिड नियन्त्रणमा नखटिने २५ स्वास्थ्यकर्मी पक्राउ
2020-06-14	Kantipur (Nepali)	कोभिड–१९ : चुनौती र सम्भावना सँगसँगै
2020-03-24	Online Khabar (Nepali)	कोभिड–१९ : हालसम्म नेपालमा ६१० नमूना परीक्षण, दुईमा संक्रमण
2020-07-08	Kantipur (Nepali)	कोभिड-१९ को पर्भाव र नेपाली क्रिकेट
2020-06-07	Kantipur (Nepali)	कोभिड–१९ पछि विद्यालय शिक्षा कसरी?
2020-06-04	Kantipur (Nepali)	कोभिड–१९ पछिको सहरी विकास
2020-06-12	Kantipur (Nepali)	कोभिड–१९ बाट आँखाको सुरक्षा
2020-04-24	Kantipur (Nepali)	कोभिड–१९ विरुद्ध सरकार कति सफल?

Date of publication (yyyy/mm/dd)	Name of news media (select from drop-down menu)	Title of news/headline
2020-08-30	Kantipur (Nepali)	कोभिडका कारण आम्दानी गुमाउँदै टेलिकम क्षेत्र
2020-05-02	Kantipur (Nepali)	कोभिडको पटाक्षेप र चाँदीका घेरा
2020-07-20	Kantipur (Nepali)	कोभिडले शिथिल आर्थतन्त्रमा मौर्दिक नीतिले राहत
2020-05-16	Online Khabar (Nepali)	कोरोना अपडेट : ९२ हजारमा परीक्षण, क्वारेन्टाइनमा १६३६६ जना
2020-03-07	Kantipur (Nepali)	कोरोना असर मास्क उद्योगमा पनि
2020-06-17	Kantipur (Nepali)	कोरोना अस्थायी अस्पताल बन्द
2020-04-02	Online Khabar (Nepali)	कोरोना अस्पतालका डाक्टरको पीडा : घरबेटीले घर नआऊ भने, होटेलमा बसेको छु
2020-06-21	Kantipur (Nepali)	कोरोना अस्पतालले संक्रमितलाई सुत्केरी गराउन मानेन
2020-05-06	Kantipur (Nepali)	कोरोना आयो, मानवता हरायो'
2020-04-26	Online Khabar (Nepali)	कोरोना उपचार कोषमा शेष घले र जमुना गुरुङको ३ करोड सहयोग
2020-08-20	Online Khabar (Nepali)	कोरोना उपचारमा बेड छुट्याउनुपर्ने निर्णय सच्याऊ : चिकित्सक संघ
2020-08-13	Kantipur (Nepali)	कोरोना उपचारमा रेमडेसिभिर प्रयोग गरिने
2020-07-21	Online Khabar (Nepali)	कोरोना उपचारवापत ८ निजी अस्पतालले लिए २३ लाख भत्ता
2020-02-16	Kantipur (Nepali)	कोरोना कहर
2020-08-17	Online Khabar (Nepali)	कोरोना कहर : बाँकेमा सुकुम्बासी बस्तीका ८५ घरपरिवार भोकभोकै
2020-05-31	Online Khabar (Nepali)	कोरोना कोषको रकम खर्च गर्न थालियो, दुई दर्जन जिल्लामा पठाइए बजेट
2020-06-16	Kantipur (Nepali)	कोरोना कोषबाट २४ करोड निकासा
2020-06-01	Kantipur (Nepali)	कोरोना कोषबाट खर्च गर्न सुरु
2020-08-19	Kantipur (Nepali)	कोरोना कोषमा एनआईसीको १ करोड
2020-08-14	Kantipur (Nepali)	कोरोना कोषमा एनएमबी लघुवित्तको १३ लाख
2020-05-25	Kantipur (Nepali)	कोरोना कोषमा एनबी बैंकको डेढ करोड
2020-06-11	Kantipur (Nepali)	कोरोना कोषमा नबिलको २६ लाख
2020-08-17	Kantipur (Nepali)	कोरोना कोषमा नबिलको ६ करोड
2020-08-15	Kantipur (Nepali)	कोरोना कोषमा सनराइजको ७९ लाख
2020-08-20	Kantipur (Nepali)	कोरोना कोषमा सिटिजन्सको २० लाख
2020-08-13	Kantipur (Nepali)	कोरोना कोषमा सिद्धार्थको २ करोड
2020-08-18	Kantipur (Nepali)	कोरोना कोषमा स्वावलम्बनको १ करोड
2020-06-13	Kantipur (Nepali)	कोरोना खर्चमा लेखाको छानबिन
2020-03-18	Kantipur (Nepali)	कोरोना जोखिम व्यवस्थापन समिति
2020-05-23	Online Khabar (Nepali)	कोरोना नभएकालाई मात्रै नाकाबाट प्रवेश दिने व्यवस्था गर्न कर्णाली सरकारको माग
2020-02-04	Kantipur (Nepali)	कोरोना नमस्ते
2020-05-28	Kantipur (Nepali)	कोरोना नियन्त्रण र पुराना कार्यक्रममा गण्डकीको जोड
2020-08-22	Kantipur (Nepali)	कोरोना नियन्त्रणमा १२ अर्ब खर्च
2020-07-29	Kantipur (Nepali)	कोरोना नियन्त्रणमा कहाँ चुक्यो सरकार ?
2020-06-03	Online Khabar (Nepali)	कोरोना नियन्त्रणमा खटिएकालाई दुर्व्यवहार गर्नेलाई पक्राउ गर्न गृहको निर्देशन
2020-05-07	Kantipur (Nepali)	कोरोना नियन्त्रणमा चुक्दै सरकार
2020-05-03	Kantipur (Nepali)	कोरोना नियन्त्रणमा प्रदेशको पहल
2020-08-27	Online Khabar (Nepali)	कोरोना नियन्त्रणमा प्रदेशले दिएको रकम खर्च भएन : मुख्यमन्त्री राउत
2020-04-08	Online Khabar (Nepali)	कोरोना परीक्षण गर्न तीन जिल्लाबाट ८०२ नमुना संकलन
2020-04-24	Online Khabar (Nepali)	कोरोना परीक्षण गर्न सबै पालिकामा स्वास्थ्यकर्मीको टोली

Date of publication (yyyy/mm/dd)	Name of news media (select from drop-down menu)	Title of news/headline
2020-04-04	Online Khabar (Nepali)	कोरोना परीक्षण बढाउन कांग्रेसको माग
2020-08-31	Online Khabar (Nepali)	कोरोना परीक्षण शुल्क अब ४ हजार मात्र
2020-04-20	Online Khabar (Nepali)	कोरोना परीक्षणको दायरा समुदाय स्तरसम्म विस्तार गर्नुपर्छ : देउवा
2020-08-02	Online Khabar (Nepali)	कोरोना परीक्षणको मापदण्ड परिवर्तन, झाडा पखालाका विरामीको पनि टेष्ट गरिने
2020-04-18	Online Khabar (Nepali)	कोरोना पोजेटिभको स्वाब काठमाडौं पठाउँदाको सास्ती
2020-02-28	Kantipur (Nepali)	कोरोना प्रभावको आर्थिक सतर्कता
2020-05-24	Online Khabar (Nepali)	कोरोना प्रभावबारे जानकारी लिन सर्वेक्षण
2020-02-27	Kantipur (Nepali)	कोरोना प्रभावले औषधिको कच्चा पदार्थ अभाव
2020-03-10	Kantipur (Nepali)	कोरोना प्रभावित देशबाट हुने आयातमा कडाइ गरिँदै
2020-06-10	Kantipur (Nepali)	कोरोना प्राथमिकतामा परेन
2020-03-12	Kantipur (Nepali)	कोरोना फोबिया
2020-02-26	Kantipur (Nepali)	कोरोना बारेको गम्भीरता
2020-08-24	Kantipur (Nepali)	कोरोना बिमा गर्ने साढे ७ लाख पुगे
2020-06-04	Kantipur (Nepali)	कोरोना बिमाको मोडालिटी परिमार्जन गरिँदै
2020-06-14	Kantipur (Nepali)	कोरोना बिमामा दाबी पर्न थाल्यो
2020-06-28	Kantipur (Nepali)	कोरोना भगाउने जन्तर
2020-02-24	Kantipur (Nepali)	कोरोना भाइरस र असंवेदनशील हामी
2020-02-02	Kantipur (Nepali)	कोरोना भाइरस र सजगता
2020-02-01	Online Khabar (Nepali)	कोरोना भाइरसको असर चितवनको पर्यटन व्यवसायमा
2020-01-29	Online Khabar (Nepali)	कोरोना भाइरसको डर, मास्कले छोपिए अनुहार
2020-02-10	Kantipur (Nepali)	कोरोना भाइरसबाट कसरी बच्ने?
2020-03-04	Kantipur (Nepali)	कोरोना भित्रिन नदिन सतर्कता
2020-06-09	Kantipur (Nepali)	कोरोना महामारी र सर्वोच्च अदालतको चासो
2020-08-06	Online Khabar (Nepali)	कोरोना महामारीमा चाडपर्व मनाउन सरकारले ल्यायो निर्देशिका
2020-07-07	Online Khabar (Nepali)	कोरोना महामारीमा सक्रिय स्वास्थ्यकर्मी र सफाइकर्मी सम्मानित
2020-08-24	Online Khabar (Nepali)	कोरोना महामारीमा स्थानीय तरकारीले बजार पायो
2020-08-23	Online Khabar (Nepali)	कोरोना महामारीलाई गिज्याउने बल्खु बजारको दृश्य
2020-08-28	Online Khabar (Nepali)	कोरोना र बाढी प्रभावित ५९७ परिवारलाई खाद्यान्न
2020-05-28	Online Khabar (Nepali)	कोरोना राहत प्याकेज : उद्योगी–कामदारलाई के दियो बजेटले ?
2020-05-07	Online Khabar (Nepali)	कोरोना रिपोर्ट नदेखाई संसद बैठकमा प्रवेश निषेध
2020-06-14	Kantipur (Nepali)	कोरोना रोकथाम र नियन्त्रणमा ८ अर्ब ३९ करोड खर्च
2020-08-09	Online Khabar (Nepali)	कोरोना रोकथामका लागि प्रदेश २ ले संघीय सरकारसँग माग्यो विशेष प्याकेज
2020-06-25	Kantipur (Nepali)	कोरोना रोकथामकै वरिपरि
2020-06-10	Online Khabar (Nepali)	कोरोना रोकथाममा अन्य प्रदेश भन्दा उत्कृष्ट छौं : मुख्यमन्त्री राई
2020-03-27	Online Khabar (Nepali)	कोरोना रोकथाममा प्रदेश २ को तयारी : पैसा छ, उपकरण छैन
2020-02-18	Kantipur (Nepali)	कोरोना रोकथाममा मास्कको प्रभावकारिता
2020-05-02	Online Khabar (Nepali)	कोरोना रोकथाममा सार्क राष्ट्रमा नेपालको अवस्था राम्रो
2020-05-22	Kantipur (Nepali)	कोरोना रोकथाममा स्थानीय तह
2020-03-14	Kantipur (Nepali)	कोरोना रोक्न प्रशिक्षण
2020-04-05	Online Khabar (Nepali)	कोरोना र्‍यापिड टेस्ट : सुरुमा ३००, त्यसपछि २० हजार !
2020-05-20	Kantipur (Nepali)	कोरोना संकटको वेक–अप कल आइसकेको छ'
2020-02-06	Kantipur (Nepali)	कोरोना संक्रमण ख्यालठट्टा होइन

Date of publication (yyyy/mm/dd)	Name of news media (select from drop-down menu)	Title of news/headline
2020-04-12	Online Khabar (Nepali)	कोरोना संक्रमण देखिएको ठाउँलाई पूर्ण लकडाउन गर्छौं : मुख्यमन्त्री राउत
2020-04-28	Online Khabar (Nepali)	कोरोना संक्रमण देखिएपछि रौतहटका दुई गाउँ सिल
2020-08-19	Online Khabar (Nepali)	कोरोना संक्रमणले चितवनका अस्पतालको सेवा खुम्चियो
2020-03-28	Online Khabar (Nepali)	कोरोना संक्रमित किशोरीको परिवारको नमुना नै संकलन गरिएन
2020-05-04	Online Khabar (Nepali)	कोरोना संक्रमित क्षेत्रमा पुगे प्रदेशमन्त्री
2020-05-16	Online Khabar (Nepali)	कोरोना संक्रमित परिवारको पीडा : आत्मबल बढाउनुको साटो खुच्चिङ भने
2020-05-20	Online Khabar (Nepali)	कोरोना संक्रमित भेटिएपछि भक्तपुरको ल्योसिखेल सिल
2020-05-20	Online Khabar (Nepali)	कोरोना संक्रमित भेटिएपछि मैतीदेवी क्षेत्र सिल
2020-05-02	Online Khabar (Nepali)	कोरोना संक्रमित भेटिएपछि रुपन्देही र बाँकेका चार स्थानमा आउ-जाउ बन्द
2020-05-14	Online Khabar (Nepali)	कोरोना संक्रमित महिला केही घण्टा बसेको कैलालीको एक टोललाई सिल गरियो
2020-08-23	Online Khabar (Nepali)	कोरोना संक्रमितका लागि मनमोहनको ३३३ शय्याको अस्पताल
2020-07-30	Online Khabar (Nepali)	कोरोना संक्रमितको उचित व्यवस्थापन गर्न माग गर्दै अदालतमा उजुरी
2020-04-26	Online Khabar (Nepali)	कोरोना संक्रमितको उपचार गर्न धरानमा बन्यो सुविधासम्पन्न अस्पताल
2020-07-01	Online Khabar (Nepali)	कोरोना संक्रमितको उपचार गर्ने अस्पतालले सरकारबाट अनुदान पाउने
2020-08-13	Online Khabar (Nepali)	कोरोना संक्रमितको औषधिमा कालोबजारी !
2020-04-09	Online Khabar (Nepali)	कोरोना संक्रमितको कोठामै छिर्दैनन् डाक्टर !
2020-08-17	Online Khabar (Nepali)	कोरोना संक्रमितको घरमा छिमेकीको ताला !
2020-08-20	Online Khabar (Nepali)	कोरोना संक्रमितको मनोबल बढाउन बुटवलमा 'होम भिजिट' अभियान
2020-08-19	Online Khabar (Nepali)	कोरोना संक्रमितलाई अण्डा र मासु
2020-03-08	Kantipur (Nepali)	कोरोना संक्रमितलाई छैन आईसीयू
2020-07-05	Kantipur (Nepali)	कोरोना सचेतना दौड
2020-05-20	Kantipur (Nepali)	कोरोनाइतर स्वास्थ्य समस्या सतहमा
2020-06-02	Kantipur (Nepali)	कोरोना–कहरमा राज्यका चुनौती
2020-04-08	Online Khabar (Nepali)	कोरोनाका कारण कसरी बदलिँदैछ नेपाली संस्कार ?
2020-03-12	Online Khabar (Nepali)	कोरोनाका कारण संसद बैठक रोक्न कांग्रेस सांसदको माग
2020-05-02	Online Khabar (Nepali)	कोरोनाका नक्कली बिरामी अस्पतालमा आउँदा…
2020-06-11	Kantipur (Nepali)	कोरोनाका नाममा अपारदर्शी खर्च
2020-05-14	Kantipur (Nepali)	कोरोनाका भ्रम निराधार
2020-08-18	Kantipur (Nepali)	कोरोनाका मृतकको अन्त्येष्टि
2020-08-18	Kantipur (Nepali)	कोरोनाकालका किताब
2020-08-09	Kantipur (Nepali)	कोरोनाकालमा खान्की
2020-06-01	Kantipur (Nepali)	कोरोनाकालमा जुठोचोखो
2020-07-04	Kantipur (Nepali)	कोरोनाकालमा 'प्लेग' पढ्नू !
2020-05-24	Kantipur (Nepali)	कोरोनाकालमा स्थानीय तह
2020-05-05	Kantipur (Nepali)	कोरोनाको आलोकमा हाम्रो युग
2020-05-14	Kantipur (Nepali)	कोरोनाको चेपमा लिपुलेक
2020-07-31	Online Khabar (Nepali)	कोरोनाको नयाँ हटस्पट बन्न सक्छ काठमाडौं
2020-02-12	Kantipur (Nepali)	कोरोनाको भूराजनीतिक असर
2020-06-16	Kantipur (Nepali)	कोरोनाको मारमा श्रमिक
2020-07-13	Kantipur (Nepali)	कोरोनाको व्यवस्थापन कमजोर
2020-05-05	Online Khabar (Nepali)	कोरोनाको हटस्पट भुल्के : त्रास कम तर जोखिम उस्तै

Date of publication (yyyy/mm/dd)	Name of news media (select from drop-down menu)	Title of news/headline
2020-03-09	Kantipur (Nepali)	कोरोनाको हल्लाले खाद्य संकट
2020-05-31	Kantipur (Nepali)	कोरोना–त्रासबीच गाउँले जगाएको आशा
2020-05-10	Online Khabar (Nepali)	कोरोनापछि मिटर घुमे जसरी बढ्दैछ बेरोजगारी
2020-05-13	Kantipur (Nepali)	कोरोनापीडित ऋणीलाई मात्र ब्याज छुट
2020-04-28	Kantipur (Nepali)	कोरोनाबाट जोगिन काठमाडौं !
2020-03-07	Kantipur (Nepali)	कोरोनाबाट बच्ने उपाय
2020-06-02	Online Khabar (Nepali)	कोरोनाबाट मृत्यु हुनेको परिवारलाई प्रदेश सरकारले एक लाख दिने
2020-04-15	Online Khabar (Nepali)	कोरोनाबारे अपवाह फैलाउने पक्राउ
2020-08-10	Online Khabar (Nepali)	कोरोनाबारे जनचेतना जगाउँदै पर्यटक प्रहरी
2020-06-09	Online Khabar (Nepali)	कोरोनाबारे सरकारलाई कांग्रेसको पाँच प्रश्न
2020-03-10	Kantipur (Nepali)	कोरोनाबारे सूचनामै संक्रमण
2020-07-13	Kantipur (Nepali)	कोरोनाभन्दा मान्छेको डर !'
2020-04-29	Kantipur (Nepali)	कोरोनाभन्दा समाजको डर
2020-08-21	Online Khabar (Nepali)	कोरोनामा १३ अर्ब ३७ करोड खर्च, धेरै खर्च रक्षा मन्त्रालयबाट
2020-02-15	Kantipur (Nepali)	कोरोनामाथि जितपछि आयोजनामा काम ः राजदूत होउ
2020-05-20	Kantipur (Nepali)	कोरोनामुक्त नेपाल पहिलो प्राथमिकता : प्रधानमन्त्री
2020-06-14	Kantipur (Nepali)	कोरोनाले खोस्दै सुनीलको पढ्ने सपना
2020-08-20	Kantipur (Nepali)	कोरोनाले खोस्यो चाडको रौनक
2020-08-10	Kantipur (Nepali)	कोरोनाले मृत्यु भएकामध्ये ५७.३ प्रतिशत दीर्घरोगी
2020-05-18	Kantipur (Nepali)	कोरोनाले मृत्यु भएकामा फरक–फरक लक्षण
2020-06-28	Kantipur (Nepali)	कोरोनाले रोकेन असारे विकास
2020-08-06	Kantipur (Nepali)	कोरोनाले ल्याएको अवसर
2020-05-28	Kantipur (Nepali)	कोरोनाले सरकारलाई छोएन
2020-02-13	Kantipur (Nepali)	कोरोनाविरुद्घ प्लान बी र सी तयार'
2020-03-03	Kantipur (Nepali)	कोरोनाविरुद्ध उच्च सतर्कता माग
2020-05-09	Kantipur (Nepali)	कोरोनाविरुद्ध एक ठाउँ
2020-03-07	Kantipur (Nepali)	कोरोनाविरुद्ध एकीकृत योजना माग
2020-02-08	Kantipur (Nepali)	कोरोनाविरुद्ध एन्टिबायोटिकले काम गर्दैन
2020-03-23	Online Khabar (Nepali)	कोरोनाविरुद्ध लड्न एनआरएनले नेपाल सरकारलाई एक करोड सहयोग गर्ने
2020-05-24	Online Khabar (Nepali)	कोरोनाविरुद्ध लड्न वीरगञ्ज महानगरले बाँड्यो चेयवनप्रास
2020-07-17	Kantipur (Nepali)	कोरोनाविरुद्ध लड्न सूर्य नेपालको ७ करोड
	Kantipur (Nepali)	कोरोनाविरुद्ध सतर्कता
2020-02-20	Kantipur (Nepali)	कोरोनाविरुद्धको संघर्षमा ऐक्यबद्धता
2020-06-22	Kantipur (Nepali)	कोरोनासँग जुध्न दिनहुँ योग
2020-06-07	Kantipur (Nepali)	कोरोनासँग जुध्न बेलायतको थप सहयोग
2020-06-06	Kantipur (Nepali)	कोषमा महासंघको २७ लाख
2020-05-09	Kantipur (Nepali)	कोसीबाट ८ जना डिस्चार्ज
2020-06-01	Kantipur (Nepali)	क्वारेन्टाइन नै आइसोलेसन
2020-08-28	Kantipur (Nepali)	क्वारेन्टाइन बनाउन २० शय्या सहयोग
2020-04-02	Online Khabar (Nepali)	क्वारेन्टाइन बस्नेको कथा : आफन्तले भेट्न मान्दैनन्, फोनमा हालखबर सोध्छन्
2020-02-07	Kantipur (Nepali)	क्वारेन्टाइनका लागि चार ठाउँ छान्न निर्देशन
2020-02-09	Kantipur (Nepali)	क्वारेन्टाइनको संरचना बन्दै

Date of publication (yyyy/mm/dd)	Name of news media (select from drop-down menu)	Title of news/headline
2020-04-26	Kantipur (Nepali)	क्वारेन्टाइनको सट्टा होम क्वारेन्टाइनले जोखिम
2020-03-03	Kantipur (Nepali)	क्वारेन्टाइनबाट घर फिर्ता
2020-05-04	Kantipur (Nepali)	क्वारेन्टाइनबाट भागे १८ भारतीय
2020-05-24	Kantipur (Nepali)	क्वारेन्टाइनबाटै संक्रमित भागाभाग
2020-06-09	Kantipur (Nepali)	'क्वारेन्टाइनबारे सीडीओले सही रिपोर्टिङ गर्दैनन्'
2020-03-01	Kantipur (Nepali)	क्वारेन्टाइनबिनै कार्यक्षेत्रमा चिनियाँ
2020-02-19	Kantipur (Nepali)	क्वारेन्टाइनमा 'संवेदनशीलता अपनाइएन'
2020-06-02	Kantipur (Nepali)	क्वारेन्टाइनमा कोचाकोचले संक्रमणको जोखिम
2020-06-08	Kantipur (Nepali)	क्वारेन्टाइनमा चरम लापरवाही
2020-06-23	Kantipur (Nepali)	क्वारेन्टाइनमा धेरै खर्च, त्यसमै बिजोग
2020-05-19	Kantipur (Nepali)	क्वारेन्टाइनमा भद्रगोल
2020-04-19	Online Khabar (Nepali)	क्वारेन्टाइनमा राखिएकाहरूलाई वडाध्यक्षले छाडिदिए
2020-02-02	Kantipur (Nepali)	क्वारेन्टाइनमा राख्न ठाउँ खोजिँदै
2020-02-18	Kantipur (Nepali)	क्वारेन्टाइनमा र्‍यालको नमुना लिइयो
2020-04-29	Kantipur (Nepali)	क्वारेन्टाइनमा वृद्धको मृत्यु
2020-02-17	Kantipur (Nepali)	क्वारेन्टाइनमा स्यान्डविच र दाल–भात
2020-05-10	Kantipur (Nepali)	क्वारेन्टाइनमै जन्मिए जुम्ल्याहा
2020-05-19	Kantipur (Nepali)	क्वारेन्टाइनमै मृत्यु हुनु चरम लापरबाही : विज्ञ
2020-06-15	Kantipur (Nepali)	क्वारेन्टाइनमै 'सामूहिक बलात्कार' तीन स्वयंसेवक पक्राउ
2020-05-20	Kantipur (Nepali)	क्वारेन्टाइनहरू नाम मात्रका
2020-06-16	Kantipur (Nepali)	क्वारेन्टिन' लाई सुरक्षित बनाउने दायित्व कसको?
2020-06-16	Kantipur (Nepali)	क्वारेन्टिनको औचित्यमाथि प्रश्न
2020-06-11	Kantipur (Nepali)	क्वारेन्टिनको कारुणिक कहानी
2020-06-28	Kantipur (Nepali)	क्वारेन्टिनमा अनुगमन खै?
2020-08-10	Online Khabar (Nepali)	क्वारेन्टिनमा आत्महत्या : विभेद र गरीबी मुख्य कारण
2020-07-28	Online Khabar (Nepali)	क्वारेन्टिनमा आत्महत्या गरेका पुरुषमा कोरोना पुष्टि
2020-05-21	Kantipur (Nepali)	क्वारेन्टिनलाई सुरक्षित बनाऊ
2020-06-10	Kantipur (Nepali)	खपत घट्दा किसानले मूल्य पाएनन्
2020-02-05	Kantipur (Nepali)	खरिपाटीमा 'आइसोलेसन सेन्टर'
2020-02-08	Kantipur (Nepali)	खरिपाटीमा भवन खाली गरिँदै
2020-07-30	Kantipur (Nepali)	खर्च घटाउने उपाय खोज्दै बैंक
2020-03-01	Kantipur (Nepali)	खाडीमा कोरोना संक्रमण बढ्दो नेपालीलाई सतर्क रहन आग्रह
2020-08-12	Kantipur (Nepali)	खाने पेट थपिए, अन्नको गेडो छैन
2020-05-05	Kantipur (Nepali)	खाली ठाउँमा खेती गरे अनुदान
2020-08-14	Kantipur (Nepali)	खाली हुन थाले उपत्यकाका पसल
2020-08-23	Kantipur (Nepali)	खाली हुन थाले सटर
2020-06-03	Kantipur (Nepali)	खाल्डोमा तरकारी, बजारमा अभाव
2020-05-14	Kantipur (Nepali)	खुम्चियो बैंकहरूको नाफा
2020-06-05	Kantipur (Nepali)	खुल्न थाले पसल
2020-05-02	Kantipur (Nepali)	खोइ के बन्द छ यहाँ?
2020-06-10	Kantipur (Nepali)	खोइ खर्च विवरण?
2020-04-28	Online Khabar (Nepali)	गण्डकमा कोभिड–१९ अस्पताल सञ्चालन अनुमति दिन वीरगञ्ज महानगरको माग

Date of publication (yyyy/mm/dd)	Name of news media (select from drop-down menu)	Title of news/headline
2020-05-14	Online Khabar (Nepali)	गण्डकीका मन्त्री भन्छन्- नेपालीलाई सीमामा परीक्षण गरेर ल्याउनुपर्छ
2020-08-17	Online Khabar (Nepali)	गण्डकीमा धमाधम निषेधाज्ञा र लकडाउन
2020-07-15	Kantipur (Nepali)	गण्डकीलाई सूर्य नेपालको एक करोड
2020-07-06	Kantipur (Nepali)	गर्भवती र सुत्केरीलाई विशेष ध्यान दिनुपर्छ’
2020-08-18	Kantipur (Nepali)	गर्भवतीको शल्यक्रियामा सकस
2020-03-07	Kantipur (Nepali)	गर्मीमा कोरोना संक्रमणको जोखिम कम
2020-06-14	Kantipur (Nepali)	गल्याङ एक साता बन्द
2020-05-28	Online Khabar (Nepali)	गाइडलाइन नहुँदा नारायणीबाट जनरल सेवा सञ्चालन गर्न समस्या
2020-07-02	Kantipur (Nepali)	गाउँका लागि सहर
2020-05-13	Online Khabar (Nepali)	गाउँपालिका अध्यक्षको विज्ञप्ति : बर्दियाको कोरोना संक्रमित दाङको अस्पतालमा नल्याऊ
2020-05-10	Kantipur (Nepali)	गाडी चलाउन समयसीमा
2020-04-25	Kantipur (Nepali)	गाडीबाट भित्रिएका सीधै घरमा
2020-08-12	Kantipur (Nepali)	गाह्रो भयो भने अस्पताल जान सक्नुहुन्छ, बेड व्यवस्था हुन्छ
2020-02-14	Kantipur (Nepali)	गुम्बा गाउँमा नमुना संकलन
2020-05-26	Online Khabar (Nepali)	गुल्मीमा गाउँपालिका अध्यक्षलाई कोरोना संक्रमण
2020-08-20	Online Khabar (Nepali)	गुल्मीमा शुक्रबार रातिदेखि निषेधाज्ञा
2020-07-17	Online Khabar (Nepali)	गैंडाकोट नगरपालिका एकसाताका लागि सिल
2020-08-16	Online Khabar (Nepali)	गोदाममै थन्कियो भेन्टिलेटर, घरमै मर्न थाले संक्रमित
2020-05-26	Online Khabar (Nepali)	गौरीफन्टा नाकाबाट एकैदिन ५०१३ नेपाली घर फर्किए
2020-08-24	Kantipur (Nepali)	घटेन पीसीआर शुल्क
2020-04-26	Kantipur (Nepali)	घट्यो औषधि उत्पादन र बिक्री
2020-05-05	Kantipur (Nepali)	घर फर्कने कामदारका पक्षमा
2020-08-07	Kantipur (Nepali)	घरकै आइसोलेसनले मनोबल उच्च
2020-03-07	Kantipur (Nepali)	घरघरमा जनचेतना जगाउन निर्देशन
2020-05-30	Kantipur (Nepali)	घरबाट निस्कन डराउँछन् नरैनापुरवासी
2020-06-16	Kantipur (Nepali)	घरबाहिर खाँदा कसरी सुरक्षित रहने?
2020-07-04	Kantipur (Nepali)	घरभाडामा छुट भए व्यवसाय धानिन्छ’
2020-08-28	Kantipur (Nepali)	घरमै बनाऔं मास्क
2020-08-18	Kantipur (Nepali)	घरमै बसेका संक्रमितहरूलाई निगरानी गरौं
2020-06-07	Kantipur (Nepali)	घरमै बस्दा झन् बढी हिंसा ?
2020-05-20	Kantipur (Nepali)	घरै बसौं, स्वस्थ रहौं
2020-03-09	Kantipur (Nepali)	घरैमा बनाएर बिक्री
2020-04-27	Online Khabar (Nepali)	घोराहीमा गरिएको पीसीआर परीक्षणमा ११ जनाको रिपोर्ट नेगेटिभ
2020-06-23	Kantipur (Nepali)	चन्दा उठाएर पीसीआर मेसिन
2020-08-30	Kantipur (Nepali)	चमत्कार दाबी गर्दै भीड
2020-08-06	Kantipur (Nepali)	चाडपर्व मनाउँदा सावधान रहौं
2020-05-03	Online Khabar (Nepali)	चाप बढेपछि उपत्यकामा थप सतर्कता
2020-04-30	Kantipur (Nepali)	चामल 'गुणस्तरहीन' भेटिएपछि वितरणमा रोक
2020-03-02	Kantipur (Nepali)	चार जना आइसोलेसनमा
2020-08-06	Online Khabar (Nepali)	चिकित्सक र प्राविधिकले पाएनन्, महानगरका कर्मचारीले लिए जोखिम भत्ता
2020-05-22	Online Khabar (Nepali)	चिकित्सक र महानगरपालिकालाई २ संस्थाको स्वास्थ्य सामग्री

Date of publication (yyyy/mm/dd)	Name of news media (select from drop-down menu)	Title of news/headline
2020-08-12	Online Khabar (Nepali)	चिकित्सक र स्वास्थ्यकर्मीको पीसीआर गरेर मात्रै सेवामा खटाउन निर्देशन
2020-08-25	Online Khabar (Nepali)	चितवनका ४ नगरपालिकाले बनाए संयुक्त आइसोलेसन
2020-06-15	Online Khabar (Nepali)	चितवनको कोरोना अस्पताल खाली गराइयो, संक्रमित भरतपुर अस्पतालमा
2020-05-05	Online Khabar (Nepali)	चितवनको कोरोना परीक्षण ल्याब पाँच दिनदेखि बन्द
2020-08-21	Online Khabar (Nepali)	चितवनमा एक साता निषेधाज्ञा थपियो
2020-04-01	Online Khabar (Nepali)	चितवनमा कोरोना भाइरस परीक्षण कहिले ?
2020-08-28	Online Khabar (Nepali)	चितवनमा भदौ १९ गतेसम्म निषेधाज्ञा
2020-04-05	Online Khabar (Nepali)	चितवनमा सोमबारबाट कोरोना भाइरसको नमूना परीक्षण सुरु हुने
2020-03-22	Kantipur (Nepali)	चित्लाङका होटल बन्द
2020-08-06	Kantipur (Nepali)	चिनियाँ दूतावासद्वारा सरकारी ल्याबको रिपोर्ट अस्वीकार
2020-03-03	Kantipur (Nepali)	चिनियाँ देख्दा हच्किन्छन् स्थानीय
2020-02-21	Kantipur (Nepali)	चिनियाँ नआउँदा पुनर्निर्माण रोकियो
2020-03-20	Kantipur (Nepali)	चिनियाँ सहयोग लिन पत्राचार
2020-08-09	Kantipur (Nepali)	चिया मजदुर भन्छन्– पुरानै ज्याला पाएनौं
2020-03-23	Kantipur (Nepali)	चीन र भारतबाट चिकित्सा सामान ल्याइने
2020-02-02	Kantipur (Nepali)	चीनतिरै लगिए मास्क
2020-04-30	Kantipur (Nepali)	चीनबाट ३ सय ४० टन स्वास्थ्य सामग्री ल्याइँदै
2020-02-02	Online Khabar (Nepali)	चीनबाट उद्दार गरिने नेपालीलाई कहाँ राख्ने ?
2020-02-11	Kantipur (Nepali)	चीनबाट नेपालीको उद्धार गर्न सांसदको माग
2020-02-06	Kantipur (Nepali)	चीनबाट फर्किएकाहरू मान्दैनन् अस्पताल बस्न
2020-02-04	Kantipur (Nepali)	चीनबाट ल्याएपछि १४ दिन निगरानीमा
2020-02-10	Kantipur (Nepali)	चीनमा रहेका विद्यार्थीको अझै उद्धार भएन
2020-02-13	Kantipur (Nepali)	चीनमै पो सुरक्षित हुन्छन् कि !
2020-05-11	Online Khabar (Nepali)	चीनले दियो ४० हजार थान पीसीआर किट
2020-03-18	Kantipur (Nepali)	चीनले राख्यो सहयोग प्रस्ताव
2020-05-18	Kantipur (Nepali)	चुनौतीको फ्रन्ट लाइनमा साना र मझौला उद्यम
2020-05-18	Kantipur (Nepali)	चैतमा ४ प्रतिशत रेमिट्यान्स घट्यो
2020-03-21	Kantipur (Nepali)	चोक चोकमा साबुनपानी
2020-05-12	Kantipur (Nepali)	छलफलबाट निकास खोज्नुपर्छ
2020-08-12	Kantipur (Nepali)	छाक टार्नै मुस्किल
2020-08-19	Kantipur (Nepali)	छिमेकीको अपमानपछि फेरि आइसोलेसनमै
2020-03-17	Kantipur (Nepali)	छुट्टै अस्पताल माग
2020-08-06	Kantipur (Nepali)	छेकबारले गरिखानै गाह्रो
2020-08-18	Kantipur (Nepali)	छोटा फिल्ममा कोरोना कहर
2020-06-21	Kantipur (Nepali)	छोरा स्याहार्न आमा आइसोलेसनमा
2020-05-05	Kantipur (Nepali)	छोरी रोइरहन्छे, म सुन्न सक्दिनँ
2020-06-26	Kantipur (Nepali)	जँचाउन जानै पाएनन् गर्भवती
2020-08-14	Kantipur (Nepali)	जटिल अवस्थाका संक्रमितले उपचार नपाउने जोखिम
2020-04-24	Online Khabar (Nepali)	जनकपुरको कोरोना रिपोर्ट काठमाडौंमा पुनः परीक्षण गरिने
2020-08-09	Online Khabar (Nepali)	जनकपुरधाममा साउनभरि नै निषेधाज्ञा
2020-05-02	Online Khabar (Nepali)	जनकपुरमा पुरानै मोडेलको पीसीआर मेसिन
2020-07-03	Kantipur (Nepali)	जनताको कर, जनप्रतिनिधिलाई सुविधा

Date of publication (yyyy/mm/dd)	Name of news media (select from drop-down menu)	Title of news/headline
2020-08-31	Online Khabar (Nepali)	जनमैत्री अस्पतालको भवनमा १०० बेडको आइसोलेसन सञ्चालन
2020-08-28	Online Khabar (Nepali)	जनमैत्री, कान्तिपुर र नेशनल पनि कोभिड विशेष अस्पताल
2020-05-25	Online Khabar (Nepali)	जनसंख्याको दुई प्रतिशतको कोरोना परीक्षण गरिन्छ : प्रधानमन्त्री
2020-07-03	Online Khabar (Nepali)	जनस्तरबाट रकम उठाएर डडेलधुरामा पीसीआर मेसिन
2020-06-10	Kantipur (Nepali)	जनस्वास्थ्य प्रयोगशालालाई गुणस्तर प्रमाणपत्र
2020-05-23	Kantipur (Nepali)	जब अँध्यारोमा जून देखियो
2020-05-03	Kantipur (Nepali)	जमिन बाँझो नराखौं
2020-06-22	Kantipur (Nepali)	जसले एक्लाएक्लै सम्हालिरहेछन्क्वारेन्टाइन र आइसोलेसन
2020-05-22	Kantipur (Nepali)	जसले कोरोना जितिन्
2020-06-25	Kantipur (Nepali)	जसले संक्रमित सम्हालिरहेकी छन्
2020-05-15	Kantipur (Nepali)	जीवनरक्षा र घरबाटै कामको योजना
2020-04-05	Online Khabar (Nepali)	जुन भाग कमजोर, त्यहीँ कोरोनाको दोस्रो हमला
2020-07-15	Online Khabar (Nepali)	जुम्लाको खलङ्गामा निषेधाज्ञा
2020-06-03	Online Khabar (Nepali)	जुम्लामा ३ दिनका लागि निषेधाज्ञा जारी
2020-06-05	Online Khabar (Nepali)	जेठ २५ देखि निश्चित जिल्लामा छोटो दुरीका सवारी चलाउने प्रस्ताव
2020-07-22	Online Khabar (Nepali)	जेठमा मगाएको अटोमेटेड पीसीआर मेसिन साउनमा आइपुग्यो
2020-06-12	Kantipur (Nepali)	जो कोरोना जितेर काममा जुटिसकेका छन्
2020-08-22	Kantipur (Nepali)	जो दिनभर संक्रमित बोक्छन्
2020-06-09	Kantipur (Nepali)	जोखिम बढ्यो, सावधानी छैन
2020-06-06	Kantipur (Nepali)	जोखिम बाँड्ने सहमतिमा कोरोना बिमा पुनः सुरु
2020-03-22	Kantipur (Nepali)	जोखिम रोक्न विपद् कोषबाट खर्च गरिने
2020-07-07	Kantipur (Nepali)	जोगिने एक मात्र उपाय सावधानी'
2020-05-03	Kantipur (Nepali)	ज्याला माग्न ६ दिन हिँडेर खोटाङबाट काठमाडौं
2020-08-19	Kantipur (Nepali)	झन्डै ५ हजार महिलालाई सहुलियत ऋण
2020-05-21	Online Khabar (Nepali)	झापाको कचनकवल गाउँपालिका सिल
2020-05-30	Online Khabar (Nepali)	झापाबाट धरान पठाइएका संक्रमितलाई भर्ना नगरी रातभर बाहिरै राखियो
2020-06-26	Kantipur (Nepali)	टिकट काट्न नसक्नेहरू खाडीमै अलपत्र
2020-08-27	Online Khabar (Nepali)	टेक अवे र होम डेलिभरी सेवा प्रदायक प्रहरी नियन्त्रणमा
2020-04-09	Online Khabar (Nepali)	टेकु अस्पतालको एउटा गेट, जसले संक्रमणबाट जोगाउँछ
2020-04-02	Online Khabar (Nepali)	टेकुमा उपचाररत कोरोना संक्रमित दुई जनाको दोस्रो रिपोर्ट पनि पोजेटिभ
2020-05-13	Kantipur (Nepali)	टेलिकमको आम्दानी घट्यो
2020-08-27	Online Khabar (Nepali)	टोखा नगरपालिकाबाट केही सिक्ने कि !
2020-03-03	Kantipur (Nepali)	टोखा महोत्सव स्थगित
2020-07-09	Kantipur (Nepali)	ट्याक्सी चलाउन दिन विकास समितिको निर्देशन
2020-08-12	Kantipur (Nepali)	ट्रेसिङ नहुादा संक्रमित घरमै
2020-04-27	Kantipur (Nepali)	ट्रेसिङको काम सन्तोषजनक : महाशाखा
2020-05-11	Kantipur (Nepali)	ट्र्याकमा फर्कन आतुर
2020-03-21	Online Khabar (Nepali)	ठमेलका अधिकांश होटल रेष्टुराँ बन्द, कर्मचारीलाई बेतलबी बिदा
2020-05-30	Kantipur (Nepali)	ठाउँ हेरी लकडाउन खुकुलो पारौं : विज्ञ
2020-06-08	Kantipur (Nepali)	ठाकुरलाई सहयोग जुट्यो
2020-08-27	Online Khabar (Nepali)	ठूला अस्पताललाई कोरोना विशेष बनाऊ : शान्ति समाज
2020-05-18	Online Khabar (Nepali)	ठोरीको सीमा आइपुगेकाहरूलाई भारततर्फ फर्काइयो

Date of publication (yyyy/mm/dd)	Name of news media (select from drop-down menu)	Title of news/headline
2020-08-27	Online Khabar (Nepali)	डाक्टरलाई कोरोना देखिएपछि जाजरकोट अस्पतालको ओपीडी सेवा बन्द
2020-06-24	Kantipur (Nepali)	डाबरका सेनिटाइजर र फ्लोर क्लिनर
2020-03-04	Kantipur (Nepali)	डेढ महिनामा ४३ लाख मास्क निर्यात
2020-05-16	Online Khabar (Nepali)	डेरामा डाक्टर र नर्सलाई अवरोध नगरौं : स्वास्थ्य मन्त्रालय
2020-08-24	Online Khabar (Nepali)	डेरावाल संक्रमितलाई घरबेटीले नै बनाइदिए आइसोलेसन
2020-07-02	Kantipur (Nepali)	डेरीको कारोबार ६० प्रतिशत
2020-04-08	Online Khabar (Nepali)	डोटीबाट ३२६ जनाको नमुना संकलन
2020-03-20	Online Khabar (Nepali)	डोटीमा कोरोना रोकथाम : हेल्थ डेस्कदेखि भाकलसम्म
2020-04-12	Online Khabar (Nepali)	डोटीमा थप चार हजारको र्‍यापिड टेस्ट गर्न माग
2020-06-10	Kantipur (Nepali)	डोरीले घेरेर आइसोलेसन
2020-06-12	Kantipur (Nepali)	ढुलमुले सरकारी नीति
2020-06-10	Kantipur (Nepali)	ढुवानीमा समस्यै समस्या
2020-03-18	Kantipur (Nepali)	ढोकैमा साबुन पानी
2020-04-12	Online Khabar (Nepali)	तत्काल कोरोना अस्पताल सञ्चालन गर्न वीरगञ्जका चिकित्सकको माग
2020-05-20	Kantipur (Nepali)	तत्काल ल्याउनुपर्ने नेपाली २० हजार
2020-08-14	Online Khabar (Nepali)	तनहुँको भानु नगरपालिकाको एउटै वडाका २० जनामा संक्रमण पुष्टि
2020-05-17	Kantipur (Nepali)	तयार छन् हाम्रा विश्वविद्यालय?
2020-03-08	Kantipur (Nepali)	तयार भए आइसोलेसन वार्ड
2020-06-07	Kantipur (Nepali)	तरकारी बिक्री नभएपछि चौपायालाई आहारा र मल
2020-08-24	Kantipur (Nepali)	तरकारीको मूल्य अचाक्ली
2020-08-12	Kantipur (Nepali)	तलबको आस मारेर स्वदेश फिर्ती
2020-03-11	Kantipur (Nepali)	ताक्लाकोटमा डेढ सय नेपाली अलपत्र
2020-05-08	Kantipur (Nepali)	तातोपानी सबै खाले सामानलाई खुला
2020-03-08	Kantipur (Nepali)	तापक्रम नाप्दै, बस चढाउँदै
2020-08-09	Kantipur (Nepali)	तारे होटलका लगानीकर्ता निराश
2020-04-09	Online Khabar (Nepali)	तिलोत्तमा क्वारेन्टाइनका १० जनाको र्‍यापिड डाइग्नोस्टिक टेस्ट
2020-08-17	Online Khabar (Nepali)	तीजमा माइत नजान वडा कार्यालयको अनुरोध
2020-05-08	Online Khabar (Nepali)	तीन दिनभित्र ४ हजार पीसीआर परीक्षण गरिने
2020-08-29	Kantipur (Nepali)	तीन मन्त्री र प्रधानसेनापति क्वारेन्टाइनमा
2020-06-29	Kantipur (Nepali)	तीन महिनामा १८ सय चार्टर उडान
2020-06-19	Kantipur (Nepali)	तीन महिनामा एटीएमबाट सवा ५४ अर्ब झिकियो
2020-04-29	Kantipur (Nepali)	तीन सय भीटीएम आवश्यक, ६० प्राप्त
2020-05-25	Kantipur (Nepali)	तीन सय रेजिडेन्ट डाक्टर घर फर्कंदै
2020-04-09	Online Khabar (Nepali)	तीन स्थानीय तहलाई शर्माको ३० लाख सहयोग
2020-08-06	Kantipur (Nepali)	तोकिएका केन्द्रबाट हजार नमुना संकलन
2020-06-18	Kantipur (Nepali)	दक्षिण कोरियाले दियो ५० हजार किट
2020-06-10	Kantipur (Nepali)	दबाबमा स्थानीय सरकार
2020-03-06	Kantipur (Nepali)	दर्जनभन्दा बढी आयोजना अन्योल
2020-05-12	Online Khabar (Nepali)	दशगजाबाट लुकिछिपी आएका थिए रुपन्देहीका ६ जना संक्रमित, एकजना भैरहवासम्म पुगेका थिए
2020-05-09	Kantipur (Nepali)	दानको प्रचार : कति जायज?
2020-06-09	Kantipur (Nepali)	दिनभरि क्वारेन्टाइन, रात परेपछि घर
2020-04-24	Kantipur (Nepali)	दिनरात संक्रमितको हेरचाह

Date of publication (yyyy/mm/dd)	Name of news media (select from drop-down menu)	Title of news/headline
2020-03-22	Kantipur (Nepali)	दिल्लीबाट धमाधम फर्किंदै नेपाली
2020-05-12	Online Khabar (Nepali)	दिल्लीबाट हिँडेरै जोगवनी, भारतीय प्रहरीले सीमा कटायो
2020-07-11	Online Khabar (Nepali)	दुई अंकमा झर्‍यो नयाँ संक्रमितको संख्या, सक्रिय संक्रमितभन्दा निको हुने धेरै
2020-08-30	Kantipur (Nepali)	दुई महिनामा ४० हजार सेयर लगानीकर्ता थपिए
2020-08-30	Online Khabar (Nepali)	दुई हप्तापछि गोरखामा हटाइयो निषेधाज्ञा, नाकामा भने कडाइ
2020-06-23	Kantipur (Nepali)	दुग्ध पदार्थ बिकाउनै सकस
2020-08-22	Kantipur (Nepali)	दुबईमा अलपत्र २ सय ३५ यात्रुलाई सोमबार ल्याइँदै
2020-08-24	Kantipur (Nepali)	दुबईमा अलपत्र २६२ यात्रुको उद्धार
2020-08-26	Kantipur (Nepali)	दुर्व्यवहारले लज्जित
2020-06-16	Kantipur (Nepali)	दूर शिक्षा रेडियो कान्तिपुरमा
2020-08-03	Kantipur (Nepali)	दूरी र मास्कले मार्सल आर्ट्सको अभ्यास प्रभावित
2020-03-17	Kantipur (Nepali)	देउवाको ध्यानाकर्षण
2020-02-20	Kantipur (Nepali)	देखिएन कोभिड–१९
2020-08-12	Online Khabar (Nepali)	देवघाट क्षेत्र विकास समितिका अध्यक्षमा कोरोना देखिएपछि देवघाट गाउँपालिका सिल
2020-05-21	Kantipur (Nepali)	देवचुलीका ५ वटा वडा सिल
2020-07-10	Kantipur (Nepali)	देश फर्के ज्यानलाई खतरा
2020-06-13	Kantipur (Nepali)	देशव्यापी बन्दै प्रदर्शन
2020-08-07	Kantipur (Nepali)	देशव्यापी लकडाउन अनावश्यक
2020-03-06	Kantipur (Nepali)	दैनिक ६ पसलमा मात्रै अनुगमन
2020-07-23	Online Khabar (Nepali)	दैलेखको ठाटीकाँध गाउँपालिकामा अनिश्चितकालीन निषेधाज्ञा
2020-08-20	Online Khabar (Nepali)	दैलेखमा आजैदेखि चार दिनसम्म निषेधाज्ञा
2020-04-05	Online Khabar (Nepali)	धनगढीका कोरोना संक्रमित युवकको दोस्रो रिपोर्ट पनि पोजेटिभ
2020-04-10	Online Khabar (Nepali)	धनगढीमा कोरोनाका परीक्षणको उपकरण जडानमा ढिलाइ
2020-04-02	Online Khabar (Nepali)	धनगढीमा कोरोनाको परीक्षण सुरु
2020-08-16	Online Khabar (Nepali)	धनुषामा एक साताका लागि निषेधाज्ञा जारी
2020-04-05	Online Khabar (Nepali)	धुलिखेलमा पनि कोरोना परीक्षण, तर सातामा दुई दिनमात्रै
2020-05-28	Kantipur (Nepali)	नबिलको २८ लाख सहयोग
2020-05-24	Online Khabar (Nepali)	नयाँबानेश्वरका तीन घर सिल
2020-05-25	Kantipur (Nepali)	नरैनापुर निषेधित क्षेत्र घोषणा
2020-05-21	Kantipur (Nepali)	नरैनापुरका संक्रमितलाई सास्ती
2020-05-26	Kantipur (Nepali)	नरैनापुरमा खटिए चिकित्सक
2020-05-24	Online Khabar (Nepali)	नरैनापुरलाई निषेधित क्षेत्र घोषणा, सेना परिचालन गर्ने निर्णय
2020-04-22	Online Khabar (Nepali)	नर्समा देखिएको आरडीटी पोजेटिभको संकेत : लहरो तान्दा पहरो जाने खतरा
2020-08-22	Kantipur (Nepali)	नर्सले मनाए अस्पतालमै
2020-05-14	Online Khabar (Nepali)	नवलपरासीका संक्रमित भारतबाट लुकिछिपी आएका थिए
2020-06-03	Kantipur (Nepali)	नष्ट गर्नेलाई कारबाही सिफारिस
2020-03-22	Kantipur (Nepali)	नाका बन्द गरिदिन प्रदेश १ को माग
2020-06-19	Kantipur (Nepali)	नाका बन्द हुँदा बीउ आयात बढ्यो
2020-05-27	Kantipur (Nepali)	नाका–नाकामा घुइँचो
2020-03-20	Kantipur (Nepali)	नाकाबाट निर्बाध आवागमन
2020-05-10	Kantipur (Nepali)	नाकामा उकुसमुकुस

Date of publication (yyyy/mm/dd)	Name of news media (select from drop-down menu)	Title of news/headline
2020-06-17	Kantipur (Nepali)	नाकामा चाप उस्तै, सास्ती घट्दै
2020-03-14	Kantipur (Nepali)	नाकामा छैन पर्याप्त सतर्कता
2020-08-04	Online Khabar (Nepali)	नाकामा झण्डै ११ हजारको परीक्षण गर्दा ४० जना संक्रमित
2020-06-13	Kantipur (Nepali)	नाकामा भारतीयको बलजफ्ती
2020-08-16	Online Khabar (Nepali)	नाकामा सक्नुभएन, क्वारेन्टिन बनाएर देखाउनुस् !
2020-03-22	Kantipur (Nepali)	नाकाहरूमा जोखिमपूर्ण आवागमन
2020-06-01	Kantipur (Nepali)	नाकाहरूमा थामिनसक्नु भीड
2020-03-05	Kantipur (Nepali)	नाकाहरूमा निगरानी कमजोर
2020-05-18	Kantipur (Nepali)	नागरिकका दुःखमा सरकार किन बेखबर ?
2020-05-04	Online Khabar (Nepali)	नारायणीका कोरोना संक्रमितको दैनिकी : नमाज पढ्ने, फोनमा गफिने
2020-07-17	Kantipur (Nepali)	निःशुल्क मास्क वितरण
2020-05-06	Kantipur (Nepali)	निको भएकामा पीसीआर पोजिटिभ
2020-05-26	Kantipur (Nepali)	निको भएर फर्के एकैपल्ट ४३ जना
2020-05-10	Kantipur (Nepali)	निको हुने दर नबढेकोप्रति विज्ञको चिन्ता
2020-08-02	Kantipur (Nepali)	निगमको घाटा १० अर्ब
2020-08-14	Kantipur (Nepali)	निजी अस्पताल भन्छन्– 'तोकिएकै शुल्कमा उपचार असम्भव'
2020-05-26	Online Khabar (Nepali)	निजी अस्पतालमा र उपत्यका प्रवेश गर्नेको आरडीटी टेस्ट बेकार !
2020-05-20	Online Khabar (Nepali)	निजी अस्पताललाई प्रयोग गर्न दिइने आरडीटी किटको गुणस्तरमै शंका
2020-08-27	Kantipur (Nepali)	निजी अस्पतालले पनि गर्न थाले संक्रमितको उपचार
2020-06-16	Kantipur (Nepali)	निजी आवास पुनर्निर्माणले गति लिँदै
2020-06-03	Kantipur (Nepali)	निजी क्षेत्रको सुझाव समेटेर नयाँ ढाँचा
2020-07-02	Kantipur (Nepali)	निजी शिक्षक अन्योलमा
2020-07-31	Kantipur (Nepali)	निजीलाई पनि स्वाब संकलन र पीसीआर अनुमति
2020-06-03	Kantipur (Nepali)	निजीलाई पीसीआर परीक्षण गर्न दिने तयारी छैन
2020-06-15	Kantipur (Nepali)	निजीले पीसीआर परीक्षण गर्न पाउने
2020-06-09	Kantipur (Nepali)	निर्मम खर्च कटौतीपछि मात्रै ऋण
2020-05-13	Kantipur (Nepali)	निर्माण क्षेत्रमा कतै काम सुरु, कतै तयारी
2020-08-23	Kantipur (Nepali)	निर्माण व्यवसायीले फिर्ता मागे धरौटी
2020-08-30	Online Khabar (Nepali)	निषेधाज्ञा हटाउन चितवनका उद्योगी व्यवसायीको माग
2020-08-28	Kantipur (Nepali)	निषेधाज्ञामा झन् चहलपहल
2020-08-24	Kantipur (Nepali)	निषेधाज्ञाले महामारी रोक्न सकिन्न
2020-08-25	Kantipur (Nepali)	निषेधाज्ञाले हेटौंडाका प्रायः उद्योग बन्द
2020-05-06	Kantipur (Nepali)	निस्फिक्री घुमफिर गरेका थिए संक्रमित
2020-08-07	Online Khabar (Nepali)	नुवाकोट आजदेखि 'लकडाउन'
2020-08-11	Online Khabar (Nepali)	नुवाकोटमा निषेधाज्ञा क्षेत्र बढाइयो, सेना परिचालन
2020-05-08	Kantipur (Nepali)	नेकपामा बहकिएको बहस
2020-06-27	Kantipur (Nepali)	नेताका गलामा कोरोना भगाउने कार्ड !
2020-06-29	Kantipur (Nepali)	नेताले भाइरस रिमुभिङ कार्ड लगाउन छाडे
2020-02-19	Kantipur (Nepali)	नेपाल कोरोनामुक्त : पर्यटन मन्त्रालय
2020-05-08	Kantipur (Nepali)	नेपाल जान सकिने भ्रम फैलाइयो'
2020-05-04	Kantipur (Nepali)	नेपाल लाइफका कर्मचारीले दिए ८ लाख
2020-08-09	Online Khabar (Nepali)	नेपालगञ्ज वडा प्रहरी कार्यालयका ३० जनामा कोरोना संक्रमण
2020-05-04	Online Khabar (Nepali)	नेपालगञ्जमा २५ गतेसम्म निषेधित क्षेत्र घोषणा

Date of publication (yyyy/mm/dd)	Name of news media (select from drop-down menu)	Title of news/headline
2020-05-08	Kantipur (Nepali)	नेपालगन्जमा ६० किट मात्रै
2020-05-11	Kantipur (Nepali)	नेपालगन्जमा पहिलो चरणको ट्रेसिङ पूरा
2020-05-07	Online Khabar (Nepali)	नेपालमा कोरोना संक्रमण भयावह हुने लक्षण देखिएको छ, सजग रहौं : प्रधानमन्त्री ओली
2020-05-02	Online Khabar (Nepali)	नेपालमा कोरोनाको प्रयोगशाला परीक्षण १३ हजार मात्र
2020-08-27	Kantipur (Nepali)	नेपालमा निको हुने दर सार्कमै सबैभन्दा कम
2020-04-04	Online Khabar (Nepali)	नेपालमा पहिलो पटक स्थानीयमा देखियो कोरोना संक्रमण
2020-04-02	Online Khabar (Nepali)	नेपालमा भेटिए कोरोनाका छैटौं संक्रमित
2020-06-05	Kantipur (Nepali)	नेपाली कामदार आजदेखि आउँदै
2020-07-21	Kantipur (Nepali)	नेपाली फर्काउन कम्पनीले चार्टर अनुमति पाएनन्
2020-06-12	Kantipur (Nepali)	नेपालीलाई उद्धार गर्न ६७ चार्टर उडान हुने
2020-07-07	Kantipur (Nepali)	नेप्सेको कारोबार प्रणालीमा समस्या
2020-08-30	Online Khabar (Nepali)	नेसनल मेडिकल कलेजमा ५ सय शय्याको आइसोलेसन केन्द्र बनाइने
2020-07-04	Kantipur (Nepali)	न्युरोडको नबदलिएको चरित्र
2020-03-27	Online Khabar (Nepali)	न्यूनतम आवागमन खुला गर्ने सरकारको निर्णय
2020-05-05	Kantipur (Nepali)	पञ्चकन्याले दियो स्वास्थ्य सामग्री
2020-05-02	Kantipur (Nepali)	परम्परागत क्वारेन्टाइन' मा पतियाको छाया
2020-07-30	Online Khabar (Nepali)	परवानीपुरमा कोरोनाले मृत्यु हुनेको परम्परागतरुपमै अन्त्येष्टि गर्ने निर्णय
2020-07-31	Kantipur (Nepali)	परीक्षण कम गरे सरकार सफल !
2020-06-15	Kantipur (Nepali)	परीक्षण ढिलाइले कामदारको उडान सर्दै
2020-06-24	Kantipur (Nepali)	परीक्षण ढिलाइले जोखिम बढ्दो
2020-03-23	Kantipur (Nepali)	परीक्षण थोरै
2020-05-14	Kantipur (Nepali)	परीक्षण दायरा बढाउन सांसदको माग
2020-06-11	Kantipur (Nepali)	परीक्षण नगर्दाको जटिलता
2020-06-11	Kantipur (Nepali)	परीक्षणबिनै घर
2020-05-28	Kantipur (Nepali)	परीक्षणबिनै धमाधम भित्रिँदै
2020-05-22	Kantipur (Nepali)	परीक्षणबिनै भारतबाट भित्रिए ४२ सय
2020-06-10	Kantipur (Nepali)	परीक्षणबिनै संक्रमित धमाधम घर
2020-06-04	Kantipur (Nepali)	परीक्षणसँगै खाँचो मनोबलको
2020-07-26	Kantipur (Nepali)	परीक्षासँगै सुरक्षा र सहुलियत
2020-03-09	Kantipur (Nepali)	पर्यटक नआएपछि चिज उद्योग बन्द
2020-07-07	Kantipur (Nepali)	पर्यटक पर्खिरहेछन् व्यवसायी
2020-05-27	Kantipur (Nepali)	पर्यटन क्षेत्रमा ५ वर्षपछि ९ लाखलाई रोजगारी
2020-04-05	Online Khabar (Nepali)	पर्साका क्वारेन्टाइनमा ३९ जना, १३७ जना फर्किए
2020-07-31	Online Khabar (Nepali)	पर्सागढी नगरपालिका अनिश्चितकालका लागि बन्द
2020-07-28	Online Khabar (Nepali)	पर्सागढीका ५ वटै स्वास्थ्य संस्थाका स्वास्थ्यकर्मीमा कोरोना संक्रमण
2020-07-23	Online Khabar (Nepali)	पर्सामा ५ थुनुवासहित ३२ जनामा कोरोना संक्रमण पुष्टि
2020-08-01	Online Khabar (Nepali)	पर्सामा कोरोना संक्रमितको शव व्यवस्थापन टोली र स्थानीयबीच झडप, ७ प्रहरी घाइते
2020-08-16	Online Khabar (Nepali)	पर्सामा निषेधाज्ञामा कडा पार्न सेना परिचालन
2020-08-08	Online Khabar (Nepali)	पर्सामा मेयरसहित ५५ जनामा कोरोना, ल्याबका कर्मचारी पनि संक्रमित
2020-08-27	Online Khabar (Nepali)	पर्सामा सबै ठाउँमा निषेधाज्ञा जरुरी देखिएन : प्रजिअ तामाङ
2020-06-14	Kantipur (Nepali)	पसल खुले पनि छैनन् ग्राहक
2020-06-07	Kantipur (Nepali)	पसल खोल्दा घरबेटीको ताला

Date of publication (yyyy/mm/dd)	Name of news media (select from drop-down menu)	Title of news/headline
2020-06-11	Kantipur (Nepali)	पसल बन्दविरुद्ध व्यवसायीको प्रदर्शन
2020-05-06	Kantipur (Nepali)	पहिचान लुकाउँदा कन्ट्याक्ट ट्रेसिङ जटिल
2020-06-09	Kantipur (Nepali)	पहिलो उडानमै मापदण्ड 'फेल'
2020-08-16	Kantipur (Nepali)	पहुँचवालाका घरघरै पीसीआर
2020-05-19	Kantipur (Nepali)	पानीसमेत छैन
2020-06-23	Kantipur (Nepali)	पालिकाभित्रै प्रयोगशाला बनाउनुपर्छ
2020-06-26	Kantipur (Nepali)	पालिकाहरूमा कतै कर छुट, कतै मिनाहा
2020-05-06	Kantipur (Nepali)	पास भएपछि कसरी रोक्नु? : सुरक्षाकर्मी
2020-06-25	Kantipur (Nepali)	पास र पीसीआरमा लुकेको नियत
2020-05-06	Kantipur (Nepali)	पीडितलाई सविता दम्पतीको सहयोग
2020-05-13	Kantipur (Nepali)	पीसीआर परीक्षण गरेर भित्र्याउने योजना
2020-04-27	Kantipur (Nepali)	पीसीआर परीक्षण घट्दै
2020-06-07	Kantipur (Nepali)	पीसीआर परीक्षण निजीलाई पनि देऊ
2020-07-11	Kantipur (Nepali)	पीसीआर परीक्षण शुल्क अत्यधिक महँगो
2020-08-30	Online Khabar (Nepali)	पीसीआर परीक्षणको शुल्क घट्यो
2020-07-24	Online Khabar (Nepali)	पीसीआर परीक्षणबारे वीरगञ्ज महानगरका गुनासै गुनासा
2020-08-28	Kantipur (Nepali)	पीसीआर शुल्क तोकिएभन्दा बढी
2020-08-31	Kantipur (Nepali)	पीसीआरको अधिकतम शुल्क अब ४४००
2020-05-09	Kantipur (Nepali)	पुनरुत्थानका लागि तत्काल दुई खर्ब चाहिने
2020-05-19	Kantipur (Nepali)	पुनरुत्थानकेन्द्रित बजेट ल्याउन सुझाव
2020-07-13	Kantipur (Nepali)	पुनरुत्थानको जिम्मा मौद्रिक नीतिलाई
2020-07-19	Kantipur (Nepali)	पुनरुत्थानमा सघाउँछ : महासंघ
2020-05-22	Kantipur (Nepali)	पुनर्कर्जा कार्यान्वयनको पाटो
2020-08-01	Kantipur (Nepali)	पूर्ण क्वारेन्टाइनमा प्रशिक्षण
2020-03-16	Kantipur (Nepali)	पूर्वतयारीको एकीकृत खाका बनाउन निर्देशन
2020-03-06	Kantipur (Nepali)	पूर्वतयारीमा जुट्न विज्ञको सुझाव
2020-08-16	Online Khabar (Nepali)	पेट्रोल पम्प व्यवसायीको आग्रह : बरु चाहिएजति लानुस्, पटक पटक नआउनुस्
2020-05-13	Online Khabar (Nepali)	पोखरा प्रयोगशालाले कोरोनाको परीक्षण गर्न मानेन'
2020-03-28	Online Khabar (Nepali)	पोखराको १ सय ५० वेडको क्वारेन्टाइन : कतै भवनमात्र, कतै स्थानीयको विरोध
2020-03-04	Kantipur (Nepali)	पोखरामा आइसोलेसन वार्ड
2020-06-02	Online Khabar (Nepali)	पोखरामा एक हप्तामै दोब्बर महंगियो तरकारी
2020-07-14	Online Khabar (Nepali)	पोखरामा बिना मास्क हिँड्ने १०२ जना कारबाहीमा परे
2020-05-15	Kantipur (Nepali)	प्रक्रिया सुरु भएका योजना अलपत्र
2020-06-11	Kantipur (Nepali)	प्रतिरोधात्मक क्षमता बढी हुने उमेर समूहकै धेरै संक्रमित
2020-05-24	Kantipur (Nepali)	प्रतिवेदन लुकाएर र्‍यापिड किट प्रयोग
2020-08-04	Online Khabar (Nepali)	प्रत्येक प्रदेशमा तत्काल १ लाख पीसीआर टेस्ट गर्न देउवाको सुझाव
2020-04-09	Online Khabar (Nepali)	प्रत्येक प्रदेशमा पठाइयो ५/५ हजार र्‍यापिड टेस्ट किट
2020-03-16	Kantipur (Nepali)	प्रत्येक प्रदेशमा परीक्षण केन्द्र माग
2020-08-09	Online Khabar (Nepali)	प्रदेश १ मा ३०० आइसोलेसन बेड थपिने
2020-05-24	Online Khabar (Nepali)	प्रदेश १ मा कोरोना अस्पताल थपिँदै, सिद्धबाबाको आश्रम पनि प्रयोग गरिने
6/24/20202	Kantipur (Nepali)	प्रदेश १ मा कोरोनाका लागि २५ करोड खर्च

Date of publication (yyyy/mm/dd)	Name of news media (select from drop-down menu)	Title of news/headline
2020-07-14	Kantipur (Nepali)	प्रदेश १ लाई ग्लोबलको १० लाख
2020-07-27	Online Khabar (Nepali)	प्रदेश २ का आठ जिल्लामा ९ वटामात्रै भेन्टिलेटर
2020-04-24	Online Khabar (Nepali)	प्रदेश २ का सबै जिल्लामा स्वास्थ्यकर्मीको टोली, १५ सय बढी नमूना संकलन गर्ने लक्ष्य
2020-08-04	Online Khabar (Nepali)	प्रदेश २ मा ५ सय बेडको आइसोलेसन थपिने
2020-05-29	Online Khabar (Nepali)	प्रदेश २ मा एकै दिन थपिए ९२ जना संक्रमित
2020-04-25	Online Khabar (Nepali)	प्रदेश २ मा कोरोनाविरुद्ध लड्न सुस्त तयारी, नारायणी अस्पतालको मात्र भर
2020-08-21	Online Khabar (Nepali)	प्रदेश २ मा निजी अस्पताललाई कोरोना उपचारमा लगाइने
2020-08-18	Online Khabar (Nepali)	प्रदेश २ मा निजी स्वास्थ्य संस्थालाई कोरोना उपचारमा लगाइने
2020-05-06	Online Khabar (Nepali)	प्रदेश २ मा पीसीआर परीक्षणमा तीव्रता, २६ सय बढीको स्वाब संकलन
2020-08-09	Online Khabar (Nepali)	प्रदेश ५ की सांसद केसीलाई कोरोना संक्रमण पुष्टि
2020-04-18	Online Khabar (Nepali)	प्रदेश ५ मा कोरोना रोकथाम र उपचार कामचलाउमात्र
2020-07-29	Online Khabar (Nepali)	प्रदेश ५ मा कोरोना संक्रमणको 'सेकेण्ड वेभ' को डर
2020-05-21	Online Khabar (Nepali)	प्रदेश ५ मा थप २५० आइसोलेसन बेड बनाउने निर्णय
2020-06-17	Kantipur (Nepali)	प्रदेश ५ ले दियो करमा छुटैछुट
2020-05-06	Online Khabar (Nepali)	प्रदेश ५ सरकारले माग्यो कोरोना परीक्षण किट र पीपीई
2020-08-24	Online Khabar (Nepali)	प्रदेश मन्त्रालयलाई प्रश्न : महामारीमा तलब–भत्ता खाएर मात्रै बस्ने हो ?
2020-05-12	Kantipur (Nepali)	प्रदेश सरकारहरूलाई बजेटको चटारो
2020-06-16	Kantipur (Nepali)	प्रदेशलाई लक्ष्मीको स्वास्थ्य सामग्री
2020-03-18	Kantipur (Nepali)	प्रदेशसभा बैठक स्थगित गर्न माग
2020-05-19	Kantipur (Nepali)	प्रदेशसभाले बोलायो बजेट अधिवेशन
2020-06-21	Kantipur (Nepali)	प्रदेशहरूमा पनि फजुल खर्च
2020-05-25	Kantipur (Nepali)	प्रदेशहरूमा स्वास्थ्य संकट
2020-06-25	Kantipur (Nepali)	प्रधानमन्त्रीको कोरोना ॅऔषधि'
2020-05-23	Online Khabar (Nepali)	प्रधानमन्त्रीज्यू, क्वारेन्टाइनस्थलबाटै कोरोना फैलियो !
2020-07-31	Online Khabar (Nepali)	प्रधानमन्त्रीसँग वीरगञ्जमा ५ सय शय्याको कोभिड अस्पतालको माग
2020-06-19	Kantipur (Nepali)	प्रयोग नभएको औषधि गोदाममा छ : सेना
2020-05-26	Kantipur (Nepali)	प्रयोगशालामा थुप्रिँदै स्वाब
2020-06-10	Kantipur (Nepali)	प्रयोगशालामा स्वाबको थुप्रो
2020-04-27	Kantipur (Nepali)	प्रवासी नेपालीको अवस्था आकलन गर्दै परराष्ट्र
2020-08-24	Kantipur (Nepali)	प्रहरीकै सुरक्षा कसले गर्ने?
2020-04-24	Kantipur (Nepali)	प्रहरीसँग छैन घर फर्कनेको विवरण
2020-07-27	Online Khabar (Nepali)	प्रादेशिक अस्पतालको आईसीयू एक साताका लागि बन्द
2020-05-13	Kantipur (Nepali)	प्रायः उद्योग 'पर्ख र हेर'मा
2020-08-03	Kantipur (Nepali)	प्लाज्मा थेरापीले जगायो आशा
2020-05-10	Kantipur (Nepali)	फाजिलका कर्मचारीलाई स्थानीय तहमा पठाइने
2020-05-09	Kantipur (Nepali)	फिर्न चाहनेको दशगजामा भीड
2020-05-15	Kantipur (Nepali)	फेरि बन्द भयो सेयर बजार
2020-07-11	Kantipur (Nepali)	फेरि बन्द हुने अवस्थामा सपिङ मल
2020-03-22	Kantipur (Nepali)	फोनबाटै परामर्श
2020-08-13	Kantipur (Nepali)	फ्रन्टलाइनका स्वास्थ्यकर्मीको पीसीआर गराउनू'
2020-05-24	Online Khabar (Nepali)	फ्रन्टलाइनमा खटिने स्वास्थ्यकर्मीको पीडा
2020-03-17	Kantipur (Nepali)	फ्रन्टलाइनमै छैन उच्च सावधानी

Date of publication (yyyy/mm/dd)	Name of news media (select from drop-down menu)	Title of news/headline
2020-08-27	Kantipur (Nepali)	बंगलादेशबाट रेमडेसिभिर सहयोग
2020-07-04	Kantipur (Nepali)	बजार खुला, व्यापार ठप्प
2020-03-06	Kantipur (Nepali)	बजारमा चलखेल हुन नदेऊ
2020-05-04	Kantipur (Nepali)	बजेट अधिवेशन २६ गतेदेखि
2020-08-01	Kantipur (Nepali)	बजेट छुट्याइयो, ल्याइएनन् भेन्टिलेटर
2020-05-28	Kantipur (Nepali)	बजेट स्वास्थ्य र रोजगारी वरिपरि
2020-05-27	Kantipur (Nepali)	बजेटबाट अपेक्षा
2020-05-11	Kantipur (Nepali)	बजेटमा ६ क्षेत्रलाई प्राथमिकता
2020-05-17	Kantipur (Nepali)	बजेटलाई स्रोत जुटाउन मुस्किल
2020-05-19	Kantipur (Nepali)	बजेटले बोल्नैपर्ने विषय
2020-07-03	Online Khabar (Nepali)	बझाङमा कोरोना संक्रमित युवकले गरे आत्महत्या
2020-07-16	Kantipur (Nepali)	बढाइएन पीसीआर परीक्षण
2020-06-17	Kantipur (Nepali)	बढेन कोरोनाबाट निको हुने दर
2020-05-21	Kantipur (Nepali)	बढ्दै छ अत्यावश्यक वस्तु आयात
2020-05-04	Kantipur (Nepali)	बढ्दो बन्दाबन्दीमा शिक्षा
2020-05-12	Kantipur (Nepali)	बढ्दो महामारी हल्का तम्तयारी
2020-05-16	Kantipur (Nepali)	बदलियो प्राथमिकता
2020-02-25	Kantipur (Nepali)	बनेन थर्मल स्क्यानर
2020-07-23	Kantipur (Nepali)	बन्दाबन्दी खुले पनि खतरा कायमै
2020-03-17	Kantipur (Nepali)	बरामद मास्क सरकारले बेच्ने
2020-08-10	Kantipur (Nepali)	बल्ल आइसोलेसन भवन खोजी
2020-08-22	Online Khabar (Nepali)	बल्ल आइसोलेसन सेन्टर बनाउन जुटे चितवनका स्थानीय तह
2020-06-14	Kantipur (Nepali)	बस गुड्दैनन्, बैंकले किस्ता छाड्दैनन्
2020-03-05	Kantipur (Nepali)	बस चढ्दा ज्वरो नाप्नुपर्ने
2020-07-05	Kantipur (Nepali)	बस व्यवसायीलाई कर्जाकै पिरलो
2020-05-19	Online Khabar (Nepali)	बाँकेको नरैनापुर अनिश्चितकालका लागि सिल
2020-05-28	Kantipur (Nepali)	बाँकेमा निषेधाज्ञा
2020-08-11	Online Khabar (Nepali)	बाँकेमा फेरि थपियो निषेधाज्ञा
2020-08-04	Online Khabar (Nepali)	बाँकेमा शनिबारसम्म लम्ब्याइयो निषेधाज्ञा
2020-05-05	Kantipur (Nepali)	बाँझो जग्गा खेती गर्न विपन्नलाई
2020-06-13	Kantipur (Nepali)	बाइकमा एक जना अव्यावहारिक
2020-05-15	Online Khabar (Nepali)	बागलुङमा संक्रमितको सम्पर्कमा आएका १२० जनाको स्वाब परीक्षण गरिँदै
2020-06-19	Online Khabar (Nepali)	बाजुराको क्वारेन्टिनमा आत्महत्या गरेको ६ दिनपछि कोरोना पुष्टि
2020-07-02	online Khabar (Nepali)	बारपाक सुलीकोटमा स्वास्थ्यकर्मी पठाउन प्रदेश सांसद दवाडीको माग
2020-08-31	Online Khabar (Nepali)	बारबरा फाउन्डेसनले स्वास्थ्यकर्मी र सुरक्षाकर्मीलाई सम्मान गर्ने
2020-07-06	Online Khabar (Nepali)	बारामा मास्क प्रयोग नगर्दा १०२ जनाले तिरे जरीवाना
2020-04-29	Online Khabar (Nepali)	बारामा संक्रमित भेटिएको दुई गाउँ सिल
2020-08-11	Online Khabar (Nepali)	बारामा स्थानीय तहहरुले पीसीआर मेसिन खरिद गर्ने
2020-07-03	Kantipur (Nepali)	बालबालिका उच्च जोखिममा छन्'
2020-05-08	Kantipur (Nepali)	बालबालिकालाई मानसिक दबाबमा नपार'
2020-07-15	Kantipur (Nepali)	बाल्ट्राको ह्यान्ड सेनिटाइजर डिस्पेन्सर
2020-05-21	Kantipur (Nepali)	बिनातयारी निजीलाई कोरोना परीक्षण अनुमति

Date of publication (yyyy/mm/dd)	Name of news media (select from drop-down menu)	Title of news/headline
2020-04-04	Online Khabar (Nepali)	बिरामीको उपचार नगर्ने निजी अस्पताललाई कारबाही गर : नागरिक समाज
2020-07-28	Kantipur (Nepali)	बिहारमा संक्रमण बढ्यो, नेपालमा झन् जोखिम
2020-08-15	Kantipur (Nepali)	बुटवलमा आधा घण्टामै कोरोना परीक्षण
2020-08-24	Online Khabar (Nepali)	बुटवलमा जनशक्ति अभाव, भैरहवामा आईसीयू वेड अलपत्र
2020-03-21	Kantipur (Nepali)	बुटवलमा पनि कोरोना अस्पताल
2020-08-12	Online Khabar (Nepali)	बुटवलमा भदौ ५ गतेसम्म लकडाउन
2020-08-31	Online Khabar (Nepali)	बुटवलमा स्वास्थ्य सामग्रीमा कालोबजारी
2020-08-14	Online Khabar (Nepali)	बुढानीलकण्ठ नगरपालिका– १० मा ५ जना भन्दा बढी भेला हुन प्रतिबन्ध
2020-05-18	Online Khabar (Nepali)	बूढानीलकण्ठको तेङ्गिङ चोक सिल, तारकेश्वरमा प्रवेश निषेध
2020-05-18	Kantipur (Nepali)	बेथिति हटाउन निर्मम बन्ने अवसर
2020-03-06	Kantipur (Nepali)	बेन्च–बारको दूरी बढ्यो
2020-06-15	Kantipur (Nepali)	बेपरवाह घरबाहिर
2020-05-29	Kantipur (Nepali)	बेरोजगारलाई कृषिमा लगाउन भूमि बैंक
2020-06-25	Kantipur (Nepali)	बेलायतबाट फर्किए १४६ नेपाली
2020-07-17	Kantipur (Nepali)	बैंक किस्ता तिर्ने समय ३ महिनादेखि १ वर्ष थपिँदै
2020-06-13	Kantipur (Nepali)	बैंकमा आउन थाल्यो कर्जा माग
2020-07-06	Kantipur (Nepali)	बैक र बीमा कम्पनीहरु शाखा घटाउने रणनीतिमा
2020-05-05	Kantipur (Nepali)	ब्याजदर घटाउन माग
2020-03-06	Kantipur (Nepali)	भक्तपुर महोत्सव स्थगित
2020-05-14	Kantipur (Nepali)	भक्तपुरमा घर बाहिर निस्कन निषेध
2020-07-08	Online Khabar (Nepali)	भक्तपुरमा मास्क नलगाई हिँड्ने ४८ जना पक्राउ
2020-08-14	Online Khabar (Nepali)	भरतपुर अस्पताल आएका १३ जनामा कोरोना, नर्स पनि संक्रमित भएपछि आकस्मिक कक्ष सिल
2020-08-16	Online Khabar (Nepali)	भरतपुर क्यान्सर अस्पतालमा अत्यावश्यकबाहेकका सेवा बन्द
2020-06-11	Online Khabar (Nepali)	भरतपुर महानगरका वडा धमाधम 'सिल', बैंकसमेत बन्द गराइयो
2020-08-14	Online Khabar (Nepali)	भरतपुर महानगरमा एकसाता लकडाउन
2020-08-14	Online Khabar (Nepali)	भरतपुरमा अत्यावश्यक बाहेकका पसल र गाडी बन्द
2020-06-18	Kantipur (Nepali)	भर्चुअल कक्षामा प्रदेशको प्राथमिकता
2020-05-04	Kantipur (Nepali)	भर्चुअल पठनपाठनका अप्ठ्यारा
2020-08-30	Online Khabar (Nepali)	भर्चुअल बैठकमा गृहमन्त्रीको गुनासो : ब्यारेक र जेलमा कोरोना बढ्यो
2020-03-11	Kantipur (Nepali)	भाइरस फैलिए चीनले सहयोग गर्ने
2020-05-25	Kantipur (Nepali)	भाइरसभन्दा खतरनाक क्वारेन्टिन
2020-06-03	Kantipur (Nepali)	भाइरसभन्दा लकडाउन खतरनाक
2020-07-12	Kantipur (Nepali)	भाडा र ऋणको बोझले ७० होटल बन्द
2020-05-17	Online Khabar (Nepali)	भारत तथा तेस्रो मुलकबाट हरेक दिन ६ हजारको उद्धार गर्ने प्रस्ताव
2020-03-20	Kantipur (Nepali)	भारततर्फ सीमा 'सिल' गर्ने प्रस्ताव
2020-05-18	Kantipur (Nepali)	भारतद्वारा पीसीआर किट सहयोग
2020-06-17	Online Khabar (Nepali)	भारतबाट आउनेको चाप घट्यो, तर सीमामा अझै हुन्न परीक्षण
2020-08-07	Online Khabar (Nepali)	भारतबाट आएकाहरू सोझै घर जान थाले
2020-03-06	Kantipur (Nepali)	भारतबाट गरिने चिनियाँ सामग्री आयात घट्यो
2020-06-08	Kantipur (Nepali)	भारतबाट घर फर्कनै सास्ती
2020-06-09	Kantipur (Nepali)	भारतबाट फर्किए साढे ७ लाख
2020-05-31	Kantipur (Nepali)	भारतबाट भित्रिएकालाई सेनाले घर पठाउने

Date of publication (yyyy/mm/dd)	Name of news media (select from drop-down menu)	Title of news/headline
2020-05-12	Online Khabar (Nepali)	भारतबाट रातारात सीमामा ल्याइन्छन् नेपाली मजदुर
2020-03-30	Online Khabar (Nepali)	भारतबाट लाख बढी भित्रिए, हजार पनि छैनन् क्वारेन्टाइनमा
2020-05-23	Online Khabar (Nepali)	भारतबाट समूहमा आएका थिए सुर्खेतका ३ जना कोराना संक्रमित
2020-05-12	Online Khabar (Nepali)	भारतमा क्वारेन्टाइनमा बसेका दुई सय ४२ लाई नेपाल ल्याइयो
2020-07-19	Kantipur (Nepali)	भारतमा नेपाली कामदारको बिचल्ली
2020-04-30	Online Khabar (Nepali)	भारतमा रोकिएका नेपालीको बिहानैदेखि धमाधम उद्धार
2020-08-10	Kantipur (Nepali)	भारतमा रोजगारी गुमेपछि गुजारा मुस्किल
2020-08-10	Kantipur (Nepali)	भारतीय सेनाद्वारा १० भेन्टिलेटर सहयोग
2020-06-21	Kantipur (Nepali)	भीटीएम नहुँदा संकलन रोकियो
2020-05-27	Kantipur (Nepali)	भीटीएम सकिँदै, स्वाब संकलन सुस्त
2020-06-11	Kantipur (Nepali)	भीड कम गर्ने उपाय
2020-06-26	Kantipur (Nepali)	भीड कम गर्ने तरिका
2020-05-18	Kantipur (Nepali)	भीडभाडले संक्रमण फैलियो
2020-08-31	Online Khabar (Nepali)	भीम अस्पतालमा आईसीयू सञ्चालन
2020-08-21	Online Khabar (Nepali)	भुल्के मोडल सिकाउने मन्त्री विराटनगरमा निरीह !
2020-05-07	Online Khabar (Nepali)	भुल्केमा थप ३३५ जनाको स्वाब संकलन
2020-08-25	Online Khabar (Nepali)	भेन्टिलेटर र आईसीयूका लागि प्रत्येक प्रदेशलाई २ करोड
2020-08-10	Online Khabar (Nepali)	भेन्टिलेटरको अभावमै गुम्छ जीवन
2020-05-19	Kantipur (Nepali)	भेरी–बबईमा दुई अर्बको ठेक्का रद्द
2020-06-24	Kantipur (Nepali)	भैरहवाबाट भन्सार कारोबार बढ्यो
2020-04-13	Online Khabar (Nepali)	भोलिदेखि वीरगञ्जमै कोरोना परीक्षण हुन्छ : मुख्यमन्त्री राउतभोलिदेखि वीरगञ्जमै कोरोना परीक्षण हुन्छ : मुख्यमन्त्री राउत
2020-05-20	Kantipur (Nepali)	म थाकिसकें
2020-08-09	Online Khabar (Nepali)	मकवानपुरको बजार क्षेत्रमा सोमबार रातिदेखि निषेधाज्ञा
2020-08-31	Online Khabar (Nepali)	मकवानपुरमा निषेधाज्ञा हट्यो, विहान १० बजेसम्म बजार खुल्ने
2020-08-28	Online Khabar (Nepali)	मकवानपुरमा निषेधाज्ञाको स्वरुप परिवर्तन गरिने
2020-05-10	Kantipur (Nepali)	मजदुर–मालिक सहमति खोज्न डाकियो बैठक
2020-05-04	Kantipur (Nepali)	मजदुरलाई सधैं हेला
2020-06-05	Kantipur (Nepali)	मजदुरले कहिले पाउँछन् पैसा?
2020-05-02	Kantipur (Nepali)	मजदुरले चैतकै तलब पाएनन्
2020-04-30	Online Khabar (Nepali)	मध्य तराईमा संक्रमितको संख्या बढेपछि पीसीआर परीक्षणमा तीब्रता
2020-03-20	Kantipur (Nepali)	मध्यपुरमा ५० शय्याको क्वारेन्टाइन
2020-06-04	Kantipur (Nepali)	मध्यम वर्ग डिप्रेसनमा
2020-05-28	Kantipur (Nepali)	मध्यम व्यवसायमा आईएफसीले सघाउने
2020-08-26	Online Khabar (Nepali)	मनाङका स्वास्थ्य प्रमुखलाई नै संक्रमण, जिल्ला अस्पताल सिल
2020-08-30	Online Khabar (Nepali)	मन्त्रालयको निर्देशन विपरीत विद्यालयमा आइसोलेसन
2020-06-24	Kantipur (Nepali)	मन्त्रालयले पठाएको किट काम लागेन
2020-05-23	Kantipur (Nepali)	मन्त्रालयले माग्यो थप ९ अर्ब
2020-05-02	Kantipur (Nepali)	मरकले राजालाई पनि छोडेन
2020-08-16	Kantipur (Nepali)	मरेपछि नागरिक देख्ने सरकार
2020-06-29	Kantipur (Nepali)	मल न मजदुर
2020-06-13	Kantipur (Nepali)	मलका लागि भौंतारिँदै किसान
2020-05-22	Kantipur (Nepali)	मलर सदाको भोक
2020-06-19	Kantipur (Nepali)	मलेसियाबाट कामदार ल्याउन सुरु

Date of publication (yyyy/mm/dd)	Name of news media (select from drop-down menu)	Title of news/headline
2020-04-29	Kantipur (Nepali)	महाकाली तरेर भारत गएका चार नेपाली नियन्त्रणमा
2020-06-06	Kantipur (Nepali)	महानगरले बनायो छानबिन समिति
2020-03-13	Kantipur (Nepali)	महामारी घोषणासँगै सतर्कता बढाइयो
2020-08-02	Kantipur (Nepali)	महामारी नियन्त्रणबाहिर
2020-08-19	Online Khabar (Nepali)	महामारी नियन्त्रणमा असहयोग गर्ने स्वास्थ्य संस्थाविरुद्ध अदालत जाने सरकारको तयारी
2020-05-04	Kantipur (Nepali)	महामारीको लैंगिक पाटो
2020-08-19	Kantipur (Nepali)	महामारीको विभाजन रेखा
2020-08-30	Kantipur (Nepali)	महामारीमा अनुशासनहीन राजनीतिक संस्कार !
2020-06-13	Kantipur (Nepali)	महामारीमा आदिवासी
2020-08-28	Kantipur (Nepali)	महामारीमा चुनावको झल्को !
2020-08-20	Kantipur (Nepali)	महामारीमा तीजको सन्देश
2020-05-02	Kantipur (Nepali)	महामारीमा नेपाली राजनीतिको रहस्यवाद
2020-06-25	Kantipur (Nepali)	महामारीमा मानसिक स्वास्थ्य
2020-05-03	Kantipur (Nepali)	महामारीमा स्थानीय सरकारसँग अपेक्षा
2020-07-27	Kantipur (Nepali)	महामारीले जाँच्दै छ शासकहरूको योग्यता
2020-06-21	Kantipur (Nepali)	महामारीले पनि नसिकाएको पाठ !
2020-05-06	Kantipur (Nepali)	महिन्द्राको घरमै सेवा
2020-05-18	Kantipur (Nepali)	महिला ओझेलमा परे
2020-06-17	Online Khabar (Nepali)	महोत्तरीको सम्सी गाउँपालिका ७२ घण्टाका लागि सिल
2020-05-21	Kantipur (Nepali)	मानव विकास वृद्धिमा ३० वर्षयताकै कमी आउन सक्छ : यूएनडीपी
2020-05-30	Kantipur (Nepali)	मानव सभ्यतामाथि बजेको ॅसाइरन’ !
2020-07-16	Kantipur (Nepali)	मानसिक तनाव भोग्दै स्वास्थ्यकर्मी
2020-07-05	Kantipur (Nepali)	मानसिक समस्या खुलेर भनौं’
2020-08-23	Kantipur (Nepali)	मानिसलाई विषादी छर्किनु अनुचित
2020-08-03	Kantipur (Nepali)	मास्क अनिवार्य
2020-07-06	Online Khabar (Nepali)	मास्क नलगाई घरबाहिर निस्किए प्रहरीले पक्राउ गर्ने
2020-07-07	Kantipur (Nepali)	मास्क नलगाई निस्किए पक्राउ
2020-08-17	Online Khabar (Nepali)	मास्क नलगाउनेलाई कारबाही गर्न खोज्दा प्रहरीमाथि नै कुटपिट
2020-07-10	Kantipur (Nepali)	मास्क नलगाए जरिवाना
2020-03-10	Kantipur (Nepali)	मास्क निःशुल्क वितरण
2020-05-03	Kantipur (Nepali)	मास्क बनाउने उद्योगलाई भ्याइनभ्याइ
2020-08-22	Kantipur (Nepali)	मास्क लगाइरहेको मार्क्सवाद
2020-07-03	Kantipur (Nepali)	मास्क लगाए पनि असुरक्षित
2020-06-19	Kantipur (Nepali)	मास्क लाउनै मुस्किल
2020-08-07	Kantipur (Nepali)	मास्कको मूल्य कति ?
2020-02-28	Kantipur (Nepali)	मास्कमा मनपरी गर्नेलाई छैन नियमन
2020-08-09	Online Khabar (Nepali)	मुख्यमन्त्री राउतले भेटे उपप्रधानमन्त्री, कोरोना रोकथामको विशेष प्याकेज ल्याउन आग्रह
2020-05-09	Online Khabar (Nepali)	मुम्बईबाट लुकिछिपी आएका थिए कपिलवस्तुका संक्रमित
2020-05-24	Kantipur (Nepali)	मुलुक फर्किनेको जमुनाहामा धुइरो
2020-07-10	Kantipur (Nepali)	मेरुदण्डको स्याहार

Date of publication (yyyy/mm/dd)	Name of news media (select from drop-down menu)	Title of news/headline
2020-08-30	Online Khabar (Nepali)	मेहतर उत्थान समाजले दियो अति विपन्नलाई राहत सामग्री
2020-06-17	Kantipur (Nepali)	मै खाऊँ, मै लाऊँ, मरिरहून् जनता !
2020-06-14	Kantipur (Nepali)	मोटरसाइकलमा एक जना मात्रै : अव्यावहारिक र औचित्यहीन
2020-03-21	Kantipur (Nepali)	मोटरसाइकलमै सामान ढुवानी
2020-08-07	Kantipur (Nepali)	मोटोपना घटाए जोखिम कम
2020-08-09	Online Khabar (Nepali)	मोरङका १० स्थानीय तहमा लकडाउन
2020-04-17	Online Khabar (Nepali)	मोरङकी युवतीमा देखियो कोरोना एन्टीबडी पोजेटिभ
2020-03-12	Online Khabar (Nepali)	मोरङको टाँडी र सुनसरीको श्रीलंका टप्पुमा क्वारेन्टाइन
2020-07-14	Kantipur (Nepali)	मौद्रिक नीतिले लिनुपर्ने नीति
2020-05-05	Kantipur (Nepali)	यथास्थितिमा 'खुकुलो पार्दा जोखिम'
2020-06-17	Kantipur (Nepali)	यसकारण वैकल्पिक सिकाइ प्रणाली
2020-06-14	Kantipur (Nepali)	यसरी खुल्दै छन् कार्यालय
2020-05-11	Kantipur (Nepali)	यसरी लडेँ कोरोनासँग
2020-06-07	Kantipur (Nepali)	यात्रु चार्टर उडानमा रोक
2020-08-11	Kantipur (Nepali)	यात्रु बोकेर भित्रिन्छन् एम्बुलेन्स
2020-07-09	Kantipur (Nepali)	युनिलिभरले ल्यायो राहत
2020-05-19	Kantipur (Nepali)	युवालाई नगरको बिनाधितो स्वरोजगार कर्जा
2020-06-10	Kantipur (Nepali)	यूएनडीपीले सुरु गर्‍यो जीविकोपार्जन पुनरुत्थान कार्यक्रम
2020-06-04	Kantipur (Nepali)	यो देश मजदुरको होइन रहेछ !
2020-07-06	Kantipur (Nepali)	यो विषाक्त मौनताले सबैलाई पिरोल्नेछ
2020-05-24	Kantipur (Nepali)	यो समय कृषिको पनि हो
2020-05-10	Kantipur (Nepali)	योजनाबद्ध कृषि व्यवसाय जरुरी
2020-03-13	Kantipur (Nepali)	रक्तदानमा सचेत हुन अपिल
2020-03-15	Kantipur (Nepali)	रगत जुटाउनै हम्मे
2020-03-02	Kantipur (Nepali)	रसुवागढी सुनसान
2020-05-15	Kantipur (Nepali)	राजधानी छिर्ने नाकामा सेना खटाउन सिफारिस
2020-07-05	Kantipur (Nepali)	राजधानी संक्रमणको उच्च जोखिममा
2020-08-23	Kantipur (Nepali)	राजधानीमा आगामी साताबाट संक्रमण घट्ने विज्ञको दाबी
2020-07-22	Online Khabar (Nepali)	राजविराजमा अनिश्चितकालका लागि निषेधाज्ञा
2020-05-05	Kantipur (Nepali)	राजस्व कम भएपछि ऋण र सहायतामा जोड
2020-05-12	Kantipur (Nepali)	रातारात दशगजामा ल्याएर छाडिन्छ
2020-08-21	Online Khabar (Nepali)	राति १२ बजेदेखि बिहान ९ बजेसम्म कालीमाटी र बल्खु तरकारी बजार खुल्ने
2020-06-10	Kantipur (Nepali)	राधादेवीहरूको चीत्कार कसले सुन्ला?
2020-04-25	Online Khabar (Nepali)	राप्ती स्वास्थ्य प्रतिष्ठानमा पीसीआर परीक्षण सुरु
2020-05-20	Online Khabar (Nepali)	राप्ती स्वास्थ्य विज्ञान प्रतिष्ठानको ओपीडी बन्द गर्ने निर्णयको विरोध
2020-05-20	Online Khabar (Nepali)	राप्ती स्वास्थ्य विज्ञान प्रतिष्ठानमा पनि कोरोनाको उपचार, ओपीडी बन्द
2020-04-10	Online Khabar (Nepali)	रामेछापका सबै स्थानीय तहमा कोरोना रोकथाम कोष
2020-04-14	Online Khabar (Nepali)	रामेछापमा र्‍यापिड टेस्ट सुरु, पहिलो रिपोर्ट नेगेटिभ
2020-08-11	Kantipur (Nepali)	राहत खोइ?
2020-05-06	Kantipur (Nepali)	राहत प्याकेज ल्याउन सुझाव
2020-05-21	Kantipur (Nepali)	राहतको खोजीमा 'राइडर'
2020-06-19	Kantipur (Nepali)	राहतको साटो काम
2020-05-18	Kantipur (Nepali)	राहतमा भेदभाव नगर्नू'

Date of publication (yyyy/mm/dd)	Name of news media (select from drop-down menu)	Title of news/headline
2020-05-21	Kantipur (Nepali)	रिचार्जरको कम मूल्यमै मास्क
2020-06-17	Kantipur (Nepali)	रित्तिँदै सरकारी खाद्य भण्डारण
2020-06-14	Kantipur (Nepali)	रिपोर्ट नआएपछि प्रहरीमाथि ढुंगामुढा
2020-06-15	Kantipur (Nepali)	रिपोर्ट माग्दा प्रहरीको लाठी
2020-02-07	Kantipur (Nepali)	रुघाखोकी बिरामीको र्‍यालको नमुना काठमाडौं पठाइयो
2020-08-23	Online Khabar (Nepali)	रुपन्देहीमा भदौ १४ सम्म निषेधाज्ञा थपियो
2020-08-30	Online Khabar (Nepali)	रुपन्देहीमा भदौ २० सम्म निषेधाज्ञा
2020-08-30	Online Khabar (Nepali)	रुपन्देहीमा भदौ २० सम्म निषेधाज्ञा सिफारिस
2020-08-18	Online Khabar (Nepali)	रुपन्देहीमा भदौ ७ सम्म निषेधाज्ञा
2020-08-16	Online Khabar (Nepali)	रुपन्देहीस्थित नेपाल आयल निगमका १० कर्मचारीमा कोरोना
2020-05-13	Kantipur (Nepali)	रूपन्देहीका पहिलो संक्रमित डिस्चार्ज
2020-08-18	Kantipur (Nepali)	रेमडेसिभिरमा कालोबजारी
2020-08-25	Kantipur (Nepali)	रेमिट्यान्समा देखिएन कोरोना प्रभाव
2020-04-30	Online Khabar (Nepali)	रेमिट्यान्समा धक्का : एकै वर्ष १ खर्ब ६३ अर्ब घट्ने
2020-05-03	Kantipur (Nepali)	रेलमार्गमा पनि काम निरन्तर
2020-05-10	Online Khabar (Nepali)	रोकिएन सीमाबाट नेपाल प्रवेश गर्ने क्रम
2020-04-27	Kantipur (Nepali)	रोकियो यार्चागुम्बु संकलन
2020-06-01	Kantipur (Nepali)	रोगको भन्दा भोकको डर
2020-08-30	Kantipur (Nepali)	रोगभन्दा भोकको पीर
2020-06-20	Kantipur (Nepali)	रोजगारी र पढाइमा विदेश जान खुला
2020-07-03	Online Khabar (Nepali)	रोल्पामा आत्महत्या गरेका युवकमा कोरोना संक्रमण पुष्टि
2020-08-26	Online Khabar (Nepali)	रौतहटमा एक घर एक परिक्षण अभियान
2020-08-23	Online Khabar (Nepali)	रौतहटमा निषेधाज्ञा एक साता थप
2020-08-05	Online Khabar (Nepali)	रौतहटमा शनिबारदेखि निषेधाज्ञा
2020-04-16	Online Khabar (Nepali)	र्‍यापिड टेस्ट नेगेटिभ आउँदैमा कोरोना संक्रमण छैन भन्न मिल्दैन : स्वास्थ्य मन्त्रालय
2020-04-13	Online Khabar (Nepali)	र्‍यापिड टेस्टले दुर्घटना त निम्त्याइरहेको छैन ?
2020-04-15	Online Khabar (Nepali)	र्‍यापिडमा पोजेटिभ, पीसीआरमा नेगेटिभ : कोरोना सर्दैन, सारिसकेको हुन सक्छ
2020-05-17	Kantipur (Nepali)	लकडाउन : अख्तियारलाई निदाउने राम्रो बहाना
2020-05-18	Kantipur (Nepali)	लकडाउन १५ दिन लम्बियो
2020-05-04	Kantipur (Nepali)	लकडाउन कसरी खोल्ने?
2020-06-01	Kantipur (Nepali)	लकडाउन कहिलेसम्म?
2020-05-04	Kantipur (Nepali)	लकडाउन खुकुलिँदा विकास निर्माण सुरु
2020-05-06	Kantipur (Nepali)	लकडाउन खुकुलो पारे जोखिम : सरकार
2020-05-05	Kantipur (Nepali)	लकडाउन खुकुलो पार्ने तयारी
2020-04-30	Kantipur (Nepali)	लकडाउन खुकुलो बनाउन मोडालिटी बन्दै
2020-06-09	Kantipur (Nepali)	लकडाउन खुकुलो बनाउने गृहकार्य अन्तिम चरणमा
2020-06-05	Kantipur (Nepali)	लकडाउन खुकुलो बनाउने तीन ढाँचा
2020-06-16	Kantipur (Nepali)	लकडाउन खुलेको ३० दिनपछि कर बुझाउने आदेशकै निरन्तरता
2020-07-23	Kantipur (Nepali)	लकडाउन खुल्यो, जोखिम बढ्यो
2020-06-11	Kantipur (Nepali)	लकडाउन खु ुकुलो, एसईई नहुने
2020-07-12	Kantipur (Nepali)	लकडाउन छ कि छैन?
2020-06-03	Kantipur (Nepali)	लकडाउन जिन्दगी

Date of publication (yyyy/mm/dd)	Name of news media (select from drop-down menu)	Title of news/headline
2020-05-31	Kantipur (Nepali)	लकडाउन जेठ ३२ सम्म
2020-05-07	Kantipur (Nepali)	लकडाउन जेठ ५ सम्म
2020-05-06	Online Khabar (Nepali)	लकडाउन जेठ ५ सम्म लम्ब्याइयो, विकास निर्माण र उद्योग सशर्त खुला
2020-05-15	Online Khabar (Nepali)	लकडाउन थप्ने सरकारको तयारी, जनस्वाथ्य विज्ञहरुसँग छलफल
2020-05-11	Kantipur (Nepali)	लकडाउन पालना भएन
2020-06-01	Kantipur (Nepali)	लकडाउन पुनर्विचार गर्न निजी क्षेत्रको माग
2020-05-25	Kantipur (Nepali)	लकडाउन रहिरहे आर्थिक वृद्धि ऋणात्मक
2020-05-23	Kantipur (Nepali)	लकडाउन लम्ब्याउँदैमा आयातित केस रोक्न सक्दैनौं’
2020-04-27	Kantipur (Nepali)	लकडाउन वैशाख २५ सम्म
2020-06-07	Kantipur (Nepali)	लकडाउन सजिलो बनाऊ
2020-05-18	Kantipur (Nepali)	लकडाउन सत्ताकवच बन्नु हुँदैन
2020-06-12	Kantipur (Nepali)	लकडाउन सहज बनाइएको हो, कोरोनाकाल सकिएको होइन
2020-05-12	Online Khabar (Nepali)	लकडाउनका बेला भारतबाट आएका थिए कपिलवस्तुका १० संक्रमित
2020-06-10	Kantipur (Nepali)	लकडाउनको ओझेलमा महिला हिंसा
2020-06-02	Kantipur (Nepali)	लकडाउनको ढाँचा फेर्ने तयारी
2020-05-27	Kantipur (Nepali)	लकडाउनको स्वरूप फेरौं
2020-06-04	Kantipur (Nepali)	लकडाउनको स्वरूप फेरौं, परीक्षण बढाऔं'
2020-06-10	Kantipur (Nepali)	लकडाउनपछि ९५ जनाको हत्या
2020-06-20	Kantipur (Nepali)	लकडाउनपछि एक दिन
2020-05-24	Kantipur (Nepali)	लकडाउनपछि पूर्वाधारमा ६५ खर्च
2020-05-03	Kantipur (Nepali)	लकडाउनमा १८ हजार सवारी नियन्त्रणमा
2020-08-06	Kantipur (Nepali)	लकडाउनमा ६१ प्रतिशत उद्योग बन्द
2020-05-31	Kantipur (Nepali)	लकडाउनमा आत्महत्या दर बढ्दै
2020-07-30	Kantipur (Nepali)	लकडाउनमा आत्महत्या र यसले उब्जाएका प्रश्न
2020-08-05	Online Khabar (Nepali)	लकडाउनमा एक चौथाइ कर्मचारीले रोजगारी गुमाए, सबैभन्दा धेरै होटल क्षेत्रका
2020-05-07	Kantipur (Nepali)	लकडाउनमा कसरी चल्छ संसद् ?
2020-07-19	Kantipur (Nepali)	लकडाउनमा कौसी खेती
2020-05-07	Kantipur (Nepali)	लकडाउनमा खेलाडी
2020-03-25	Online Khabar (Nepali)	लकडाउनमा जनताबाट सरकारले गरेको अपेक्षा के हो ? (प्रदीप ज्ञवालीसँग भिडियो वार्ता)
2020-05-11	Kantipur (Nepali)	लकडाउनमा थपियो ९१ अर्ब निक्षेप
2020-08-26	Kantipur (Nepali)	लकडाउनमा पनि केही आयोजनाको उल्लेख्य प्रगति
2020-05-05	Kantipur (Nepali)	लकडाउनमा राजनीतिक नौटंकी
2020-06-18	Kantipur (Nepali)	लकडाउनमा लिए सांसदले सवारी भत्ता
2020-05-18	Kantipur (Nepali)	लकडाउनमा लिसेन्कोको सम्झना
2020-06-14	Kantipur (Nepali)	लकडाउनमा लुलो सरकार
2020-05-21	Kantipur (Nepali)	लकडाउनमा शुल्क बुझाउन ताकेता
2020-05-20	Kantipur (Nepali)	लकडाउनमा सवारी बिमा शुल्क छुट
2020-05-31	Kantipur (Nepali)	लकडाउनमात्रै समाधान होइन
2020-06-03	Kantipur (Nepali)	लकडाउनमै कर तिर्न ताकेता
2020-07-23	Online Khabar (Nepali)	लकडाउनयता तीन सय शिक्षकको गयो जागिर
2020-05-05	Kantipur (Nepali)	लकडाउनले अर्थतन्त्र ठप्प

Date of publication (yyyy/mm/dd)	Name of news media (select from drop-down menu)	Title of news/headline
2020-06-04	Kantipur (Nepali)	लकडाउनले आजित व्यवसायी
2020-04-17	Online Khabar (Nepali)	लकडाउनले उपचार पनि महँगो हुँदोरहेछ ...
2020-05-27	Kantipur (Nepali)	लकडाउनले खुम्च्यायो लगानी
2020-06-04	Kantipur (Nepali)	लकडाउनले गाँसै खोसिदियो
2020-06-04	Kantipur (Nepali)	लकडाउनले ज्यान गुमाउँदै 'आमा' हरू
2020-06-02	Kantipur (Nepali)	लकडाउनले निकास दिँदैन
2020-06-02	Kantipur (Nepali)	लकडाउनले बनायो मजदुरलाई ॅमाग्ने’
2020-07-26	Kantipur (Nepali)	लकडाउनले बैंकको ४० अर्ब ब्याज उठेन
2020-06-01	Kantipur (Nepali)	लकडाउनले बोटमै सुके फूल
2020-05-03	Kantipur (Nepali)	लकडाउनले मिर्गौला रोगी भोकभोकै
2020-07-02	Kantipur (Nepali)	लकडाउनले रोकियो सेजको गति
2020-08-12	Kantipur (Nepali)	लक्षण देखिए पनि ३ दिनदेखि अस्पताल नपाएका संक्रमितको प्रश्न मरेपछि मात्रै रेस्पोन्स गर्नुहुन्छ?’
2020-07-10	Kantipur (Nepali)	लक्षण नदेखिएपछि परीक्षण ठप्प
2020-06-07	Kantipur (Nepali)	लक्षण नभएका संक्रमित घरकै आइसोलेसनमा
2020-08-22	Kantipur (Nepali)	लक्षण भएका २५% संक्रमितलाई अक्सिजन जरुरी
2020-08-04	Online Khabar (Nepali)	लक्षणसहितका संक्रमित देखिए एक महिना लकडाउन : मुख्यमन्त्री शाही
2020-07-16	Kantipur (Nepali)	लक्ष्यको आधा राजस्व
2020-08-08	Kantipur (Nepali)	लघुवित्तको ऋणले थलिए गाउँ
2020-08-09	Kantipur (Nepali)	लघुवित्तको साहु अवतार
2020-06-07	Kantipur (Nepali)	लर्को उस्तै
2020-05-13	Online Khabar (Nepali)	ललितपुरको आरोग्य फाउन्डेसन र निदान अस्पताल सिल
2020-05-07	Kantipur (Nepali)	लुकीछिपी छिर्न खोज्नेको सीमामा भीड
2020-08-16	Online Khabar (Nepali)	लुकीछिपी नेपाल प्रवेश पूर्ण रुपमा बन्द गर्ने प्रयासमा छौं : गृहमन्त्री
2020-05-24	Online Khabar (Nepali)	लुम्बिनी विकास कोषले कोरोना कोषलाई दियो १५ लाख
2020-08-12	Kantipur (Nepali)	लेबनानमा रोजगारी गुमाउँदै नेपाली
2020-05-15	Online Khabar (Nepali)	वडाध्यक्षले दिल्लीबाट छोरालाई ल्याए, क्वारेन्टाइनमा राखेनन्
2020-08-10	Online Khabar (Nepali)	वडाले पसल बन्द गर्न भन्यो, व्यापारीले टेरेनन्
2020-02-13	Kantipur (Nepali)	वाइडबडी शनिबार वुहान उड्ने
2020-05-27	Kantipur (Nepali)	वाग्मती प्रदेशमा स्वास्थ्य र कृषिलाई प्राथमिकता
2020-03-15	Kantipur (Nepali)	वाग्मती सफाइमा जागरण अभियान
2020-07-17	Kantipur (Nepali)	वायुसेवाका १४ संक्रमित फेरि सोल्टीमै
2020-08-28	Kantipur (Nepali)	विकल्प खोज्दै पार्टी प्यालेस व्यवसायी
2020-06-25	Kantipur (Nepali)	विकल्पमा सुतीको मास्क
2020-05-12	Kantipur (Nepali)	विचार नगरी निर्णय
2020-05-03	Kantipur (Nepali)	विज्ञ भन्छन्– ‘डिस्इन्फेक्टेन्ट टनेल कामै छैन’
2020-05-04	Kantipur (Nepali)	विज्ञ भन्छन्– ‘परीक्षण नबढाए लकडाउनको अर्थ छैन’
2020-06-04	Kantipur (Nepali)	विज्ञ भन्छन्– 'स्वास्थ्य संकटकालको हतारो नगरौं’
2020-05-30	Kantipur (Nepali)	विदेशबाट १० दिनभित्र २५ हजारलाई ल्याइने
2020-05-12	Kantipur (Nepali)	विदेशबाट आउन चाहने झन्डै ६ लाख
2020-07-20	Kantipur (Nepali)	विदेशबाट आउनेलाई पीसीआर अनिवार्य
2020-06-28	Kantipur (Nepali)	विदेशबाट आउनेलाई सिधै घर

Date of publication (yyyy/mm/dd)	Name of news media (select from drop-down menu)	Title of news/headline
2020-06-03	Kantipur (Nepali)	विदेशबाट आएकालाई यसरी पुर्‍याइन्छ घर
2020-05-29	Online Khabar (Nepali)	विदेशबाट उद्दार गर्ने कार्ययोजना तयार, पैसा तिरेर होटल क्वारेन्टिनमा बस्न पाइने
2020-08-24	Online Khabar (Nepali)	विदेशबाट उद्धार गरिने नेपालीलाई निशुल्क क्वारेन्टिनमा राख : सांसद संग्रौला
2020-05-31	Online Khabar (Nepali)	विदेशबाट उद्धार गरिनेको व्यवस्थापनमा सेना खटिने, १० दिनभित्र क्वारेन्टिन बनाइने
2020-06-01	Kantipur (Nepali)	विदेशबाट नेपालीलाई उद्धार गर्न दिनमा १० उडान
2020-07-22	Kantipur (Nepali)	विदेशबाट फर्किनेलाई होम क्वारेन्टाइन !
2020-04-06	Online Khabar (Nepali)	विदेशबाट हेटौंडा आएका ११ सहित १७ जनाको रिपोर्ट नेगेटिभ
2020-08-13	Online Khabar (Nepali)	विदेशमा अप्ठेरामा परेका नेपालीको अधिकार संरक्षण गर : मानव अधिकार आयोग
2020-08-27	Online Khabar (Nepali)	विदेशमा अप्ठेरोमा परेकालाई विना भेदभाव उद्धार गर्नुपर्छ : एनआरएनए
2020-07-17	Online Khabar (Nepali)	विदेशमा अलपत्र परेकालाई अब सरकारी खर्चमा उद्धार गरिने
2020-07-10	Kantipur (Nepali)	विदेशमा बिचल्ली
2020-03-10	Kantipur (Nepali)	विदेशी ह्वात्तै घटे
2020-02-12	Kantipur (Nepali)	विद्यार्थीलाई चीनबाट शनिबार ल्याइने
2020-03-11	Kantipur (Nepali)	विद्यार्थीलाई मास्क
2020-06-16	Kantipur (Nepali)	विद्यालय छिट्टै खुल्छन् : प्रधानमन्त्री
2020-05-24	Kantipur (Nepali)	विद्यालय शुल्क कसरी तिर्ने?
2020-06-25	Kantipur (Nepali)	विद्यालयका क्वारेन्टिन हटाऊ
2020-03-06	Kantipur (Nepali)	विद्युत् आयोजना बढी प्रभावित
2020-06-07	Kantipur (Nepali)	विद्युत् प्राधिकरणलाई हतार
2020-05-03	Kantipur (Nepali)	विपत्मा सामाजिक पूर्वाग्रह
2020-03-29	Online Khabar (Nepali)	विपन्न परिवारलाई तिलोत्तमा नगरपालिकाको खाद्यान्न सहयोग
2020-05-27	Kantipur (Nepali)	विपन्नलाई शुल्क लिएर परीक्षण नगराउनू ः सर्वोच्च
2020-03-02	Kantipur (Nepali)	विमानस्थलमा सबैको जाँच
2020-03-11	Kantipur (Nepali)	विमानस्थलमा हेल्थ डेस्क
2020-03-09	Kantipur (Nepali)	विमानस्थललाई चेम्बरको सहयोग
2020-06-15	Kantipur (Nepali)	विराटनगर र बागलुङमा प्रदर्शन
2020-08-10	Online Khabar (Nepali)	विराटनगरको एउटै वडामा ४७ संक्रमित, गुद्रीबजार सिल
2020-04-29	Online Khabar (Nepali)	विराटनगरमा कोरोना अस्पतालको फोहोर कसरी गरिन्छ विसर्जन ?
2020-08-28	Online Khabar (Nepali)	विराटनगरमा न्यूरो अस्पतालले सुरु गर्‍यो कोभिड उपचार
2020-08-02	Kantipur (Nepali)	विराटनगरमा परीक्षण बढाइयो
2020-07-31	Online Khabar (Nepali)	विराटनगरमा फेरि दुई साता लकडाउन
2020-08-24	Online Khabar (Nepali)	विराटनगरले किन्यो पीसीआर मेसिन, मेयरले दिए एक वर्षको सुविधा
2020-08-01	Online Khabar (Nepali)	विशेश्वरको आत्महत्या : कोरोनालाई जिते, गाउँ समाजसँग हारे !
2020-06-12	Kantipur (Nepali)	विश्व बैंकको ४५ करोड डलर ऋण स्वीकृत
2020-07-19	Kantipur (Nepali)	विस्तारकारी नीतिको कार्यान्वयन पाटो
2020-04-11	Online Khabar (Nepali)	वीर अस्पतालमा पनि सुरु भयो कोरोना परीक्षण
2020-07-02	Kantipur (Nepali)	वीर, पाटन र शिक्षणमा सशुल्क पीसीआर परीक्षण
2020-07-27	Online Khabar (Nepali)	वीरगञ्ज टू राजविराज : कोरोनाको 'साङ्लो' बन्न नपाओस्
2020-07-30	Online Khabar (Nepali)	वीरगञ्ज हेल्थ केयरलाई अस्थायी कोभिड अस्पताल बनाउन सिफारिस
2020-05-21	Online Khabar (Nepali)	वीरगञ्जको गण्डक अस्पतालमा सुरु भयो कोरोना उपचार

Date of publication (yyyy/mm/dd)	Name of news media (select from drop-down menu)	Title of news/headline
2020-04-18	Online Khabar (Nepali)	वीरगञ्जको गण्डक अस्पताललाई कोभिड अस्पताल बनाइने
2020-05-08	Online Khabar (Nepali)	वीरगञ्जको छपकैयामा थप २७२ जनाको थ्रोट स्वाब संकलन
2020-07-25	Online Khabar (Nepali)	वीरगञ्जमा आजबाट अनिश्चितकालीन निषेधाज्ञा जारी
2020-04-25	Online Khabar (Nepali)	वीरगञ्जमा उपचाररत ४ जनाको तेस्रो रिपोर्ट नेगेटिभ
2020-04-12	Online Khabar (Nepali)	वीरगञ्जमा कोरोना परीक्षण ल्याब नहुनु दुःखद : सभापति खड्का
2020-05-04	Kantipur (Nepali)	वीरगन्जमा एक संक्रमित थपिए
2020-08-10	Kantipur (Nepali)	वीरगन्जलाई हेर
2020-08-13	Online Khabar (Nepali)	वीरेन्द्रनगर नगरपालिका एक साता बन्द हुने
2020-02-01	Kantipur (Nepali)	वुहानका विद्यार्थी फिर्ता ल्याउन माग गर्दै नेपालले चीनलाई पठायो पत्र
2020-02-03	Online Khabar (Nepali)	वुहानबाट नेपालीको प्रश्न : सरकार ! हाम्रो मृत्यु पर्खिएको हो ?
2020-02-16	Kantipur (Nepali)	वुहानबाट फर्कने सोझै खरिपाटीमा
2020-03-11	Kantipur (Nepali)	वैदेशिक रोजगारीमा जाने घटे
2020-05-27	Kantipur (Nepali)	वैशाखमा ३९५ घट्यो रेमिट्यान्स
2020-07-02	Kantipur (Nepali)	व्यवसाय जोगाउन रेस्टुराँको होम डेलिभरी
2020-05-10	Kantipur (Nepali)	व्यवसायीका समस्या समाधान गर्छौं'
2020-08-12	Online Khabar (Nepali)	व्यवसायीलाई आफ्नै खर्चमा कोरोना परीक्षण गर्न सरकारको आग्रह
2020-03-22	Kantipur (Nepali)	शंका लागे ११८०
2020-03-23	Kantipur (Nepali)	शंकास्पद'लाई खोज्दै स्वास्थ्यकर्मी
2020-05-30	Kantipur (Nepali)	शक्ति सञ्चयका लागि सानो बजेट'
2020-04-04	Online Khabar (Nepali)	शनिबारबाट चितवनमै कोरोना भाइरस परीक्षण सुरु गर्ने तयारी
2020-06-26	Kantipur (Nepali)	शव व्यवस्थापनमा सधैं किचलो
2020-05-15	Kantipur (Nepali)	शिक्षण अस्पतालका ४९ स्वास्थ्यकर्मी क्वारेन्टाइनमा
2020-08-14	Online Khabar (Nepali)	शिक्षण अस्पतालका थप ४ जना स्वास्थ्यकर्मीमा संक्रमण
2020-08-04	Kantipur (Nepali)	शिक्षण अस्पतालको सफलता
2020-05-17	Kantipur (Nepali)	शिक्षणमा छैन चिकित्सक बस्ने क्वारेन्टाइन
2020-05-18	Kantipur (Nepali)	शिक्षणमा भद्रगोल
2020-08-11	Kantipur (Nepali)	शिक्षा मन्त्रालयको अलमल
2020-05-27	Kantipur (Nepali)	शिक्षामा अनलाइन कक्षालाई प्राथमिकता
2020-05-10	Kantipur (Nepali)	शुल्क तिरेर डायलसिस
2020-05-22	Kantipur (Nepali)	शुल्क लिएर परीक्षण नहोस्
2020-07-02	Kantipur (Nepali)	संकटबाट निस्किन अर्को बजेट विचार गरौं'
2020-05-16	Kantipur (Nepali)	संकटमा नागरिकको सीमाबोध
2020-06-26	Kantipur (Nepali)	संकटमा निस्फिक्री सरकार
2020-07-16	Kantipur (Nepali)	संकटमा पनि असारे भुक्तानी सीमाभन्दा बढी
2020-07-09	Kantipur (Nepali)	संकटमा यातायात व्यवसायी
2020-05-15	Kantipur (Nepali)	संकटमा सम्भावनाको घेरा
2020-08-25	Kantipur (Nepali)	संकटमाथि अचाक्ली मूल्यवृद्धि
2020-06-03	Kantipur (Nepali)	संकटसँग जुध्न छाडेर सरुवा
2020-05-25	Kantipur (Nepali)	संक्रमण ४२ जिल्लामा
2020-08-06	Kantipur (Nepali)	संक्रमण तीव्र भए भेन्टिलेटर र आईसीयू पुग्दैन'
2020-05-07	Kantipur (Nepali)	संक्रमण तेस्रो चरणमा तर ट्रेसिङ पहिलो चरणमै
2020-05-14	Kantipur (Nepali)	संक्रमण दोस्रो चरणमै : विज्ञ

Date of publication (yyyy/mm/dd)	Name of news media (select from drop-down menu)	Title of news/headline
2020-05-29	Kantipur (Nepali)	संक्रमण नियन्त्रण र उपचारमा प्राथमिकता
2020-03-10	Kantipur (Nepali)	संक्रमण फैलिए कसरी थेग्ने?
2020-05-18	Kantipur (Nepali)	संक्रमण बढेसँगै विद्यार्थी अन्योलमा
2020-08-11	Kantipur (Nepali)	संक्रमण बढ्दा आत्तिए प्रदेश र स्थानीय तह
2020-07-03	Kantipur (Nepali)	संक्रमण बढ्दो
2020-08-25	Kantipur (Nepali)	संक्रमण बढ्यो, ट्रेसिङ र टेस्टिङ बढेन
2020-06-09	Kantipur (Nepali)	संक्रमण र डरबीच पसल खोल्छन् व्यवसायी
2020-06-09	Kantipur (Nepali)	संक्रमण रोक्न असफल : कांग्रेस
2020-05-26	Kantipur (Nepali)	संक्रमण रोक्न कठिन
2020-06-13	Kantipur (Nepali)	संक्रमणका हटस्पटको साक्षी हुँदा
2020-05-14	Kantipur (Nepali)	संक्रमणको उच्च जोखिममा पुरुष
2020-08-25	Kantipur (Nepali)	संक्रमणको उच्च जोखिममा बालबालिका
2020-06-29	Kantipur (Nepali)	संक्रमणको चपेटामा 'फ्रन्टलाइनर'
2020-08-28	Online Khabar (Nepali)	संक्रमणको जोखिममा उपत्यका, निषेधाज्ञा लगाएर के गर्दैछ सरकार ?
2020-04-24	Kantipur (Nepali)	संक्रमणको जोखिममा कारागार
2020-05-21	Kantipur (Nepali)	संक्रमणको स्रोत क्वारेन्टिन
2020-05-03	Kantipur (Nepali)	संक्रमणपछि गाउँ र सीमामा विशेष निगरानी
2020-02-15	Kantipur (Nepali)	संक्रमणबाट बच्न आयुर्वेद
2020-07-06	Kantipur (Nepali)	संक्रमणबीच अस्पताल कसरी चलाउने?
2020-08-02	Kantipur (Nepali)	संक्रमणमुक्त हुँदा पनि छिःछिः
2020-05-24	Kantipur (Nepali)	संक्रमित ओसार्न फोहोर बोक्ने ट्र्याक्टर !
2020-05-02	Kantipur (Nepali)	संक्रमित क्षेत्रकी गर्भवती अस्पतालबाट निकाला
2020-05-15	Kantipur (Nepali)	संक्रमित क्षेत्रबाट आएकामाथि छैन चासो
2020-06-15	Kantipur (Nepali)	संक्रमित क्षेत्रबाट आएकालाई बिनापरीक्षण फर्काइँदै
2020-06-10	Kantipur (Nepali)	संक्रमित गर्भवतीको शल्यक्रिया
2020-05-15	Kantipur (Nepali)	संक्रमित चढेको ट्रक खोजिँदै
2020-02-29	Kantipur (Nepali)	संक्रमित देशबाट निर्बाध नेपाल प्रवेश
2020-06-05	Kantipur (Nepali)	संक्रमित बढेपछि कोरोना बिमा स्थगित
2020-05-20	Kantipur (Nepali)	संक्रमित बसेकै क्वारेन्टाइनमा छैन परीक्षण
2020-05-26	Online Khabar (Nepali)	संक्रमित भेटिनेवित्तिकै सिल नगर्न स्वास्थ्य मन्त्रालयको आग्रह
2020-05-31	Kantipur (Nepali)	संक्रमित रातभर अस्पतालबाहिरै
2020-08-28	Online Khabar (Nepali)	संक्रमित स्वास्थ्यकर्मीले नै गर्छन् कोरोनाका बिरामीको उपचार
2020-08-16	Online Khabar (Nepali)	संक्रमित हुँदा मैले ठूलो अपराध गरेजस्तो गरियो'
2020-07-03	Kantipur (Nepali)	संक्रमितको अनुगमन भएन
2020-08-07	Online Khabar (Nepali)	संक्रमितको उपचार गर्न मन्त्रीकै फोर्स
2020-04-07	Online Khabar (Nepali)	संक्रमितको उपचार गर्नेको ज्यान गए प्रदेश १ सरकारले ५० लाख दिने
2020-08-04	Kantipur (Nepali)	संक्रमितको खाजामा कीरा
2020-05-22	Kantipur (Nepali)	संक्रमितको शव ओसार्नेलाई ५० हजार
2020-06-01	Kantipur (Nepali)	संक्रमितलाई गोठमा राखियो
2020-08-30	Kantipur (Nepali)	संक्रमितलाई पाइलैपिच्छे सास्ती
2020-06-02	Kantipur (Nepali)	संक्रमितलाई लक्षण देखिए मात्रै अस्पताल
2020-06-05	Kantipur (Nepali)	संक्रमितलाई 'विभेद' को डर
2020-08-30	Kantipur (Nepali)	संक्रमितलाई विभेद नगरौं'

Date of publication (yyyy/mm/dd)	Name of news media (select from drop-down menu)	Title of news/headline
2020-08-20	Kantipur (Nepali)	संक्रमितलाई वैकल्पिक उपचारको खोजी
2020-07-02	Kantipur (Nepali)	संक्रमितहरू विवाहदेखि वडा भेलासम्म
2020-04-28	Kantipur (Nepali)	संघको आस गर्दै प्रदेश
2020-05-15	Kantipur (Nepali)	संघले दियो स्वास्थ्य सामग्री
2020-06-06	Kantipur (Nepali)	संयन्त्रै संयन्त्रले सकस
2020-06-02	Online Khabar (Nepali)	संसदीय समितिको निर्देशन : क्वारेन्टिन मापदण्ड अनुसार सञ्चालन गर
2020-05-14	Online Khabar (Nepali)	संसदीय समितिको निर्देशन : स्वास्थ्य सामग्रीको यथाशीघ्र व्यवस्था गर
2020-06-18	Kantipur (Nepali)	संसद्मा कांग्रेसको ध्यानाकर्षण सूचना
2020-05-13	Kantipur (Nepali)	संसद्लाई नै झूटो विवरण पेस
2020-06-26	Kantipur (Nepali)	सकसमा संक्रमित
2020-05-31	Kantipur (Nepali)	सकिए परीक्षण किट
2020-07-09	Kantipur (Nepali)	सचियामाको ह्यान्ड सेनिटाइजर
2020-05-27	Kantipur (Nepali)	सञ्चार क्षेत्रलाई राहत जरुरी'
2020-03-22	Kantipur (Nepali)	'सञ्चारकर्मीलाई घरबाटै काम गर्ने व्यवस्था मिलाऊ'
2020-05-06	Online Khabar (Nepali)	सडकमार्गबाटै मलेसिया पुर्‍याइयो किट, भोलि काठमाडौं आइपुग्ने
2020-04-25	Online Khabar (Nepali)	सडकमै बनाइयो स्वचालित स्यानिटाइजिङ स्टेसन
2020-05-05	Online Khabar (Nepali)	सत्तारुढ सांसदको प्रश्न : कोरोनासँग लड्नुपर्ने बेला सरकारको ध्यान कता छ ?
2020-05-05	Kantipur (Nepali)	सत्यमोहनले देखेका दुई महामारी
2020-06-15	Kantipur (Nepali)	सपिङ मल सोमबारदेखि खुल्दै
2020-07-16	Kantipur (Nepali)	सपिङ मलमा भाडा विवाद चर्किंदै
2020-08-31	Online Khabar (Nepali)	सप्तरी र महोत्तरीमा एक साता लम्बियो निषेधाज्ञा
2020-08-03	Online Khabar (Nepali)	सप्तरीमा ६ जना प्रहरीमा कोरोना संक्रमण पुष्टि
2020-04-01	Online Khabar (Nepali)	सप्तरीमा पटनाबाट फर्किएका ८ जना क्वारेन्टाइनमा
2020-08-18	Online Khabar (Nepali)	सप्तरीमा भदौ १५ गतेसम्म निषेधाज्ञा थप
2020-08-03	Online Khabar (Nepali)	सप्तरीमा मंगलबारदेखि निषेधाज्ञा
2020-06-08	Kantipur (Nepali)	सफाइकर्मीलाई क्रिएसनको राहत
2020-08-10	Online Khabar (Nepali)	सबै अस्पतालमा कोरोना संक्रमितलाई राख्ने तयारी
2020-08-19	Kantipur (Nepali)	सबै अस्पतालमा कोरोनाको उपचार गर्नु उचित हो?
2020-05-16	Kantipur (Nepali)	सबै अस्पतालमा महामारीको उपचार
2020-03-03	Kantipur (Nepali)	सबै उपाय अवलम्बन गर्नू : सर्वोच्च
2020-08-30	Online Khabar (Nepali)	सबै कोरोना संक्रमितको जिम्मा लिन सरकारलाई निर्देशन
2020-03-23	Kantipur (Nepali)	सबै सीमा नाका एक साता बन्द
2020-06-05	Kantipur (Nepali)	समय तोकेर व्यापार
2020-05-14	Kantipur (Nepali)	समाजको बहुपत्रीय वर्ग र सरकार
2020-08-28	Kantipur (Nepali)	समाजलाई प्रश्न–'हामीलाई घृणा किन ?'
2020-07-30	Online Khabar (Nepali)	समुदायमा कोरोना देखिएपछि नेपालगन्जमा निषेधाज्ञा
2020-07-19	Kantipur (Nepali)	समुदायमा संक्रमण बुझ्न सर्वेक्षण कहिले ?
2020-08-27	Kantipur (Nepali)	समूहगत परीक्षण नगरी बित्यो निषेधाज्ञाको एक साता
2020-04-21	Online Khabar (Nepali)	समूहमा नमाज नपढ्न सरकारको अपिल
2020-06-12	Kantipur (Nepali)	सम्बोधन गर्नुपर्ने सरोकार
2020-03-22	Kantipur (Nepali)	सय शय्याको अस्पताल बन्दै
2020-04-27	Kantipur (Nepali)	सय शय्याको कोरोना अस्पताल
2020-06-05	Kantipur (Nepali)	सरकार ! पेटभरी खान मन छ

Date of publication (yyyy/mm/dd)	Name of news media (select from drop-down menu)	Title of news/headline
2020-05-14	Kantipur (Nepali)	सरकार असफल'
2020-06-21	Kantipur (Nepali)	सरकार आर्थिक संकटमा
2020-06-18	Kantipur (Nepali)	सरकार निर्दयी बन्दै
2020-03-20	Online Khabar (Nepali)	सरकार लक डाउनतर्फ जाँदै छ ? प्रधानमन्त्रीलाई भेटेपछि ईश्वर पोखरेलले बोलाए बैठक
2020-05-08	Kantipur (Nepali)	सरकार, सीमाको हारगुहार सुन
2020-04-23	Online Khabar (Nepali)	सरकारका प्रवक्ता भन्छन्-जोखिम हेरेर मात्र लकडाउनबारे निर्णय गर्छौं
2020-05-22	Online Khabar (Nepali)	सरकारको अलमल : पहिले काठमाडौंमा, अहिले सिमानामा
2020-06-08	Kantipur (Nepali)	सरकारको अस्पष्ट नीति
2020-08-24	Kantipur (Nepali)	सरकारको उपस्थिति शून्य
2020-03-21	Kantipur (Nepali)	सरकारको थप तयारी
2020-08-16	Online Khabar (Nepali)	सरकारको निर्णय विपरीत खुलेको विद्यालयका शिक्षकमा कोरोना
2020-04-25	Online Khabar (Nepali)	सरकारको निष्कर्ष : लकडाउनले कोरोना संक्रमण नियन्त्रणमा
2020-05-01	Online Khabar (Nepali)	सरकारलाई निर्देशन : लकडाउन खुकुलो बनाउनू, जनआस्था कायम गर्नू
2020-08-26	Online Khabar (Nepali)	सरकारले कोरोना नियन्त्रणको योजना सार्वजनिक गरोस् : कांग्रेस
2020-04-10	Kantipur (Nepali)	सरकारले नै फैलायो अफवाह
2020-07-05	Online Khabar (Nepali)	सरकारले भन्यो : ढलमा भाइरस देखिँदैमा समुदायमा कोरोना संक्रमण फैलियो भन्न मिल्दैन
2020-08-02	Online Khabar (Nepali)	सरकारले वीरगञ्जमा पठायो उच्चस्तरीय स्वास्थ्य टोली
2020-05-19	Kantipur (Nepali)	सरकारले सहयोग र समन्वय नगरे उद्योग व्यवसाय बन्द गर्ने चेतावनी
2020-06-12	Kantipur (Nepali)	सरकारविरुद्ध उत्रिए सडकमा युवा
2020-06-12	Kantipur (Nepali)	सरकारी कार्यालय दुई सिफ्टमा
2020-05-22	Online Khabar (Nepali)	सर्लाहीका कोरोना संक्रमित भारतबाट लुकेर नेपाल छिरेका थिए
2020-08-16	Online Khabar (Nepali)	सर्लाहीमा एक साता थपियो निषेधाज्ञा
2020-08-23	Online Khabar (Nepali)	सर्लाहीमा निषेधाज्ञाको म्याद एक साता थपियो
2020-08-09	Online Khabar (Nepali)	सर्लाहीमा सोमबारदेखि निषेधाज्ञा
2020-05-17	Online Khabar (Nepali)	सर्वोच्चको आदेश : तरकारी र फलफूल आयात गर्दा कोरोना परीक्षण गर्नू
2020-08-06	Kantipur (Nepali)	सवा ५ लाख सरकारी कर्मचारीको कोरोना बिमा
2020-05-12	Kantipur (Nepali)	सविताबाट सहयोग जारी
2020-03-22	Kantipur (Nepali)	सशस्त्रको अस्पताल कोरोनाका लागि मात्रै
2020-04-02	Online Khabar (Nepali)	सहकारी दिवसको अवसरमा कोरोना नियन्त्रण केन्द्र स्थापना
2020-06-29	Kantipur (Nepali)	सहकारीमा बचत न ब्याज
2020-08-25	Kantipur (Nepali)	सहयोग जुटाएर पीसीआर मेसिन
2020-06-05	Kantipur (Nepali)	सहरमा रोजगारी गुम्यो, गाउँमा बीउ–मल छैन
2020-05-05	Online Khabar (Nepali)	सांसदको आग्रह – विदेशमा अलपत्र नेपालीलाई स्वदेश फर्काऊ
2020-08-06	Kantipur (Nepali)	सांसदहरूको 'जबर्जस्ती' भ्रमण !
2020-05-13	Kantipur (Nepali)	सांसदहरूलाई राजधानी बाहिर जान रोक
2020-07-21	Online Khabar (Nepali)	साउन १५ बाट होटल खुल्ने, भोज र पार्टी गर्न नपाइने
2020-07-19	Kantipur (Nepali)	साउन २१ बाट आन्तरिक उडान !
2020-06-01	Online Khabar (Nepali)	साझा पार्टीको माग– सांसद विकास कोषको रकमले कोरोना किट किनौं
2020-06-10	Kantipur (Nepali)	साढे १४ हजार नमुना पालोमा
2020-03-23	Kantipur (Nepali)	साढे ५ लाख शय्याका क्वारेन्टाइन बनाइने

Date of publication (yyyy/mm/dd)	Name of news media (select from drop-down menu)	Title of news/headline
2020-07-07	Kantipur (Nepali)	साढे पाँच महिनापछि रसुवागढीबाट आयात सुरु
2020-06-02	Kantipur (Nepali)	सातामै भित्रिए १ लाख ३ हजार
2020-03-19	Online Khabar (Nepali)	सातै प्रदेशमा कोरोना परीक्षणको व्यवस्था मिलाउन सरकारलाई निर्देशन
2020-05-17	Kantipur (Nepali)	साना उद्यममा ६०५ रोजगारी गुम्यो'
2020-08-02	Kantipur (Nepali)	सानालाई ऐन, ठूलालाई चैन !
2020-05-03	Kantipur (Nepali)	सानालाई संरक्षण
2020-06-27	Kantipur (Nepali)	सामाजिक दूरी
2020-06-14	Kantipur (Nepali)	सामाजिक दूरी राखेर प्रदर्शन
2020-06-08	Kantipur (Nepali)	सामाजिक दूरीसहित व्यापार
2020-07-07	Kantipur (Nepali)	सार्वजनिक यातायात चलाउन छलफलमा जुटे व्यवसायी
2020-07-15	Kantipur (Nepali)	सार्वजनिक यातायातमा राज्यको बेवास्ता
2020-07-11	Kantipur (Nepali)	सार्वजनिक सवारी चले संक्रमण 'भयावह'
2020-07-31	Online Khabar (Nepali)	सार्वजनिक सवारीमा यात्रा : चिउँडोमा मास्क, सामाजिक दुरी कागजमै सीमित
2020-03-21	Kantipur (Nepali)	सावधानी अपनाउँदैनन् चालक
2020-06-05	Kantipur (Nepali)	सावधानी अपनाउन सिफारिस
2020-03-13	Kantipur (Nepali)	सिजनमै छैनन् पर्यटक
2020-05-02	Kantipur (Nepali)	सिनेमामा चौतर्फी सं‌कट
2020-05-17	Online Khabar (Nepali)	सिन्धुपाल्चोकका ४ वडा र स्वास्थ्य चौकी सिल, बाह्रबिसे बजार पनि बन्द
2020-05-13	Online Khabar (Nepali)	सिरहामा आरडीटी किट अभाव
2020-08-07	Online Khabar (Nepali)	सिराहाको लहानमा 'लकडाउन'
2020-08-16	Online Khabar (Nepali)	सिराहामा भदौ १५ गतेसम्म लकडाउन
2020-06-06	Kantipur (Nepali)	सीजीको प्रसूति केन्द्रलाई सहयोग
2020-06-11	Kantipur (Nepali)	सीडीओभन्दा प्रदेश सरकार तल !
2020-04-18	Online Khabar (Nepali)	सीमा क्षेत्रमा अलपत्र नेपालीको उद्धार गर्न १० दलको माग
2020-08-26	Kantipur (Nepali)	सीमा वारपार गराउन लेनदेन !
2020-05-14	Kantipur (Nepali)	सीमाका अलपत्र २९०० भित्रिए
2020-05-09	Kantipur (Nepali)	सीमाका क्वारेन्टाइनमा पीसीआर
2020-05-14	Kantipur (Nepali)	सीमाको गुहार नसुन्ने राष्ट्रवाद
2020-05-09	Kantipur (Nepali)	सीमापारि रोकिएकालाई भित्र्याउन प्रधानमन्त्री अनिच्छुक
2020-07-28	Kantipur (Nepali)	सीमामा निस्फिक्री आउजाउले जोखिम
2020-05-15	Kantipur (Nepali)	सीमामा बाँसको बार
2020-05-07	Kantipur (Nepali)	सीमामा रोकिएकालाई भित्र्याउन सुरक्षा निकायको सुझाव
2020-05-08	Kantipur (Nepali)	सीमामा सुरक्षाकर्मीमाथि फेरि आक्रमण
2020-03-21	Kantipur (Nepali)	सीमामा स्वयंसेवी युवा
2020-04-26	Kantipur (Nepali)	सीमामै अड्किए २,३१८ नेपाली
2020-07-11	Kantipur (Nepali)	सीसीएमसीमा नगर प्रतिनिधि !
2020-08-30	Kantipur (Nepali)	सुत्केरीलाई जबर्जस्ती आइसोलेसन
2020-04-05	Online Khabar (Nepali)	सुदूरपश्चिम सरकार भन्छ– विदेशबाट आएका सबैलाई परीक्षणको दायरामा ल्याउनुपर्छ
2020-04-11	Online Khabar (Nepali)	सुदूरपश्चिमका सबै जिल्लामा सुरु भयो र्‍यापिड टेस्ट
2020-04-28	Online Khabar (Nepali)	सुदूरपश्चिममा १५ हजार जनामा कोरोना परीक्षण
2020-04-11	Online Khabar (Nepali)	सुदूरपश्चिममा १९०० जनामा र्‍यापिड टेस्ट, सबैको रिपोर्ट नेगेटिभ
2020-04-07	Online Khabar (Nepali)	सुदूरपश्चिममा १९५ नमुना संकलन, ६६ जनाको काठमाडौं पठाइयो

Date of publication (yyyy/mm/dd)	Name of news media (select from drop-down menu)	Title of news/headline
2020-03-28	Online Khabar (Nepali)	सुदूरपश्चिममा कोरोना भाइरस रोकथामका लागि ४० करोडको कोष
2020-04-07	Online Khabar (Nepali)	सुदूरपश्चिममा न चिकित्सक, न भेन्टिलेटर
2020-04-04	Online Khabar (Nepali)	सुदूरपश्चिममा भेटिए ३ कोरोना संक्रमित
2020-06-10	Kantipur (Nepali)	सुदूरपश्चिममा स्वाब संकलन नै रोकियो
2020-06-15	Kantipur (Nepali)	सुदूरपश्चिमले बढायो मन्त्री–सांसदको तलब
2020-05-09	Kantipur (Nepali)	सुदूरमा सबै निको
2020-05-03	Kantipur (Nepali)	सुनसान रसुवागढी
2020-05-14	Kantipur (Nepali)	सुनौली जाने घटेपछि रुपैयाँ जोगियो
2020-05-21	Kantipur (Nepali)	सुरक्षाकर्मी उच्च जोखिममा
2020-08-18	Online Khabar (Nepali)	सुरक्षाकर्मीको तलब काट्न हतार, प्रोत्साहन भत्ता दिन आनाकानी
2020-03-22	Kantipur (Nepali)	सुरक्षात्मक तालिम लिँदै चिकित्सक
2020-05-17	Kantipur (Nepali)	सुर्खेतमा ५० योजनाले लिए निर्माण अनुमति
2020-06-17	Online Khabar (Nepali)	सुर्खेतमा आत्महत्या गरेकी युवतीमा कोरोना संक्रमण पुष्टि
2020-07-01	Online Khabar (Nepali)	सुर्खेतमा होटल र रेष्टुरेन्ट बन्द गर्न प्रशासनको निर्देशन
2020-03-27	Online Khabar (Nepali)	सुस्त कन्ट्याक ट्रेसिङ, जनस्वास्थ्यविद् भन्छन् – अमेरिकाले जस्तो खेलाँची नगरौं
2020-03-20	Kantipur (Nepali)	सेना ‘हाई अलर्ट’
2020-08-11	Kantipur (Nepali)	सेनालाई १० वटा भेन्टिलेटर
2020-05-06	Kantipur (Nepali)	सेनाले अझै ल्याइपुर्‍याएन स्वास्थ्य सामग्री
2020-06-18	Kantipur (Nepali)	सेनाले गरेको खरिदमा कुनै अनियमितता छैन’
2020-06-14	Kantipur (Nepali)	सेनाले भारतबाट औषधि ल्याउने प्रक्रिया अलपत्र
2020-07-03	Kantipur (Nepali)	सेनाले ल्याएका सामान मागेभन्दा फरक
2020-06-02	Kantipur (Nepali)	सेनाले ल्याएकामा पीसीआर किट थोरै
2020-05-17	Kantipur (Nepali)	सेनाले ल्यायो स्वास्थ्य सामग्री
2020-06-26	Kantipur (Nepali)	सेनिटाइजर र मास्क उत्पादनमा हडताल गर्न निषेध
2020-08-24	Kantipur (Nepali)	सेभ द चिल्ड्रेनले दियो ७ थान भेन्टिलेटर
2020-05-04	Kantipur (Nepali)	सेभ द चिल्ड्रेनले दियो पीपीई
2020-06-29	Kantipur (Nepali)	सेयर बजार सोमबारदेखि खुल्दै
2020-06-21	Kantipur (Nepali)	सेयर बजारबारे अझै टुंगो लागेन
2020-05-19	Kantipur (Nepali)	सेल पेबाट कोरोना बिमा
2020-03-23	Kantipur (Nepali)	सेल्फ–क्वारेन्टाइनमा कसरी बस्ने ?
2020-08-31	Kantipur (Nepali)	सैनिकद्वारा गोली हानी आत्महत्या
2020-07-30	Kantipur (Nepali)	सौराहाका होटल बिहीबारदेखि खुल्दै
2020-05-04	Kantipur (Nepali)	स्काउटले बढायो सक्रियता
2020-05-19	Kantipur (Nepali)	स्ट्यान्डर्ड चार्टर्डले दियो १ करोड १६ लाख
2020-05-16	Kantipur (Nepali)	स्थानीय तहमै रोजगारी
2020-06-14	Kantipur (Nepali)	स्थानीय सरकार मौन
2020-04-04	Online Khabar (Nepali)	स्थानीयमा कोरोना संक्रमण : सम्भावित महामारीको पूर्वसूचना !
2020-05-17	Kantipur (Nepali)	स्थानीयमा त्रास
2020-05-06	Kantipur (Nepali)	स्थिरता र अस्थिरताको भाष्य
2020-08-30	Kantipur (Nepali)	स्याउ उत्पादन आधा घट्यो
2020-05-27	Online Khabar (Nepali)	स्यानिटाइज गरियो नारायणी अस्पताल, नियमित सेवा सुरु गर्न अन्योल
2020-05-06	Kantipur (Nepali)	स्यानिटाइजर स्प्रेले स्वास्थ्यमा गम्भीर असर

Date of publication (yyyy/mm/dd)	Name of news media (select from drop-down menu)	Title of news/headline
2020-05-10	Kantipur (Nepali)	स्वदेशी उद्योगले बढाए सेनिटाइजर उत्पादन
2020-05-18	Kantipur (Nepali)	स्वस्थ माटोमै दिगो रहन्छ कृषि
2020-06-21	Kantipur (Nepali)	स्वाब व्यवस्थापनको त्रुटिले गलत नतिजा'
2020-04-28	Kantipur (Nepali)	स्वाब संकलन नै त्रुटिपूर्ण ः विज्ञ
2020-05-10	Kantipur (Nepali)	स्वाब संकलन र ट्रेसिङलाई गति
2020-08-13	Online Khabar (Nepali)	स्वाब संकलनमा सहयोग गर्न बस्नेत परिवारलाई मेयरको आग्रह
2020-06-08	Kantipur (Nepali)	स्वार्थमा चुकेको विश्वनेतृत्व
2020-05-06	Kantipur (Nepali)	स्वास्थ्य जाँचबिनै उपत्यका भित्रनेको लर्को
2020-05-18	Kantipur (Nepali)	स्वास्थ्य पूर्वाधारको चुनौती
2020-06-05	Online Khabar (Nepali)	स्वास्थ्य मन्त्रालयको टेस्टिङ गाइडलाइन : क्वारेन्टिन बसेका सबैको पीसीआर परीक्षण नगरिने
2020-05-13	Online Khabar (Nepali)	स्वास्थ्य मन्त्रालयमा पत्रकारलाई प्रवेश निषेध !
2020-08-06	Online Khabar (Nepali)	स्वास्थ्य मन्त्रालयले भन्यो : कार्यालयहरुमा गोष्ठी र बैठक नगर्नू
2020-07-01	Online Khabar (Nepali)	स्वास्थ्य मन्त्रालयले भन्यो : बालबालिकामा संक्रमण बढ्यो, अभिभावक सचेत हुनुस्
2020-08-13	Online Khabar (Nepali)	स्वास्थ्य मन्त्रालयले भन्यो : स्वाब दिएपछि अनिवार्य आइसोलेसनमा बसौं
2020-06-03	Online Khabar (Nepali)	स्वास्थ्य संकटकाल घोषणा गर्न स्वास्थ्य मन्त्रालयको सिफारिस
2020-05-17	Kantipur (Nepali)	स्वास्थ्य संकटमा बढ्दो सामाजिक असमानता
2020-06-22	Kantipur (Nepali)	स्वास्थ्य सामग्री खरिदमा निर्देशन दिनेलाई उन्मुक्ति?
2020-06-20	Kantipur (Nepali)	स्वास्थ्य सामग्री खरिदमा मूल्य अन्तर उच्च
2020-04-27	Kantipur (Nepali)	स्वास्थ्य सामग्री लिन विमान चीनतिर
2020-04-24	Kantipur (Nepali)	स्वास्थ्य सामग्री ल्याउन बोलपत्र
2020-07-23	Kantipur (Nepali)	स्वास्थ्य सुरक्षाबिनै यात्रा
2020-05-21	Kantipur (Nepali)	स्वास्थ्यकर्मी उच्च जोखिममा
2020-03-08	Kantipur (Nepali)	स्वास्थ्यकर्मी नै जोखिममा
2020-08-24	Online Khabar (Nepali)	स्वास्थ्यकर्मी र सुरक्षाकर्मीलाई सम्मानजनक व्यवहार गरौं : मानव अधिकार आयोग
2020-06-26	Kantipur (Nepali)	स्वास्थ्यकर्मी संक्रमित भएपछि सेवा बन्द
2020-08-24	Kantipur (Nepali)	स्वास्थ्यकर्मीमाथिको दुर्व्यवहार अस्वीकार्य
2020-08-23	Kantipur (Nepali)	स्वास्थ्यकर्मीलाई प्रोत्साहन खोइ?
2020-08-24	Kantipur (Nepali)	स्वास्थ्यकर्मीलाई सद्भाव चाहिन्छ
2020-06-05	Kantipur (Nepali)	स्वास्थ्यका ६ उच्च अधिकारीको सरुवा
2020-05-28	Online Khabar (Nepali)	स्वास्थ्यको पूर्वाधार विकासमा उल्लेख्य बजेट, कोरोना उपचारका लागि ६ अर्ब
2020-06-16	Kantipur (Nepali)	स्वास्थ्यमा प्राथमिकता, सांसदलाई निरन्तरता
2020-06-01	Kantipur (Nepali)	स्वास्थ्यमा सुधारको खाँचो
2020-05-30	Kantipur (Nepali)	स्वास्थ्यलाई विशाल ग्रुपको सहयोग
2020-08-24	Kantipur (Nepali)	स्विपर नै जाँच्छन् संक्रमितको ज्वरो
2020-07-24	Online Khabar (Nepali)	स्विस सरकारले दियो २० हजार पीसीआर किट
2020-07-30	Online Khabar (Nepali)	स्वीकृति पाएका ल्याबले आफैं स्वाब संकलन गरेर कोरोना परीक्षण गर्न पाउने
2020-05-07	Online Khabar (Nepali)	स्वीस सरकारले दिएको ३० हजार पीसीआर किट आज आइपुग्ने
2020-07-02	Kantipur (Nepali)	हचुवामा उद्धार र घरफिर्ती
2020-08-05	Kantipur (Nepali)	'हटस्पट' मा आइसोलेसन अभाव

Date of publication (yyyy/mm/dd)	Name of news media (select from drop-down menu)	Title of news/headline
2020-08-14	Kantipur (Nepali)	हटस्पट’ वीरगन्जमा अभावै अभाव
2020-06-28	Kantipur (Nepali)	हटस्पटका आइसोलेसनमा बिजोग
2020-06-14	Kantipur (Nepali)	हटस्पटमै छैन कन्ट्याक्ट ट्रेसिङ
2020-05-01	Online Khabar (Nepali)	हरेक परिवारको कम्तीमा एक जनामा कोरोना जाँच गर्न निर्देशन
2020-06-03	Online Khabar (Nepali)	हल्का लक्षण भएका कोरोना संक्रमित पनि घरमै आइसोलेसनमा बस्न पाउने
2020-06-13	Kantipur (Nepali)	हाँस, रोएर केही हुँदैन
2020-07-04	Kantipur (Nepali)	हाकिमको अफर
2020-02-22	Kantipur (Nepali)	हात धुँदा संक्रमणबाट मुक्ति
2020-03-08	Kantipur (Nepali)	हात धुन निःशुल्क पानी
2020-03-21	Kantipur (Nepali)	हात धुन सिकाउँदै स्काउटर
2020-03-19	Kantipur (Nepali)	हात धुने बानी बसालौं
2020-06-29	Kantipur (Nepali)	हात धुने बिमार !
2020-03-17	Kantipur (Nepali)	हात धोएर अस्पतालमा
2020-03-21	Kantipur (Nepali)	हात नधोए सामान किन्न पाइँदैन
2020-06-09	Kantipur (Nepali)	हातमा मसाल थमाएजस्तै
2020-05-04	Online Khabar (Nepali)	हातले नछोइकन निस्किन्छ साबुन, सेनिटाजर र पानी
2020-05-04	Kantipur (Nepali)	हामीलाई कोरोनाले झुक्यायो'
2020-06-09	Kantipur (Nepali)	हामीले छोएको पैसा चल्दैन'
2020-05-04	Kantipur (Nepali)	हाम्रो इन्टरनेट कस्तो छ?
2020-02-27	Kantipur (Nepali)	हुम्लामा होटल बुकिङ शून्य
2020-06-19	Kantipur (Nepali)	हेटौंडाका २०५ उद्योग खुलेनन्
2020-08-24	Online Khabar (Nepali)	हेटौंडामा भदौ १५ गतेसम्म निषेधाज्ञा
2020-07-15	Kantipur (Nepali)	होटल खुलाउन माग
2020-08-26	Online Khabar (Nepali)	होम आइसोलेसनको बेडबाट हेर्दा...
2020-08-21	Online Khabar (Nepali)	होम आइसोलेसनमा थिए, एम्बुलेन्स बोलाउँदा बोलाउँदै बिते
2020-08-20	Online Khabar (Nepali)	होम आइसोलेसनमा बस्ने संक्रमितलाई टोखा नगरपालिकले दैनिक ३५० रुपैयाँ दिने
2020-08-16	Kantipur (Nepali)	होम आइसोलेसनमा बस्नेले नियम मान्दैनन्

Bangladesh Media articles

Date of the news	News URL	Title of the news
09/03/2020	https://www.tbsnews.net/bangladesh/health/prices-7-hand-sanitizer-brands-fixed-54133	Prices of seven hand sanitizer brands fixed
09/03/2020	https://www.tbsnews.net/bangladesh/announcement-made-close-border-haat-check-spread-coronavirus-54082	Announcement made to close border haat to check spread of Coronavirus
09/03/2020	https://www.tbsnews.net/bangladesh/2-mymensingh-pharmacies-fined-selling-masks-higher-price-53986	2 Mymensingh pharmacies fined for selling masks at higher price
09/03/2020	https://www.tbsnews.net/bangladesh/health/why-such-overreaction-53944	Why such overreaction over coronavirus?
09/03/2020	https://www.tbsnews.net/international/coronavirus-chronicle/coronavirus-prompts-panic-shopping-dhaka-53866	Coronavirus prompts panic shopping in Dhaka
09/03/2020	https://www.tbsnews.net/bangladesh/family-relatives-corona-patients-quarantined-health-secretary-53827	Closing educational institutions for corona not reasonable'
09/03/2020	https://www.tbsnews.net/bangladesh/health/be-alert-dont-panic-experts-53776	Be alert, don’t panic: Experts
09/03/2020	https://www.tbsnews.net/economy/stock/coronavirus-panic-dhaka-stocks-tumble-205-points-53752	Investors lose Tk17,000 crore in day of corona nervousness
09/03/2020	https://www.tbsnews.net/international/coronavirus-chronicle/uber-delivery-services-compensate-drivers-who-catch-virus-53773	Uber, delivery services to compensate drivers who catch virus
09/03/2020	https://www.tbsnews.net/bangladesh/education/universities-plan-move-classes-online-if-coronavirus-breaks-out-53641	Universities plan to move classes online if coronavirus breaks out
10/03/2020	https://www.tbsnews.net/bangladesh/health/private-hospitals-unable-unwilling-treat-coronavirus-patients-54169	Private hospitals unable, unwilling to treat coronavirus patients
10/03/2020	https://www.tbsnews.net/markets/store-shelves-empty-coronavirus-fears-lead-hoarding-54259	Store shelves empty as coronavirus fears lead to hoarding
10/03/2020	https://www.tbsnews.net/companies/coronavirus-hotline-numbers-announced-toll-free-banglalink-subscribers-54265	Banglalink announces toll-free calls to coronavirus hotlines
10/03/2020	https://www.tbsnews.net/bangladesh/crime/rab-raids-pharmacies-overcharging-complaints-54331	16 pharmacies fined for selling masks, sanitisers at hiked price
10/03/2020	https://www.tbsnews.net/bangladesh/health/woman-flees-sylhet-hospital-after-suspicion-corona-infection-54499	Woman flees Sylhet hospital after suspicion of corona infection
11/03/2020	https://www.tbsnews.net/bangladesh/health/few-people-markets-schools-amid-coronavirus-fear-55012	Fewer people at markets, schools amid virus fear
11/03/2020	https://www.tbsnews.net/companies/pharma/pharma-industry-braces-raw-material-crisis-54640	Pharma industry braces for raw material crisis

Date of the news	News URL	Title of the news
11/03/2020	https://www.tbsnews.net/economy/trade/dwindling-raw-material-stocks-worry-businesses-54589	**Dwindling raw material stocks worry businesses**
12/03/2020	https://www.tbsnews.net/companies/summit-donates-5-thermal-scanners-health-ministry-55522	**Summit donates 5 thermal scanners to health ministry**
13/03/2020	https://www.tbsnews.net/economy/rmg/apparel-exporters-face-cancellation-orders-55579	**RMG makers stare at order cancellations**
13/03/2020	https://www.tbsnews.net/economy/industry/coronavirus-dampens-luxury-hotel-business-chattogram-55684	**Coronavirus dampens luxury hotel business in Chattogram**
13/03/2020	https://www.tbsnews.net/markets/fear-corona-grips-shoppers-56002	**Fear of corona grips shoppers**
13/03/2020	https://www.tbsnews.net/international/coronavirus-chronicle/unilever-asks-employees-work-home-amid-coronavirus-concerns	**Unilever asks employees to work from home amid coronavirus concerns**
14/03/2020	https://www.tbsnews.net/bangladesh/summit-group-presents-thermal-scanners-health-ministry-56071	**Summit group presents thermal scanners to the Health Ministry**
14/03/2020	https://www.tbsnews.net/economy/trade/import-falls-17-month-chattogram-port-56227	**Import falls 17% in a month at Chattogram port**
15/03/2020	https://www.tbsnews.net/bangladesh/health/coronavirus-demand-closure-educational-institutions-gets-louder-56848	**Coronavirus: Demand for closure of educational institutions gets louder**
15/03/2020	https://www.tbsnews.net/bangladesh/crime/daraz-fined-selling-mask-high-price-56845	**Daraz fined for selling mask at high price**
15/03/2020	https://www.tbsnews.net/bangladesh/some-countries-want-cancel-apparel-orders-tipu-munshi-56809	**Some countries want to cancel apparel orders: Tipu Munshi**
15/03/2020	https://www.tbsnews.net/bangladesh/health/forgotten-ones-our-fight-against-coronavirus-56656	**The forgotten ones in our fight against coronavirus**
15/03/2020	https://www.tbsnews.net/economy/trade/raw-material-supply-eases-export-markets-worsen-due-coronavirus-56440	**Raw material supply eases, but export markets worsen due to coronavirus**
16/03/2020	https://www.tbsnews.net/bangladesh/health/few-test-facility-inability-quarantine-patients-expose-holes-56857	**Few test facility, inability to quarantine returnees expose holes**
16/03/2020	https://www.tbsnews.net/economy/aviation/aviation-nosedives-corona-fallout-56905	**Aviation nosedives on corona fallout**
16/03/2020	https://www.tbsnews.net/bangladesh/education/govt-orders-closure-all-educational-institutions-march-17-56947	**Educational institutions to remain closed till March 31**
16/03/2020	https://www.tbsnews.net/economy/foreign-aid/coronavirus-wb-provide-100m-loan-support-bangladesh-57061	**Coronavirus: WB to provide $100m loan to support Bangladesh**
16/03/2020	https://www.tbsnews.net/economy/posh-hotel-employees-fear-job-losses-57085	**Posh hotel employees fear job losses**
16/03/2020	https://www.tbsnews.net/bangladesh/telecom/gp-encourages-part-its-team-work-home-57166	**GP encourages a part of its team to work from home**

Date of the news	News URL	Title of the news
16/03/2020	https://www.tbsnews.net/companies/telecom/banglalink-implement-work-home-policy-curb-coronavirus-spread-57202	**Banglalink to implement work from home policy to curb coronavirus spread**
16/03/2020	https://www.tbsnews.net/bangladesh/coronavirus-guardians-happy-shutdown-educational-institutions-57214	**Coronavirus: Guardians happy with shutdown of educational institutions**
16/03/2020	https://www.tbsnews.net/bangladesh/no-decision-yet-over-closure-mills-factories-secy-57220	**No decision yet over closure of mills, factories: Secy**
16/03/2020	https://www.tbsnews.net/bangladesh/health/coronavirus-govt-issues-directives-home-quarantine-57226	**Coronavirus: Govt issues directives for home quarantine**
16/03/2020	https://www.tbsnews.net/glitz/cinema-halls-remain-closed-until-april-2-57256	**Cinema halls to remain closed until April 2**
16/03/2020	https://www.tbsnews.net/bangladesh/health/bankers-start-using-gloves-touch-money-57280	**Bankers start using gloves to touch money**
17/03/2020	https://www.tbsnews.net/bangladesh/health/dncc-launches-hand-wash-programme-25-spots-city-57670	**DNCC launches hand wash programme at 25 spots in city**
17/03/2020	https://www.tbsnews.net/bangladesh/health/returnee-expats-asked-not-go-mosques-islamic-foundation-57664	**Returnee expats asked not to go to mosques: Islamic Foundation**
17/03/2020	https://www.tbsnews.net/bangladesh/health/coronavirus-bidyanondo-sprays-disinfectant-vehicles-distributes-hand-sanitisers	**Coronavirus: Bidyanondo sprays disinfectant in vehicles, distributes hand sanitisers**
17/03/2020	https://www.tbsnews.net/bangladesh/telecom/gp-robi-banglalink-encourage-staff-work-home-over-coronavirus-57310	**GP, Robi, Banglalink encourage staff to work from home over coronavirus**
18/03/2020	https://www.tbsnews.net/bangladesh/dhakas-elite-clubs-plan-closures-amid-coronavirus-58108	**Dhaka’s elite clubs plan closures amid coronavirus**
18/03/2020	https://www.tbsnews.net/bangladesh/health-ministry-seeks-tk200cr-coronavirus-fund-58132	**Tongi Bishwa Ijtema grounds to be turned into quarantine centre**
18/03/2020	https://www.tbsnews.net/coronavirus-chronicle/gonoshasthaya-develops-simple-cheap-effective-coronavirus-test-system-58171	**Gonoshasthaya develops ‘simple, cheap, effective’ Coronavirus test system**
18/03/2020	https://www.tbsnews.net/bangladesh/campaigning-chattogram-city-corporation-polls-continues-amid-corona-scare-58195	**Campaigning for Chattogram City Corporation polls continues amid corona scare**
18/03/2020	https://www.tbsnews.net/bangladesh/corona-unit-be-set-each-division-maleque-58165	**‘Corona unit’ to be set up at each division: Maleque**
18/03/2020	https://www.tbsnews.net/bangladesh/coronavirus-25000-people-attend-laxmipur-prayer-58159	**Coronavirus: 25,000 people attend Laxmipur prayer**
18/03/2020	https://www.tbsnews.net/bangladesh/govt-urges-people-not-hoard-enough-food-country-58204	**Minister's assurance ignored, panic buying spree continues**
19/03/2020	https://www.tbsnews.net/coronavirus-chronicle/slum-residents-have-little-protection-against-covid-19-58432	**Slum residents have little protection against Covid-19**

Date of the news	News URL	Title of the news
19/03/2020	https://www.tbsnews.net/coronavirus-chronicle/frontline-fighters-lack-enough-personal-safety-gear-58288	**Frontline fighters lack enough personal safety gear**
19/03/2020	https://www.tbsnews.net/bangladesh/city-hospitals-empty-corridors-and-worried-faces-58630	**At city hospitals, empty corridors and worried faces**
19/03/2020	https://www.tbsnews.net/bangladesh/crime/2-detained-selling-used-masks-gloves-58300	**2 detained for selling used masks, gloves**
19/03/2020	https://www.tbsnews.net/tech/smartphones/huawei-offers-free-mobile-disinfection-service-bangladesh-58354	**Huawei offers free mobile disinfection service in Bangladesh**
19/03/2020	https://www.tbsnews.net/bangladesh/health/gonoshasthaya-kendra-now-can-import-materials-corona-testing-kit-58405	**Gonoshasthaya Kendra now can import raw materials for corona testing kit**
20/03/2020	https://www.tbsnews.net/bangladesh/bida-distribute-free-face-masks-across-country-58927	**Bida to distribute free face masks across the country**
20/03/2020	https://www.tbsnews.net/markets/panic-buying-raises-commodity-prices-more-58780	**Rice, onion retailers cash in on panic buying**
20/03/2020	https://www.tbsnews.net/bangladesh/crime/rab-detains-one-spreading-rumors-about-coronavirus-facebook-58882	**RAB detains one for spreading rumors about coronavirus on Facebook**
20/03/2020	https://www.tbsnews.net/bangladesh/health/drug-stores-see-buyers-rush-dhaka-58897	**Drug stores see buyers' rush in Dhaka**
20/03/2020	https://www.tbsnews.net/bangladesh/lakshmipur-town-shops-closed-till-march-31-58912	**Lakshmipur town shops closed till March 31**
20/03/2020	https://www.tbsnews.net/economy/shopping-malls-empty-staff-fear-losing-jobs-58921	**Shopping malls empty, staff fear losing jobs**
21/03/2020	https://www.tbsnews.net/coronavirus-chronicle/lower-income-people-suffer-double-blow-59296	**Lower income people suffer double blow**
21/03/2020	https://www.tbsnews.net/economy/rmg/eight-lakh-garment-workers-chattogram-face-corona-risk-59314	**Eight lakh garment workers in Chattogram face corona risk**
21/03/2020	https://www.tbsnews.net/coronavirus-chronicle/sajida-foundation-provide-10000-personal-protective-equipments-59272	**Sajida Foundation to provide 10,000 personal protective equipments**
21/03/2020	https://www.tbsnews.net/coronavirus-chronicle/coronavirus-dru-launches-ambulance-service-members-59266	**Coronavirus: DRU launches ambulance service for members**
21/03/2020	https://www.tbsnews.net/bangladesh/education/coronavirus-british-council-postpones-regular-courses-classes-59239	**Coronavirus: British Council postpones regular courses, classes**
21/03/2020	https://www.tbsnews.net/bangladesh/4-doctors-15-staff-delta-medical-quarantined-59221	**4 doctors, 15 staff of Delta Medical quarantined**
21/03/2020	https://www.tbsnews.net/bangladesh/who-suggests-lockdown-curb-spread-coronavirus-59215	**WHO suggests lockdown to curb spread of coronavirus**

Date of the news	News URL	Title of the news
21/03/2020	https://www.tbsnews.net/coronavirus-chronicle/adb-support-bangladesh-fight-against-coronavirus-59209	**ADB to support Bangladesh in fight against coronavirus**
21/03/2020	https://www.tbsnews.net/coronavirus-chronicle/jack-ma-donate-corona-testing-kits-protective-gears-bangladesh-59104	**Jack Ma to donate corona testing kits, protective gears to Bangladesh**
21/03/2020	https://www.tbsnews.net/bangladesh/infrastructure/world-bank-approves-170-million-better-sanitation-dhaka-59029	**World Bank approves $170 million for better sanitation in Dhaka**
21/03/2020	https://www.tbsnews.net/companies/sheba-xyz-employees-start-home-office-59011	**Sheba XYZ employees start home office**
21/03/2020	https://www.tbsnews.net/glitz/star-cineplex-shuts-down-due-covid-19-outbreak-58996	**Star Cineplex shuts down due to Covid-19 outbreak**
21/03/2020	https://www.tbsnews.net/economy/factories-not-be-closed-labour-ministry-59185	**Factories will not be closed: State minister**
22/03/2020	https://www.tbsnews.net/coronavirus-chronicle/coughing-hospitals-dont-want-you-59350	**Coughing? Hospitals don’t want you**
22/03/2020	https://www.tbsnews.net/bangladesh/health/brac-university-pharmacy-faculty-starts-making-hand-rub-59401	**BRAC University Pharmacy Faculty starts making hand rub**
22/03/2020	https://www.tbsnews.net/bangladesh/doctor-arrested-spreading-coronavirus-rumours-59410	**Doctor arrested for spreading rumours over coronavirus**
22/03/2020	https://www.tbsnews.net/bangladesh/court/high-court-orders-supply-protective-gears-doctors-nurses-59482	**Supply personal protective material to doctors, nurses: High Court**
22/03/2020	https://www.tbsnews.net/bangladesh/carew-co-producing-hand-sanitiser-59557	**Carew & Co producing hand sanitiser**
22/03/2020	https://www.tbsnews.net/bangladesh/industries-ministry-takes-safety-measures-check-coronavirus-spread-59614	**Industries ministry takes safety measures to check coronavirus spread**
22/03/2020	https://www.tbsnews.net/economy/rmg/bangladeshi-rmg-factories-start-making-masks-donating-rubana-huq-59536	**Bangladeshi RMG factories start making masks, donating: Rubana Huq**
22/03/2020	https://www.tbsnews.net/bangladesh/coronavirus-outbreak-could-become-worse-april-dscc-mayor-59563	**Coronavirus outbreak could become worse by April : Dhaka South Mayor**
22/03/2020	https://www.tbsnews.net/bangladesh/telecom/grameenphone-tells-everyone-stay-safe-home-59674	**Grameenphone tells everyone to stay safe at home**
22/03/2020	https://www.tbsnews.net/markets/shop-owners-association-decides-close-shopping-malls-59653	**Shopping malls to be shut, not superstores**
22/03/2020	https://www.tbsnews.net/bangladesh/health/synchronised-and-effective-approach-must-fight-coronavirus-59659	**‘Synchronised and effective approach a must to fight coronavirus’**
22/03/2020	https://www.tbsnews.net/bangladesh/legal-notice-airing-special-programmes-covid-19-59662	**Legal notice for airing special programmes on Covid-19**

Date of the news	News URL	Title of the news
22/03/2020	https://www.tbsnews.net/coronavirus-chronicle/all-taxes-waived-import-corona-safety-kits-and-service-materials-59668	**All taxes waived on import of corona safety kits and service materials**
22/03/2020	https://www.tbsnews.net/economy/bcsir-provide-free-hand-sanitizers-people-59686	**BCSIR to provide free hand sanitizers for people**
22/03/2020	https://www.tbsnews.net/bangladesh/health/1000-fmc-dockyard-staff-members-living-working-site-chattogram-59713	**1,000 FMC Dockyard staff members living, working on-site in Chattogram**
22/03/2020	https://www.tbsnews.net/bangladesh/health/two-house-owners-waive-rent-tenants-amid-coronavirus-crisis-59701	**Two house owners waive rent for tenants amid coronavirus crisis**
22/03/2020	https://www.tbsnews.net/bangladesh/21-shyambazar-warehouses-fined-tk4050-lakh-59728	**21 Shyambazar warehouses fined Tk40.50 lakh**
22/03/2020	https://www.tbsnews.net/coronavirus-chronicle/delta-hospital-doctor-tests-positive-coronavirus-59740	**Delta hospital doctor who treated dead corona patient also got infected**
23/03/2020	https://www.tbsnews.net/coronavirus-chronicle/coronavirus-bangladesh/ms-bangladesh-steps-produce-ppe-60043	**M&S Bangladesh steps up to produce PPE**
23/03/2020	https://www.tbsnews.net/economy/banking/bb-allows-advance-payment-500000-import-corona-treatment-materials-59953	**BB allows advance payment of $500,000 to import corona treatment materials**
23/03/2020	https://www.tbsnews.net/bangladesh/we-have-enough-stock-ppe-bma-59875	**We have enough stock of PPE: BMA**
23/03/2020	https://www.tbsnews.net/bangladesh/gulshan-society-launches-disinfection-programme-amid-coronavirus-59866	**Gulshan Society launches disinfection programme amid coronavirus**
23/03/2020	https://www.tbsnews.net/markets/supermarkets-will-remain-open-59749	**Supermarkets that will remain open**
23/03/2020	https://www.tbsnews.net/economy/rmg-factories-operating-precautionary-measures-60022	**RMG factories operating with precautionary measures**
23/03/2020	https://www.tbsnews.net/coronavirus-chronicle/coronavirus-bangladesh/bidyanondo-providing-food-home-quarantined-capital	**Bidyanondo providing food for home-quarantined in capital**
23/03/2020	https://www.tbsnews.net/coronavirus-chronicle/coronavirus-bangladesh/prime-bank-launches-awareness-campaign-fight	**Prime Bank launches awareness campaign to fight Coronavirus**
23/03/2020	https://www.tbsnews.net/coronavirus-chronicle/coronavirus-bangladesh/locals-protest-against-admitting-corona-patient-uttara	**Locals protest against admitting corona patient in Uttara hospitals**
23/03/2020	https://www.tbsnews.net/coronavirus-chronicle/coronavirus-bangladesh/chattogram-hospital-closes-icu-hdu-units-after-death	**Chattogram hospital closes ICU, HDU units after death of pneumonia patient**
23/03/2020	https://www.tbsnews.net/bangladesh/health/sufficient-azithromycin-stock-fight-corona-drug-administration-dg-59821	**Sufficient Azithromycin in stock to fight corona: Drug Administration DG**

Date of the news	News URL	Title of the news
23/03/2020	https://www.tbsnews.net/sports/players-ask-everyone-stay-aware-amid-coronavirus-outbreak-59986	**Players ask everyone to stay aware amid coronavirus outbreak**
23/03/2020	https://www.tbsnews.net/bangladesh/health/ppe-not-need-much-now-health-minister-60115	**PPE not needed that much now: Health minister**
23/03/2020	https://www.tbsnews.net/economy/rmg/apparel-makers-assure-timely-pay-workers-60199	**Apparel makers assure timely pay to workers**
24/03/2020	https://www.tbsnews.net/coronavirus-chronicle/coronavirus-bangladesh/brac-producing-masks-plans-ppe-production-battle	**BRAC producing masks, plans PPE production to battle coronavirus**
	https://www.tbsnews.net/bangladesh/daraz-takes-steps-ensure-customers-basic-needs-amid-corona-60262	**Daraz takes steps to ensure customers basic needs amid corona**
24/03/2020	https://www.tbsnews.net/coronavirus-chronicle/coronavirus-bangladesh/jica-give-five-pcr-machines-60292	**Jica to give five PCR machines**
24/03/2020	https://www.tbsnews.net/bangladesh/brac-unilever-jointly-work-against-coronavirus-60397	**BRAC, Unilever jointly work against coronavirus**
24/03/2020	https://www.tbsnews.net/glitz/meena-teaches-children-how-fight-deadly-virus-60364	**Meena' teaches children how to fight the deadly virus**
24/03/2020	https://www.tbsnews.net/coronavirus-chronicle/coronavirus-bangladesh/residents-fear-short-supply-shops-shutting-down-60337	**Residents in fear of short supply as shops shutting down**
24/03/2020	https://www.tbsnews.net/coronavirus-chronicle/coronavirus-bangladesh/pathao-responses-coronavirus-pandemic-60508	**Pathao responses to coronavirus pandemic**
24/03/2020	https://www.tbsnews.net/coronavirus-chronicle/coronavirus-bangladesh/mjf-donates-one-day-salary-gonoshasthaya-make-corona	**MJF donates one-day salary to Gonoshasthaya to make corona testing kit**
24/03/2020	https://www.tbsnews.net/coronavirus-chronicle/coronavirus-bangladesh/local-representatives-working-curb-coronavirus-tazul	**Local representatives working to curb coronavirus: Tazul**
24/03/2020	https://www.tbsnews.net/coronavirus-chronicle/coronavirus-bangladesh/anwer-khan-modern-hospitals-icu-team-quarantined-60505	**Anwer Khan Modern Hospital's ICU team quarantined**
24/03/2020	https://www.tbsnews.net/coronavirus-chronicle/coronavirus-bangladesh/shibchar-people-want-protect-themselves-60622	**In Shibchar, people want to protect themselves**
24/03/2020	https://www.tbsnews.net/coronavirus-chronicle/coronavirus-bangladesh/chattogram-doctors-rejecting-flu-patients-60469	**Chattogram doctors rejecting flu patients due to lack of PPE**
24/03/2020	https://www.tbsnews.net/economy/banking/hsbc-bangladesh-promises-support-its-customers-during-challenging-time-60553	**HSBC Bangladesh promises to support its customers during this challenging time**
25/03/2020	https://www.tbsnews.net/bangladesh/health/standard-bank-distributes-free-hand-sanitizer-among-people-61042	**Standard Bank distributes free hand sanitizer among people**

Date of the news	News URL	Title of the news
25/03/2020	https://www.tbsnews.net/companies/rangs-motors-starts-disinfection-program-terminals-61060	**Rangs Motors starts disinfection program at terminals**
25/03/2020	https://www.tbsnews.net/companies/telecom/huawei-joins-hands-bangladesh-combat-covid-19-60997	**Huawei joins hands with Bangladesh to combat Covid-19**
25/03/2020	https://www.tbsnews.net/coronavirus-chronicle/coronavirus-bangladesh/no-hospital-can-refuse-treat-any-patient-health	**No hospital can refuse to treat any patient: Health Ministry**
25/03/2020	https://www.tbsnews.net/coronavirus-chronicle/coronavirus-bangladesh/rehab-gives-tk25-lakh-govt-collect-coronavirus-test-kit	**REHAB donates Tk25 lakh to govt for buying coronavirus kits**
25/03/2020	https://www.tbsnews.net/international/sex-workers-one-worlds-largest-brothels-appeal-funds-due-coronavirus-60967	**Sex workers in one of world's largest brothels appeal for funds due coronavirus**
25/03/2020	https://www.tbsnews.net/tech/corona-crisis-tech-start-apploye-lends-hand-local-firms-61003	**Corona crisis: Tech start-up Apploye lends hand to local firms**
25/03/2020	https://www.tbsnews.net/economy/industry/apparel-factories-no-orders-can-shut-61078	**Apparel factories with no orders can shut**
25/03/2020	https://www.tbsnews.net/coronavirus-chronicle/coronavirus-bangladesh/chattogram-yet-get-coronavirus-testing-kits-61036	**Chattogram yet to get coronavirus testing kits**
25/03/2020	https://www.tbsnews.net/coronavirus-chronicle/coronavirus-bangladesh/india-provides-masks-head-cover-bangladesh-60868	**India provides masks, head-cover to Bangladesh**
25/03/2020	https://www.tbsnews.net/coronavirus-chronicle/coronavirus-bangladesh/evaly-deliver-daily-commodities-customers-doorstep	**Evaly offers home delivery for daily commodities**
25/03/2020	https://www.tbsnews.net/companies/bkash-customers-allowed-add-money-visa-card-60742	**bKash introduces add money service from Visa to contain corona spread**
26/03/2020	https://www.tbsnews.net/coronavirus-chronicle/coronavirus-bangladesh/when-flowers-seem-grenades-love-still-flowers-61444	**When flowers seem like grenades and love still flowers**
26/03/2020	https://www.tbsnews.net/economy/rmg/bgmea-pleas-close-garment-factories-amid-virus-scare-61570	**BGMEA pleas to close garment factories amid virus scare**
26/03/2020	https://www.tbsnews.net/bangladesh/health/digital-healthcare-launches-free-online-coronavirus-symptom-checkup-61522	**Digital Healthcare launches free online coronavirus symptom checkup**
26/03/2020	https://www.tbsnews.net/bangladesh/30-tv-stations-under-govt-scanner-check-coronavirus-rumours-61519	**30 TV stations under govt scanner to check coronavirus rumours**
26/03/2020	https://www.tbsnews.net/coronavirus-chronicle/coronavirus-bangladesh/8-more-labs-carry-out-coronavirus-tests-61462	**8 labs not yet ready for coronavirus test**
26/03/2020	https://www.tbsnews.net/glitz/bongobd-makes-premium-contents-free-its-subscribers-61234	**‘Bongobd’ makes premium contents free for its subscribers**

Date of the news	News URL	Title of the news
26/03/2020	https://www.tbsnews.net/bangladesh/energy/prepaid-household-gas-consumers-get-emergency-balance-tk-2000-61408	**Titas, Karnaphuli Gas increase emergency balance for domestic users**
26/03/2020	https://www.tbsnews.net/coronavirus-chronicle/coronavirus-bangladesh/niketan-takes-extraordinary-steps-protect-residents	**Niketan takes extraordinary steps to protect residents**
26/03/2020	https://www.tbsnews.net/economy/trade/online-shopping-surges-no-one-deliver-goods-61561	**Online shopping surges, but no one to deliver goods**
27/03/2020	https://www.tbsnews.net/coronavirus-chronicle/coronavirus-bangladesh/clifton-group-donating-10-lakh-masks-chattogram-61690	**Clifton Group donating 10 lakh masks in Chattogram**
27/03/2020	https://www.tbsnews.net/coronavirus-chronicle/coronavirus-bangladesh/bidyanondo-distribute-foods-among-people-worst-hit	**Bidyanondo to distribute foods among people worst hit by lockdown**
27/03/2020	https://www.tbsnews.net/coronavirus-chronicle/coronavirus-bangladesh/police-enforce-social-distancing-through-humiliation	**Police enforce social distancing through humiliation, harassment**
27/03/2020	https://www.tbsnews.net/coronavirus-chronicle/coronavirus-bangladesh/covid-19-are-we-preparing-homeless-and-slum-dwellers	**COVID-19: Are we preparing the homeless and slum-dwellers?**
27/03/2020	https://www.tbsnews.net/coronavirus-chronicle/coronavirus-bangladesh/factories-making-protective-gears-having-buying-orders	**Factories making protective gears, having buying orders need not to close**
27/03/2020	https://www.tbsnews.net/bangladesh/child-protection-priority-curbing-coronavirus-spread-61816	**'Child protection a priority in curbing coronavirus spread'**
27/03/2020	https://www.tbsnews.net/economy/rmg/accord-suspends-inspections-bangladesh-amid-corona-fear-61882	**Accord suspends inspections in Bangladesh amid corona fear**
27/03/2020	https://www.tbsnews.net/coronavirus-chronicle/coronavirus-bangladesh/bangladesh-receives-30000-coronavirus-testing-kits-jack	**Bangladesh receives Covid-19 testing kits from Jack Ma**
27/03/2020	https://www.tbsnews.net/bangladesh/shutdown-misery-daily-wage-earners-61909	**Shutdown a misery for daily wage-earners**
27/03/2020	https://www.tbsnews.net/bangladesh/keeping-us-clean-risking-their-lives-61873	**Keeping us clean, risking their lives**
28/03/2020	https://www.tbsnews.net/coronavirus-chronicle/coronavirus-bangladesh/gulshan-people-join-hands-keep-corona-bay-62032	**Gulshan people join hands to keep corona at bay**
28/03/2020	https://www.tbsnews.net/coronavirus-chronicle/coronavirus-bangladesh/beximco-provides-ppe-healthcare-professionals-62179	**Beximco provides PPE to healthcare professionals**
28/03/2020	https://www.tbsnews.net/coronavirus-chronicle/covid-19-bangladesh/bad-days-ahead-daily-wage-earners-62272	**Bad days ahead for daily wage-earners**
28/03/2020	https://www.tbsnews.net/bangladesh/govt-delivering-aid-ultra-poor-families-doorstep-62269	**Govt delivering aid to ultra-poor families' doorstep**

Date of the news	News URL	Title of the news
28/03/2020	https://www.tbsnews.net/coronavirus-chronicle/coronavirus-bangladesh/disinfectants-be-sprayed-each-area-capital-lgrd	**Disinfectants to be sprayed in each area of capital: LGRD minister**
28/03/2020	https://www.tbsnews.net/economy/chattogram-small-traders-fight-survival-62155	**Chattogram small traders fight for survival**
28/03/2020	https://www.tbsnews.net/coronavirus-chronicle/coronavirus-bangladesh/chattograms-low-income-workers-great-risk-62104	**Chattogram's low-income workers at great risk**
28/03/2020	https://www.tbsnews.net/coronavirus-chronicle/coronavirus-bangladesh/charity-defines-bangladesh-during-crisis-62086	**Charity that defines Bangladesh during crisis**
28/03/2020	https://www.tbsnews.net/coronavirus-chronicle/coronavirus-bangladesh/adb-provide-300000-support-bangladeshs-fight-against	**ADB to provide $300,000 to support Bangladesh's fight against corona**
28/03/2020	https://www.tbsnews.net/bangladesh/no-protection-conservancy-workers-chattogram-62077	**No protection for conservancy workers in Chattogram**
29/03/2020	https://www.tbsnews.net/companies/fbcci-provide-health-safety-equipment-healthcare-professionals-62605	**FBCCI to provide health safety equipment to healthcare professionals**
	https://www.tbsnews.net/coronavirus-chronicle/covid-19-bangladesh/bashundhara-proposes-build-5000-bed-coronavirus-hospital	**Bashundhara offers to build 5,000-bed coronavirus hospital**
	https://www.tbsnews.net/coronavirus-chronicle/covid-19-bangladesh/bgmea-plans-make-20000-ppe-suits-rubana-huq-62536	**BGMEA plans to make 20,000 PPE suits: Rubana Huq**
	https://www.tbsnews.net/coronavirus-chronicle/covid-19-bangladesh/rmg-factories-step-make-5-lakh-ppe-62374	**RMG factories step up to make 5 lakh PPE**
	https://www.tbsnews.net/coronavirus-chronicle/covid-19-bangladesh/corporates-lending-hand-fight-virus-62479	**Corporates lending a hand to fight virus**
	https://www.tbsnews.net/coronavirus-chronicle/covid-19-bangladesh/jack-ma-donates-30000-more-coronavirus-testing-kits	**Jack Ma donates 30,000 more coronavirus testing kits to Bangladesh**
	https://www.tbsnews.net/coronavirus-chronicle/covid-19-bangladesh/bpo-transports-ppes-test-kits-civil-surgeon-offices-62470	**Postal service launches nationwide free delivery of medical equipment**
	https://www.tbsnews.net/coronavirus-chronicle/covid-19-bangladesh/patient-contact-without-ppe-sends-more-doctors-nurses	**Patient contact without PPE sends more doctors, nurses to quarantine**
	https://www.tbsnews.net/coronavirus-chronicle/covid-19-bangladesh/bangladeshi-physician-sending-ppe-china-62419	**Bangladeshi physician sending PPE from China**
	tbsnews.net/coronavirus-chronicle/covid-19-bangladesh/not-coronavirus-its-hunger-we-fear-62395	**'Not coronavirus, it's hunger that we fear'**

Date of the news	News URL	Title of the news
	https://www.tbsnews.net/coronavirus-chronicle/covid-19-bangladesh/saber-chowdhury-donates-700-ppe-mugda-hospital-62335	**Saber Chowdhury donates 700 PPE to Mugda hospital**
03/30/2020	https://www.tbsnews.net/coronavirus-chronicle/covid-19-bangladesh/govt-media-work-together-tackle-coronavirus-crisis-hasan	**Govt, media to work together to tackle coronavirus crisis: Hasan**
	https://www.tbsnews.net/coronavirus-chronicle/covid-19-bangladesh/low-income-people-get-food-support-countrywide-ministry	**Low-income people to get food support countrywide: Ministry**
	https://www.tbsnews.net/bangladesh/health/inmates-face-coronavirus-risk-crowded-jails-62860	**Inmates face coronavirus risk in crowded jails**
	https://www.tbsnews.net/coronavirus-chronicle/covid-19-bangladesh/coronavirus-us-bangla-provide-ppe-medical-college	**Coronavirus: US Bangla to provide PPE to medical college hospitals free of cost**
	https://www.tbsnews.net/coronavirus-chronicle/covid-19-bangladesh/just-45-ventilators-fight-against-coronavirus-62704	**Just 45 ventilators in fight against coronavirus**
	https://www.tbsnews.net/companies/walton-gives-employees-tk77cr-profit-earnings-62695	**Walton gives employees Tk77cr from profit earnings**
	https://www.tbsnews.net/economy/sadarghat-boatmen-bear-brunt-shutdown-62683	**Sadarghat boatmen bear brunt of shutdown**
	https://www.tbsnews.net/coronavirus-chronicle/covid-19-bangladesh/modality-pms-tk5000-crore-covid-19-fund-be-set-today-62677	**Modality for PM's Tk5,000 crore Covid-19 fund to be set today**
	https://www.tbsnews.net/coronavirus-chronicle/covid-19-bangladesh/coronavirus-army-donates-tk-25cr-pms-relief-fund-62650	**Coronavirus: Army donates Tk 25cr to PM's relief fund**
	https://www.tbsnews.net/coronavirus-chronicle/covid-19-bangladesh/clean-green-bangladesh-and-garib-foundation-distribute	**Clean Green Bangladesh and Garib Foundation distribute food items in Dhaka**
	https://www.tbsnews.net/coronavirus-chronicle/covid-19-bangladesh/momtaz-partners-brac-calls-defeat-coronavirus-63001	**Folk queen partners with Brac to fight coronavirus socially**
	https://www.tbsnews.net/bangladesh/health/stadiums-can-be-used-hospitals-coronavirus-patients-63007	**'Stadiums can be used as hospitals for coronavirus patients'**
	https://www.tbsnews.net/coronavirus-chronicle/covid-19-bangladesh/17-more-labs-be-set-corona-sample-test-health-minister	**17 more labs to be set up for corona sample test: health minister**<br><br>**(*P.S Not sure if private included*)**
	https://www.tbsnews.net/coronavirus-chronicle/covid-19-bangladesh/summit-group-provides-govt-tk3-crore-fight-coronavirus	**Summit Group provides govt Tk3 crore to fight coronavirus**
	https://www.tbsnews.net/economy/banking/prime-bank-takes-measures-fight-coronavirus-spread-62983	**Prime Bank takes measures to fight coronavirus spread**

Date of the news	News URL	Title of the news
	https://www.tbsnews.net/coronavirus-chronicle/covid-19-bangladesh/coronavirus-police-donate-boishakhi-allowance-pms-relief	**Coronavirus: Police to donate Boishakhi allowance to PM's relief and welfare fund**
	https://www.tbsnews.net/coronavirus-chronicle/covid-19-bangladesh/coronavirus-islamic-foundation-issues-guidelines-over	**Coronavirus: Islamic Foundation issues guidelines over namaz, safety**
	https://www.tbsnews.net/bangladesh/cmp-along-ksrm-lend-hand-3000-families-62959	**CMP along with KSRM lend a hand to 3,000 families**
	https://www.tbsnews.net/bangladesh/crime/man-fined-selling-cough-syrup-coronavirus-vaccine-62947	**Man fined for selling cough syrup as coronavirus vaccine**
	https://www.tbsnews.net/coronavirus-chronicle/covid-19-bangladesh/ucb-donates-pms-relief-fund-tackle-coronavirus-62950	**UCB donates to PM's relief fund to tackle coronavirus**
	https://www.tbsnews.net/bangladesh/ccnf-holds-online-press-conference-journalists-dhaka-and-coxs-bazar-62998	**CCNF holds online press conference with journalists from Dhaka and Cox's Bazar**
	https://www.tbsnews.net/coronavirus-chronicle/covid-19-bangladesh/virus-fallout-forces-many-chattogram-domestic-workers-beg	**Virus fallout forces many of Chattogram domestic workers to beg**
03/31/2020	https://www.tbsnews.net/economy/protecting-people-economy-top-priority-finance-minister-63490	**Protecting people, economy is top priority: Finance minister**
	https://www.tbsnews.net/coronavirus-chronicle/covid-19-bangladesh/navana-group-give-2-storey-building-chattogram-corona	**Navana group to give 2-storey building in Chattogram for corona hospital**
	https://www.tbsnews.net/coronavirus-chronicle/covid-19-bangladesh/coxs-bazar-communities-making-masks-aid-fight-against	**Cox's Bazar communities making masks to aid fight against Covid-19**
	https://www.tbsnews.net/coronavirus-chronicle/covid-19-bangladesh/us-bangla-group-set-temporary-hospital-coronavirus	**US-Bangla Group to set up temporary hospital for coronavirus patients in Narayanganj**
	https://www.tbsnews.net/coronavirus-chronicle/covid-19-bangladesh/rajshahi-district-administration-launches-mobile-vegetable	**Rajshahi district administration launches mobile vegetable market**
	https://www.tbsnews.net/coronavirus-chronicle/covid-19-bangladesh/fbcci-spray-disinfectant-narayanganj-uttara-63430	**FBCCI to spray disinfectant Narayanganj-Uttara**
	https://www.tbsnews.net/coronavirus-chronicle/covid-19-bangladesh/rumours-over-coronavirus-police-looking-82-admins-social	**Police looking for 82 admins of social media pages**
	https://www.tbsnews.net/companies/walton-make-life-saving-medical-equipment-ventilators-63421	**Walton to make life-saving medical equipment**
	https://www.tbsnews.net/coronavirus-chronicle/covid-19-bangladesh/young-engineers-claim-have-developed-robot-handle-covid	**Young engineers claim to have developed robot to handle COVID patients**

Date of the news	News URL	Title of the news
	https://www.tbsnews.net/coronavirus-chronicle/covid-19-bangladesh/pran-rfl-group-sets-isolation-unit-natore-63361	**PRAN-RFL Group sets up isolation unit in Natore**
	https://www.tbsnews.net/coronavirus-chronicle/covid-19-bangladesh/pran-rfl-prepares-isolation-unit-natore-treat-corona	**PRAN-RFL prepares isolation unit in Natore to treat corona patient**
	https://www.tbsnews.net/bangladesh/commerce-minister-calls-homeowners-consider-workers-rents-sympathy-63358	**Commerce minister calls on homeowners to consider workers' rents with sympathy**
	https://www.tbsnews.net/coronavirus-chronicle/covid-19-bangladesh/afc-health-fortis-heart-institute-opens-corona-care	**AFC Health Fortis Heart Institute opens corona care hotline**
	https://www.tbsnews.net/coronavirus-chronicle/covid-19-bangladesh/china-committed-supporting-bangladesh-fighting-covid-19	**China committed to supporting Bangladesh in fighting Covid-19 pandemic: Chinese ambassador**
	https://www.tbsnews.net/coronavirus-chronicle/covid-19-bangladesh/lack-soap-water-failing-dhaka-wasas-noble-initiative-63274	**Lack of soap, water failing Dhaka Wasa's noble initiative**
	https://www.tbsnews.net/coronavirus-chronicle/covid-19-bangladesh/mill-owners-donate-50-tonnes-rice-day-labourers-bogura	**Mill owners donate 50 tonnes rice for day labourers in Bogura**
	https://www.tbsnews.net/coronavirus-chronicle/covid-19-bangladesh/jubo-league-man-arrested-spreading-rumour-about	**Jubo League man arrested for spreading rumour about coronavirus**
	https://www.tbsnews.net/coronavirus-chronicle/covid-19-bangladesh/fake-hand-sanitisers-selling-original-bottles-63181	**Fake hand sanitisers selling in original bottles**
01/04/2020	https://www.tbsnews.net/economy/banking/36-private-banks-donate-tk137-crore-pms-fund-sunday-63955	36 private banks to donate Tk137 crore to PM's fund Sunday
01/04/2020	https://www.tbsnews.net/bangladesh/telecom/gp-donate-50000-ppes-and-10000-pcr-kits-coronavirus-hospitals-63814	GP to donate 50,000 PPEs and 10,000 PCR kits to coronavirus hospitals
01/04/2020	https://www.tbsnews.net/coronavirus-chronicle/covid-19-bangladesh/rt-pcr-machine-can-test-96-samples-3-hours-detect	RT-PCR machine can test 96 samples in 3 hours to detect coronavirus
01/04/2020	https://www.tbsnews.net/coronavirus-chronicle/covid-19-bangladesh/tom-tailor-bangladesh-donate-staffs-one-day-salary-bgmea	Tom Tailor Bangladesh to donate staff's one day salary to BGMEA fund
01/04/2020	https://www.tbsnews.net/coronavirus-chronicle/covid-19-bangladesh/undpunited-group-join-hands-covid-19-response-63865	UNDP,United Group join hands for COVID-19 response
01/04/2020	https://www.tbsnews.net/bangladesh/pathaos-tong-expands-its-geographical-reach-shwapno-63586	Pathao's 'tong' expands its geographical reach with Shwapno
01/04/2020	https://www.tbsnews.net/coronavirus-chronicle/covid-19-bangladesh/govt-block-50-websites-82-facebook-pages-spreading-rumours	Govt to block 50 websites, 82 Facebook pages for spreading rumours
01/04/2020	https://www.tbsnews.net/coronavirus-chronicle/covid-19-bangladesh/relief-distribution-begins-bogura-63940	Relief distribution begins in Bogura

Date of the news	News URL	Title of the news
01/04/2020	https://www.tbsnews.net/coronavirus-chronicle/covid-19-bangladesh/bangladesh-navy-distributes-relief-among-poor-chattogram	Bangladesh Navy distributes relief among poor in Chattogram
01/04/2020	https://www.tbsnews.net/economy/trade/shop-owners-seek-tk2500-crore-financial-support-63976	Shop owners seek Tk2,500 crore financial support
01/04/2020	https://www.tbsnews.net/coronavirus-chronicle/covid-19-bangladesh/owners-can-run-factories-if-they-want-commerce-minister	Owners can run factories, if they want: Commerce minister
01/04/2020	https://www.tbsnews.net/coronavirus-chronicle/covid-19-bangladesh/doctors-urged-provide-healthcare-services-patients-63877	Doctors urged to provide healthcare services to patients
01/04/2020	https://www.tbsnews.net/coronavirus-chronicle/covid-19-bangladesh/six-fined-spreading-rumours-over-coronavirus-barishal	Six fined for spreading rumours over coronavirus in Barishal
01/04/2020	https://www.tbsnews.net/bangladesh/crime/man-arrested-dhaka-spreading-rumours-over-coronavirus-63856	Man arrested in Dhaka for spreading rumours over coronavirus
01/04/2020	https://www.tbsnews.net/coronavirus-chronicle/covid-19-bangladesh/mobile-court-fines-jti-tk-10000-rajshahi-63847	Mobile Court fines JTI Tk 10,000 in Rajshahi
01/04/2020	https://www.tbsnews.net/coronavirus-chronicle/covid-19-bangladesh/finance-ministry-reveals-guideline-tk5000cr-stimulus	Finance Ministry reveals guideline for Tk5,000cr stimulus package
01/04/2020	https://www.tbsnews.net/bangladesh/50-restaurant-staff-could-lose-jobs-63799	50% restaurant staff could lose jobs
01/04/2020	https://www.tbsnews.net/coronavirus-chronicle/covid-19-bangladesh/steps-taken-meet-milk-egg-fish-meat-crisis-minister-63772	Steps taken to meet milk, egg, fish, meat crisis: Minister
01/04/2020	https://www.tbsnews.net/panorama/empty-streets-humanity-forms-new-bonds-survival-63691	On empty streets, humanity forms new bonds for survival
01/04/2020	https://www.tbsnews.net/coronavirus-chronicle/covid-19-bangladesh/free-transport-service-doctors-nurses-begins-63682	Free transport service for doctors, nurses begins
01/04/2020	https://www.tbsnews.net/coronavirus-chronicle/covid-19-bangladesh/no-possibility-sheltering-homeless-chattogram-dc-63673	No possibility of sheltering the homeless: Chattogram DC
01/04/2020	https://www.tbsnews.net/bangladesh/over-3-lakh-tourism-workers-may-lose-jobs-pata-63982	Over 3 lakh tourism workers may lose jobs: Pata
02/04/2020	https://www.tbsnews.net/coronavirus-chronicle/covid-19-bangladesh/uber-provide-transport-medical-professionals-64423	Uber to provide transport to medical professionals
02/04/2020	https://www.tbsnews.net/coronavirus-chronicle/covid-19-bangladesh/robi-use-crowdsourced-data-helping-govt-tackle-coronavirus	Robi to use crowdsourced data for helping govt tackle coronavirus
02/04/2020	https://www.tbsnews.net/companies/countrys-leading-e-commerce-companies-working-together-64402	Country's leading e-commerce companies working together
02/04/2020	https://www.tbsnews.net/bangladesh/brac-mobilises-tk15cr-fund-responding-virus-fallout-64420	Brac mobilises Tk15cr fund responding to virus fallout
02/04/2020	https://www.tbsnews.net/economy/trade/bangladesh-lifts-ban-export-masks-hand-sanitizers-64366	Export ban on mask, hand sanitiser lifted
02/04/2020	https://www.tbsnews.net/markets/oms-rice-be-sold-tk10-kg-64357	OMS rice to be sold at Tk10 per kg
02/04/2020	https://www.tbsnews.net/bangladesh/health/fbcci-distributing-ppe-masks-hospitals-essential-service-workers-64339	FBCCI distributing PPE, masks to hospitals, essential service workers

Date of the news	News URL	Title of the news
02/04/2020	https://www.tbsnews.net/sports/91-first-class-cricketers-donate-their-salaries-battle-coronavirus-64282	91 first-class cricketers donate their salaries to battle Coronavirus
02/04/2020	https://www.tbsnews.net/coronavirus-chronicle/covid-19-bangladesh/banglalink-launches-digital-healthcare-platform-daktarbhai	'Daktarbhai' offers free health services to Banglalink subscribers
02/04/2020	https://www.tbsnews.net/bangladesh/adb-provides-134-million-avoid-skills-trainees-drop-out-due-covid-19-lockdown-64261	ADB provides $1.34 million to avoid skills trainees' drop out due to Covid-19 lockdown
02/04/2020	https://www.tbsnews.net/coronavirus-chronicle/covid-19-bangladesh/substandard-ppe-floods-dhakas-markets-64042	Substandard PPE floods Dhaka's markets
02/04/2020	https://www.tbsnews.net/coronavirus-chronicle/covid-19-bangladesh/govt-giving-aid-beneficiary-list-not-finalised-yet-64186	Govt giving aid, but beneficiary list not finalised yet
02/04/2020	https://www.tbsnews.net/coronavirus-chronicle/covid-19-bangladesh/six-labs-work-testing-so-little-64081	Six labs at work but testing so little!
02/04/2020	https://www.tbsnews.net/coronavirus-chronicle/covid-19-bangladesh/bangladesh-coast-guard-distributes-masks-and-soap-among	Bangladesh Coast Guard distributes masks and soap among poor people
02/04/2020	https://www.tbsnews.net/coronavirus-chronicle/covid-19-bangladesh/protest-front-police-station-chattogram-relief-64411	Protest in front of police station in Chattogram for relief
03/04/2020	https://www.tbsnews.net/coronavirus-chronicle/covid-19-bangladesh/consult-doctors-says-pm-issuing-31-directives-64459	Consult doctors, says PM issuing 31 directives
03/04/2020	https://www.tbsnews.net/sports/shakibs-foundation-provide-coronavirus-testing-kits-hospitals-64714	Shakib's foundation to provide coronavirus testing kits for hospitals
03/04/2020	https://www.tbsnews.net/coronavirus-chronicle/covid-19-bangladesh/ityadi-stands-poor-fight-coronavirus-64648	Ityadi stands by poor to fight coronavirus
03/04/2020	https://www.tbsnews.net/coronavirus-chronicle/covid-19-bangladesh/how-private-citizens-are-making-who-grade-ppe-64453	How private citizens are making WHO-grade PPE
03/04/2020	https://www.tbsnews.net/bangladesh/stealing-poors-rice-64789	Stealing the poor's rice
03/04/2020	https://www.tbsnews.net/economy/trade/dcci-seeks-emergency-fund-smes-informal-sector-64783	DCCI seeks emergency fund for SMEs, informal sector
03/04/2020	https://www.tbsnews.net/coronavirus-chronicle/covid-19-bangladesh/police-introduce-free-food-home-ctg-64744	Police introduce 'free food at home' in Ctg
03/04/2020	https://www.tbsnews.net/glitz/iflix-content-made-free-amid-coronavirus-64690	iflix content made free amid coronavirus
03/04/2020	https://www.tbsnews.net/coronavirus-chronicle/covid-19-bangladesh/no-aid-14-lakh-ctg-slum-dwellers-due-incoordination-64576	No aid for 14 lakh Ctg slum dwellers due to incoordination
03/04/2020	https://www.tbsnews.net/bangladesh/health/diagnostic-centres-dhaka-wear-vacant-look-64537	Diagnostic centres in Dhaka wear a deserted look
04/04/2020	https://www.tbsnews.net/coronavirus-chronicle/covid-19-bangladesh/dhaka-south-hotlines-see-big-increase-calls-65191	Dhaka South hotlines see big increase in calls
04/04/2020	https://www.tbsnews.net/economy/shipbuilders-demand-incentive-package-fight-covid-19-impacts-65173	Shipbuilders demand incentive package to fight Covid-19 impacts
04/04/2020	https://www.tbsnews.net/coronavirus-chronicle/covid-19-bangladesh/private-university-students-taking-online-classes-64867	Private university students taking online classes

Date of the news	News URL	Title of the news
04/04/2020	https://www.tbsnews.net/coronavirus-chronicle/covid-19-bangladesh/jhorapata-foundation-stands-beside-helpless-65167	Jhorapata foundation stands beside the helpless
04/04/2020	https://www.tbsnews.net/coronavirus-chronicle/covid-19-bangladesh/asa-provide-food-15-lakh-poor-65152	ASA to provide food to 1.5 lakh poor
04/04/2020	https://www.tbsnews.net/bangladesh/health/bangladesh-makes-favipiravir-treat-covid-19-patients-65143	Beximco, Beacon pharma make Japanese Covid-19 drug
04/04/2020	https://www.tbsnews.net/coronavirus-chronicle/covid-19-bangladesh/idlc-launches-month-long-food-distribution-programme	IDLC launches month-long food distribution programme for corona-hit people
04/04/2020	https://www.tbsnews.net/coronavirus-chronicle/covid-19-bangladesh/bashundhara-group-gives-police-25-thousand-masks-65098	Bashundhara Group gives police 25 thousand masks
04/04/2020	https://www.tbsnews.net/coronavirus-chronicle/covid-19-bangladesh/mashrafes-foundation-provide-medical-service-amid	Mashrafe's foundation to provide medical service amid coronavirus
04/04/2020	https://www.tbsnews.net/coronavirus-chronicle/covid-19-bangladesh/shakibs-foundation-confidence-group-donate-tk20lakh-combat	Shakib's foundation with Confidence Group to donate Tk20lakh to combat Covid-19
04/04/2020	https://www.tbsnews.net/bangladesh/health/voluntary-medical-responses-time-social-distancing-64969	Voluntary medical responses in the time of social distancing
04/04/2020	https://www.tbsnews.net/coronavirus-chronicle/covid-19-bangladesh/bangladesh-scouts-conduct-online-awareness-campaign-covid	Bangladesh Scouts conduct online awareness campaign on Covid-19
04/04/2020	https://www.tbsnews.net/coronavirus-chronicle/covid-19-bangladesh/world-bank-fast-tracks-100m-coronavirus-support-bangladesh	Bangladesh to get $100m from World Bank to fight Coronavirus
04/04/2020	https://www.tbsnews.net/bangladesh/people-rushing-towards-dhaka-foot-65014	A perilous return of Bangladeshi garment workers amid shutdown
04/04/2020	https://www.tbsnews.net/coronavirus-chronicle/covid-19-bangladesh/1-lakh-chattogram-transport-workers-face-bleak-future	1 lakh Chattogram transport workers face bleak future
04/04/2020	https://www.tbsnews.net/coronavirus-chronicle/covid-19-bangladesh/consider-closing-rmg-factories-tackle-coronavirus-dscc	Consider closing RMG factories to tackle coronavirus: DSCC Mayor
04/04/2020	https://www.tbsnews.net/coronavirus-chronicle/covid-19-bangladesh/dncrp-commerce-ministry-teams-monitor-markets-hospitals	DNCRP, Commerce Ministry teams monitor markets, hospitals
04/04/2020	https://www.tbsnews.net/coronavirus-chronicle/covid-19-bangladesh/maleque-concerted-efforts-contain-spread-covid-19-65134	Maleque for concerted efforts to contain spread of COVID-19
04/04/2020	https://www.tbsnews.net/bangladesh/crime/chattogram-chairman-embezzled-govt-relief-65161	Chattogram UP chairman embezzled govt relief?
04/04/2020	https://www.tbsnews.net/bangladesh/crime/555-sacks-govt-rice-seized-jashore-2-held-65164	555 sacks of govt rice seized in Jashore, 2 held
05/04/2020	https://www.tbsnews.net/coronavirus-chronicle/covid-19-bangladesh/ksrm-donates-ppe-healthcare-professionals-chattogram-65614	KSRM donates PPE to healthcare professionals in Chattogram
05/04/2020	https://www.tbsnews.net/economy/banking/al-arafah-islami-bank-donates-tk5-crore-prime-ministers-relief-fund-65578	Al-Arafah Islami Bank donates Tk5 crore to prime minister’s relief fund
05/04/2020	https://www.tbsnews.net/coronavirus-chronicle/covid-19-bangladesh/pran-rfl-lends-hand-coronavirus-hit-people-65563	Pran-Rfl lends hand to coronavirus-hit people

Date of the news	News URL	Title of the news
05/04/2020	https://www.tbsnews.net/coronavirus-chronicle/covid-19-bangladesh/bangladesh-investment-bankers-community-support-65551	Bangladesh investment bankers in community support
05/04/2020	https://www.tbsnews.net/coronavirus-chronicle/covid-19-bangladesh/jci-dhaka-east-distributes-essentials-350-families-65446	JCI Dhaka East distributes essentials to 350 families
05/04/2020	https://www.tbsnews.net/coronavirus-chronicle/covid-19-bangladesh/consumer-rights-protection-directorate-monitor-hospitals	Consumer rights protection directorate to monitor hospitals
05/04/2020	https://www.tbsnews.net/economy/rmg/garment-workers-big-trouble-65635	Garment workers in a big trouble
05/04/2020	https://www.tbsnews.net/companies/egeneration-provide-free-enablement-and-training-remote-work-tool-65371	eGeneration to provide free enablement and training of remote work tool
05/04/2020	https://www.tbsnews.net/coronavirus-chronicle/covid-19-bangladesh/abandoned-gloves-can-spread-covid-19-65416	Abandoned gloves can spread Covid-19
06/04/2020	https://www.tbsnews.net/coronavirus-chronicle/covid-19-bangladesh/sonali-rupali-and-janata-donate-tk375cr-pms-relief-fund	Sonali, Rupali and Janata donate Tk3.75cr to PM’s relief fund
06/04/2020	https://www.tbsnews.net/coronavirus-chronicle/covid-19-bangladesh/agrani-bank-donates-tk125cr-pms-relief-fund-66079	Agrani Bank donates Tk1.25cr to PM’s relief fund
06/04/2020	https://www.tbsnews.net/coronavirus-chronicle/covid-19-bangladesh/nrbc-bank-forms-health-desk-fight-coronavirus-66067	NRBC Bank forms health desk to fight coronavirus
06/04/2020	https://www.tbsnews.net/coronavirus-chronicle/covid-19-bangladesh/marico-donates-tk50-lakh-pms-relief-and-welfare-fund-66064	Marico donates Tk50 lakh to PM’s Relief and Welfare Fund
06/04/2020	https://www.tbsnews.net/coronavirus-chronicle/covid-19-bangladesh/merchant-bankers-lend-hand-poor-66010	Merchant bankers lend a hand to the poor
06/04/2020	https://www.tbsnews.net/coronavirus-chronicle/covid-19-bangladesh/salma-adil-foundation-distributes-ppe-chandanaish-65935	Salma-Adil Foundation distributes PPE in Chandanaish
06/04/2020	https://www.tbsnews.net/bangladesh/police-donate-tk-20-crore-pms-relief-fund-65806	Police donate Tk 20 crore to PM's relief fund
	https://www.tbsnews.net/coronavirus-chronicle/covid-19-bangladesh/coast-guard-donates-one-day-salary-pms-relief-fund-65932	Coast Guard donates one-day salary to PM's relief fund
06/04/2020	https://www.tbsnews.net/coronavirus-chronicle/covid-19-bangladesh/jamuna-group-donates-tk10-crore-pms-relief-fund-65812	Jamuna Group donates Tk10 crore to PM’s relief fund
06/04/2020	https://www.tbsnews.net/coronavirus-chronicle/covid-19-bangladesh/fbcci-donates-tk2cr-pms-relief-fund-fight-coronavirus	FBCCI donates Tk2cr to PM's relief fund to fight coronavirus
06/04/2020	https://www.tbsnews.net/coronavirus-chronicle/covid-19-bangladesh/red-crescent-launches-psychosocial-support-cell-covid-19	Red Crescent launches ‘psychosocial support cell’ on COVID-19
	https://www.tbsnews.net/bangladesh/pabna-lged-executive-engineer-accused-extorting-money-66028	Pabna LGED executive engineer accused of extorting money
	https://www.tbsnews.net/bangladesh/telecom/airtel-offers-lucrative-stay-home-packages-users-66061	Airtel offers lucrative stay-home packages for users
	https://www.tbsnews.net/economy/rmg/bgmea-bkmea-decide-shut-all-factories-till-april-14-66031	BGMEA, BKMEA decide to shut all factories till April 14

Date of the news	News URL	Title of the news
	https://www.tbsnews.net/coronavirus-chronicle/covid-19-bangladesh/sigminds-ai-recognises-faces-wearing-masks-65938	Sigmind's AI recognises faces wearing masks
07/04/2020	https://www.tbsnews.net/coronavirus-chronicle/covid-19-bangladesh/united-group-contributes-tk5-crore-fight-pandemic-covid-19	**United group contributes Tk5 crore to fight pandemic Covid-19**
08/04/2020	https://www.tbsnews.net/sports/u-19-champions-fund-battle-coronavirus-66910	**U-19 champions for fund to battle coronavirus**
08/04/2020	https://www.tbsnews.net/companies/empowering-youth-fight-covid-19-enriching-digital-maps-66883	**Empowering the youth to fight COVID-19 by enriching digital maps**
08/04/2020	https://www.tbsnews.net/tech/gpa2i-moves-update-google-map-help-emergency-services-66811	**GP, a2i updating digital maps to support emergency services**
09/04/2020	https://www.tbsnews.net/coronavirus-chronicle/covid-19-bangladesh/bangladeshi-it-firm-develops-low-cost-ventilator-67141	**Bangladeshi IT firm develops low-cost ventilator**
09/04/2020	https://www.tbsnews.net/coronavirus-chronicle/covid-19-bangladesh/shutdown-couldnt-stop-shahajpath-high-school-67111	**Shutdown couldn't stop Shahajpath High School**
10/04/2020	https://www.tbsnews.net/coronavirus-chronicle/covid-19-bangladesh/hellodoc24-renders-free-coronavirus-counseling-67618	**HelloDoc24 renders free coronavirus counseling**
10/04/2020	https://www.tbsnews.net/coronavirus-chronicle/covid-19-bangladesh/extreme-poverty-rises-60-percent-amid-covid-19-brac-survey	**Extreme poverty rises 60% amid Covid-19 outbreak: Brac survey**
10/04/2020	https://www.tbsnews.net/bangladesh/34-healthcare-staffers-lose-jobs-chattogram-67423	**34 healthcare staffers lose jobs in Chattogram**
11/04/2020	https://www.tbsnews.net/coronavirus-chronicle/covid-19-bangladesh/covid-19-ypsa-distributes-relief-1070-ship-breaking-0	**Covid-19: Ypsa distributes relief to 1070 ship-breaking labourers**
11/04/2020	https://www.tbsnews.net/coronavirus-chronicle/covid-19-bangladesh/actionaid-bangladeshs-response-covid-19-67912	**ActionAid Bangladesh's response to Covid-19**
11/04/2020	https://www.tbsnews.net/coronavirus-chronicle/covid-19-bangladesh/php-family-distributes-relief-10000-families-donates-tk2	**PHP Family distributes relief to 10,000 families, donates Tk2 cr to PM fund**
11/04/2020	https://www.tbsnews.net/coronavirus-chronicle/covid-19-bangladesh/gsk-partners-care-bangladesh-support-covid-19-affected	**GSK partners with Care Bangladesh to support Covid-19 affected people**
11/04/2020	https://www.tbsnews.net/coronavirus-chronicle/covid-19-bangladesh/celebrate-bangla-new-year-shubho-noboborsho-bot-67837	**Celebrate Bangla New Year with 'Shubho Noboborsho Bot'**
11/04/2020	https://www.tbsnews.net/coronavirus-chronicle/covid-19-bangladesh/unilever-bangladesh-pledges-tk200-m-tackle-covid-19-67825	**Unilever Bangladesh pledges Tk200 m to tackle Covid-19**
11/04/2020	https://www.tbsnews.net/coronavirus-chronicle/covid-19-bangladesh/muslim-ambassadors-provided-6-tonnes-food-aid-poor-67813	**Muslim ambassadors provided 6 tonnes of food aid to the poor**
11/04/2020	https://www.tbsnews.net/coronavirus-chronicle/covid-19-bangladesh/switzerland-backs-bangladeshs-covid-19-response-67717	**Switzerland backs Bangladesh's COVID-19 response**
12/04/2020	https://www.tbsnews.net/coronavirus-chronicle/covid-19-bangladesh/bgmea-provides-relief-covid-19-affected-people-68368	**BGMEA provides relief for Covid-19 affected people**
12/04/2020	https://www.tbsnews.net/coronavirus-chronicle/covid-19-bangladesh/mashrafe-lauds-nagads-initiatives-fighting-covid-19-68356	**Mashrafe lauds Nagad's initiatives for fighting Covid-19**

Date of the news	News URL	Title of the news
12/04/2020	https://www.tbsnews.net/coronavirus-chronicle/covid-19-bangladesh/bangladesh-scouts-distribute-soap-bars-fight-covid-19	**Bangladesh Scouts to distribute soap bars to fight Covid-19**
13/04/2020	https://www.tbsnews.net/tech/huawei-assists-bd-china-medical-experts-sharing-knowledge-over-covid-19-68767	**Huawei assists BD-China medical experts sharing knowledge over Covid-19**
13/04/2020	https://www.tbsnews.net/coronavirus-chronicle/covid-19-bangladesh/ceab-launches-help-them-stay-home-support-bangladeshis	**CEAB launches 'Help them stay home' to support Bangladeshis**
13/04/2020	https://www.tbsnews.net/economy/banking/bankers-get-months-salary-working-10-day-during-shutdown-68572	**Bank employees working during shutdown to get double basic salary**
14/04/2020	https://www.tbsnews.net/coronavirus-chronicle/covid-19-bangladesh/govt-incorporates-ngo-testing-covid-19-69172	**Govt incorporates NGO in testing COVID-19**
14/04/2020	https://www.tbsnews.net/glitz/celebrate-pohela-boishakh-live-music-and-events-online-69037	**Celebrate Pohela Boishakh with live music and events online**
14/04/2020	https://www.tbsnews.net/glitz/bangladeshi-influencers-fighting-coronavirus-instagram-68959	**Bangladeshi influencers fighting coronavirus on Instagram**
14/04/2020	https://www.tbsnews.net/bangladesh/health/mobile-team-treats-general-patients-sylhet-68929	**Mobile team treats general patients in Sylhet**
15/04/2020	https://www.tbsnews.net/bangladesh/bcs-taxation-association-donates-one-day-salary-pms-relief-and-welfare-fund-69742	**BCS Taxation Association donates one day salary to PM's Relief and Welfare Fund**
15/04/2020	https://www.tbsnews.net/companies/pran-rfl-provides-food-40000-families-69727	**Pran-Rfl provides food for 40,000 families**
15/04/2020	https://www.tbsnews.net/bangladesh/education/icone-organises-online-educational-events-during-covid-19-crisis-69721	**Icone organises online educational events during Covid-19 crisis**
15/04/2020	https://www.tbsnews.net/companies/lafargeholcim-distributes-food-among-workers-69709	**LafargeHolcim distributes food among workers**
15/04/2020	https://www.tbsnews.net/coronavirus-chronicle/covid-19-bangladesh/city-bank-distributes-food-among-1-lakh-poor-69694	**City Bank distributes food among 1 lakh poor**
15/04/2020	https://www.tbsnews.net/coronavirus-chronicle/covid-19-bangladesh/grameen-shikkha-distributes-emergency-foods-mirpur-slums	**Grameen Shikkha distributes emergency foods in Mirpur slums**
15/04/2020	https://www.tbsnews.net/economy/rmg/bkash-steps-help-apparel-workers-get-salary-69523	**bKash steps up to help apparel workers get salary**
15/04/2020	https://www.tbsnews.net/thoughts/private-sector-reacting-enough-69463	**Private sector is reacting but is that enough?**
16/04/2020	https://www.tbsnews.net/companies/brac-driving-school-raising-covid-19-awareness-70186	**BRAC Driving School raising COVID-19 awareness**
16/04/2020	https://www.tbsnews.net/companies/lafargeholcim-declares-layoff-dhaka-office-70171	**LafargeHolcim declares layoff at Dhaka office**
16/04/2020	https://www.tbsnews.net/companies/grameen-telecom-working-ensure-safety-doctors-and-healthcare-workers-70108	**Grameen Telecom working to ensure safety of doctors and healthcare workers**
16/04/2020	https://www.tbsnews.net/coronavirus-chronicle/covid-19-bangladesh/brac-gives-430-rooms-set-quarantine-centre-70006	**Brac gives 430 rooms to set up quarantine centre**
16/04/2020	https://www.tbsnews.net/glitz/musicians-collaborate-shwapnos-effort-combat-covid-19-69835	**Musicians collaborate with Shwapno's effort to combat COVID-19**

Date of the news	News URL	Title of the news
17/04/2020	https://www.tbsnews.net/bangladesh/health/ibrahim-cardiac-ccu-closed-11-mitford-hospital-staff-test-covid-19-positive-70657	**Ibrahim Cardiac CCU closed; 11 Mitford Hospital staff test Covid-19 positive**
17/04/2020	https://www.tbsnews.net/economy/industry/pabna-bscic-busy-producing-essential-products-70645	**Pabna BSCIC busy producing essential products**
17/04/2020	https://www.tbsnews.net/coronavirus-chronicle/covid-19-bangladesh/brac-assist-1-lakh-more-families-cash-70585	**Brac to assist 1 lakh more families with cash**
17/04/2020	https://www.tbsnews.net/bangladesh/lafargeholcim-bangladesh-employees-be-fully-paid-during-layoff-70573	**LafargeHolcim Bangladesh employees to be fully paid during layoff**
18/04/2020	https://www.tbsnews.net/coronavirus-chronicle/covid-19-bangladesh/bpmca-walton-step-provide-ppes-sylhets-frontline	**BPMCA, Walton step up to provide PPEs for Sylhet's frontline healthcare professionals**
18/04/2020	https://www.tbsnews.net/coronavirus-chronicle/covid-19-bangladesh/chittagong-bourse-supports-4000-shutdown-hit-families	**Chittagong bourse supports 4,000 shutdown-hit families**
18/04/2020	https://www.tbsnews.net/markets/sunamganj-man-surprises-locals-selling-vegetables-token-price-70894	**Sunamganj man surprises locals selling vegetables at token price**
18/04/2020	https://www.tbsnews.net/coronavirus-chronicle/covid-19-bangladesh/grameen-kalyan-offers-healthcare-services-food-support	**Grameen Kalyan offers healthcare services, food support during pandemic**
18/04/2020	https://www.tbsnews.net/economy/rmg/over-95-workers-salary-disbursed-bgmea-70855	**Over 95% workers' salary disbursed: BGMEA**
19/04/2020	https://www.tbsnews.net/coronavirus-chronicle/covid-19-bangladesh/ngo-leaders-reaction-covid-19-response-ensure-inclusive	**NGO leaders' reaction to Covid-19 response: Ensure inclusive coordination at all levels**
19/04/2020	https://www.tbsnews.net/coronavirus-chronicle/covid-19-bangladesh/mr-noodles-lends-hand-coronavirus-hit-people-71377	**Mr Noodles lends hand to coronavirus-hit people**
19/04/2020	https://www.tbsnews.net/coronavirus-chronicle/covid-19-bangladesh/hsbc-continues-supports-keeping-covid-19-impact-down-71362	**HSBC continues supports for keeping Covid-19 impact down**
19/04/2020	https://www.tbsnews.net/coronavirus-chronicle/covid-19-bangladesh/mushfiqur-puts-historic-bat-auction-raise-funds-against	**Mushfiqur puts historic bat up for auction to raise funds against virus**
19/04/2020	https://www.tbsnews.net/coronavirus-chronicle/covid-19-bangladesh/front-line-medical-workers-receive-grameen-produced-ppes	**Front-line medical workers receive Grameen produced PPEs**
19/04/2020	https://www.tbsnews.net/coronavirus-chronicle/covid-19-bangladesh/ksrm-staff-donate-tk30-lakh-wages-chattogram-district	**KSRM staff donate Tk30 lakh from wages to Chattogram district relief fund**
19/04/2020	https://www.tbsnews.net/economy/banking/prime-bank-ready-return-deposits-demand-71293	**Prime Bank to support clients to recoup Covid-19 losses**
19/04/2020	https://www.tbsnews.net/bangladesh/praava-health-launches-telemedicine-services-during-covid-19-outbreak-71269	**Praava Health launches telemedicine services to assist patients during pandemic**
19/04/2020	https://www.tbsnews.net/tech/robi-a2i-launch-campaign-finding-ideas-overcome-covid-19-pandemic-71275	**Robi, a2i launch campaign finding ideas to overcome Covid-19 pandemic**
19/04/2020	https://www.tbsnews.net/bangladesh/districts/coxda-distributes-relief-goods-among-newspaper-hawkers-71257	**CoxDA distributes relief goods among newspaper hawkers**

Date of the news	News URL	Title of the news
20/04/2020	https://www.tbsnews.net/economy/guardian-life-insurance-launches-online-claims-tool-amid-shutdown-71719	**Guardian Life Insurance launches online claims tool amid shutdown**
20/04/2020	https://www.tbsnews.net/bangladesh/radisson-blu-chattogram-bay-view-strengthens-community-bond-through-business-activities	**Radisson Blu Chattogram Bay View strengthens community bond through business activities**
20/04/2020	https://www.tbsnews.net/coronavirus-chronicle/covid-19-bangladesh/sp-blames-rmg-workers-coronavirus-spread-gazipur-71488	**RMG owners blamed for Gazipur virus spike**
21/04/2020	https://www.tbsnews.net/coronavirus-chronicle/covid-19-bangladesh/hamdard-donates-tk25-lakh-pms-relief-fund-72241	**Hamdard donates Tk25 lakh to PM's relief fund**
21/04/2020	https://www.tbsnews.net/coronavirus-chronicle/covid-19-bangladesh/nrbc-bank-continues-distribution-ppes-medical-staff-72235	**NRBC Bank continues distribution of PPEs for medical staff**
21/04/2020	https://www.tbsnews.net/bangladesh/brac-phwc-and-kaan-petey-roi-launch-telecounselling-service-moner-jotno-mobile-e	**BRAC, PHWC and Kaan Petey Roi launch Telecounselling service 'Moner Jotno Mobile E' for anxieties related to Covid-19**
21/04/2020	https://www.tbsnews.net/coronavirus-chronicle/covid-19-bangladesh/covid-19-salma-adil-foundation-distributes-ppe-gazipur	**Covid-19: Salma-Adil Foundation distributes PPE in Gazipur**
21/04/2020	https://www.tbsnews.net/coronavirus-chronicle/covid-19-bangladesh/gk-covid-19-food-assistance-fund-group-donates-tk11-lakh	**GK Covid-19 Food Assistance Fund Group donates Tk11 lakh to Gonoshasthaya Kendra for providing food relief**
22/04/2020	https://www.tbsnews.net/coronavirus-chronicle/covid-19-bangladesh/birdem-closes-icu-patients-detected-covid-19-72679	**Birdem closes ICU as patients detected with Covid-19**
22/04/2020	https://www.tbsnews.net/coronavirus-chronicle/covid-19-bangladesh/global-ppe-shortage-amid-covid-19-72649	**Global PPE shortage amid Covid-19**
22/04/2020	https://www.tbsnews.net/coronavirus-chronicle/covid-19-bangladesh/ceab-distributes-relief-goods-among-250-daily-workers	**CEAB distributes relief goods among 250 daily workers**
22/04/2020	https://www.tbsnews.net/companies/banglalink-distribute-relief-among-14500-households-association-sena-kalyan-sangstha-and	**Banglalink to distribute relief among 14,500 households in association with Sena Kalyan Sangstha and Army**
22/04/2020	https://www.tbsnews.net/coronavirus-chronicle/covid-19-bangladesh/automated-sanitising-chamber-established-chattogram-city	**Automated sanitising chamber established in Chattogram City Corporation**
22/04/2020	https://www.tbsnews.net/coronavirus-chronicle/covid-19-bangladesh/four-jashore-hospitals-ready-provide-covid-19-treatment	**Four Jashore hospitals ready to provide Covid-19 treatment**
22/04/2020	https://www.tbsnews.net/economy/banking/prime-bank-prioritise-banking-pandemic-could-change-transaction-behaviour-72355	**Prime bank to prioritise digital banking as pandemic could change transaction behaviour**
22/04/2020	https://www.tbsnews.net/sports/shakib-puts-world-cup-bat-auction-battle-coronavirus-72259	**Shakib puts World Cup bat on auction to battle coronavirus**
23/04/2020	https://www.tbsnews.net/tech/huawei-extends-device-warranty-avail-best-customer-service-mind-73030	**Huawei extends device warranty to avail best customer service in mind**

Date of the news	News URL	Title of the news
23/04/2020	https://www.tbsnews.net/companies/grameenphone-starts-distribution-ppe-dghs-designated-hospitals-73027	**Grameenphone starts the distribution of PPE to DGHS designated hospitals**
23/04/2020	https://www.tbsnews.net/coronavirus-chronicle/covid-19-bangladesh/general-holidays-extended-may-5-butfactories-reopen-phases	**Factories to open ensuring safety, public transport will resume gradually**
23/04/2020	https://www.tbsnews.net/coronavirus-chronicle/covid-19-bangladesh/marico-fbcci-distributing-food-among-5000-families-gazipur	**Marico, FBCCI distributing food among 5,000 families in Gazipur**
24/04/2020	https://www.tbsnews.net/bangladesh/grameenphone-brac-team-help-one-lakh-poor-families-73366	**Grameenphone, Brac team up to help one lakh poor families**
24/04/2020	https://www.tbsnews.net/tech/adb-aim-launch-global-hackathon-digital-ideas-respond-covid-19-crisis-73315	**ADB, AIM launch global hackathon for digital ideas to respond to Covid-19 crisis**
25/04/2020	https://www.tbsnews.net/companies/akij-stands-beside-covid-19-fighters-73783	**Akij stands beside Covid-19 fighters**
25/04/2020	https://www.tbsnews.net/economy/rmg/do-not-ask-workers-return-dhaka-bgmea-advises-its-members-73447	**BGMEA advises not to ask workers to return to Dhaka**
26/04/2020	https://www.tbsnews.net/coronavirus-chronicle/covid-19-bangladesh/kit-wont-be-handed-over-third-party-zafrullah-74146	**Kit won't be handed over to third party: Zafrullah**
26/04/2020	https://www.tbsnews.net/economy/banking/prime-bank-simplifies-internet-banking-registration-promote-digital-banking-74152	**Prime Bank simplifies Internet Banking registration to promote digital banking**
26/04/2020	https://www.tbsnews.net/bangladesh/health/covid-19-gazi-group-donates-ppes-6-hospitals-dhaka-74047	**Covid-19: Gazi Group donates PPEs to 6 hospitals in Dhaka**
26/04/2020	https://www.tbsnews.net/bangladesh/yfb-arranges-iftar-500-people-74035	**YFB arranges iftar for 500 people**
26/04/2020	https://www.tbsnews.net/bangladesh/health/praava-health-launches-covid-19-self-assessment-tool-chatbot-and-web-page-74002	**Praava Health launches self-assessment tool, webpage to bust Covid-19 myths**
27/04/2020	https://www.tbsnews.net/coronavirus-chronicle/covid-19-bangladesh/grameen-shakti-provides-food-aid-300-underprivileged	**Grameen Shakti provides food aid to 300 underprivileged families**
27/04/2020	https://www.tbsnews.net/economy/stock/icab-issues-covid-19-guidelines-accountants-74182	**ICAB issues COVID-19 guidelines for accountants**
28/04/2020	https://www.tbsnews.net/coronavirus-chronicle/covid-19-bangladesh/pksf-affiliates-donate-tk27-crore-poor-74887	**PKSF affiliates donate Tk27 crore for the poor**
28/04/2020	https://www.tbsnews.net/coronavirus-chronicle/covid-19-bangladesh/obhai-brings-medical-emergency-transport-service-74845	**OBHAI brings medical emergency transport service**
28/04/2020	https://www.tbsnews.net/coronavirus-chronicle/covid-19-bangladesh/rmg-owners-help-workers-if-get-infected-coronavirus-home	**RMG owners to help workers if get infected with coronavirus: Home minister**
28/04/2020	https://www.tbsnews.net/coronavirus-chronicle/covid-19-bangladesh/mtb-employees-donate-one-day-salary-help-poor-74653	**MTB employees donate one-day salary to help poor**
29/04/2020	https://www.tbsnews.net/coronavirus-chronicle/covid-19-bangladesh/covid-19-pepsico-brac-provide-14-million-meals-underserved	**Covid-19: PepsiCo, BRAC to provide 1.4 million meals to underserved families**
29/04/2020	https://www.tbsnews.net/coronavirus-chronicle/covid-19-bangladesh/sylhet-mp-lauded-serving-constituency-amid-covid-19	**Sylhet MP lauded for serving constituency amid Covid-19 outbreak**
29/04/2020	https://www.tbsnews.net/economy/trade/walton-opens-e-sales-amid-lockdown-75403	**Walton opens e-sales amid lockdown**

Date of the news	News URL	Title of the news
29/04/2020	https://www.tbsnews.net/coronavirus-chronicle/covid-19-bangladesh/first-private-covid-19-testing-lab-launched-narayanganj	**First private Covid-19 testing lab launched in Narayanganj**
29/04/2020	https://www.tbsnews.net/coronavirus-chronicle/covid-19-bangladesh/lafargeholcim-distributes-food-packets-and-masks-amid	**LafargeHolcim distributes food packets and masks amid Covid-19**
29/04/2020	https://www.tbsnews.net/coronavirus-chronicle/covid-19-bangladesh/man-who-put-his-life-danger-save-others-75124	**The man who put his life in danger to save others**
30/04/2020	https://www.tbsnews.net/bangladesh/eu-contributes-tk230cr-strengthen-social-security-bangladesh-75820	**EU contributes Tk230cr to strengthen social security in Bangladesh**
30/04/2020	https://www.tbsnews.net/coronavirus-chronicle/covid-19-bangladesh/amar-daktar-provide-health-services-during-pandemic-75823	**Amar Daktar to provide health services during pandemic**
30/04/2020	https://www.tbsnews.net/coronavirus-chronicle/covid-19-bangladesh/six-leading-agencies-join-forces-tackle-covid-19	**AHP along with 6 leading agencies join forces to tackle Covid-19 in Bangladesh**
30/04/2020	https://www.tbsnews.net/coronavirus-chronicle/covid-19-bangladesh/gonoshasthayas-covid-19-testing-kit-permitted-trial-75628	**Gonoshasthaya's Covid-19 testing kit permitted for trial**
30/04/2020	https://www.tbsnews.net/coronavirus-chronicle/covid-19-bangladesh/tofail-ahmed-gives-relief-10000-families-private-fund	**Tofail Ahmed gives relief to 10,000 families from private fund**
30/04/2020	https://www.tbsnews.net/coronavirus-chronicle/covid-19-bangladesh/bgmea-forms-committees-inspect-factories-amid-covid-19	**BGMEA forms committees to inspect factories amid Covid-19**
30/04/2020	https://www.tbsnews.net/coronavirus-chronicle/covid-19-bangladesh/nrbc-bank-provides-ppe-among-front-liners-4-places-75529	**NRBC bank provides PPE among front liners in 4 places**
01/05/2020	https://www.tbsnews.net/coronavirus-chronicle/covid-19-bangladesh/sena-kalyan-sangstha-continues-exemplary-effort-tackle	**Sena Kalyan Sangstha continues exemplary effort to tackle Covid-19 crisis**
01/05/2020	https://www.tbsnews.net/coronavirus-chronicle/covid-19-bangladesh/ideb-cell-makes-nirapad-nagarik-chamber-disinfection	**IDEB cell makes 'Nirapad Nagarik Chamber' disinfection tunnel**
01/05/2020	https://www.tbsnews.net/coronavirus-chronicle/covid-19-bangladesh/two-friends-initiative-help-people-affected-coronavirus	**Two friends' initiative to help people affected by coronavirus**
01/05/2020	https://www.tbsnews.net/coronavirus-chronicle/covid-19-bangladesh/7-more-rmg-workers-infected-coronavirus-savar-76078	**7 more RMG workers infected with coronavirus in Savar**
01/05/2020	https://www.tbsnews.net/coronavirus-chronicle/covid-19-bangladesh/esdo-calls-protecting-health-safety-frontline-workers	**ESDO calls for protecting health, safety of frontline workers**
01/05/2020	https://www.tbsnews.net/coronavirus-chronicle/covid-19-bangladesh/mothers-battle-get-her-children-tested-coronavirus-75901	**A mother's battle to get her children tested for coronavirus**
02/05/2020	https://www.tbsnews.net/coronavirus-chronicle/covid-19-bangladesh/9-more-rmg-workers-tested-positive-covid-19-76441	**9 More RMG workers tested positive for Covid-19**
02/05/2020	https://www.tbsnews.net/economy/banking/bangladesh-bank-creates-money-worth-over-tk70000cr-revive-economy-76435	**Bangladesh Bank creates money worth over Tk70,000cr to revive economy**

Date of the news	News URL	Title of the news
02/05/2020	https://www.tbsnews.net/economy/welfare-oriented-budget-cards-ease-covid-19-fallout-76432	**Welfare-oriented budget on cards to ease Covid-19 fallout**
02/05/2020	https://www.tbsnews.net/coronavirus-chronicle/covid-19-bangladesh/two-garment-workers-found-positive-covid-19-khulna-76411	**Two garment workers found positive for Covid-19 in Khulna**
02/05/2020	https://www.tbsnews.net/coronavirus-chronicle/covid-19-bangladesh/rajshahi-bscic-making-1200-litre-hand-sanitizer-daily	**Rajshahi BSCIC making 1,200 litre hand sanitizer daily**
02/05/2020	https://www.tbsnews.net/coronavirus-chronicle/covid-19-bangladesh/dmch-burn-unit-starts-admitting-covid-19-patients-76384	**DMCH burn unit starts admitting Covid-19 patients**
02/05/2020	https://www.tbsnews.net/economy/trade/bsti-issuing-quicker-clearance-imported-goods-76381	**BSTI issuing quicker clearance for imported goods**
02/05/2020	https://www.tbsnews.net/coronavirus-chronicle/covid-19-bangladesh/bsmmu-committee-formed-evaluate-gonoshasthayas-kit-76363	**BSMMU committee formed to evaluate Gonoshasthaya's kit**
02/05/2020	https://www.tbsnews.net/coronavirus-chronicle/covid-19-bangladesh/bangladesh-extends-suspension-visa-arrival-till-may-7	**Bangladesh extends suspension of visa-on-arrival till May 7**
02/05/2020	https://www.tbsnews.net/coronavirus-chronicle/wateraid-develops-easy-use-hand-washing-station-manual-fight-covid-19-76312	**WaterAid develops 'easy-to-use' hand washing station manual to fight Covid-19**
02/05/2020	https://www.tbsnews.net/coronavirus-chronicle/covid-19-bangladesh/cf-agents-bangladesh-and-india-hold-meeting-start-business	**C&F agents of Bangladesh and India hold meeting to start business**
02/05/2020	https://www.tbsnews.net/economy/banking/brac-bank-enhances-services-support-community-during-pandemic-76402	**Brac Bank enhances services to support community during pandemic**
03/05/2020	https://www.tbsnews.net/coronavirus-chronicle/covid-19-bangladesh/entertainment-industry-anxious-about-massive-losses-eid	**Entertainment industry anxious about massive losses this Eid season**
03/05/2020	https://www.tbsnews.net/coronavirus-chronicle/covid-19-bangladesh/grameen-telecom-provide-1-million-non-medical-face-masks	**Grameen Telecom to provide 1 million non-medical face masks for free**
03/05/2020	https://www.tbsnews.net/economy/debt-relief-tk27000-crore-bangladesh-76795	**Debt relief of Tk2,700 crore for Bangladesh**
03/05/2020	https://www.tbsnews.net/coronavirus-chronicle/covid-19-bangladesh/committees-formed-face-covid-19-get-next-zero-attention	**Committees formed to face Covid-19 get next to zero attention**
03/05/2020	https://www.tbsnews.net/companies/runner-announces-full-salary-no-job-cut-76789	**Runner announces full salary, no job cut**
03/05/2020	https://www.tbsnews.net/coronavirus-chronicle/covid-19-bangladesh/nrbc-bank-provides-protective-equipment-journalists-76714	**NRBC Bank provides protective equipment to journalists**
03/05/2020	https://www.tbsnews.net/companies/uninterrupted-mobile-services-ensured-mobile-recharge-during-lockdown-through-bkash-76690	**Uninterrupted mobile services ensured mobile recharge during lockdown through bKash**
03/05/2020	https://www.tbsnews.net/coronavirus-chronicle/covid-19-bangladesh/bidyanondo-suhana-anis-ahmed-foundation-team-feed-poor	**Bidyanondo, Suhana & Anis Ahmed Foundation team up to feed the poor**
03/05/2020	https://www.tbsnews.net/bangladesh/health/unilever-bangladesh-supports-icddrbs-ongoing-covid-19-emergency-response-76645	**Unilever Bangladesh supports icddr,b's ongoing Covid-19 emergency response**

Date of the news	News URL	Title of the news
04/05/2020	https://www.tbsnews.net/coronavirus-chronicle/covid-19-bangladesh/standard-bank-employees-provide-food-30000-families-77200	**Standard Bank employees provide food for 30,000 families**
04/05/2020	https://www.tbsnews.net/coronavirus-chronicle/covid-19-bangladesh/covid-19-rehab-starts-providing-meals-its-workers-77158	**Covid-19: REHAB starts providing meals to its workers**
04/05/2020	https://www.tbsnews.net/bangladesh/bpmca-withdraws-decision-cut-salaries-and-bonuses-over-doctors-protest-77086	**BPMCA withdraws decision to cut salaries and bonuses over doctors' protest**
04/05/2020	https://www.tbsnews.net/coronavirus-chronicle/covid-19-bangladesh/share-your-fare-initiative-aiming-support-marginalised	**'Share Your Fare': An initiative aiming to support marginalised Rickshaw Pullers**
04/05/2020	https://www.tbsnews.net/coronavirus-chronicle/covid-19-bangladesh/restrictions-ease-covid-19-infections-spike-77251	**Restrictions ease as Covid-19 infections spike**
05/05/2020	https://www.tbsnews.net/coronavirus-chronicle/covid-19-bangladesh/90-health-workers-including-15-doctors-test-positive-covid	**90 health workers including 15 doctors test positive for Covid-19 in Narayanganj**
05/05/2020	https://www.tbsnews.net/economy/banking/banks-will-not-pay-more-double-salary-attending-office-77617	**Banks will not pay more than double salary for attending office**
06/05/2020	https://www.tbsnews.net/economy/here-comes-pay-chop-78145	**Here comes the pay chop**
06/05/2020	https://www.tbsnews.net/economy/health-ministry-formulates-technical-guidelines-resume-economic-activities-78115	**Shoppers have to go through thermal scanner**
06/05/2020	https://www.tbsnews.net/economy/covid-19-cost-bangladesh-tourism-sector-tk40bn-unwto-78118	**Covid-19 to cost Bangladesh tourism sector Tk40bn: UNWTO**
06/05/2020	https://www.tbsnews.net/coronavirus-chronicle/covid-19-bangladesh/govt-waives-vat-ppe-surgical-mask-78067	**Govt waives VAT on PPE, surgical mask**
06/05/2020	https://www.tbsnews.net/coronavirus-chronicle/covid-19-bangladesh/spike-covid-19-cases-after-garments-factories-reopen	**Spike in Covid-19 cases after garments factories reopen in Narayanganj**
07/05/2020	https://www.tbsnews.net/coronavirus-chronicle/covid-19-bangladesh/ksrm-provides-ppe-satkania-police-78649	**KSRM provides PPE to Satkania police**
07/05/2020	https://www.tbsnews.net/coronavirus-chronicle/covid-19-bangladesh/us-govt-provides-over-22-million-fight-covid-19-bangladesh	**US Govt provides over $22 million to fight Covid-19 in Bangladesh**
07/05/2020	https://www.tbsnews.net/coronavirus-chronicle/covid-19-bangladesh/evaly-donates-personal-health-safety-equipment-cttc-78391	**Evaly donates personal health safety equipment for CTTC**
08/05/2020	https://www.tbsnews.net/coronavirus-chronicle/covid-19-bangladesh/covid-19-medicine-bangladesh-only-two-weeks-away-78976	**Covid-19 medicine in Bangladesh only two weeks away**
08/05/2020	https://www.tbsnews.net/coronavirus-chronicle/covid-19-bangladesh/gonoshasthaya-kit-performance-trial-likely-begin-sunday	**Gonoshasthaya kit performance trial likely to begin Sunday**
08/05/2020	https://www.tbsnews.net/economy/rmg/10-rmg-workers-contract-covid-19-gazipur-78940	**10 RMG workers contract Covid-19 in Gazipur**
08/05/2020	https://www.tbsnews.net/coronavirus-chronicle/covid-19-bangladesh/coronavirus-sylhet-shopping-malls-markets-will-be-shut	**Coronavirus: Sylhet shopping malls, markets will be shut until Eid**
08/05/2020	https://www.tbsnews.net/coronavirus-chronicle/covid-19-bangladesh/impulse-hospital-treat-covid-19-positive-policemen-78841	**Impulse Hospital to treat Covid-19 positive policemen**

Date of the news	News URL	Title of the news
08/05/2020	https://www.tbsnews.net/coronavirus-chronicle/covid-19-bangladesh/they-distribute-food-needy-amid-pandemic-78754	**They distribute food to the needy amid the pandemic**
08/05/2020	https://www.tbsnews.net/economy/5-star-hotels-seeking-alternative-opportunities-recover-losses-78748	**5-star hotels seeking alternative opportunities to recover losses**
09/05/2020	https://www.tbsnews.net/economy/rmg/bgmea-requests-members-not-terminate-workers-may-79378	**BGMEA requests members not to terminate workers in May**
09/05/2020	https://www.tbsnews.net/markets/coxs-bazar-ctg-and-mymensingh-markets-not-reopen-eid-79297	**Cox's Bazar, Rajshahi, Ctg and Mymensingh markets not to reopen before Eid**
09/05/2020	https://www.tbsnews.net/coronavirus-chronicle/covid-19-bangladesh/sajida-foundations-contribution-health-finance-and	**Sajida foundation's contribution to health, finance and awareness amid Covid-19 crisis**
10/05/2020	https://www.tbsnews.net/coronavirus-chronicle/covid-19-bangladesh/max-group-donates-tk3-crore-pms-relief-and-welfare-fund	**MAX Group donates Tk3 crore to PM's relief and welfare fund**
10/05/2020	https://www.tbsnews.net/coronavirus-chronicle/covid-19-bangladesh/covid-19-cse-donates-tk25-lakh-pms-relief-fund-79729	**Covid-19: CSE donates Tk25 lakh to PM's relief fund**
10/05/2020	https://www.tbsnews.net/coronavirus-chronicle/covid-19-bangladesh/bashundhara-group-donates-safety-equipment-bgb-79699	**Bashundhara Group donates safety equipment to BGB**
10/05/2020	https://www.tbsnews.net/coronavirus-chronicle/covid-19-bangladesh/cartoon-people-and-a2i-organises-cartoon-contest-raise	**Cartoon People and a2i organises cartoon contest to raise coronavirus awareness**
10/05/2020	https://www.tbsnews.net/coronavirus-chronicle/covid-19-bangladesh/holy-family-hospital-dedicated-covid-19-patients-79672	**Holy Family hospital dedicated for Covid-19 patients**
10/05/2020	https://www.tbsnews.net/coronavirus-chronicle/covid-19-bangladesh/nestle-donates-covid-19-relief-pms-fund-79537	**Nestle donates Covid-19 relief in PM's fund**
10/05/2020	https://www.tbsnews.net/coronavirus-chronicle/covid-19-bangladesh/bgmea-launches-covid-19-test-centre-rmg-workers-79444	**BGMEA launches Covid-19 test centre for RMG workers**
11/05/2020	https://www.tbsnews.net/economy/aviation/flight-social-distancing-worsen-airline-woe-80224	**In-flight social distancing to worsen airline woe**
11/05/2020	https://www.tbsnews.net/bangladesh/health/private-hospitals-lose-licence-refusing-covid-19-patients-80218	**Private hospitals to lose licence for refusing emergency patients or anybody**
11/05/2020	https://www.tbsnews.net/bangladesh/health/covid-19-sample-collection-booth-opens-dhaka-reporters-unity-80194	**Covid-19 sample collection booth opens at Dhaka Reporters Unity**
11/05/2020	https://www.tbsnews.net/coronavirus-chronicle/covid-19-bangladesh/narayanganj-people-shopping-spree-amid-spike-covid-19	**Narayanganj people on shopping spree amid spike in Covid-19 cases**
11/05/2020	https://www.tbsnews.net/bangladesh/education/uiu-cblu-conducts-online-fdp-80101	**UIU, CBLU conducts Online FDP**
11/05/2020	https://www.tbsnews.net/coronavirus-chronicle/covid-19-bangladesh/unilever-red-crescent-join-hands-combat-covid-19-across	**Unilever, Red Crescent join hands to combat Covid-19 across country**
11/05/2020	https://www.tbsnews.net/coronavirus-chronicle/covid-19-bangladesh/gonoshasthaya-seeks-temporary-approval-its-covid-19-test	**Gonoshasthaya seeks interim approval for Covid-19 testing kits**

Date of the news	News URL	Title of the news
11/05/2020	https://www.tbsnews.net/coronavirus-chronicle/covid-19-bangladesh/state-minister-foreign-affairs-donates-ventilators-n95	**State minister for foreign affairs donates ventilators, N95 masks to police hospital**
12/05/2020	https://www.tbsnews.net/coronavirus-chronicle/covid-19-bangladesh/pran-rfl-donates-covid-19-sample-collection-booths-4	**Pran-Rfl donates Covid-19 sample collection booths to 4 hospitals**
12/05/2020	https://www.tbsnews.net/coronavirus-chronicle/covid-19-bangladesh/covid-19-hospitals-still-running-low-oxygen-80659	**Covid-19 hospitals still running low on oxygen**
12/05/2020	https://www.tbsnews.net/coronavirus-chronicle/covid-19-bangladesh/gonoshasthaya-set-submit-its-kit-bsmmu-wednesday-80647	**Gonoshasthaya set to submit its kit to BSMMU on Wednesday**
12/05/2020	https://www.tbsnews.net/coronavirus-chronicle/covid-19-bangladesh/brac-assisting-dghs-install-50-kiosks-covid-19-sample	**BRAC teams up with DGHS to install Covid-19 test kiosks in Dhaka**
12/05/2020	https://www.tbsnews.net/coronavirus-chronicle/covid-19-bangladesh/foodpanda-introduces-insurance-its-riders-80563	**Foodpanda introduces insurance for its riders**
12/05/2020	https://www.tbsnews.net/coronavirus-chronicle/covid-19-bangladesh/abul-khair-group-provides-oxygen-covid-19-treatment-80479	**Abul Khair Group provides oxygen for Covid-19 treatment**
12/05/2020	https://www.tbsnews.net/feature/panorama/covid-19-impact-low-income-communities-stare-grim-reality-80410	**Covid-19 impact: Low-income communities stare at grim reality**
13/05/2020	https://www.tbsnews.net/economy/banking/brac-bank-waives-penal-fees-dps-81145	**Brac Bank waives penal fees on DPS**
13/05/2020	https://www.tbsnews.net/coronavirus-chronicle/covid-19-bangladesh/bsrm-carries-out-csr-initiatives-amid-covid-19-crisis	**BSRM carries out CSR initiatives amid Covid-19 crisis**
13/05/2020	https://www.tbsnews.net/bangladesh/health/shohoz-now-comes-digital-healthcare-solution-81136	**Shohoz now comes up with digital healthcare solution**
13/05/2020	https://www.tbsnews.net/coronavirus-chronicle/covid-19-bangladesh/two-narayanganj-markets-shut-they-failed-follow-health	**Two Narayanganj markets shut as they failed to follow health rules**
13/05/2020	https://www.tbsnews.net/coronavirus-chronicle/covid-19-bangladesh/markets-shopping-malls-shut-down-tangail-defying-health	**Markets, shopping malls shut down in Tangail for defying health guidelines**
13/05/2020	https://www.tbsnews.net/companies/walton-makes-medicart-robot-covid-19-patients-81115	**Walton makes Medicart robot for Covid-19 patients**
13/05/2020	https://www.tbsnews.net/coronavirus-chronicle/covid-19-bangladesh/grameen-telecom-provides-masks-and-ppes-police-army-and	**Grameen Telecom provides masks and PPEs to the Police, Army and RAB**
13/05/2020	https://www.tbsnews.net/coronavirus-chronicle/covid-19-bangladesh/singer-provides-home-appliances-bangladesh-police	**Singer provides home appliances for Bangladesh Police Hospitals**
13/05/2020	https://www.tbsnews.net/coronavirus-chronicle/covid-19-bangladesh/bsrm-group-supports-helpless-amid-covid-19-80974	**BSRM group supports helpless amid Covid-19**
13/05/2020	https://www.tbsnews.net/coronavirus-chronicle/covid-19-bangladesh/gonoshasthaya-submits-200-covid-19-testing-kits-bsmmu	**Gonoshasthaya Kendra hands over Covid-19 test kits to BSMMU**
13/05/2020	https://www.tbsnews.net/companies/metlife-bangladesh-launches-covid-19-support-programme-customers-80899	**MetLife Bangladesh launches Covid-19 support programme for customers**
14/05/2020	https://www.tbsnews.net/coronavirus-chronicle/covid-19-bangladesh/zaber-and-zubair-develops-coronavirus-killing-fabric-81532	**Zaber and Zubair develops ‘coronavirus killing fabric’**

Date of the news	News URL	Title of the news
14/05/2020	https://www.tbsnews.net/coronavirus-chronicle/covid-19-bangladesh/old-faujians-lends-hand-covid-19-victims-81478	**Old Faujians lends a hand to Covid-19 victims**
14/05/2020	https://www.tbsnews.net/coronavirus-chronicle/covid-19-bangladesh/swedish-clothing-company-hm-donates-8-ventilators-and-1500	**Swedish clothing company H&M donates 8 ventilators and 1500 PPE to Bangladesh**
14/05/2020	https://www.tbsnews.net/coronavirus-chronicle/covid-19-bangladesh/robi-brings-33gb-data-free-doctors-6-months-81277	**Robi brings 33GB data for free to doctors for 6 months**
14/05/2020	https://www.tbsnews.net/coronavirus-chronicle/covid-19-bangladesh/unicef-welcomes-release-children-detention-81349	**Unicef welcomes release of children from detention**
15/05/2020	https://www.tbsnews.net/coronavirus-chronicle/covid-19-bangladesh/field-hospital-brings-relief-ctg-covid-19-patients-81766	**Field hospital brings relief to Ctg Covid-19 patients**
16/05/2020	https://www.tbsnews.net/coronavirus-chronicle/covid-19-bangladesh/grameen-telecom-provides-healthcare-equipment-hospitals	**Grameen Telecom provides healthcare equipment to hospitals**
16/05/2020	https://www.tbsnews.net/coronavirus-chronicle/covid-19-bangladesh/health-minister-inaugurates-dedicated-covid-19-hospital	**Health minister inaugurates dedicated Covid-19 hospital building**
16/05/2020	https://www.tbsnews.net/coronavirus-chronicle/covid-19-bangladesh/jatri-gives-dhaka-chaka-green-dhaka-staff-eid-gifts-82126	**Jatri gives Dhaka Chaka, Green Dhaka staff Eid gifts**
16/05/2020	https://www.tbsnews.net/economy/banking/beneficiaries-getting-pms-cash-aid-easily-through-bkash-amid-pandemic-81916	**Beneficiaries getting PM's cash aid easily through bKash amid pandemic**
17/05/2020	https://www.tbsnews.net/companies/shwapno-recruits-300-staff-amid-covid-19-pandemic-82468	**Shwapno recruits 300 staff amid Covid-19 pandemic**
17/05/2020	https://www.tbsnews.net/coronavirus-chronicle/covid-19-bangladesh/praava-health-starts-testing-covid-19-82372	**Praava Health starts testing for Covid-19**
18/05/2020	https://www.tbsnews.net/economy/banking/standard-chartered-encourages-clients-join-fight-against-coronavirus-82882	**Standard Chartered encourages clients to join fight against coronavirus**
18/05/2020	https://www.tbsnews.net/coronavirus-chronicle/covid-19-bangladesh/bkash-provides-food-5000-virus-hit-families-82870	**bKash provides food for 5,000 virus-hit families**
18/05/2020	https://www.tbsnews.net/sports/mashrafes-bracelet-bought-whopping-42-lakhs-then-gifted-back-him-82510	**Mashrafe's bracelet bought for whopping 42 lakhs, then gifted back to him**
19/05/2020	https://www.tbsnews.net/bangladesh/hsbc-launches-joy-giving-bring-smiles-100000-faces-83344	**HSBC launches 'Joy of Giving' to bring smiles to 100,000 faces**
19/05/2020	https://www.tbsnews.net/coronavirus-chronicle/covid-19-bangladesh/dhpb-scouts-brac-work-jointly-fight-covid-19-83329	**DHPB, Scouts, Brac to work jointly to fight Covid-19**
19/05/2020	https://www.tbsnews.net/coronavirus-chronicle/covid-19-bangladesh/ficci-donates-11000-ppe-dghs-83317	**FICCI donates 11,000 PPE to DGHS**
19/05/2020	https://www.tbsnews.net/economy/fbcci-help-smes-availing-special-loans-stimulus-package-83293	**FBCCI to help SMEs availing special loans from stimulus package**
19/05/2020	https://www.tbsnews.net/coronavirus-chronicle/covid-19-bangladesh/jti-continues-supporting-pandemic-hit-people-83272	**JTI continues supporting pandemic-hit people**
19/05/2020	https://www.tbsnews.net/economy/resurgent-bangladesh-launched-help-private-sector-recover-83245	**'Resurgent Bangladesh' launched to help private sector recover**

Date of the news	News URL	Title of the news
20/05/2020	https://www.tbsnews.net/coronavirus-chronicle/covid-19-bangladesh/priyoshop-one-stop-shopping-point-fight-covid-19-83818	**PriyoShop, a one-stop-shopping point, to fight Covid-19**
20/05/2020	https://www.tbsnews.net/panorama/feeding-people-hiding-shadows-83428	**Feeding the people hiding in the shadows**
21/05/2020	https://www.tbsnews.net/coronavirus-chronicle/covid-19-bangladesh/beximco-pharma-introduces-worlds-first-generic-remdesivir	**Beximco Pharma introduces world's first generic remdesivir for Covid-19 treatment**
21/05/2020	https://www.tbsnews.net/bangladesh/health/ctg-private-hospital-employees-hold-protests-arrears-eid-bonus-84220	**Ctg private hospital employees hold protests for arrears, Eid bonus**
21/05/2020	https://www.tbsnews.net/coronavirus-chronicle/covid-19-bangladesh/coca-cola-and-care-bangladesh-support-over-50000-people	**Coca-Cola and CARE Bangladesh support over 50,000 people with a month's food ration**
22/05/2020	https://www.tbsnews.net/economy/rmg/bangladesh-threatens-blacklist-non-paying-british-brands-84559	**Bangladesh threatens to blacklist non-paying British brands**
23/05/2020	https://www.tbsnews.net/coronavirus-chronicle/covid-19-bangladesh/covid-19-test-gonoshasthaya-kit-begins-may-26-84904	**Covid-19 test with Gonoshasthaya kit begins May 26**
23/05/2020	https://www.tbsnews.net/bangladesh/radisson-blu-distributes-iftar-box-among-healthcare-officials-kurmitola-hospital-84766	**Radisson Blu distributes iftar box among healthcare officials at Kurmitola Hospital**
23/05/2020	https://www.tbsnews.net/coronavirus-chronicle/covid-19-bangladesh/evaly-donates-health-care-products-999-84649	**Evaly donates health care products for 999**
24/05/2020	https://www.tbsnews.net/coronavirus-chronicle/covid-19-bangladesh/navana-donates-medical-equipment-covid-19-frontline	**Navana donates medical equipment to Covid-19 frontline workers**
24/05/2020	https://www.tbsnews.net/bangladesh/health/eskayef-starts-distributing-remdesivir-hospitals-coronavirus-treatment-85132	**Eskayef starts distributing Remdesivir to hospitals for coronavirus treatment**
24/05/2020	https://www.tbsnews.net/coronavirus-chronicle/covid-19-bangladesh/cultural-activists-sylhet-providing-food-love-people-85048	**Cultural activists of Sylhet providing 'food of love' to people**
25/05/2020	https://www.tbsnews.net/companies/novoair-celebrates-eid-ul-fitr-distressed-people-85336	**Novoair celebrates Eid- Ul-Fitr with distressed people**
25/05/2020	https://www.tbsnews.net/coronavirus-chronicle/covid-19-bangladesh/gonoshasthaya-postpones-covid-19-test-its-kit-85384	**Gonoshasthaya postpones Covid-19 test with its kit on drug admin's order**
25/05/2020	https://www.tbsnews.net/bangladesh/manobotar-eid-utshob-85360	**'Manobotar Eid Utshob'**
26/05/2020	https://www.tbsnews.net/bangladesh/health/all-50-bed-hospitals-must-provide-treatment-covid-non-covid-patients-85636	**All 50-bed hospitals must provide treatment to Covid, non-Covid patients**
26/05/2020	https://www.tbsnews.net/coronavirus-chronicle/covid-19-bangladesh/2-chattogram-hospitals-dedicated-treat-covid-19-patients	**2 Chattogram hospitals dedicated to treat Covid-19 patients**
27/05/2020	https://www.tbsnews.net/economy/banking/prab-makes-coronavirus-awareness-video-alo-asbei-85897	**PRAB makes coronavirus awareness video "Alo asbei"**
28/05/2020	https://www.tbsnews.net/coronavirus-chronicle/covid-19-bangladesh/novartis-donates-safety-gears-combat-coronavirus-86251	**Novartis donates safety gears to combat coronavirus**

Date of the news	News URL	Title of the news
28/05/2020	https://www.tbsnews.net/coronavirus-chronicle/covid-19-bangladesh/citi-foundation-donate-tk85-lakh-materialise-relief	Citi Foundation to donate Tk85 lakh to materialise relief programmes
28/05/2020	https://www.tbsnews.net/coronavirus-chronicle/covid-19-bangladesh/us-embassy-provides-covid-19-response-equipment-kamalapur	US Embassy provides Covid-19 response equipment to Kamalapur Railway General Hospital
30/05/2020	https://www.tbsnews.net/coronavirus-chronicle/covid-19-bangladesh/bangladeshi-scientists-complete-genome-sequencing-novel	Bangladeshi scientists complete genome sequencing of novel coronavirus
30/05/2020	https://www.tbsnews.net/bangladesh/road-transport/combating-covid-19-uber-forms-transport-safety-alliance-86794	Combating Covid-19: Uber forms transport safety alliance
30/05/2020	https://www.tbsnews.net/coronavirus-chronicle/covid-19-bangladesh/health-ministry-issues-revised-health-guidelines	Health ministry issues revised health guidelines for workplaces
30/05/2020	https://www.tbsnews.net/bangladesh/education/brac-university-establishes-student-assistance-fund-86710	Brac University establishes student assistance fund
30/05/2020	https://www.tbsnews.net/economy/adb-provides-grant-bangladesh-boosting-urban-primary-healthcare-facilities-86692	ADB provides grant to boost urban primary healthcare facilities
01/06/2020	https://www.tbsnews.net/thoughts/small-businesses-need-take-careful-steps-order-survive-pandemic-87271	Small businesses need to take careful steps in order to survive the pandemic
01/06/2020	https://www.tbsnews.net/feature/panorama/hard-times-pandemic-hit-landless-poor-87511	Hard times for the pandemic-hit landless poor
01/06/2020	https://www.tbsnews.net/coronavirus-chronicle/covid-19-bangladesh/star-rated-coxs-bazar-hotel-turn-isolation-centre-87517	Star-rated Cox's Bazar hotel to turn into isolation centre
01/06/2020	https://www.tbsnews.net/economy/reopening-restaurants-still-uncertain-87553	Reopening of restaurants still uncertain
02/06/2020	https://www.tbsnews.net/coronavirus-chronicle/covid-19-bangladesh/maintain-health-directives-social-distancing-while	Maintain health directives, social distancing while operating public transport: Quader
02/06/2020	https://www.tbsnews.net/coronavirus-chronicle/covid-19-bangladesh/abb-starts-collecting-data-covid-19-affected-bankers-87835	ABB starts collecting data on Covid-19-affected bankers
02/06/2020	https://www.tbsnews.net/companies/prime-bank-prioritise-health-safety-post-shutdown-operations-87964	Prime Bank to prioritise health safety in post shutdown operations
02/06/2020	https://www.tbsnews.net/coronavirus-chronicle/covid-19-bangladesh/s-alam-group-donates-medical-equipment-3-ctg-hospitals	S Alam Group donates medical equipment to 3 Ctg hospitals
03/06/2020	https://www.tbsnews.net/coronavirus-chronicle/covid-19-bangladesh/stanchart-now-lends-hand-covid-19-patients-88378	StanChart now lends a hand to Covid-19 patients
03/06/2020	https://www.tbsnews.net/coronavirus-chronicle/covid-19-bangladesh/one-million-non-medical-masks-distributed-grameen-telecom	One million non-medical masks distributed by Grameen Telecom

Date of the news	News URL	Title of the news
04/06/2020	https://www.tbsnews.net/coronavirus-chronicle/covid-19-bangladesh/govt-prepares-hospital-treat-covid-19-positive-officials	**Govt prepares hospital to treat Covid-19 positive officials**
04/06/2020	https://www.tbsnews.net/coronavirus-chronicle/covid-19-bangladesh/bgmeas-first-covid-19-test-lab-launched-88900	**BGMEA's first Covid-19 testing lab launched**
04/06/2020	https://www.tbsnews.net/coronavirus-chronicle/covid-19-bangladesh/doctors-associations-launch-telemedicine-service-covid-19	**Doctors' associations launch telemedicine service for Covid-19 patients**
05/06/2020	https://www.tbsnews.net/analysis/public-expenditure-and-deficit-financing-covid-19-era-89239	**Public Expenditure and deficit financing in the Covid-19 era**
06/06/2020	https://www.tbsnews.net/coronavirus-chronicle/covid-19-bangladesh/bb-moves-work-home-following-health-rules-89623	**BB moves to work from home following health rules**
07/06/2020	https://www.tbsnews.net/companies/pharma/beximco-supplies-generic-remdesivir-drugs-nigeria-89971	**Beximco supplies generic Remdesivir drugs to Nigeria**
07/06/2020	https://www.tbsnews.net/coronavirus-chronicle/covid-19-bangladesh/du-tests-coronavirus-samples-40-minutes-using-rt-lamp-kit	**DU tests coronavirus samples in 30 minutes using RT-LAMP kit**
08/06/2020	https://www.tbsnews.net/bangladesh/health/health-ministry-recruit-3000-new-people-including-1200-medical-technologists-90499	**Health Ministry to recruit 3,000 new people including 1,200 medical technologists**
09/06/2020	https://www.tbsnews.net/bangladesh/health/govt-import-oxygen-cylinders-hfnc-91054	**Govt to import oxygen cylinders, HFNC**
09/06/2020	https://www.tbsnews.net/bangladesh/crime/2-chattogram-traders-fined-tk430-lakh-selling-oxygen-cylinders-high-prices-91021	**2 Chattogram traders fined Tk4.30 lakh for selling oxygen cylinders at high prices**
09/06/2020	https://www.tbsnews.net/economy/industry/govt-approves-health-safety-guidelines-factories-establishments-91015	**Govt approves health safety guidelines for factories, establishments**
10/06/2020	https://www.tbsnews.net/economy/banking/covid-19-sibl-urges-clients-use-mobile-app-safer-banking-91552	**Covid-19: SIBL urges clients to use mobile app for safer banking**
11/06/2020		
12/06/2020	https://www.tbsnews.net/coronavirus-chronicle/covid-19-bangladesh/rohingya-cyclists-share-covid-19-info-door-door-92488	**Rohingya cyclists share Covid-19 info door-to-door**
13/06/2020	https://www.tbsnews.net/bangladesh/districts/deputy-minister-nowfel-donates-tk3-lakh-temporary-hospital-workers-92827	**Deputy Minister Nowfel donates Tk3 lakh to temporary hospital workers**
13/06/2020	https://www.tbsnews.net/coronavirus-chronicle/covid-19-bangladesh/26-hospitals-getting-centralised-oxygen-supply-combat	**26 hospitals getting centralised oxygen supply to combat Covid-19**

Date of the news	News URL	Title of the news
14/06/2020	https://www.tbsnews.net/coronavirus-chronicle/covid-19-bangladesh/just-190-new-ventilators-installed-despite-high-demand	Just 190 new ventilators installed despite high demand
15/06/2020	https://www.tbsnews.net/economy/trade/pandemics-gainer-mask-factories-work-overdrive-93712	Covid-19 unmasks business potential for mask makers
16/06/2020	https://www.tbsnews.net/coronavirus-chronicle/covid-19-bangladesh/saf-bma-jointly-launch-covid-19-sample-collection-booths	Covid-19 sample collection booth for doctors launched in Chattogram
16/06/2020	https://www.tbsnews.net/coronavirus-chronicle/covid-19-bangladesh/chattogram-city-corporation-fires-10-doctors-avoiding-duty	10 doctors fired for avoiding duty at isolation centre in Ctg
17/06/2020	https://www.tbsnews.net/coronavirus-chronicle/covid-19-bangladesh/banks-ordered-ensure-csr-support-covid-19-treatment-94681	Banks ordered to ensure CSR support for Covid-19 treatment
17/06/2020	https://www.tbsnews.net/coronavirus-chronicle/covid-19-bangladesh/icddrb-begins-clinical-trial-ivermectin-treat-patients	Icddr,b begins clinical trial of Ivermectin to treat patients with Covid-19
18/06/2020	https://www.tbsnews.net/coronavirus-chronicle/covid-19-bangladesh/unilever-bangladesh-donates-critical-medical-equipment	Unilever Bangladesh donates critical medical equipment to fight against Covid-19
19/06/2020	https://www.tbsnews.net/coronavirus-chronicle/covid-19-bangladesh/grameen-telecom-donates-ppe-volunteers-ambulance-service	Grameen Telecom donates PPE to volunteers, ambulance service providers
19/06/2020	https://www.tbsnews.net/coronavirus-chronicle/covid-19-bangladesh/tk-group-donates-20-ventilators-chattogram-hospitals-95560	TK Group donates 20 ventilators to Chattogram hospitals
19/06/2020	https://www.tbsnews.net/coronavirus-chronicle/covid-19-bangladesh/covid-19-baf-aircraft-brings-medical-aid-s-korea-95542	Covid-19: BAF aircraft brings medical aid from S Korea
19/06/2020	https://www.tbsnews.net/rohingya-crisis/iom-organises-itc-visit-rohingyas-increase-awareness-covid-19-95467	IOM organises ITC visit for Rohingyas to increase awareness of Covid-19
19/06/2020	https://www.tbsnews.net/companies/startups/ekhoni-youth-initiative-address-urgent-needs-amid-covid-19-crisis-95362	Ekhoni: A youth initiative to address urgent needs amid Covid-19 crisis
20/06/2020	https://www.tbsnews.net/coronavirus-chronicle/covid-19-bangladesh/covid-19-impacts-95-households-income-world-vision-survey	Covid-19 impacts 95% households' income: World Vision survey
21/06/2020	https://www.tbsnews.net/coronavirus-chronicle/covid-19-bangladesh/army-supervise-1000-bed-covid-19-isolation-centre-dncc	Army to supervise 1,000-bed Covid-19 isolation centre at DNCC market
22/06/2020		
23/06/2020	https://www.tbsnews.net/economy/foreign-aid/jica-provide-tk79cr-medical-equipment-bangladesh-97273	Jica to provide Tk79cr medical equipment to Bangladesh
24/06/2020	https://www.tbsnews.net/coronavirus-chronicle/covid-19-bangladesh/govt-forms-coordination-cell-prevent-covid-19-97648	Govt forms coordination cell to prevent Covid-19

Date of the news	News URL	Title of the news
24/06/2020	https://www.tbsnews.net/coronavirus-chronicle/covid-19-bangladesh/icddrb-begin-covid-19-testing-97384	icddr,b to begin Covid-19 testing
25/06/2020	https://www.tbsnews.net/coronavirus-chronicle/covid-19-bangladesh/bgmea-hospital-chattogram-being-converted-covid-19	BGMEA Hospital in Chattogram being converted into Covid-19 isolation centre
26/06/2020		
27/06/2020	https://www.tbsnews.net/coronavirus-chronicle/covid-19-bangladesh/icu-scarcity-acute-yet-who-cares-98902	ICU scarcity acute, yet who cares?
27/06/2020	https://www.tbsnews.net/coronavirus-chronicle/covid-19-bangladesh/70-bed-isolation-centre-opened-chattogram-98878	70-bed isolation centre opened in Chattogram
28/06/2020	https://www.tbsnews.net/economy/food-service-worker-family-starving-99295	Food service worker, family starving
28/06/2020	https://www.tbsnews.net/coronavirus-chronicle/covid-19-bangladesh/clean-cities-initiative-jointly-launched-healthy	The Clean Cities initiative jointly launched by Healthy Bangladesh, PPRC, UNDP
28/06/2020	https://www.tbsnews.net/coronavirus-chronicle/covid-19-bangladesh/iom-opens-new-covid-19-isolation-and-treatment-centers	IOM opens new Covid-19 isolation and treatment centers in Cox's Bazar
29/06/2020	https://www.tbsnews.net/coronavirus-chronicle/covid-19-bangladesh/cmp-bidyanondo-field-hospital-starts-operations-ctg	'CMP-Bidyanondo Field Hospital' starts operations in Ctg Wednesday
30/06/2020	https://www.tbsnews.net/economy/banking/bangladesh-bank-sees-pandemic-cloud-over-banks-100207	Bangladesh Bank sees pandemic cloud over banks
01/07/2020	https://www.tbsnews.net/coronavirus-chronicle/covid-19-bangladesh/bidyanondo-cmp-field-hospital-begins-journey-100684	Bidyanondo-CMP field hospital begins journey
01/07/2020	https://www.tbsnews.net/coronavirus-chronicle/covid-19-bangladesh/ilo-partners-bgmea-bkmea-launch-covid-19-safety-learning	ILO partners with BGMEA, BKMEA to launch Covid-19 safety "Learning Hub" for RMG workers
01/07/2020	https://www.tbsnews.net/coronavirus-chronicle/covid-19-bangladesh/new-building-ctg-port-hospital-inaugurated-treat-covid-19	New building of Ctg Port Hospital inaugurated to treat Covid-19 patients
01/07/2020	https://www.tbsnews.net/archive/2020/07/01	Yunus Centre joins hand with prominent world figures to declare Covid-19 vaccine as global common good
02/07/2020	https://www.tbsnews.net/coronavirus-chronicle/covid-19-bangladesh/bgmea-inaugurates-50-bed-dedicated-covid-19-field-hospital	BGMEA inaugurates 50-bed dedicated Covid-19 field hospital in Chattogram
03/07/2020	https://www.tbsnews.net/economy/rmg/ilo-launches-covid-19-safety-training-rmg-workers-101170	ILO launches Covid-19 safety training for RMG workers

Date of the news	News URL	Title of the news
04/07/2020	https://www.tbsnews.net/coronavirus-chronicle/covid-19-bangladesh/samorita-hospital-opens-covid-19-unit-101788	**Samorita Hospital opens Covid-19 unit**
05/07/2020	https://www.tbsnews.net/coronavirus-chronicle/covid-19-bangladesh/covid-19-units-open-four-khulna-private-hospitals-102208	**Covid-19 units to open in four Khulna private hospitals**
06/07/2020	https://www.tbsnews.net/companies/berger-paints-bangladesh-launches-hand-sanitisers-102649	**Berger Paints Bangladesh launches hand sanitisers**
06/07/2020	https://www.tbsnews.net/coronavirus-chronicle/covid-19-bangladesh/four-khulna-pvt-hospitals-ready-covid-19-units-102598	**Four Khulna pvt hospitals to ready Covid-19 units**
06/07/2020	https://www.tbsnews.net/coronavirus-chronicle/covid-19-bangladesh/barishal-and-khulna-healthcare-professionals-get-ipc-and	**Barishal and Khulna healthcare professionals get IPC and PPE from UK AID and Concern Worldwide**
07/07/2020	https://www.tbsnews.net/bangladesh/health/only-9-public-hospitals-get-budget-allocation-oxygen-103195	**Only 9 public hospitals get budget allocation for oxygen**
08/07/2020	https://www.tbsnews.net/coronavirus-chronicle/covid-19-bangladesh/96-covid-19-patients-recover-beacon-pharmas-favipira-trial	**96% of Covid-19 patients recover in Beacon pharma's Favipira trial**
09/07/2020	https://www.tbsnews.net/coronavirus-chronicle/covid-19-bangladesh/us-embassy-provides-covid-19-response-equipment-0	**US Embassy provides Covid-19 response equipment to Bangladesh Police**
10/07/2020	https://www.tbsnews.net/coronavirus-chronicle/covid-19-bangladesh/website-launched-chattogram-make-covid-19-information	**Website launched in Chattogram to make Covid-19 information easily accessible**
11/07/2020		
12/07/2020	https://www.tbsnews.net/coronavirus-chronicle/covid-19-bangladesh/dghs-revokes-covid-19-testing-permission-5-institutes	**DGHS revokes Covid-19 testing permission of 5 institutes**
12/07/2020	https://www.tbsnews.net/companies/bashundhara-group-stands-families-3-covid-19-victim-journalists-105229	Bashundhara Group stands by families of 3 Covid-19 victim journalists
12/07/2020	https://www.tbsnews.net/coronavirus-chronicle/covid-19-bangladesh/covid-19-negative-certificate-must-bangladeshis-travel	**Covid-19 negative certificates mandatory for air travel from July 23**
13/07/2020	https://www.tbsnews.net/coronavirus-chronicle/covid-19-bangladesh/only-families-dead-frontline-fighters-will-get	**Only families of dead frontline fighters will get compensation for now**
14/07/2020	https://www.tbsnews.net/companies/employees-top-priority-marico-bangladesh-amid-pandemic-106300	**Employees top priority to Marico Bangladesh amid pandemic**
14/07/2020	https://www.tbsnews.net/coronavirus-chronicle/covid-19-bangladesh/ruet-students-develop-emergency-ventilators-covid-19	**RUET students develop emergency ventilators for Covid-19 patients**
15/07/2020		

Date of the news	News URL	Title of the news
16/07/2020	https://www.tbsnews.net/coronavirus-chronicle/covid-19-bangladesh/coxs-bazar-dc-hands-over-financial-aid-433-small-tourism	**Cox's Bazar DC hands over financial aid to 433 small tourism-related businesses**
16/07/2020	https://www.tbsnews.net/coronavirus-chronicle/covid-19-bangladesh/covid-19-decreased-income-has-affected-livelihood-983-poor	**Covid-19: Decreased income has affected livelihood of 98.3% of the poor**
16/07/2020	https://www.tbsnews.net/coronavirus-chronicle/covid-19-bangladesh/clinic-sealed-pabna-covid-19-sample-collection-sans	**Clinic sealed off in Pabna for Covid-19 sample collection sans approval**
17/07/2020		
18/07/2020	https://www.tbsnews.net/bangladesh/health/16-hospitals-recommended-international-passengers-covid-19-test-107941	**16 hospitals recommended for international passengers' Covid-19 test**
19/07/2020	https://www.tbsnews.net/bangladesh/health/khulna-upazila-hospitals-leave-patients-lurch-108352	**Khulna upazila hospitals leave patients in the lurch**
20/07/2020	https://www.tbsnews.net/coronavirus-chronicle/covid-19-bangladesh/no-covid-19-tests-treatment-except-govt-approved	**No Covid-19 tests, treatment except in govt-approved institutions: DGHS**
20/07/2020	https://www.tbsnews.net/coronavirus-chronicle/covid-19-bangladesh/testing-launched-provide-covid-19-certificates-travellers	**Testing launched to provide Covid-19 certificates for travellers: ISPR**
21/07/2020	https://www.tbsnews.net/coronavirus-chronicle/covid-19-bangladesh/test-starts-proposed-covid-19-facility-mohakhali-under	**Test starts at proposed Covid-19 facility in Mohakhali under army supervision**
22/07/2020	https://www.tbsnews.net/coronavirus-chronicle/covid-19-bangladesh/bangladesh-makes-covid-19-certificate-mandatory-most	**Bangladesh makes Covid-19 certificate mandatory for most foreigners to depart**
23/07/2020		
24/07/2020		
25/07/2020	https://www.tbsnews.net/coronavirus-chronicle/covid-19-bangladesh/contact-numbers-covid-19-testing-centres-expats-111286	**Contact numbers of Covid-19 testing centres for expats**
26/07/2020	https://www.tbsnews.net/coronavirus-chronicle/brac-school-joins-global-project-analysing-gendered-dynamics-covid-19-111793	**Online centre launched to expand Covid-19 and gender studies**
27//07/2020	https://www.tbsnews.net/coronavirus-chronicle/covid-19-bangladesh/igp-inaugurates-covid-19-ward-amcgh-112375	**IGP inaugurates Covid-19 ward at AMCGH**
27//07/2020	https://www.tbsnews.net/coronavirus-chronicle/covid-19-bangladesh/dr-zafrullah-wants-pm-open-gonoshasthaya-corona-unit	**Dr Zafrullah wants PM to open Gonoshasthaya corona unit**
28/07/2020	https://www.tbsnews.net/coronavirus-chronicle/covid-19-bangladesh/iom-supports-government-led-efforts-points-entry-combat	**IOM supports government-led efforts at points of entry to combat Covid-19**
28/07/2020	https://www.tbsnews.net/coronavirus-chronicle/covid-19-bangladesh/chevron-provides-covid-19-relief-nine-schools-dhaka-112693	**Chevron provides Covid-19 relief to nine schools in Dhaka**

Date of the news	News URL	Title of the news
29/07/2020		
30/07/2020	https://www.tbsnews.net/coronavirus-chronicle/covid-19-bangladesh/icddrb-provide-covid-19-free-certificates-foreigners	**Icddr,b to provide Covid-19-free certificates to foreigners**
31/07/2020	https://www.tbsnews.net/companies/urmi-group-introduces-high-performace-anti-viral-face-mask-turag-protex-114112	**Urmi Group introduces high performace anti-viral face mask "Turag Protex"**
31/07/2020	https://www.tbsnews.net/coronavirus-chronicle/covid-19-bangladesh/pcr-labs-remain-open-covid-19-test-during-eid-holidays	**PCR labs to remain open for Covid-19 test during Eid holidays**
01/08/2020	https://www.tbsnews.net/economy/rmg/coronavirus-takes-away-joys-eid-rmg-workers-gazipur-114427	**Coronavirus takes away the joys of Eid from RMG workers in Gazipur**
01/09/2020	https://www.tbsnews.net/bangladesh/health/right-way-127108	**Is this the right way?**
01/09/2020	https://www.tbsnews.net/coronavirus-chronicle/covid-19-bangladesh/bus-services-resume-fully-amid-no-clear-trend-infection	**Bus services resume fully amid no clear trend of infection**
01/09/2020	https://www.tbsnews.net/economy/rmg/rmg-exports-back-track-after-7-months-127105	**RMG exports back on track after 7 months**
01/09/2020	https://www.tbsnews.net/economy/smes-deserve-more-support-amid-covid-19-pandemic-speakers-127084	**SMEs deserve more support amid Covid-19 pandemic: Speakers**
02/09/2020	https://www.tbsnews.net/bangladesh/education/university-students-get-cheap-internet-127603	**University students to get cheap internet**
02/09/2020	https://www.tbsnews.net/bangladesh/education/class-8-students-get-promotion-without-exams-127582	**Class-8 students to get promotion without exams**
02/09/2020	https://www.tbsnews.net/feature/panorama/saki-chowdhury-queen-awardees-quest-raise-funds-bangladeshi-covid-19-victims-127174	**Saki Chowdhury: A Queen Awardee's quest to raise funds for Bangladeshi Covid-19 victims**
02/09/2020	https://www.tbsnews.net/feature/panorama/pandemic-silences-musical-instrument-127729	**The pandemic silences musical instrument**
03/09/2020	https://www.tbsnews.net/world/global-economy/japan-adds-india-and-bangladesh-china-exit-subsidy-destinations-128035	**Japan adds India and Bangladesh to 'China exit' subsidy destinations**
03/09/2020	https://www.tbsnews.net/economy/industry/covid-crushes-local-rod-cement-business-127690	**Covid crushes local rod-cement business**
04/09/2020	https://www.tbsnews.net/economy/rmg/apparel-factories-look-workers-hinting-recovery-128446	**Apparel factories look for workers, hinting at recovery**
04/09/2020	https://www.tbsnews.net/coronavirus-chronicle/covid-19-bangladesh/korean-cro-conduct-phase-iii-trial-sinovacs-covid-19	**icddr,b to run the Covid-19 vaccine trial in Bangladesh, Korean CRO will monitor trial data**
05/09/2020	https://www.tbsnews.net/bangladesh/long-road-recovery-real-estate-sector-128878	**Long road to recovery for real estate sector**
05/09/2020	https://www.tbsnews.net/economy/trade/exports-cruising-nicely-amid-pandemic-128869	**Exports cruising nicely amid pandemic**

Date of the news	News URL	Title of the news
05/09/2020	https://www.tbsnews.net/economy/missed-instalments-leave-many-realtors-soup-128875	**Missed instalments leave many realtors in the soup**
05/09/2020	https://www.tbsnews.net/bangladesh/migration/returnee-women-migrants-receive-covid-19-prevention-packages-128854	**Returnee women migrants to receive Covid-19 prevention packages**
05/09/2020	https://www.tbsnews.net/economy/covid-19-women-bear-economic-brunt-most-128506	**Covid-19: Women bear the economic brunt the most**
05/09/2020	https://www.tbsnews.net/bangladesh/transport/6-lakh-smart-driving-licence-seekers-caught-brta-puzzle-128515	**6 lakh smart driving licence seekers caught in BRTA puzzle**
06/09/2020	https://www.tbsnews.net/economy/banking/internet-banking-transaction-ceiling-enhanced-four-times-129334	**Internet Banking transaction ceiling enhanced by four times**
06/09/2020	https://www.tbsnews.net/bangladesh/education/public-university-students-get-loan-smartphone-129319	**Public university students to get loan for smartphone**
06/09/2020	https://www.tbsnews.net/coronavirus-chronicle/covid-19-bangladesh/bangladesh-completes-genome-sequencing-263-covid-19	**Bangladesh completes genome sequencing of 263 Covid-19 samples**
07/09/2020	https://www.tbsnews.net/analysis/6-months-covid-bangladesh-looking-back-and-forward-129769	**6 months of Covid in Bangladesh: Looking back and forward**
07/09/2020	https://www.tbsnews.net/economy/banking/stimulus-packages-disbursement-picks-business-normalises-129760	**Stimulus packages: Disbursement picks up as business normalises**
07/09/2020	https://www.tbsnews.net/analysis/economy-recovery-track-129754	**Economy on a recovery track**
07/09/2020	https://www.tbsnews.net/coronavirus-chronicle/covid-19-bangladesh/brac-university-fast-track-cold-chain-assessment-design	**BRAC University to fast-track cold-chain assessment, design of mass Covid-19 vaccination**
07/09/2020	https://www.tbsnews.net/coronavirus-chronicle/covid-19-bangladesh/bangladesh-army-donates-covid-19-medical-equipment	**Bangladesh Army donates Covid-19 medical equipment to Evercare Hospital**
07/09/2020	https://www.tbsnews.net/economy/banking/brac-bank-offers-healthcare-package-mastercard-users-129529	**BRAC Bank offers healthcare package for MasterCard users**
07/09/2020	https://www.tbsnews.net/economy/rmg/covid-19-affected-livelihoods-82pc-rmg-workers-sanem-129505	**Covid-19 affected livelihoods of 82% of RMG workers: Sanem**
08/09/2020	https://www.tbsnews.net/analysis/v-shaped-recovery-likely-health-worries-still-remain-strong-130249	**As a V-shaped recovery is likely, health worries still remain strong**
08/09/2020	https://www.tbsnews.net/analysis/smes-need-be-salvaged-complete-recovery-130243	**SMEs need to be salvaged for complete recovery**
08/09/2020	https://www.tbsnews.net/economy/mixed-bag-recovery-130252	**A mixed bag of recovery**
08/09/2020	https://www.tbsnews.net/economy/dhaka-opens-arms-japanese-investment-130234	**Dhaka opens arms to Japanese investment**

Date of the news	News URL	Title of the news
08/09/2020	https://www.tbsnews.net/coronavirus-chronicle/covid-19-bangladesh/dghs-suspends-covid-19-testing-permission-aalok-health	**DGHS suspends Covid-19 testing permission of Aalok Health Care**
08/09/2020	https://www.tbsnews.net/bangladesh/education/ministry-asks-authorities-prepare-reopening-primary-schools-130168	**Ministry asks authorities to prepare for reopening primary schools**
08/09/2020	https://www.tbsnews.net/coronavirus-chronicle/covid-19-bangladesh/isde-distributes-food-disinfectant-700-jobless-families	**ISDE distributes food, disinfectant to 700 jobless families**
08/09/2020	https://www.tbsnews.net/analysis/covid-19-six-months-how-pandemic-affected-health-and-economy-bangladesh-130066	**Covid-19 six months: How the pandemic affected health and economy of Bangladesh**
08/09/2020	https://www.tbsnews.net/thoughts/covid-19-double-blow-youth-130018	**Covid-19 is a double blow for the youth**
08/09/2020	https://www.tbsnews.net/feature/panorama/where-new-normal-129832	**Where is the 'new normal'?**
09/09/2020	https://www.tbsnews.net/bangladesh/revenue-generation-shows-little-sign-recovery-130780	**Revenue generation shows little sign of recovery**
09/09/2020	https://www.tbsnews.net/bangladesh/education/covid-19-school-closure-cause-skill-loss-throughout-century-130774	**Covid-19 school loss to hurt economy through the century**
09/09/2020	https://www.tbsnews.net/economy/stock/associated-oxygen-ipo-subscription-begins-thursday-130738	**Associated Oxygen IPO subscription begins Thursday**
09/09/2020	https://www.tbsnews.net/economy/stock/associated-oxygen-ipo-subscription-begins-thursday-130738	**Banks' CSR expenditure in health sector almost doubles during pandemic**
10/09/2020	https://www.tbsnews.net/bangladesh/health/online-appointments-outdoor-medical-services-bsmmu-available-now-131266	**Online appointments for outdoor medical services at BSMMU available now**
10/9/2020	https://www.tbsnews.net/economy/industry/tourism-needs-skilled-workforce-131122	**Tourism needs skilled workforce**
10/09/2020	https://www.tbsnews.net/bangladesh/applied-all-countries-covid-19-vaccine-pm-130978	**Applied to all countries for Covid-19 vaccine: PM**
10/09/2020	https://www.tbsnews.net/companies/guardian-life-insurance-launches-online-programme-youths-skill-development-130972	**Guardian Life Insurance launches online programme for youth's skill development**
11/9	https://www.tbsnews.net/coronavirus-chronicle/covid-19-bangladesh/govt-will-bring-vaccine-wherever-available-momen-131560	**Govt will bring vaccine from wherever available: Momen**
11/09/2020	https://www.tbsnews.net/economy/rules-cards-protect-online-buyers-interests-131665	**Rules on cards to protect online buyers' interests**
11/09/2020	https://www.tbsnews.net/thoughts/diminishing-dreams-english-medium-private-candidates-131302	**Diminishing dreams of English medium private candidates**
12/09/2020	https://www.tbsnews.net/bangladesh/telecom/robi-clocks-33-profit-amid-pandemic-132025	**Robi clocks 3.3% profit amid pandemic**
12/09/2020	https://www.tbsnews.net/bangladesh/law-order/right-health-needs-be-preserved-amid-pandemic-legal-experts-132028	**Right to health needs to be preserved amid pandemic: Legal experts**

Date of the news	News URL	Title of the news
12/09/2020	https://www.tbsnews.net/economy/aviation/no-more-seating-restrictions-domestic-flights-tomorrow-caab-132001	**No more seating restrictions on domestic flights from tomorrow: CAAB**
12/09/2020	https://www.tbsnews.net/bangladesh/education/revamped-education-policy-focus-e-learning-dipu-moni-131992	**Revamped education policy to focus on e-learning : Dipu Moni**
13/09/2020	https://www.tbsnews.net/economy/banking/school-banking-thrives-amid-pandemic-132433	**School banking thrives amid pandemic**
13/09/2020	https://www.tbsnews.net/economy/banking/5-lakh-social-safety-net-accounts-opened-during-apr-jun-132436	**5 lakh social safety net accounts opened during Apr-Jun**
13/09/2020	https://www.tbsnews.net/bangladesh/education/class-xi-admission-process-begins-132331	**College admission begins**
14/09/2020	https://www.tbsnews.net/economy/aviation/pilots-overstressed-132973	**Pilots overstressed**
14/09/2020	https://www.tbsnews.net/bangladesh/health/evercare-hospital-offers-bmt-services-first-time-bangladesh-132910	**Evercare Hospital offers all kinds of BMT services for first time in Bangladesh**
14/09/2020	https://www.tbsnews.net/coronavirus-chronicle/covid-19-bangladesh/significant-losses-income-due-covid-19-lead-food	**Income losses lead to food insecurity in Cox's Bazar Sadar during pandemic: WFP**
15/09/2020	https://www.tbsnews.net/economy/ctg-customs-posts-positive-growth-revenue-collection-133462	**Ctg customs posts positive growth in revenue collection**
15/09/2020	https://www.tbsnews.net/bangladesh/govt-job-seekers-get-flexibility-age-limit-due-pandemic-133459	**Govt job seekers to get flexibility on age-limit due to pandemic**
15/09/2020	https://www.tbsnews.net/bangladesh/education/jsc-jdc-students-be-promoted-evaluation-schools-ministry-133447	**JSC, JDC students to be promoted as per evaluation by schools: Ministry**
15/09/2020	https://www.tbsnews.net/bangladesh/transport/100pc-train-ticket-sale-begins-wednesday-133300	**Passenger trains to resume operation at full capacity from Wednesday**
15/09/2020	https://www.tbsnews.net/economy/adb-forecasts-68-percent-growth-bangladesh-current-fiscal-133039	**ADB sees robust growth for Bangladesh, fourth in Asia**
16/09/2020	https://www.tbsnews.net/economy/655-beneficiaries-social-safety-nets-are-non-poor-cpd-133675	**Non-poor eatup bigger pie of safety allowances**
17/09/2020	https://www.tbsnews.net/coronavirus-chronicle/covid-19-bangladesh/saif-power-group-donates-4-sets-high-power-oxygen-nasal	**Saif Power Group donates 4 sets of high-power oxygen nasal cannulas to Cox's Bazar district administration**
17/09/2020	https://www.tbsnews.net/coronavirus-chronicle/covid-19-bangladesh/covid-19-status-capitals-3-hospitals-discarded-134221	**Covid-19 status of capital's 3 hospitals discarded**
18/09/2020	https://www.tbsnews.net/coronavirus-chronicle/covid-19-bangladesh/chattogram-covid-facilities-shutting-down-over-fund-crisis	**Chattogram Covid facilities shutting down over fund crisis**
19/09/2020	https://www.tbsnews.net/coronavirus-chronicle/covid-19-bangladesh/scientists-find-sars-cov-2-rna-bangladesh-wastewater	**Scientists find SARS-CoV-2 RNA in Bangladesh wastewater**

Date of the news	News URL	Title of the news
20/09/2020	https://www.tbsnews.net/bangladesh/labour-welfare-foundation-provides-tk40cr-9500-workers-135493	**Labour Welfare Foundation provides Tk40cr to 9,500 workers**
21/09/2020	https://www.tbsnews.net/bangladesh/ngos-crisis-pandemic-shrinks-funds-135976	**NGOs in crisis as pandemic shrinks funds**
21/09/2020	https://www.tbsnews.net/coronavirus-chronicle/covid-19-bangladesh/bangladesh-talks-least-5-vaccine-makers-135904	**'Bangladesh in talks with at least 5 vaccine makers'**
21/09/2020	https://www.tbsnews.net/coronavirus-chronicle/covid-19-bangladesh/pm-all-out-preparations-fight-possible-second-wave-covid	**Prepare to tackle second wave of Covid-19: PM**
21/09/2020	https://www.tbsnews.net/bangladesh/wfp-provides-cash-assistance-urban-poor-meet-food-nutrition-needs-135649	**WFP provides cash assistance to urban poor to meet food, nutrition needs**
21/09/2020	https://www.tbsnews.net/coronavirus-chronicle/covid-19-bangladesh/govt-allows-antigen-tests-135631	**Govt allows antigen tests**
22/09/2020	https://www.tbsnews.net/coronavirus-chronicle/covid-19-bangladesh/pmo-preserves-premiers-instructions-covid-deal-future	**PMO preserves premier's instructions on Covid to deal with future epidemic**
22/09/2020	https://www.tbsnews.net/coronavirus-chronicle/covid-19-bangladesh/govt-against-lockdown-keep-economy-afloat-cabinet-secy	**Govt against lockdown to keep economy afloat: Cabinet secretary**
23/09/2020	https://www.tbsnews.net/coronavirus-chronicle/bold-public-spending-needed-covid-19-economic-recovery-unctad-136624	**Bold public spending needed for Covid-19 economic recovery: UNCTAD**
23/09/2020	https://www.tbsnews.net/economy/aid-commitment-drops-will-improve-136432	**Aid commitment drops but will improve**
24/09/2020	https://www.tbsnews.net/coronavirus-chronicle/covid-19-bangladesh/front-line-doctors-distress-137359	**Front line doctors in distress**
24/09/2020	https://www.tbsnews.net/coronavirus-chronicle/covid-19-bangladesh/when-virus-leaves-its-damage-lingers-136930	**When the virus leaves but its damage lingers**
25/09/2020	https://www.tbsnews.net/coronavirus-chronicle/covid-19-bangladesh/pandemic-forces-mom-sell-flowers-holding-20-day-old-baby	**Pandemic forces mom to sell flowers holding 20-day-old baby in lap**
26/09/2020	https://www.tbsnews.net/economy/7000-digital-entrepreneurs-emerge-ctg-pandemic-137698	**7,000 digital entrepreneurs emerge in Ctg in pandemic**
26/09/2020	https://www.tbsnews.net/coronavirus-chronicle/covid-19-bangladesh/glimpse-betel-leaf-sellers-life-amid-pandemic-137692	**A glimpse into a betel-leaf seller's life amid pandemic**
26/09/2020	https://www.tbsnews.net/bangladesh/world-bank-approves-200m-bangladesh-accessing-safe-water-137782	**E-commerce outlets offering grocery see highest growth**
27/09/2020	https://www.tbsnews.net/coronavirus-chronicle/covid-19-bangladesh/undp-launches-maskupbangladesh-138403	**UNDP launches #MaskUpBangladesh**
27/09/2020	https://www.tbsnews.net/economy/rajshahi-sugar-mill-staff-unpaid-three-months-138085	**Rajshahi Sugar Mill staff unpaid for three months**

Date of the news	News URL	Title of the news
27/09/2020	https://www.tbsnews.net/coronavirus-chronicle/covid-19-bangladesh/hairdresser-chunnu-wait-more-clients-138121	**Hairdresser Chunnu in wait for more clients**
28/09/2020	https://www.tbsnews.net/bangladesh/8th-5-year-plan-quick-recovery-pandemic-shocks-gets-top-priority-138880	**8th 5-year plan: Quick recovery from pandemic shocks gets top priority**
28/09/2020	https://www.tbsnews.net/coronavirus-chronicle/covid-19-bangladesh/covid-19-double-edged-sword-dentist-138514	**Covid-19: A double-edged sword for a dentist**
29/09/2020	https://www.tbsnews.net/bangladesh/education/closure-educational-institutions-extended-till-october-31-139273	**Closure of educational institutions extended till October 31**
29/09/2020	https://www.tbsnews.net/bangladesh/education/interactive-cares-launches-e-learning-platform-interactive-cares-courses-139186	**Interactive Cares launches e-learning platform 'Interactive Cares Courses'**
29/09/2020	https://www.tbsnews.net/bangladesh/health/how-are-our-cardiac-patients-doing-during-pandemic-138928	**How are our cardiac patients doing during the pandemic?**
29/09/2020	https://www.tbsnews.net/bangladesh/health/service-closure-leaves-ctg-cardiac-patients-lurch-138925	**Service closure leaves Ctg cardiac patients in the lurch**
30/09/2020	https://www.tbsnews.net/coronavirus-chronicle/covid-19-bangladesh/uk-vaccine-be-available-bangladesh-no-clinical-trial-here	**UK vaccine to be available to Bangladesh, no clinical trial here: UK envoy**

Ghana Media articles

DD/MM/YYYY	Author Affiliation	Newspaper	Title
14/03/2020	Daily Guide Network<br><br>Melvin Tarlue	Daily Guide Network	Banks Take Precautionary Measures Against Coronavirus
16/03/2020	Daily Guide Network	Daily Guide Network	Report responsibly on covid-19
16/03/2020	Daily Guide Network	Daily Guide Network	Akufo-Addo Meets To Strategise How To Produce Sanitizers And Medicines In Ghana For Coronavirus
17/03/2020	Daily Guide Network<br>Melvin Tarlue	Daily Guide Network	Jack Ma Foundation Donates Coronavirus Testing Kits To Africa
18/03/2020	Daily Guide Network<br><br>Melvin Tarlue	Daily Guide Network	Coronavirus: NYA Urges Youth To Take Precaution
18/03/2020	Daily Guide Network	Daily Guide Network	GHAMRO Cautions Its Members

DD/MM/YYYY	Author Affiliation	Newspaper	Title
	George Clifford Owusu		
18/03/2020	Daily Guide Network<br><br>Sharon Willis Brown-Acquah	Daily Guide Network	Ghana CEOs Join Fight Against Coronavirus
18/03/2020	Daily Guide Network<br><br>Melvin Tarlue	Daily Guide Network	Coronavirus Outbreak: Ghanaian Churches Hold Digital Services
19/03/2020	Daily Guide Network<br><br>Not listed	Daily Guide Network	Delta Agro Limited Gives To Korle-Bu In Coronavirus Prevention
19/03/2020	Daily Guide Network<br><br>Jamila Akweley Okertchiri	Daily Guide Network	Mulan Activity Center Gives Back
20/03/2020	Daily Guide Network<br><br>Not listed	Daily Guide Network	St. John's Fertility Centre Donates To GIS
20/03/2020	Daily Guide Network<br><br>Melvin Tarlue	Daily Guide Network	Ghana Bar Association Issues Guidelines Over Coronavirus
27/03/2020	Daily Guide Network	Daily Guide Network	Napo Offers Covid-19 Suggestions
28/03/2020	Daily Guide Network	Daily Guide Network	Govt Disinfects Kumasi Markets
29/03/2020	Daily Guide Network	Daily Guide Network	Guinness Ghana Supports Fight Against Covid-19 Registration
30/03/2020	Daily Guide Network	Daily Guide Network	Ofori-Atta Predicts Drop In 2020 GDP

DD/MM/YYYY	Author Affiliation	Newspaper	Title
30/03/2020	Daily Guide Network	Daily Guide Network	ADB Fights Covid-19 With GHC1m
30/03/2020	Daily Guide Network	Daily Guide Network	Andre Ayew Supports COVID-19 Fight In Northern Region
30/03/2020	Daily Guide Network	Daily Guide Network	Coronavirus Update: Drones Spray Markets In Bono Region
30/03/2020	Daily Guide Network	Daily Guide Network	Great Kofata Gives Gh100, 000 In Coronavirus Fight
04/04/2020	Daily Guide Network	Daily Guide Network	New Data Methods Helping Fight Against Covid-19 – GSS
04/04/2020	Daily Guide Network	Daily Guide Network	Foodstuff Locked Up At Techiman Market
05/04/2020	Daily Guide Network	Daily Guide Network	Prophet Owusu Bempah Donates To Ghana Prisons Service

DD/MM/YYYY	Author Affiliation	Newspaper	Title
06/04/2020	Daily Guide Network	Daily Guide Network	Gospel Artiste Ohemaa Mercy Distributes Hand Sanitizers To Market Women
06/04/2020	Daily Guide Network	Daily Guide Network	Massive Disinfection In WR Markets
06/04/2020	Daily Guide Network	Daily Guide Network	Female Doctors Support Vulnerable People In Kumasi
26/03/2020	Daily Guide Network<br><br>Not listed	Daily Guide Network	COVID-19: Scrap CST To Aid Online Studies
26/03/2020	Daily Guide Network<br><br>Not listed	Daily Guide Network	Kasapreko Donates 2,400 Bottles Of Sanitizers To MOI
26/03/2020	Daily Guide Network<br><br>Not listed	Daily Guide Network	Lockdown Now! Doctors Urge Nana
26/03/2020	Daily Guide Network<br><br>Not listed	Daily Guide Network	Covid-19 Cushion: Republic Bank Defers Loan Repayment
26/03/2020	Daily Guide Network<br><br>Not listed	Daily Guide Network	Akufo-Addo Hosts Transport Owners
25/03/2020	Daily Guide Network<br><br>Not listed	Daily Guide Network	Covid-19 Measures: Gov't Cleans Agbogbloshie Market
25/03/2020	Daily Guide Network	Daily Guide Network	Enterprise Group Donates To 3 Health Outfits

DD/MM/YYYY	Author Affiliation	Newspaper	Title
	Not listed		
25/03/2020	Daily Guide Network<br><br>Not listed	Daily Guide Network	TUC Demands Possible Shutdown
25/03/2020	Daily Guide Network<br><br>Not listed	Daily Guide Network	Govt Donates Covid-19 Items In UE
25/03/2020	Daily Guide Network<br><br>Not listed	Daily Guide Network	GIBA, GJA Train On COVID-19 Communications
25/03/2020	Daily Guide Network<br><br>Not listed	Daily Guide Network	Kasapreko Donates 2,400 Bottles Of Sanitizers To MOI To Aid Fight Against COVID 19
**DD/MM/YYYY**	**Author Affiliation**	**Newspaper**	**Title**
28/04/2020	Daily Guide Network	Daily Guide Network	AFCONS Infrastructure Supports Assembly With PPE
29/04/2020	Daily Guide Network	Daily Guide Network	Small Scale Miners, Others Assist Covid-19 Fund
29/04/2020	Daily Guide Network	Daily Guide Network	FON Packaging Gives to Accra Regional Hospital
30/04/2020	Daily Guide Network	Daily Guide Network	Nestlé Ghana Suppprts Covid-19 Relief Efforts
30/04/2020	Daily Guide Network	Daily Guide Network	Muslims Donate To Covid-19 Fund
1/05/2020	Daily Guide Network	Daily Guide Network	45 Arrested For Holding Church Service
1/05/2020	Daily Guide Network	Daily Guide Network	Zenith Bank announces enhancement of its USSD Service *966#
2/05/2020	Daily Guide Network	Daily Guide Network	ADB Donates To 4 Medical Institutions In Central Region
4/05/2020	Daily Guide Network	Daily Guide Network	Free Food For Para-Athletes…To Face Covid-19
4/05/2020	Daily Guide Network	Daily Guide Network	Tobinco To Produce Covid-19 Drugs
4/05/2020	Daily Guide Network	Daily Guide Network	Azar Group Supports Covid-19 Trust Fund
5/05/2020	Daily Guide Network	Daily Guide Network	Abroso Supports Covid-19 Fight
5/05/2020	Daily Guide Network	Daily Guide Network	Dr Caesar Donates Lina Energy Tea To Korle-Bu Hospital

DD/MM/YYYY	Author Affiliation	Newspaper	Title
5/05/2020	Daily Guide Network	Daily Guide Network	Nyonkopa, Bc Ghana Support Cocoa Farmers
5/05/2020	Daily Guide Network	Daily Guide Network	3 Groups Donate To Covid-19 Fund
**DD/MM/YYYY**	**Author Affiliation**	**Newspaper**	**Title**
22/04/2020	Daily Guide Network	Daily Guide Network	Shippers' Authority Assists Ridge, Tema Hospitals
23/04/2020	Daily Guide Network	Daily Guide Network	Standard Chartered Commits GHS1m To Fight Covid-19
23/04/2020	Daily Guide Network	Daily Guide Network	Suame Market Goes Double-Track
23/04/2020	Daily Guide Network	Daily Guide Network	Coronavirus Response: Face Mask Mandatory In Accra
23/04/2020	Daily Guide Network	Daily Guide Network	Sunyani, Techiman Markets Closed Down
23/04/2020	Daily Guide Network	Daily Guide Network	Deseret Hospital Gets Covid-19 Prevention Items
24/04/2020	Daily Guide Network	Daily Guide Network	Zoomlion Disinfects Prison Headquarters, James Camp Prisons And Soul Clinic School
24/04/2020	Daily Guide Network	Daily Guide Network	Charger Limited Aids War Memorial Hospital
26/04/2020	Daily Guide Network	Daily Guide Network	Soldiers Builds COVID-19 Isolation Centre At Kwabenya
28/04/2020	Daily Guide Network	Daily Guide Network	PZ Traders Undergo Covid-19 Test
28/04/2020	Daily Guide Network	Daily Guide Network	Agormanya Market Closed Down
16/04/2020	Daily Guide Network	Daily Guide Network	Rebecca Foundation Presents Relief Boxes To Greater Kumasi
16/04/2020	Daily Guide Network	Daily Guide Network	Rebecca, Merck Foundations Donate Relief Boxes To Less Privileged
18/04/2020	Daily Guide Network	Daily Guide Network	LPG Marketers Association Donates To Covid-19 Fund
18/04/2020	Daily Guide Network	Daily Guide Network	Zoomlion Donates $20,000 To Noguchi
18/04/2020	Daily Guide Network	Daily Guide Network	Gov't Decides On 200 Pregnant Kayayies
19/04/2020	Daily Guide Network	Daily Guide Network	Victory Bible Church Donates Food To Over 2,000 Awoshie Residents
20/04/2020	Daily Guide Network	Daily Guide Network	Zipline Ghana Begin Emergency Delivery Of Covid-19 Test Samples
21/04/2020	Daily Guide Network	Daily Guide Network	COVID-19 : Hisense, Despite Group Donate To Ayawaso West
21/04/2020	Daily Guide Network	Daily Guide Network	AH Hotel Enjoys Zoomlion Disinfection Service

DD/MM/YYYY	Author Affiliation	Newspaper	Title
22/04/2020	Daily Guide Network	Daily Guide Network	President Pushes For Guidelines For Face Masks Production
16/04/2020	Daily Guide Network<br><br>Not listed	Daily Guide Network	Covid-19 Fight: Betway Supports Government With GHS 150,000
15/04/2020	Daily Guide Network<br><br>Not listed	Daily Guide Network	Zenith Bank Supports COVID-19 Fund With GHS1m
15/04/2020	Daily Guide Network<br><br>Not listed	Daily Guide Network	Access Bank Joins COVID-19 Fight With Ambulance Donation To UPSA
15/04/2020	Daily Guide Network<br><br>Not listed	Daily Guide Network	8 Companies Donate To Covid-19 Fund
15/04/2020	Daily Guide Network<br><br>Not listed	Daily Guide Network	Alpha Lotto Donates Ghc100,000 To Covid-19 Fund
14/04/2020	Daily Guide Network<br><br>Not listed	Daily Guide Network	GB Foods Supports Covid-19 Fund
14/04/2020	Daily Guide Network<br><br>Not listed	Daily Guide Network	Jay Kay Industries Donates To Covid-19 Fund
14/04/2020	Daily Guide Network<br><br>Emmanuel Opoku	Daily Guide Network	Gold Fields Support COVID-19 Fight With $833,000
14/04/2020	Daily Guide Network<br><br>Not listed	Daily Guide Network	COVID-19 : TT Brothers Show Love To Tema SOS
13/04/2020	Daily Guide Network<br><br>Not listed	Daily Guide Network	Covid-19: 70 Doctors Trained For Risk Communication
13/04/2020	Daily Guide Network<br><br>Not listed	Daily Guide Network	Safebond Supports Police, Military In Covid-19 Fight

DD/MM/YYYY	Author Affiliation	Newspaper	Title
13/04/2020	Daily Guide Network<br><br>Not listed	Daily Guide Network	Coronavirus Response: Bawumia Launches Covid-19 Tracker App
12/04/2020	Daily Guide Network<br><br>Not listed	Daily Guide Network	B5 Plus Fights COVID 19 With Free Oxygen
11/04/2020	Daily Guide Network<br><br>Not listed	Daily Guide Network	Angel Supports K'si Hospitals
11/04/2020	Daily Guide Network<br><br>Not listed	Daily Guide Network	Ho Central Market to Be Closed On Market Days
10/04/2020	Daily Guide Network<br><br>Not listed	Daily Guide Network	COVID-19: Glico Provides Essentials To Prisons Service
10/04/2020	Daily Guide Network<br><br>Not listed	Daily Guide Network	Chobi Ghana Donates To Rebecca Foundation's Covid-19 Campaign
10/04/2020	Daily Guide Network<br><br>Not listed	Daily Guide Network	Sunda Donates Toiletries To KATH
10/04/2020	Daily Guide Network<br><br>Not listed	Daily Guide Network	Covid-19 Relief: IPMC Supports 500 Households In Nima
09/04/2020	Daily Guide Network<br><br>Not listed	Daily Guide Network	Nana Inaugurates Covid-19 Fund Board
09/04/2020	Daily Guide Network<br><br>Not listed	Daily Guide Network	Ghana Exim Bank Donates GHC250,000 To Covid-19 Fund
09/04/2020	Daily Guide Network<br><br>Not listed	Daily Guide Network	GIIF Donates Ghc 500k To Covid-19 Trust Fund
09/04/2020	Daily Guide Network<br><br>Not listed	Daily Guide Network	More Donations For Covid-19 Fight

DD/MM/YYYY	Author Affiliation	Newspaper	Title
09/04/2020	Daily Guide Network<br><br>Not listed	Daily Guide Network	Gov't To Launch New Mobile App Tracing Coronavirus Carriers
08/04/2020	Daily Guide Network<br><br>Not listed	Daily Guide Network	Mastercard Foundation, NBSSI Support MSMEs
08/04/2020	Daily Guide Network<br><br>Not listed	Daily Guide Network	Keda, Sunda International, Donate To Covid-19 Fund
08/04/2020	Daily Guide Network<br><br>Not listed	Daily Guide Network	Hollard Ghana Donates 100k To Covid-19 Fund
08/04/2020	Daily Guide Network<br><br>Not listed	Daily Guide Network	More Coys Donate To Covid-19 Fund
08/04/2020	Daily Guide Network<br><br>Not listed	Daily Guide Network	Obuasi Traders Resist Relocation Move
08/04/2020	Daily Guide Network<br><br>Not listed	Daily Guide Network	NYA Supports MoH With Essentials To Fight Covid-19
07/04/2020	Daily Guide Network<br><br>Daniel Frimpong	Daily Guide Network	CEO Network To Donate Ghc200,000 To Covid-19 Fund
07/04/2020	Daily Guide Network<br><br>Not listed	Daily Guide Network	Wilmar, Eastern Region MMDCEs Support Covid-19 Relief Fund
07/04/2020	Daily Guide Network<br><br>Not listed	Daily Guide Network	Republic Bank Supports Covid-19 Fight With $2m
07/04/2020	Daily Guide Network<br><br>Not listed	Daily Guide Network	MTN Announces Ghc 5m Package For Covid-19
07/04/2020	Daily Guide Network<br><br>Not listed	Daily Guide Network	More Donations For Covid-19 Fund

DD/MM/YYYY	Author Affiliation	Newspaper	Title
07/04/2020	Daily Guide Network<br><br>Not listed	Daily Guide Network	Foundation Supports Fight Against Covid-19.
26/5/2020	Daily Guide Network	Daily Guide Network	De Colours Foundation Fights Covid-19 In Ketu South
27/5/2020	Daily Guide Network	Daily Guide Network	Covid19 Relief: Ketu South MCE Lauds Foundation For 300 Disabled Persons
28/5/2020	Daily Guide Network Emmanuel Opoku	Daily Guide Network	Takoradi Markets Closed Down Over Covid-19
28/5/2020	Daily Guide Network	Daily Guide Network	Apsonic Motors Supports Tema Immigration
13/05/2020	Daily Guide Network	Daily Guide Network	GHANSU Shows Love To Members
13/05/2020	Daily Guide Network	Daily Guide Network	BIMA Ghana Offers GHS500,000 Support For Customers
13/05/2020	Daily Guide Network	Daily Guide Network	Charger Ltd CEO Supports Media In Upper East
13/05/2020	Daily Guide Network	Daily Guide Network	Ghana Friendship Association Of Edmonton Donates $20k To Covid-19 Fund
14/05/2020	Daily Guide Network	Daily Guide Network	Aurum Institute Supports Covid-19 Fight
15/05/2020	Daily Guide Network	Daily Guide Network	GGA Donates To Achimota Hospital
15/05/2020	Daily Guide Network	Daily Guide Network	Dagbon Forum Donates PPE In NR
15/05/2020	Daily Guide Network	Daily Guide Network	Registrar-General Releases Guidelines For AGMs
15/05/2020	Daily Guide Network	Daily Guide Network	ADR Practitioners Support Covid-19 Fund
16/05/2020	Daily Guide Network	Daily Guide Network	COVID-19 Vivo Energy and Retailers Install Water Storage Facilities at Lorry Parks and Markets
18/05/2020	Daily Guide Network	Daily Guide Network	Journalists Trained On Covid-19 Safety Measures
19/05/2020	Daily Guide Network	Daily Guide Network	Obuasi Rotational System Enters Second Week
20/05/2020	Daily Guide Network	Daily Guide Network	Absa Ghana Introduces 50 New Intelligent ATMs

DD/MM/YYYY	Author Affiliation	Newspaper	Title
03/06/2020	Daily Guide Network<br><br>Melvin Tarlue	Daily Guide Network	Gov't Releases Guidelines For Tourism, Hospitality Sectors
05/06/2020	Daily Guide Network	Daily Guide Network	Zoomlion Begins National Disinfection Exercise Of Mosques
10/06/2020	Daily Guide Network	Daily Guide Network	OLAM, Temasek Foundation Donate COVID-19 Test Kits
10/06/2020	Daily Guide Network<br><br>Jamila Akweley Okertchiri	Daily Guide Network	Intravenous Infusion Supports Noguchi
13/06/2020	Daily Guide Network	Daily Guide Network	GBA Donates PPE To Judicial Service
13/06/2020	Daily Guide Network<br><br>Issah Mohammed	Daily Guide Network	Accra Friendship Club Supports Mamobi Hospital
16/06/2020	Daily Guide Network<br><br>Eric Kombat	Daily Guide Network	POS Foundation Supports Tamale Central Prison With PPEs To Fight Covid-19

DD/MM/YYYY	Author Affiliation	Newspaper	Title
06/05/2020	Daily Guide Network	Daily Guide Network	GCB Bank Donates 40 Jumbo Poly Tanks To Gov't
06/05/2020	Daily Guide Network	Daily Guide Network	Baptist Donates To Covid-19 Fund
06/05/2020	Daily Guide Network	Daily Guide Network	Qatar Charity, COMOG Assist 200 Families
7/05/2020	Daily Guide Network	Daily Guide Network	Fidelity Supports Covid-19 Isolation Facility
7/05/2020	Daily Guide Network	Daily Guide Network	Banks To Raise GHS3 Billion - To Support Hospitality Industry
7/05/2020	Daily Guide Network	Daily Guide Network	Fabrimetal Partners Gov't In Covid19 Fight; Pledges To Provide Oxygen
7/05/2020	Daily Guide Network	Daily Guide Network	B5 Plus Dismisses Price Increment Report; Offers Free Oxygen For Hospitals
8/05/2020	Daily Guide Network	Daily Guide Network	Trust Hospital Closes for Fumigation
8/05/2020	Daily Guide Network	Daily Guide Network	Churches Prepare For Reopening
12/05/2020	Daily Guide Network	Daily Guide Network	Hollard Provides Free Covid-19 Cover For All
13/05/2020	Daily Guide Network	Daily Guide Network	Spare Parts Dealers, Others Support Covid Fund
29/06/2020	Ellen Dapaah	Citi newsroom	GMA elated over extension of tax reliefs, allowances for health workers
30/6/2020	Daily Guide Network Jamila Akweley Okertchiri	Daily Guide Network	The FDA seizes fake covid-19 drug
30/6/2020	Daily Guide Network Melvin Tarlue	Daily Guide Network	Greater Accra Bar Fights Coronavirus At Accra High Court
30/6/2020	Daily Guide Network Melvin Tarlue	Daily Guide Network	Comply with covid-19 restrictions
01/07/2020	Daily Guide Network Jamila Akweley Okertchir	Daily Guide Network	Euroget Donates Ambulance To GHS
2/7/2020	Daily Guide Network	Daily Guide Network	Commercial Drivers Strike In Takoradi

DD/MM/YYYY	Author Affiliation	Newspaper	Title
	Emmanuel Opoku		
02/07/2020	Daily Guide Network<br>Daniel Bampoe	Daily Guide Network	ECG Power Queens Supports Koforidua Prisons
2/7/2020	Desmond Kofi Tawiah	Graphic online	COVID-19 Private Sector Fund appeals for support G
2/7/2020	Victoria Kissiedu	Graphic online	YINSON supports COVID-19 fight with biological safety cabinet
2/7/2020	Bbc.com	Graphic online	Corona Quacks: Exposing fake coronavirus cures in Ghana
2/7/2020	Joshua Bediako Koomson	Graphic online	Military deployment to ensure border safety - Defence Minister
2/7/2020	Emmanuel Bruce	Graphic online	Springboard partners two institutions to launch COVID-19 recovery programme -Aims to support 692,000 young people
17/07/2020	Daily Guide Network	Daily Guide Network	Fidelity Bank Builds Capacity Of SMEs
17/06/2020	Daily Guide Network<br>Malvin Tarlue	Daily Guide Network	Gov't Working To Set Up 2 Covid-19 Hospitals In Ashanti Region Minister
18/06/2020	Daily Guide Network<br>Enerst Kofi Adu	Daily Guide Network	NALAG Wants Assembly Members In Covid-19 Fight
18/06/2020<br>17/06/2020	Graphic.com.gh	Graphic online	HELEH Africa Foundation donates to Covid 19 National Trust Fund
20/06/2020<br>18/6/2020	Albert Tetteh Wayoe	Graphic online	Swimming: GSA issues guidelines for reopening of pools
23/07/2020	Daily Guide Network<br>Jamila Akweley Okertchiri	Daily Guide Network	China's First Lady Supports Mrs Akufo-Addo In Covid-19 Fight
23/07/2020	Daily Guide Network	Daily Guide Network	Local Govt Ministry Applauded For 2nd Disinfection Exercise In Markets, Lorry Parks

DD/MM/YYYY	Author Affiliation	Newspaper	Title
23/07/2020	Maxwell Akalaare Adombila	Graphic online	Large enterprises to benefit from GH₵1.2bn stimulus package
24/07/2020	Daily Guide Network	Daily Guide Network	Demand For Educational TV Channel Grows Amid COVID-19
24/07/2020	Graphic.com.gh	Graphic online	Markets, lorry parks in Ahafo undergo second disinfection exercise
14/07/2020	Graphic.com.gh	Graphic online	BOST Fumigate Head Office in wake of covid-19 infection
14/07/2020	Graphic<br><br>Linda Sarfoa Antwi	Graphic showbiz	'Corona life' to start streaming on Tuesday
14/07/2020	Graphic.com.gh	Graphic online	SafeCare 4Covid App introduced for health care workers
14/07/2020	Graphic.com.gh	Graphic online	Covid-19: Ibrahim Mahama donates anaesthetic machine to UGMC
15/07/2020	My joy online.com	My joy online	FLF Africa launches financial literacy for corona virus Alleviation
15/07/2020	Daily Guide Network<br><br>Jamila Akweley Okertchiri	Daily Guide	PSGH Supports Pharmacists
15/07/2020	Daily Guide Network<br><br>Melvin Tarlue	Daily Guide	Arrest, Prosecute Persons Who Flout Covid-19 Protocols GBA
15/07/2020	Emelia Ennin Abbey	Graphic	Fake contact tracer arrested
16/7/2020	Daily Guide Network<br><br>y Sharon Willis Brown-Acquah	Daily Guide	CEO Network Makes 2nd Donation To Covid-19 Trust Fund
16/7/2020	Daily Guide Network	Daily Guide	CRI Rejects Shutting Down Schools
17/7/2020	Daily Guide Network	Daily Guide	Covid-19 Fund Spends GH32m
17/7/2020	Daily Guide Network	Daily Guide	Covid-19 Trust Fund Gives To 11 Institutions
20/7/2020	Gertrude Anka Nyavi	Graphic online	HELEH: Africa foundation launches campaign against covid-19 stigma
20/7/2020	Graphic.com.gh	Graphic online	STRATCOM Africa releases covid-19 related cartoon against stigma and discrimination

**DD/MM/YYYY**	**Author Affiliation**	**Newspaper**	**Title**
20/7/2020	Graphic.com.gh	Graphic online	Invest in Africa and mastercard foundation join forces to assist covid-hit companies in Ghana, Kenya and Senegal
8/07/2020	Joshua Bediako Koomson	Graphic online	M Y Caeser company limited present immune boosters to parliament house
8/07/2020	Graphic Online	Graphic online	Noguchi receives US$100,000 support from ATC Ghana to fight COVID-19
9/07/2020	Daily Guide Network<br><br>Melvin Tarlue.	Daily Guide Network	Gov't Considering Covid19 Inspection Regime For Companies
9/07/2020	Graphic.com.gh	Graphic online	COVID-19: Beko Electronics supports Adentan Health Directorate
10/07/2020	Daily Guide Network<br><br>Issah Mohammed-.	Daily Guide Network	Kasoa Drivers Exploit Passengers
10/07/2020	Daily graphic	Graphic online	NPP-USA donates GH¢130,000 to support fight against COVID-19
10/07/2020	Graphic.com.gh	Graphic online	COVID-19: StarTimes donates 10,000 face masks to Police
12/07/2020	Daniel Kenu	Graphic online	Kumasi: COVID-19 Private Fund donates to Cheshire Home and Children's Home
03/07/2020	Daily Guide Network<br><br>Melvin Tarlue	Daily Guide Network	Sethi Brothers Ghana Donates Ghc 200,000 To Foreign Ministry
5/07/2020	Rosalind K. Amoh	Graphic online	GMA cries out over increasing COVID-19 spread as 3 top doctors succumb to disease
06/07/2020	Daily Guide Network	Daily Guide Network	Mantrac Ghana Donates $10,000 To Covid-19 Trust Fund

DD/MM/YYYY	Author Affiliation	Newspaper	Title
07/07/2020	Graphic business	Graphic online	MTN Ghana zero-rates CoRe website
08/07/2020	Daily Guide Network<br><br>Ernest Kofi Adu	Daily Guide Network	Transport Fares Up By 15%
24/7/2020	Daily Guide Network	Daily Guide Network	New Crystal Provides Support Services To Organizations
24/7/2020	Getrude Ankah Nyavi	Graphic online	Covid 19: Tobinco produces hydroxychloroquine locally
24/7/2020	Desmond Kofi Tawiah	Graphic online	COVID-19: Omni Speciality Products donates to Noguchi
24/7/2020	Daily graphic	Graphic online	CH foundation donates $100,000 to covid-19 private sector fund
26/7/2020	Enoch Darfah Frimpong	Graphic online	Local firms produce, distribute 6.2 million nose mask
26/7/2020	Graphic.com.gh	Graphic online	Joana Gyan foundation to Donate PPE to Wasa Agona Amenfi
28 Jul 2020	Graphic<br><br>Enoch Darfah Frimpong	Graphic	Transport operators agree to reduce transport fares by 10 percent from Aug 1
28 Jul 2020	Graphic<br><br>Ruby Delanyo Buafor	Graphic	Reduce transport fares — COPEC
28 Jul 2020	Graphic<br><br>Kwame Larweh	Graphic	Top Archive donates to COVID-19 Trust Fund
31 Jul 2020	Graphic<br><br>Maclean Kwofi	Graphic	Ghana's e-learning initiative gains momentum

DD/MM/YYYY	Author Affiliation	Newspaper	Title
02 Aug 2020	Daily Guide Network<br><br>Jamila Akweley Okertchiri	Daily Guide Network	UG, SOFT Develop Chlorine Disinfectant To Aid Covid-19 Fight
13/08/2020	News Desk	Graphic online	Sambus Geospatial assists Health Service with location software
16/08/2020	Graphic.com.gh	Graphic online	COVID-19: Lions Club donates to Madina Polyclinic
17/08/2020	Daily Guide Network	Daily Guide Network	MultiChoice Ghana Donates PPE To KATH, Ga East Hospita
17/08/2020	Graphic.com.gh	Graphic online	Philanthropic group supports three orphanages to fight COVID–19
18/08/2020	Graphic.com.gh	Graphic online	Zipline lauded for efforts in fighting COVID-19
20/08/2020	Daily graphic	Graphic online	COVID-19 Trust Fund presents GH¢6.8m to Infectious Diseases Centre G
03/08/2020	Graphic<br><br>GRAPHIC.COM.GH	Graphic	How the COVID-19 National trust Fund spent GHC32million
5 August 2020	Daily Guide Network<br><br>Melvin Tarlue	Daily Guide Network	GIZ Donates Covid-19 Support Items To Agric Ministry
5 August 2020	Daily Guide Network<br><br>Jamila AkweleyOkertchiri	Daily Guide Network	VAMED Engineering Supports Covid-19 Fight
6 August 2020	Graphic<br><br>Samuel Tei Adano	Graphic	44 health facilities benefit from National COVID-19 Trust Fund
7 August 2020	Graphic<br><br>Graphic.com.gh	Graphic	GCB to donate GHC100,000 to Upper West Health directorate
11/08/2020	Graphic<br><br>Emmanuel Baah	Graphic	Asanteman Europe gives to 3 institutions
05/09/2020	Daily Guide Network	Daily Guide Network	GHS Organizes Covid19 Quiz For Kids

DD/MM/YYYY	Author Affiliation	Newspaper	Title
	Melvin Tarlue		
02/09/2020	No Author	Cifi Fm Online	Millennium Promise Alliance partners GHS to launch paperless Health declaration form at airport
31/08/2020	Citi Newsroom	Citi Newsroom	Beiersdorf Ghana donates 50,000 Nivea skin care products to GHS
31/08/2020	Graphic.com	Graphic	ATOA Ghana organises training for Travel Agents
22/09/2020	Graphic	Daily Graphic	Stanbic Bank Ghana donates additional COVID-19 test kits to Ghana Health Service
17/09/2020	Maclean Kwofi	Graphic Online	Guinness supports bar operators with GH¢10m
14/09/2020	Daily Guide Network<br><br>Issah Mohammed	Daily Guide Network	MoH Receives 100,000 Face Masks
04/04/2020	Daily Guide Network<br><br>Melvin Tarlue	Daily Guide Network	Guinness Ghana Announces Ghc 10m Covid-19 Support For Customers
08/09/2020	Graphic Business	Graphic	AGRA mobilises private sector for agricultural investment opportunities

225 total
